# Massive gene presence-absence variation shapes an open pan-genome in the Mediterranean mussel

**DOI:** 10.1186/s13059-020-02180-3

**Published:** 2020-11-10

**Authors:** Marco Gerdol, Rebeca Moreira, Fernando Cruz, Jessica Gómez-Garrido, Anna Vlasova, Umberto Rosani, Paola Venier, Miguel A. Naranjo-Ortiz, Maria Murgarella, Samuele Greco, Pablo Balseiro, André Corvelo, Leonor Frias, Marta Gut, Toni Gabaldón, Alberto Pallavicini, Carlos Canchaya, Beatriz Novoa, Tyler S. Alioto, David Posada, Antonio Figueras

**Affiliations:** 1 Department of Life Sciences, Università degli Studi di Trieste, Via Licio Giorgieri 5, 34127 Trieste, Italy; 2 Instituto de Investigaciones Marinas (IIM - CSIC), Eduardo Cabello, 6, 36208 Vigo, Spain; 3 CNAG-CRG, Centre for Genomic Regulation (CRG), Barcelona Institute of Science and Technology (BIST), Baldiri i Reixac 4, 08028 Barcelona, Spain; 4 CRG - Centre for Genomic Regulation, Doctor Aiguader, 88, 08003 Barcelona, Spain; 5 Department of Biology, Università degli Studi di Padova, Via Ugo Bassi 58/B, 35131 Padova, Italy; 6 Universitat Pompeu Fabra (UPF), 08003 Barcelona, Spain; 7 Department of Biochemistry, Genetics and Immunology, University of Vigo, 36310 Vigo, Spain; 8 Norce Norwegian Research Centre AS, Bergen, Norway; 9 New York Genome Center, New York, NY 10013 USA; 10 ICREA, Pg. Lluís Companys 23, 08010 Barcelona, Spain; 11 Current address: Barelona Supercomputing Centre (BSC-CNS) and Institute for Research in Biomedicine (IRB), 08034 Barcelona, Spain; 12 Anton Dohrn Zoological Station, 80121 Villa Comunale, Naples, Italy; 13 Biomedical Research Center (CINBIO), University of Vigo, 36310 Vigo, Spain; 14 Galicia Sur Health Research Institute, 36310 Vigo, Spain

**Keywords:** Mussel, Bivalve, Pan-genome, Presence-absence variation, Structural variants, Hemizygosity, Dispensable gene, Phylome, Innate immunity, Antimicrobial peptides

## Abstract

**Background:**

The Mediterranean mussel *Mytilus galloprovincialis* is an ecologically and economically relevant edible marine bivalve, highly invasive and resilient to biotic and abiotic stressors causing recurrent massive mortalities in other bivalves. Although these traits have been recently linked with the maintenance of a high genetic variation within natural populations, the factors underlying the evolutionary success of this species remain unclear.

**Results:**

Here, after the assembly of a 1.28-Gb reference genome and the resequencing of 14 individuals from two independent populations, we reveal a complex pan-genomic architecture in *M. galloprovincialis*, with a *core* set of 45,000 genes plus a strikingly high number of *dispensable* genes (20,000) subject to presence-absence variation, which may be entirely missing in several individuals. We show that dispensable genes are associated with hemizygous genomic regions affected by structural variants, which overall account for nearly 580 Mb of DNA sequence not included in the reference genome assembly. As such, this is the first study to report the widespread occurrence of gene presence-absence variation at a whole-genome scale in the animal kingdom.

**Conclusions:**

*Dispensable* genes usually belong to young and recently expanded gene families enriched in survival functions, which might be the key to explain the resilience and invasiveness of this species. This unique pan-genome architecture is characterized by dispensable genes in accessory genomic regions that exceed by orders of magnitude those observed in other metazoans, including humans, and closely mirror the open pan-genomes found in prokaryotes and in a few non-metazoan eukaryotes.

## Background

The Mediterranean mussel *Mytilus galloprovincialis* Lamarck, 1819 (Bivalvia, Mytilida), a member of the *M. edulis* species complex, is an edible cosmopolitan bivalve mollusk and an important seafood product in Europe and China. This shellfish has been consumed by humans since 6000 BC, and its global production currently exceeds 400 thousand tons per year [1]. Due to its invasive nature, this species has spread far beyond its native range, and it is considered a worldwide threat for autochthonous bivalve populations [2]. Like many other marine invertebrates, mussels have separate sexes and reproduce by broadcast spawning. Upon the release of gametes into the open water, and after fertilization, larvae can travel long distances carried by the oceanic currents [3]. Metamorphosis takes place during planktonic life, which ends after 1 to 2 months—depending on water temperature and food availability—with the settlement of juveniles and the start of the sessile adult life. Mussel beds are therefore usually composed by genetically heterogeneous individuals derived from large, randomly mating populations of different geographical origin. While genetic introgression in mussels has been broadly documented [4], a number of natural and genetic barriers concur in maintaining the genetic discontinuities observed both between different species belonging to the *M. edulis* species complex and between different lineages belonging to the same species [5].

Due to their filter-feeding habits, mussels are constantly exposed to a wide range of potentially pathogenic microorganisms, biotoxins, and anthropogenic pollutants. However, they display a remarkable resilience to stress and infections, can evolve novel traits in response to predation within a few generations [6], and have the ability to rapidly adapt to environmental changes, such as ocean acidification [7]. Moreover, mussels are capable of significant bioaccumulation [8], without experiencing the massive mortalities often seen in other farmed bivalves [9, 10].

Although *M. galloprovincialis* displays a morphologically conserved karyotype compared with other mussels and has not undergone known whole-genome duplication or allopolyploidization events [11], it shares with other bivalves a relatively large and complex genome, characterized by high heterozygosity and numerous mobile elements [12–17]. These factors posed a serious challenge to previous assembly efforts, which resulted in extremely fragmented genome sequences for this species [18, 19] which, unlike the congeneric *Mytilus coruscus* [20] and a few other mussel species [15], still lacks a highly contiguous reference genome assembly.

The remarkable level of intraspecific sequence diversity which characterizes several bivalve immune gene families [21], together with the recent implication of high standing genetic variation within natural populations in the extraordinary capability of environmental adaptation of *M. galloprovincialis* [7], stimulate further investigation about the role played by the genomic complexity of this species in explaining its invasiveness and resilience [18]. While a small but growing number of studies connected gene presence-absence variation (PAV) to the generation of this molecular diversity in a few gene families encoding antimicrobial peptides (AMPs) [22–25], it is presently unclear whether and to what extent this phenomenon is widespread in bivalve genomes.

Gene PAV is intimately linked to the pan-genome concept, which can be defined as a genome that includes a set of *core* genes found in all individuals and *dispensable* genes that are entirely missing in some individuals within a population [26]. Pan-genomes have been extensively studied in prokaryotes and viruses, where a simple genome architecture and frequent lateral gene transfer events facilitate the acquisition of accessory genomic sequence that may provide an evolutionary advantage in the colonization of new ecological niches or in the interaction with the host [27–29]. In Eukaryotes, pan-genomes have been occasionally reported in plants, fungi, and microalgae, where they have been often associated with the development of phenotypic traits linked with environmental adaptation, resistance to diseases, and intraspecific differentiation [30–36]. Although a few studies have recently extended the pan-genome concept to the animal kingdom, to the best of our knowledge, these have so far mostly linked the *dispensable* fraction of animal genomes with intergenic regions, bringing little evidence in support of the association between accessory genomic regions and gene PAV with adaptation [37–39].

We here report an improved, highly contiguous reference genome assembly for *M. galloprovincialis*, obtained from the sequencing of a single female individual (nicknamed *Lola*) and provide evidence in support of massive gene PAV through the analysis of whole-genome resequencing (WGR) data from 14 additional individuals. The widespread observation of the gene PAV phenomenon, which involves 20,000 protein-coding genes significantly enriched in functions related with survival, provides strong evidence in support of the presence of an open pan-genome in the Mediterranean mussel.

## Results

### An overview of the mussel reference genome

**Fig. 1:**
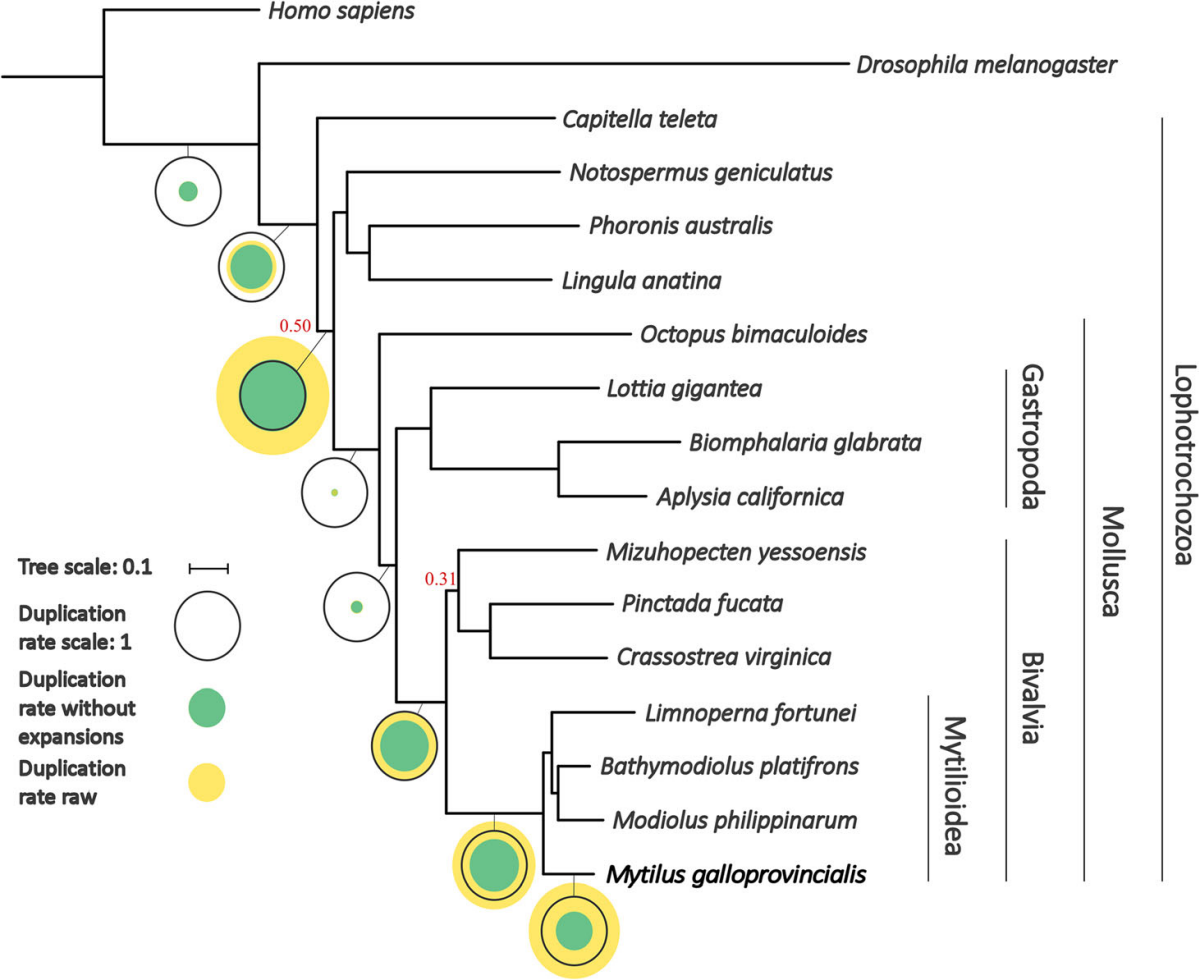
Species tree obtained from the concatenation of 177 widespread single-gene families. In bold, we highlight the genome sequenced in this study. All branches were very well supported (aLRT > 0.99) except those with a number in red. In the tree, Bivalvia is a sister lineage to Gastropoda, with both molluscan classes forming a clade sister to Cephalopoda. Mollusca appears as a sister branch of a clade containing Phoronida (*Phoronis*), Nemertea (*Notospermus*), and Brachiopoda (*Lingula*) with low support (0.50). Open circles represent duplication rates (genome-wide average number of duplications per gene). Yellow circles represent the estimated duplication rates before removing large expansions consisting of more than 20 paralogs appearing in a single node. Green circles represent the duplication rates obtained after the removal of such events. Black circles serve as a scale and correspond to a duplication rate of 1

Our multi-step hierarchical de novo assembly strategy (Additional file 1: Data Note 1) resulted in a 1.28-Gb genome, of slightly smaller size compared to cytogenetic estimates [40], but of higher quality and contiguity compared to previous attempts [18, 19] (10,577 scaffolds; contig N50 = 71.42 kb; scaffold N50 = 207.64 kb). This genome shared some typical features of other bivalves, such as a low GC content (32%) and a widespread presence of repeats (43% of the assembly), but it was particularly rich in both protein-coding (60,338) and non-coding (75,973) genes. While these figures largely exceed those observed in most sequenced bivalve species [12, 14], they closely matched the numbers recently reported in the king scallop [17] and in the zebra mussel [41] (Additional file 1: Data Note 3). The reconstruction of the evolutionary relationships among *M. galloprovincialis* and 15 selected lophotrochozoan species [42–45], followed by an analysis of gene family trees [46], revealed that this large gene repertoire is the result of multiple lineage-specific duplication events that took place after the split between *Mytilus* and the rest of Mytilida (Fig. 1, Additional file 1: Data Note 5). Most mussel protein-coding genes were functionally annotated based on sequence similarity (56.17%) and were supported by transcriptomic evidence (78.70%). However, more than 5000 genes belong to recently acquired gene families specific of the *Mytilus* lineage, with uncharacterized function (Additional file 1: Data Note 20) [47].

### A genome characterized by widespread heterozygosity and hemizygosity

**Fig. 2:**
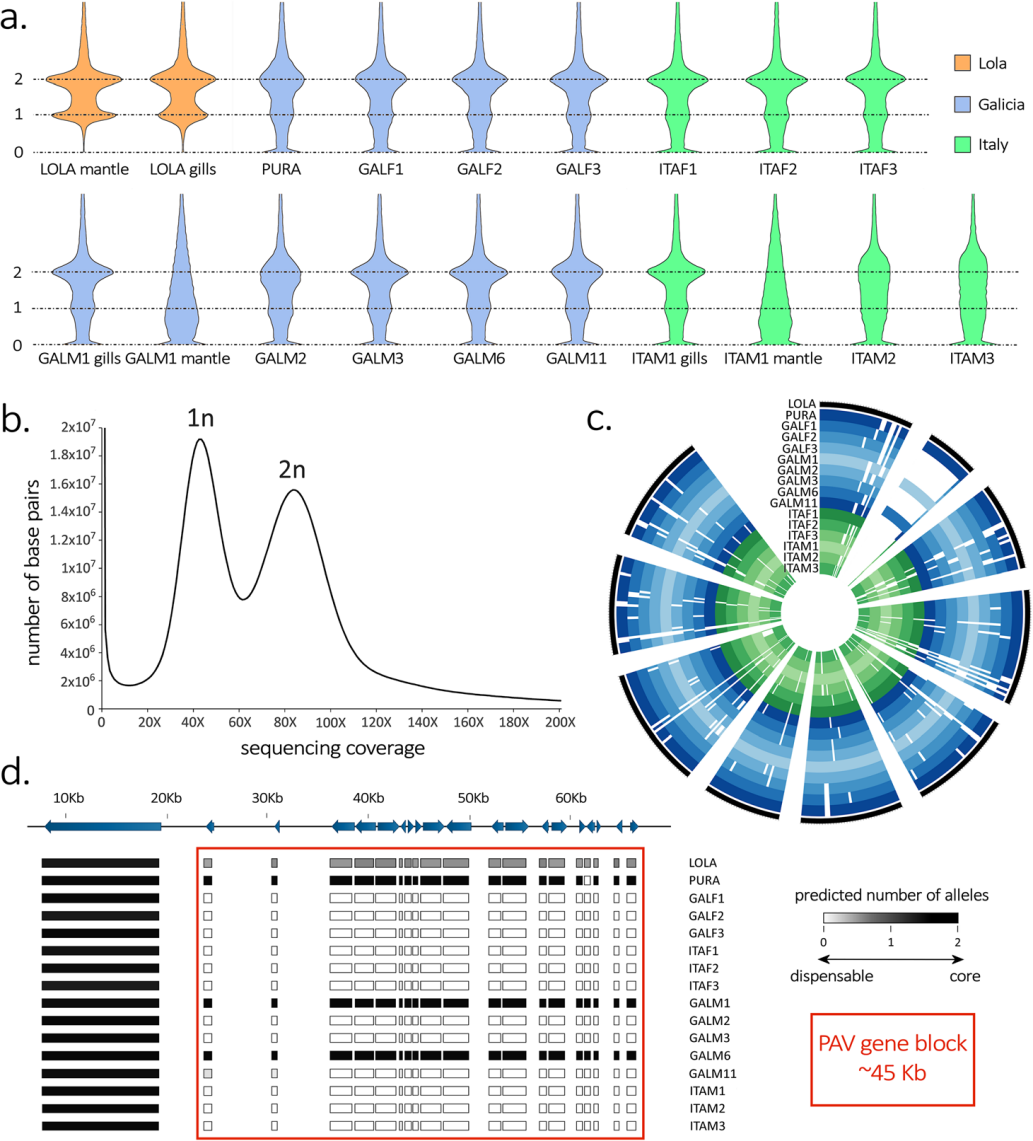
**a** Violin plots displaying the “per gene” coverage of the protein-coding genes annotated in the mussel genomes, normalized by the expected haploid mussel genome size, calculated based on the mapping of Illumina PE libraries, and obtained either from the mantle or gills tissue.  “0”, “1” and “2” indicate the number of alleles per individual. **b** Summary of the “per base” coverage of the *Lola* assembly (evaluated by the mapping of Illumina PE reads obtained from the mantle tissue). Two main peaks of coverage are clearly visible, corresponding to the hemizygous (41.5X) and homozygous (83X) peaks of coverage based on genome size estimates. The peak located at 0 indicates approximately 70 Mb of genome assembly which did not achieve any mapping based on *Q* ≥ 60. **c** Circus plot summarizing the presence-absence variation phenomenon on the nine longest genome scaffolds, plus the scaffold 02822, which contains a large fraction of *dispensable* genes. Italian and Galician mussel genomes are indicated with green and blue shades of color, respectively. Full and empty circles indicate present and absent genes, respectively. Scaffold names, reported in clockwise order, are: s00009, s029833, s00073, s00003, s00002, s00060, s00247, s00011, s00241 and s00029. **d** Detail of presence-absence variation in the genomic scaffold 02822; note that this scaffold contains a single *core* gene and a large (about 45 kb) block of *dispensable* genes, which are only found in four out of the analyzed genomes (including *Lola*, where the gene block is present in a single copy only). Gene IDs are, from left to right, as follows: MGAL10A041721, MGAL10A025495, MGAL10A021236, MGAL10A038341, MGAL10A006169, MGAL10A040493, MGAL10A046398, MGAL10A002899, MGAL10A052538, MGAL10A016640, MGAL10A080632, MGAL10A080632, MGAL10A050728, MGAL10A069289, MGAL10A086703, MGAL10A046961, MGAL10A008710, MGAL10A090544, MGAL10A069343, MGAL10A077743 and MGAL10A011823

The contribution of heterozygosity to the overall intraspecific genomic variation was estimated in *Lola, Pura* (a female individual subject of a previous assembly effort [18]), and in the 14 resequenced genomes by analyzing only those regions shared by all individuals. The average heterozygosity rate observed across individuals was 1.73 ± 0.24%, indicating that the mussel genome harbors a very high density of single-nucleotide polymorphisms (SNPs), 12–22-fold higher than the human genome [48–50] (Additional file 1: Data Note 6). This value is in line with previous reports [4, 7] and with genomic evidence collected in other mytilids [15, 16]. However, while this high heterozygosity rate may seem rather large when compared to other animal species, it does not appear to be the main source of intraspecific genomic diversity in *M. galloprovincialis*. Indeed, structural variation, and large insertion/deletion polymorphisms in particular, appear to be a key aspect in the genome of this species. In spite of the assembly strategy we adopted, aimed at the removal of sequence derived from alternative haplotypes, the haploid reference genome assembly still contained a high fraction (36.78%) of sequence with low coverage. The bimodal distribution of the read mapping coverage in *Lola* (Fig. 2b) clearly shows that such regions are found in a hemizygous state, i.e., they are present in only one of the two homologous chromosomes.

### Massive gene presence-absence variation

The hemizygous fraction of the mussel genome does not only include intergenic regions, but also contains nearly one-third of the protein-coding genes annotated in the reference genome, as well as a significant fraction of non-coding genes (Additional file 1: Data Note 8 and 9). Even more surprisingly, our analyses revealed that 24.25% of the protein-coding genes and 16.71% of the non-coding genes were entirely missing in at least one of the resequenced genomes, i.e., they were subject to gene PAV [51, 52] (Fig. 2a–c). On average, each individual lacked 4829 (8.01%) protein-coding genes and 3744 (5.12%) non-coding genes found in *Lola*.

Unlike the 45,518 *core* protein-coding genes found in homozygous genomic regions in *Lola* and in all the resequenced genomes, the 14,820 genes subject to PAV are *dispensable* and often associated with hemizygous genomic regions, i.e., they can be present in either one, two, or in neither of the two homologous chromosomes of the different mussels analyzed. Indeed, most of the genes encoded by hemizygous genomic regions in *Lola* either displayed a sequencing coverage consistent with hemizygosity or were entirely absent in the resequenced genomes (58.50% and 23.23% on average, respectively, Additional file 1: Fig. S56). On the other hand, the overwhelming majority (98.05%) of the genes present in homozygous genomic regions in *Lola* were present in all the resequenced genomes, in 85.46% of cases with a sequencing coverage also consistent with homozygosity (Additional file 1: Fig. S57).

**Fig. 3:**
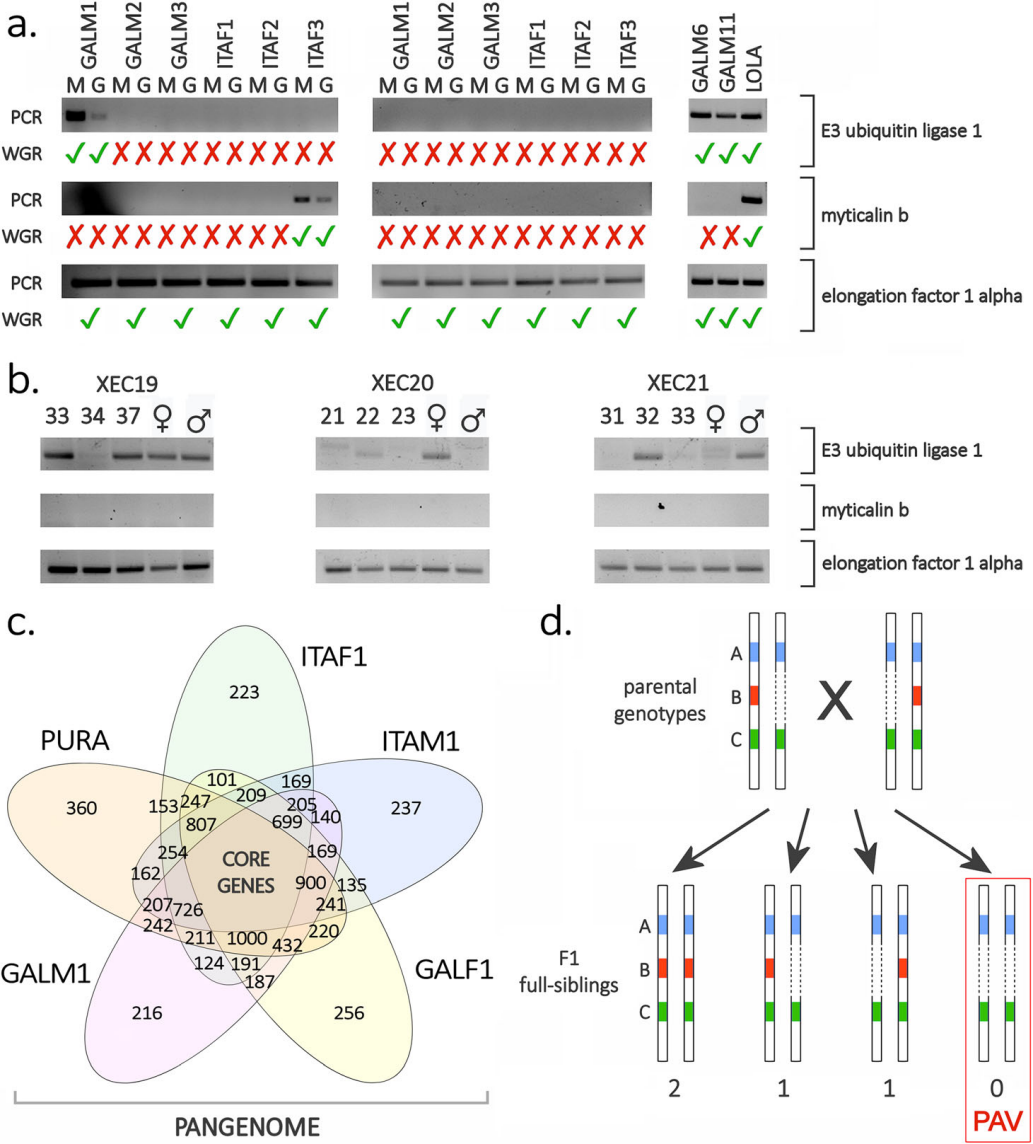
**a** Validation of the presence-absence variation phenomenon by PCR, carried out on the genomic DNA extracted from the mantle (M) or gills (G) of the 14 mussel individuals subjected to whole-genome resequencing. In *Lola*, GALM6 and GALM11, genomic DNA was extracted from the mantle tissue only. One *core* gene (elongation factor 1 alpha) and two *dispensable* genes (E3 ubiquitin ligase 1 and myticalin B1) were tested. Tick and cross symbols indicate expectations based on the in silico analysis of whole-genome resequencing (WGR) data. **b** Observation of the presence-absence variation phenomenon by PCR carried out in 3 full-sib mussels obtained from a controlled cross (parents were also tested and are indicated by ♂ and ♀, respectively). One *core* gene (elongation factor 1 alpha) and two *dispensable* genes (E3 ubiquitin ligase 1 and myticalin B1) were tested. XEC19, XEC20, and XEC21 indicate the three mussel families subjected to this investigation. The original photographs of the agarose gels used for the preparation of this figure and technical details about these experiments are available in Additional file 1: Data Note 12. **c** Structure of the mussel pangenome, as exemplified by a Venn diagram representing the overlap between the gene sets found in *Pura* (a female individual previously sequenced by Murgarella and colleagues [18]) and in four resequenced genomes, i.e., ITAF1, ITAM1, GALF1, and GALM1 (note that all these genes are present in *Lola*). Genes shared by all individuals define the *core* genome, whereas genes shared by some, but not all, individuals are *dispensable*. The entire complement of *core* and *dispensable* genes defines the mussel pangenome. **d** Schematic overview of the possible origin of a gene presence-absence variation phenomenon. A cross between two parents carrying two *core* genes (A and C) and one *dispensable* gene (B) is depicted. In this case, both parents carry a single copy of the *dispensable* gene (i.e., the *dispensable* gene is present in a hemizygous genomic region). Based on Mendelian inheritance, PAV should be observed in 25% of the offspring produced by this cross. “0”, “1” and “2” indicate the expected number of copies of the dispensable gene

We ruled out the possibility that our observations were hampered by biases or confounding factors linked with the library preparation, sequencing, or bioinformatics analyses through a series of additional tests. First and foremost, the visual inspection on agarose gel of PCR amplification bands resulting from the analysis of twelve *dispensable* genes plus five *core* genes revealed a complete overlap between in silico predicted and experimentally observed PAV patterns (Fig. 3a, Additional file 1: Data Note 12). A similar PCR-based approach was extended to three independent families of full-sib mussels comprising the two parents and three first-generation offspring each. This allowed us to bring further support to the hypothesis that the dispensable genes are encoded by hemizygous genomic regions and that they follow a Mendelian mode of inheritance (Fig. 3d). Moreover, the presence of dispensable genes in hemizygous genomic regions was confirmed by the analysis of mapping data derived from a second round of sequencing of *Lola* obtained from a different tissue (gills, see Additional file 1: Fig. S33-S34), and the possible effect of mapping artifacts was excluded through computational simulations (Additional file 1: Data Note 10).

### The mussel pan-genome

Our recursive pan-genome reassembly strategy (summarized in Additional file 1: Fig. S66), paired with a strict decontamination pipeline (Additional file 1: Fig. S67), led to the generation of 267,538 contigs unambiguously taxonomically assigned to *M. galloprovincialis*, accounting for 578.74 Mb sequence data not present in *Lola*. Consistently with the high contiguity observed for some *dispensable* genes contained in large (up to 30 kb) hemizygous genomic regions in *Lola* (Fig. 2d, Additional file 1: Data Note 17), several large assembled pan-genomic contigs had protein-coding potential. This process brought the cumulative size of the mussel pan-genome assembly to 1.86 Gb, and the total number of annotated protein-coding genes to 65,625 (20,106 of which are *dispensable*). On average, each resequenced genome included 1974 out of the 5286 newly annotated protein-coding genes. Overall, we estimate that each resequenced genome lacked, on average, 8141 *dispensable* genes found in the mussel pan-genome (Additional file 1: Data Note 15).

### Characterization and functional enrichment of *dispensable* genes

Although mussel *dispensable* genes generally display a shorter ORF length and a lower gene architecture complexity than *core* genes, they mostly retain signatures of functionality, which include the presence of conserved regulatory elements and the lack of significant GC or codon usage bias (Additional file 1: Data Note 17). While *dispensable* genes display, on average, expression levels 3× lower than *core* genes, nearly 60% of them are supported by mild or strong transcriptional evidence, accounting for 3–10% of the global transcriptional activity, depending on the tissue considered (Additional file 1: Data Note 16). They are also on average evolutionarily younger, subject to an increased lineage-specific duplication rate and four times more likely to be taxonomically restricted than *core* genes (Additional file 1: Data Note 19–20).

**Fig. 4:**
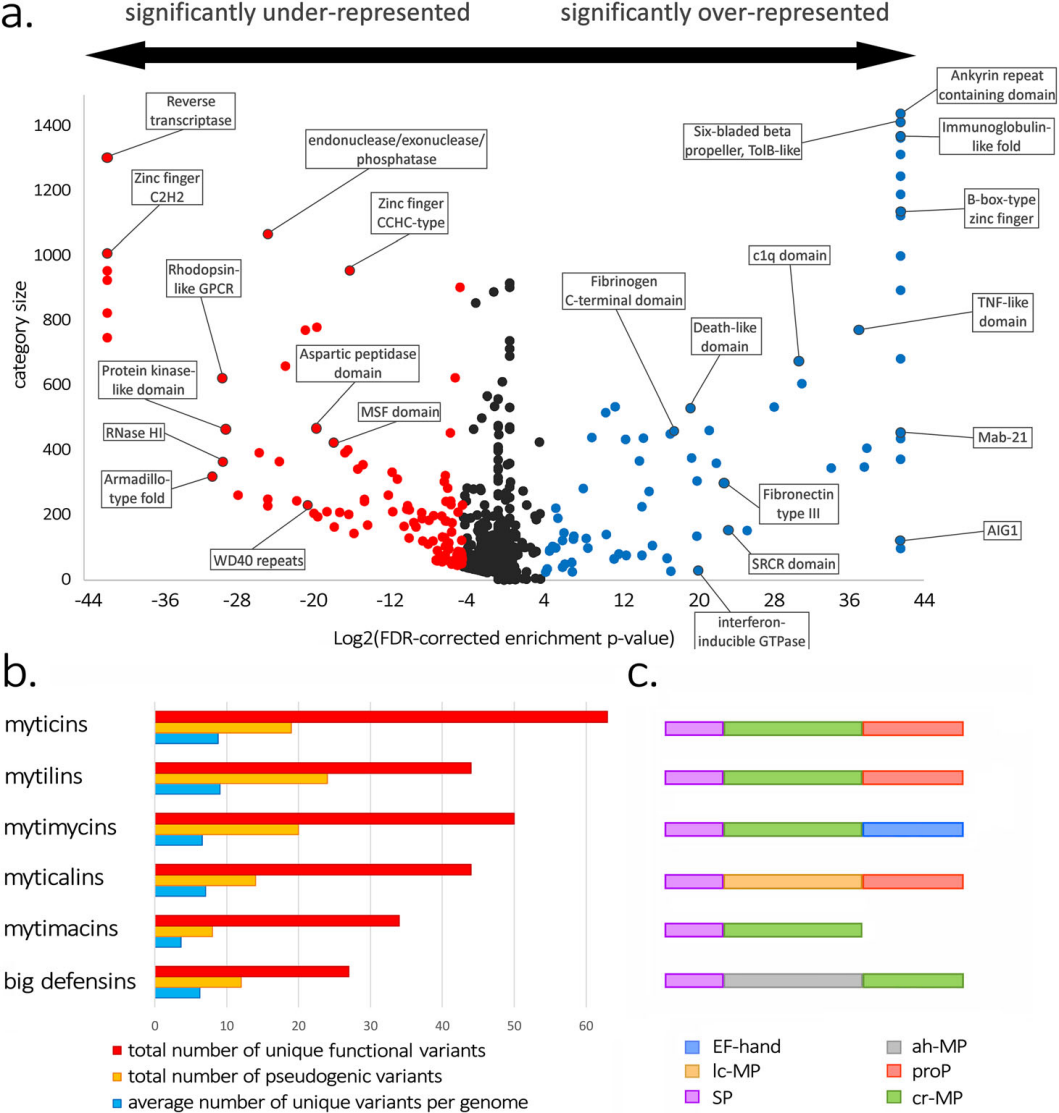
**a** Correlation between the abundance of conserved protein domains (*Y* axis) and their level of enrichment in the *dispensable* mussel genes (*X* axis). Each dot represents a conserved protein domain, with specific examples highlighted within boxes. Domains shown in red and blue are under- and over-represented, respectively, in the *dispensable* gene set compared to the *core* genome. **b** Total number of unique potentially functional sequence variants and pseudogenes, and average number of unique functional variants per genome for the six families of antimicrobial peptides analyzed (myticins, mytilins, mytimycins, myticalins, mytimacins, and big defensins). **c** Schematic organization of the precursor proteins of the six AMP families displayed in panel **b**. SP: signal peptide; ProP: propeptide region; EF-hand: EF-hand domain; lc-MP: linear cationic mature peptide; ah-MP: alpha helical mature peptide; cr-MP: cysteine-rich mature peptide

We identified several mussel gene families significantly more prone to PAV than expected by chance (Fig. 4a). The functional annotation of the *dispensable* genes found both in the reference genome and in the recursively re-assembled pan-genomic contigs revealed an enrichment in functions related to survival. These may be provided by proteins with marked protein- or carbohydrate-binding properties (e.g., pattern recognition receptors like C1qDC proteins, FReDs, and Ig domain-containing proteins), involved in apoptosis pathways (e.g., DEATH and BIR), or playing a role in immune signaling (e.g., interferon-inducible and IMAP GTPases) (Additional file 1: Data Note 18).

Mussel gene families encoding antimicrobial peptides (AMPs) were also subject to massive gene PAV. Although several dozen different sequence variants were identified for each AMP family in the resequenced genomes, each individual mussel possessed a unique combination of a small number of variants, with very little overlap with other mussels from the same population (Fig. 4b, Additional file 1: Data Note 21). On the other hand, other gene families were significantly under-represented among *dispensable* genes. Notably, these included genes encoding transposable elements (and therefore found in multiple copies in the genome), such as reverse transcriptase and RNase H-like proteins, or with housekeeping functions (e.g., protein kinases and G protein-coupled receptors).

## Discussion

A pan-genome contains a set of *core* genes present in all individuals of the same species, which are fundamental for survival, and *dispensable* genes, which are only found in a subset of the individuals, and usually have accessory functions [26]. The expansion of pan-genomic studies to Eukaryotes has later extended this concept to intergenic genomic regions and to the activity of mobile elements [37, 39, 50, 53]. However, we will refer here to the original pan-genome definition, associated with the accessory functions of *dispensable* genes and with the acquisition of the ability to quickly respond to selective pressure [27] and to colonize new ecological niches [28]. The widespread occurrence of PAV in mussels is most certainly consistent with the gene-centric definition provided by Medini and colleagues [26] and reveals an “open” pan-genomic architecture with a high rate of *dispensable* to core *genes*, i.e., 1:3 (Fig. 3c).

The small size, simple organization and fast gene gain, loss, and horizontal transfer rates of bacterial and viral genomes [54–56] can explain the presence of a large number of *dispensable* genes in these organisms. However, pan-genomes have been also occasionally reported in eukaryotes, such as plants, fungi, and microalgae, where they may represent an unearthed source of intraspecific genomic diversity [57]. For example, in some cultivated crops, *dispensable* genes contribute significantly to the development of agronomic traits [30–32], such as improved resistance to disease [36]. Another key example of the adaptive importance of the pan-genome architecture is provided by some fungi, whose pathogenic potential, antimicrobial resistance, and host immune system avoidance are fueled by *dispensable* genes [33, 34, 58]. In the context of ecological adaptation, the cosmopolitan oceanic distribution and ability to thrive in different habitats of the coccolithophore *Emiliania huxley* can be explained by the acquisition of accessory metabolic activities provided by *dispensable* genes [35].

In spite of the growing number of reports of pan-genomes in eukaryotes, large-scale studies have been restricted to a few vertebrate species [39], targeting in particular human populations [37, 38, 50]. Moreover, the presence of accessory genomic regions not included in metazoan reference assemblies has never been associated with the massive occurrence of gene PAV, and the general impact of this phenomenon on animal intraspecific diversity has always been presumed to be minimal and in some cases deleterious [59].

Our observations, strongly supported by both experimental (Additional file 1: Data Note 12) and computational evidence (Additional file 1: Data Note 13), revealed that the *dispensable* fraction of the mussel pan-genome exceeds by approximately 5 and 15 times that of *Sus scrofa* and *Homo sapiens*, respectively [37, 39]. Moreover, while just 240 genes (i.e., 1.17% of the total) are presumably *dispensable* in humans [38], we show that 25% of mussel genes are subject to PAV and that each of the individual mussels resequenced in this study lacked on average 8141 *dispensable* genes identified in the pan-genome, pointing out that the fraction of protein-coding genes affected by this phenomenon in mussel is 20 times higher than in humans (Additional file 1: Data Note 22). To the best of our knowledge, PAV has been only marginally explored in bivalves as a phenomenon linked to a few gene families involved in immune functions, such as big defensins in *Crassostrea gigas* [22, 24] and myticins and myticalins in *M. galloprovincialis* [23, 25]. Therefore, this is the first study to report the widespread occurrence of gene PAV at a whole-genome scale in the animal kingdom.

Besides the 60,338 protein-coding genes present in *Lola* and the 5286 protein-coding genes associated with the recursively re-assembled contigs (Additional file 1: Data Note 15), the mussel pan-genome might include several thousand additional dispensable genes in natural populations which still remain unexplored (Additional file 1: Data Note 22). This open pan-genomic architecture is strongly supported by the recursive reassembly of 580 Mb DNA sequence not present in the reference assembly, by the observation that several *dispensable* genes were only observed in single individuals, and by the fact that the pan-genome assembly growth curve was far from reaching saturation (Additional file 1: Data Note 14).

The *Mytilus* genus has a complex evolutionary history, characterized by extensive gene flow among congeneric species, a process which is still ongoing in mosaic hybrid zones [60–62]. However, the analysis of nuclear and mitochondrial genetic markers ruled out the possibility that our resequenced individuals were hybrids between *M. galloprovincialis* and other *Mytilus* species (Additional file 1: Data Note 7), which suggest that *dispensable* genes are unlikely to be recently introgressed allelic variants that cannot be mapped to the reference genome due to their sequence divergence (Additional file 1: Data Note 10). While genetic admixture among contemporary mussel species cannot explain the mussel pan-genome architecture, the role of ancient hybridization and homologous recombination between ancestral *Mytilus* species remains to be investigated, as similar processes have been identified as the key drivers of PAV in plants [63]. Similarly, the possible role of transposable elements in the origin and spread of the PAV phenomenon [53] will be fully elucidated only with the availability of a chromosome-scale assembly (Additional file 1: Data Note 17.4).

Regardless of the origin of mussel *dispensable* genes, their absence or presence in a hemizygous or homozygous state in the mussel genome suggests that the PAV phenomenon might be strictly dependent on the matching between paternal and maternal chromosomes during the fertilization process and that *dispensable* genes might have Mendelian inheritance (Fig. 3d). This hypothesis was confirmed by the observation of F1 proportions fully compatible with a Mendelian pattern in full-sibs resulting from a controlled cross between individuals showing PAV at the E3 ubiquitin ligase 1 gene (Fig. 3b).

Our finding that a large fraction of the mussel genome is in a single-allele state is congruent with the presence of chromosome structural variation [64] and with the significant intra-individual and inter-population variation in nuclear DNA content reported in previous cytogenetic studies [65]. This also mirrors the situation previously described in other metazoans with high intraspecific genome diversity, such as the roundworm *Caenorhabditis brenneri* and the ascidian *Ciona savignyi*, which have genomes characterized by significant structural variations and frequent polymorphic indels [66, 67]. The very high amount of intraspecific genomic diversity revealed in our study may come at the cost of interfering with conventional homologous chromosome pairing, recombination, and segregation during meiosis.

The observation of highly skewed coverage profiles in the sequencing libraries from the gonadal tissue of some (but not all) male mussels, regardless of the stage of sexual maturation, may support this hypothesis (Additional file 1: Data Note 23). These results, confirmed by a second independent round of resequencing, were not obtained in non-reproductive tissues (i.e., gills) or in female individuals (Fig. 2a). We suspect that this observation may be the result of a significant presence of aneuploid gametes, potentially generated by an aberrant meiotic process linked with the high structural divergence between homologous chromosomes. Several studies have reported the presence of strong genetic barriers in *Mytilus*, acting both between and within species. Although intrinsic post-zygotic selection has been invoked as one of the most likely mechanisms underpinning the preservation of mosaic hybrid zones [62, 68], the nature of this process still remains to be elucidated. Here, we postulate that the reduced fertility of the offspring produced by individuals carrying “structurally incompatible” chromosomes may be key for explaining post-zygotic selection and the maintenance of the pan-genome architecture in mussels.

Whether the pan-genomic architecture of the mussel genome provides a selective advantage at the population level is a fundamental question. In our opinion, the large over-representation of genes involved in the response to stress and survival in the variable fraction of the mussel pan-genome (Additional file 1: Data Note 18) and the impact of PAV on the molecular diversification of AMPs (Fig. 4b, Additional file 1: Data Note 21) may suggest an adaptive role for the pan-genomic architecture. This would be consistent with the benefits provided by the development of a complex arsenal of immune molecules in sessile species characterized by high population densities such as mussels, where the spread of pathogens can be very efficient [69, 70]. We can speculate that the accessory functions provided by the 20,000 *dispensable* mussel genes might underpin an improved ability to adapt to challenging and varying environmental conditions, resulting in the cosmopolitan distribution and high invasiveness potential of this species [2, 6, 7] and explaining a high standing genetic variation in mussel populations [7]. Since a large number of genes subject to PAV seem to belong to recently expanded, taxonomically restricted gene families with unknown function (Additional file 1: Data Note 19–20), the putative adaptive benefits of PAV might extend well beyond immunity and survival, with a potential impact on multiple aspects of mussel biology. At the present stage, in the absence of experimental data linking phenotypic variation and fitness to PAV in different ecological contexts, this remains a working hypothesis that needs to be formally tested.

Curiously, the genome of the congeneric non-invasive mussel *M. coruscus* [20], whose geographical distribution is limited to the Yellow Sea, displays a significantly lower number of protein-coding genes and a much lower level of heterozygosity compared with *M. galloprovincialis* (Additional file 1: Data Note 3 and 6), which suggests that the prevalence of PAV may vary from species to species. Future investigations, which will hopefully benefit from the release of additional chromosome-scale genome assemblies, should be aimed at investigating whether the pan-genomic architecture we described in the Mediterranean mussel is shared by other mollusks.

## Conclusions

We provide, for the first time, significant evidence in support of the existence of widespread gene PAV in a metazoan pan-genome. The unusual structure of the mussel genome is the result of the massive presence of hemizygous genomic regions, which contain several thousand *dispensable* protein-coding genes. The enrichment of these genes in functions associated to resilience to stress and immune response warrants further investigation on the possible links between massive PAV and the evolutionary success of mussels, exemplified by the cosmopolitan distribution of this species in temperate marine coastal waters. Most likely, extensive PAV might be found in other cosmopolitan marine invertebrates characterized by broadcast spawning, very large effective population size and subject to similar environmental pressures, including other bivalve species where similarly high heterozygosity rates have been reported.

## Methods

### Reference genome sequencing and assembly

The genomic DNA extracted from the mantle tissue of a single female mussel individual nicknamed *Lola*, collected at Ría de Vigo (Spain), was processed to generate different sequencing libraries for sequencing on an Illumina HiSeq2000 platform. A short-insert (800 bp) paired-end (PE) library, whose output accounted for an expected 110X genome coverage [18], was complemented with two long-insert mate-pair (MP) libraries, with a fragment size of 3 and 5 kb, respectively. Moreover, a fosmid library with 150,000 clones was used to generate 150 pools containing 1000 clones each, and two additional independent fosmid-end (FE) libraries were also constructed and sequenced. Overall, 330 Gb of raw sequence data were produced by Illumina sequencing, and 15.63 Gb additional raw sequence data (10.5X coverage) was obtained from the sequencing of a SMRT library on a PacBio Sequel platform.

We followed a hybrid multi-step de novo assembly strategy, which combined algorithmic strategies from the *de Bruijn* graph and Overlap-Layout-Consensus methods (Additional file 1: Fig. S2), with the aim to produce a highly contiguous non-redundant haploid reference assembly of the mussel genome which, based on preliminary *k-mer* count analyses [71], was expected to display a considerable proportion of duplicated sequence and high heterozygosity.

Briefly, the non-redundant unitigs, built with ABySS [72] and merged with ASM [73] to remove large artefactual duplicated haplotype blocks, served as anchors for the hybrid assembly of PacBio reads with DBG2OLC [74]. This noisy preliminary assembly was polished with Raccoon (https://github.com/lukud/raccoon-), using the sequencing data derived from the Illumina PE800 library. SSPACEv3.0 [75] was then used to perform a first round of scaffolding using all the available PE, MP, and FE libraries available, and a second round of scaffolding with PacBio reads was performed with SSPACE-LongRead [76]. The scaffolding procedure was re-iterated a second time, with both Illumina and PacBio reads, and was followed by a MP and FE libraries-derived gap-closing step performed with PBJelly [77].

The resulting assembly was subjected to an additional round of polishing with Proovread [78], and the coding portion of the genome was further refined with GATK [79], based on the alignment between the genome sequence and available transcriptome data generated with STAR [80]. RNA-seq data was also used for an additional round of scaffolding with AGOUTI v0.2.4 [81]. Finally, a local region of assembly, which included the myticin gene cluster, was improved by the combination of *Platanus* [82] and DBG2OLC [74].

All the aforementioned steps of the assembly were paired with strict decontamination procedures, which employed KRAKEN 2 [83] and BLASTN [84, 85]. These were aimed at removing exogenous DNA sequence which may have resulted from accidental environmental or laboratory contamination, a common issue in NGS approaches [86]. A final analysis of our final assembly (mg10) using *Blobtools v1.1.1* [87, 88] confirmed the absence of known contaminants in the genome sequence. Detailed information concerning the DNA extraction, library preparation, sequencing, genome assembly, and decontamination processes are provided in Additional file 1: Data Note 1.2.

### Quality evaluation, gene model construction, and functional annotation

The completeness of the genome assembly was estimated with BUSCO v.3 [89], using a set of 843 conserved metazoan single-copy orthologs as a reference, and the resulting data about the present, fragmented, duplicated, and missing gene models were compared with previous genome assembly efforts carried out in *M. galloprovincialis* [18, 19] (Additional file 1: Data Note 1.3.3). The residual presence of artefactual duplications was assessed with the Kmer Analysis Toolkit [90]. Consensus gene models were obtained by combining transcript alignments generated with PASA v 2.0.2 [91], bivalve protein alignments created with SPALN v2.2.2 [92], and ab initio gene predictions obtained with GeneID [93], GeneMark-ES [94], GlimmerHMM [94], and Augustus [95]. Evidences derived from these methods were assigned different weights and combined into consensus CDS predictions with EvidenceModeler-1.1.1. Gene models were subjected to an additional round of quality control to refine the annotation of UTRs and alternatively spliced exons (Additional file 1: Data Note 2.1 and 2.2). Gene models were functionally annotated with InterPro [96], KEGG [97], Blast2GO [98], SignalP [99], and NCBI CDsearch [100] (Additional file 1: Data Note 2.3). The gene models supported by PASA, but lacking a CDS, were considered as non-coding genes and included in a separate annotation track (Additional file 1: Data Note 2.5).

The completeness and integrity of gene models, as well as the genome assembly size and the number and density of gene models, were compared with several other recently sequenced molluscan genomes (Additional file 1: Data Note 3). Each gene model was assigned a support level (high, mild, or low) based on evidence obtained from *Lola* gills and digestive gland transcriptomes, as well as from several publicly available *M. galloprovincialis* RNA-seq datasets (Additional file 1: Data Note 4).

### Whole-genome resequencing of 14 additional individuals and pan-genome recursive assembly

The genome of 14 additional adult *M. galloprovincialis* specimens, belonging to two independent European populations (Ría de Vigo, Spain, 9 individuals, and Goro lagoon, Italy, 6 individuals, Additional file 1: Data Note 6.1), was resequenced on an Illumina HiSeq 2500 platform, aiming at achieving a 35X genome sequencing coverage. Raw sequencing data from the previous assembly of *Pura* was also included in this analysis [18]. In total, besides *Lola*, whole-genome resequencing (WGR) data of comparable quality was obtained for six female and eight male individuals. Trimmed sequencing reads were mapped on the mussel reference genome, and unmapped reads were collected and de novo assembled with the CLC Genomics Workbench v.20 (Qiagen, Hilden, Germany). Newly assembled contigs were added to the reference assembly, building a mussel pan-genome. This process, inspired by a similar approach previously carried out by other authors [32], was performed recursively for the 14 individuals (+ *Pura*), mapping the reads obtained from each genome against the growing pan-genome (Additional file 1: Fig. S66). All the de novo assembled contigs underwent a strict filtering process, aimed at removing exogenous contaminants, based on strict coverage and GC content criteria, and the detection of BLAST matches against known contaminants (Additional file 1: Fig. S67). Assembled contigs satisfying threshold quality criteria (detailed in Additional file 1: Data Note 14) were annotated following the same procedure described above for the reference genome.

### Presence-absence variation analysis

Quality-trimmed sequencing reads obtained from all individuals were independently mapped to the reference assembly and to the accessory pan-genomic contigs, with BWA mem (v0.7.15) [101]. As detailed in Additional file 1: Data Note 8, the mapping strategy we used aimed at tolerating multi-mappings (i.e., the alignment of reads with similar scores on different genomic positions). Exon mapping data, extracted with BEDtools [102], were used to calculate the average read coverage per base within the coding region of each gene. These values, normalized by the expected haploid genome size, allowed us to classify genes either as “present” or “absent,” depending on whether their normalized coverage exceeded 0.25 (i.e., less than 25% of expectations for a gene found in a hemizygous genomic region). This strict threshold was set to put a major focus on the high-confidence identification of putatively absent genes, at the cost of the detection of some false positives in the set of “present” genes. The same procedure was extended to non-coding genes annotated in the reference genome (Additional file 1: Data Note 9). The expected hemizygous and homozygous peaks of coverage for each genome were estimated with an accurate calibration pipeline, based on a set of over 4000 genes displaying high coverage stability across individuals, as explained in Additional file 1: Data Note 23.

The comparative analysis of gene PAV among individuals led to their categorization either as *core* (i.e., present on all individuals) or *dispensable* (i.e., absent in one or more individuals) genes. Please note that all the genes annotated in the accessory pan-genomic contigs were, by definition, *dispensable*, as they were not found in *Lola*, as verified by an accurate re-mapping of genomic reads obtained from both gills and mantle (Additional file 1: Data Note 14).

### Validation of PAV patterns and further characterization of *dispensable* genes

We explored whether the PAV patterns observed could be explained by technical artifacts linked with mapping stringency criteria or by high sequence diversity between allelic variants, computationally simulating the effect of decreasing mapping stringency on mapping rates, and of increasing diversity between allelic variants on the drop of observed sequencing coverage (Additional file 1: Data Note 10). We further confirmed the widespread nature of PAV in mussels though the analysis of the distribution of the genes encoded by the accessory pan-genomic contigs in the resequenced individuals (Additional file 1: Data Note 15) and identified further cases of PAV through the analysis of several publicly available *M. galloprovincialis* transcriptomes (Additional file 1: Data Note 13).

The PAV phenomenon was further confirmed by PCR on 13 mussel genomes, through the evaluation of the presence-absence of specific amplification bands on agarose for 12 selected *dispensable* gene targets, expected to produce discordant PCR results across individuals due to PAV, and 5 *core* genes. These experimental observations were compared with in silico predictions (see the details in Additional file 1: Data Note 12.1). Moreover, similar PCR analyses were extended to three different families of full-sib mussels, produced after induced spawning of a single male and a single female individual, to test whether the presence-absence of *dispensable* genes could be explained by Mendelian inheritance (see the details in Additional file 1: Data Note 12.2.).

We assessed to what extent *dispensable* genes were associated with hemizygous genomic regions by evaluating whether their coverage was consistent with the presence of zero, one, or two alleles in each individual (Additional file 1: Data Note 11). Particular attention was focused on the analysis of a few selected large genomic regions characterized by the presence of several contiguous dispensable genes (see Additional file 1: Data Note 17.1). We characterized the transcriptional activity of dispensable genes in different tissues through the mapping of several RNA-seq datasets (as detailed in Additional file 1: Data Note 16) and evaluated whether they were associated with significant codon usage bias, functional promoters, architectural alterations, and flanking transposable elements (Additional file 1: Data Note 17).

*Dispensable* genes were subjected to functional enrichment analyses with hypergeometric tests [103], which identified over- or under-represented associated Gene Ontology terms and conserved domain annotations, based on a FDR-corrected *p* value threshold of 0.05 (Additional file 1: Data Note 18). The phylome data (see below, and Additional file 1: Data Note 5) allowed us to investigate the association of *dispensable* genes with recent linage-specific gene cluster expansions, through the comparison between the rates of gene duplication with the background rate of the genome (Additional file 1: Data Note 19), and to evaluate their overlap with taxonomically restricted gene (TRG) families (Additional file 1: Data Note 20).

### Phylome reconstruction

The mussel phylome was reconstructed using the PhylomeDB pipeline [46], as detailed in Additional file 1: Data Note 5.1. This approach, which involved 16 target species, enabled the detection of orthology and paralogy relationships (Additional file 1: Data Note 5.2), lineage-specific gene duplications (Additional file 1: Data Note 5.4), and associated significantly enriched annotations, based on the genes annotated in *Lola* (Additional file 1: Data Note 5.5). Moreover, species trees were built using three different approaches: (i) a maximum likelihood analysis, carried out with PhyML v3.0 on a concatenated gene alignment dataset [42]; (ii) a gene-tree parsimony analysis, carried with the dup-tree algorithm [43]; and (iii) a coalescent-based analysis, performed with ASTRAL-III [44, 104] (Additional file 1: Data Note 5.3).

### Assessment of genetic introgression from congeneric species

Exploiting previously published data and experimentally validated haplotypes, we inspected whether *Lola*, *Pura*, and the resequenced mussel genomes displayed genetic signatures consistent with their identification as part of a “pure” *M. galloprovincialis* lineage, or any evidence of hybridization with congeneric species *M. edulis* and *M. trossulus* existed. For this purpose, we recovered in each individual the two alleles for three target nuclear loci Glu-5′ [105, 106], mac-1 [107–109], and EFbis [110, 111], and the sequence of the mitochondrial markers 16S rRNA and COI [60, 112, 113]. As detailed in Additional file 1: Data Note 7, amplicon size was predicted by in silico PCR, and the nucleotide sequences, aligned with MUSCLE [114] with sequences of known taxonomic origin retrieved from GenBank, were used to build neighbor joining (NJ) phylogenetic trees [115].

### Analysis of target PAV-associated gene families

We collected the nucleotide sequences of the *core* gene EEF1A1 and its *dispensable* paralogous gene EEF1A1-bis from *Lola*, *Pura,* and the 14 resequenced individuals. Similarly, all sequence variants available for the myticin, mytilin, big defensins, mytimycin, mytimacin, and myticalin gene families were recovered, with particular focus on the exons encoding the mature peptide region of these AMPs. We studied their association with the PAV phenomenon and investigated their molecular phylogeny and the ongoing process of pseudogenization of several variants with a NJ phylogenetic reconstruction approach (Additional file 1: Data Note 21).

## Supplementary information

**Additional file 1.** Manuscript data notes. This file includes additional data notes 1–24, along with all the supplementary figures and most supplementary tables referenced in the main text.

**Additional file 2.** Large supplementary tables. This file includes Table S1, Table S3, Table S4, Table S6, Table S16, Table S17, Table S18, Table S19, Table S20, Table S21, Table S22, Table S23, Table S24, Table S25, Table S26, Table S27, Table S28, Table S29, Table S30, Table S31, Table S32, Table S33, Table S34, Table S40, Table S52, Table S53, Table S54, Table S55, Table S56 and Table S57.

**Additional file 3.** Review history.

## Data Availability

All the genome sequencing data obtained in this work, as well as the genome assembly and the annotation, are available in the European Nucleotide Archive (ENA) under the project IDs PRJEB24883 (Lola) [116] and PRJNA230138 (resequencing) [117]. A genome browser and a blast server for this genome can be accessed in our local server (https://denovo.cnag.cat/mussel/). The mussel phylome is available for download or browsing at PhylomeDB [118].
