# Supplementary material for: Massive gene presence-absence variation shapes an open pan-genome in the Mediterranean mussel
Source: Genome Biol. 2020 Nov 10;21:275. doi: 10.1186/s13059-020-02180-3 (PMC7653742; doi:10.1186/s13059-020-02180-3)
Supplement: Supplementary file 1 — Additional file 1. Manuscript data notes. This file includes additional data notes 1–24, along with all the supplementary figures and most supplementary tables referenced in the main text. [file 13059_2020_2180_MOESM1_ESM.docx]

**Additional File 1**

**Massive gene presence-absence variation shapes an open pan-genome in the Mediterranean mussel** Marco Gerdol^1^, Rebeca Moreira^2^, Fernando Cruz^3^, Jessica Gómez-Garrido^3^, Anna Vlasova^4^, Umberto Rosani^5^, Paola Venier^5^, Miguel A. Naranjo-Ortiz^4,6^, Maria Murgarella^7^, Samuele Greco^1^, Pablo Balseiro^2,8^, André Corvelo^3,9^, Leonor Frias^6^, Marta Gut^3,6^, Toni Gabaldón^4,6,10^, Alberto Pallavicini^1,11^, Carlos Canchaya^7,12,13^, Beatriz Novoa^2^, Tyler S. Alioto^3,6^, David Posada^7,12,13*^, Antonio Figueras^2*^

1. Università degli Studi di Trieste, Department of Life Sciences, Via Licio Giorgieri 5, 34127 Trieste, Italy
2. Instituto de Investigaciones Marinas (IIM - CSIC), Eduardo Cabello, 6, 36208 Vigo, Spain.
3. CNAG-CRG, Centre for Genomic Regulation (CRG), Barcelona Institute of Science and Technology (BIST), Baldiri i Reixac 4, 08028 Barcelona, Spain
4. CRG - Centre for Genomic Regulation. Doctor Aiguader, 88. 08003 Barcelona, Spain
5. Università degli Studi di Padova, Department of Biology, Via Ugo Bassi 58/B, 35131 Padova, Italy
6. Universitat Pompeu Fabra (UPF). 08003 Barcelona, Spain
7. Department of Biochemistry, Genetics and Immunology, University of Vigo, 36310 Vigo, Spain
8. Norce Norwegian Research Centre AS, Bergen, Norway

1. New York Genome Center, New York, New York 10013, USA
2. ICREA, Pg. Lluís Companys 23, 08010 Barcelona, Spain
3. Anton Dohrn Zoological Station, 80121 Villa Comunale, Naples, Italy
4. Biomedical Research Center (CINBIO), University of Vigo, 36310 Vigo, Spain
5. Galicia Sur Health Research Institute, 36310 Vigo, Spain.

^*^Corresponding authors

**Table of contents**

[1. Data Note 1 – Sequencing and *de novo* assembly 7](#_Toc52551432)

[1.1. Sampling location 7](#_Toc52551433)

[1.2. Whole-genome sequencing and *de novo* assembly 7](#_Toc52551434)

[1.2.1. Genome assembly part 1: hybrid assembly 9](#_Toc52551435)

[1.2.2. Genome assembly part 2: polishing and scaffolding (mg8) 13](#_Toc52551436)

[1.2.3. Genome assembly part 3: scaffolding with RNA-seq data (mg9) 16](#_Toc52551437)

[1.2.4. Genome assembly part 4: refinement of the myticin gene cluster (mg10) 17](#_Toc52551438)

[1.2.5. Summary of the different versions of the assembly 18](#_Toc52551439)

[1.2.6. Search for contaminant traces in the final assembly 18](#_Toc52551440)

[1.3. Genome assembly evaluation 21](#_Toc52551441)

[1.3.1. Assessment of gene completeness and duplication 22](#_Toc52551442)

[1.3.2. The *k-mer* spectra supports a better collapse of heterozygous sequences 22](#_Toc52551443)

[1.3.3. Comparative overview with previous *de novo* assembly efforts 24](#_Toc52551444)

[2. Data Note 2 – Genome annotation 25](#_Toc52551445)

[2.1. Generation of consensus gene models 25](#_Toc52551446)

[2.1.1. Transcript alignment 25](#_Toc52551447)

[2.1.2. Protein alignment 25](#_Toc52551448)

[2.1.3. Repeat finding 25](#_Toc52551449)

[2.1.4. *Ab initio* gene predictions 26](#_Toc52551450)

[2.2. Generation of consensus CDS models 27](#_Toc52551451)

[2.3. Functional annotation of protein-coding genes 28](#_Toc52551452)

[2.4. Filtering of MGAL9C annotations 29](#_Toc52551453)

[2.5. Non-coding RNA annotation 30](#_Toc52551454)

[2.6. MGAL10B annotation 30](#_Toc52551455)

[3. Data Note 3 - Comparative assessment of genome completeness 32](#_Toc52551456)

[3.1. Genome completeness and gene model integrity evaluation 32](#_Toc52551457)

[3.2. Genome size 33](#_Toc52551458)

[3.3. Protein-coding gene number 35](#_Toc52551459)

[3.4. Gene density 35](#_Toc52551460)

[4. Data Note 4 – Support of gene models by RNA-seq data 37](#_Toc52551461)

[4.1. Transcriptomic datasets used 37](#_Toc52551462)

[4.2. Transcriptomic support calculation 38](#_Toc52551463)

[4.3. *Lola* transcriptome 40](#_Toc52551464)

[5. Data Note 5 – Phylome reconstruction 42](#_Toc52551465)

[5.1. Mussel phylome reconstruction 42](#_Toc52551466)

[5.2. Prediction of gene duplications, and orthology/paralogy relationships 44](#_Toc52551467)

[5.3. Species tree reconstruction 44](#_Toc52551468)

[5.4. Lineage-specific gene family expansion events 47](#_Toc52551469)

[6. Data Note 6 - Whole genome resequencing and heterozygosity rate estimates 50](#_Toc52551470)

[6.1. Resequencing of 14 additional mussels and re-analysis of *Pura* 50](#_Toc52551471)

[6.2. Initial estimate of heterozygosity rates by *k-mer* analysis 51](#_Toc52551472)

[6.3. Improved estimate of heterozygosity rates 56](#_Toc52551473)

[7. Data Note 7 – Assessment of genetic introgression from congeneric species 59](#_Toc52551474)

[7.1. Overview of molecular markers used for the assessment of genetic introgression and methodology used 59](#_Toc52551475)

[7.2. Polyphenolic adhesive protein gene Glu-5’ fragment polymorphisms 60](#_Toc52551476)

[7.3. EF1bis locus polymorphisms 61](#_Toc52551477)

[7.4. Mitochondrial DNA molecular markers 63](#_Toc52551478)

[8. Data Note 8 – Presence-Absence Variation (PAV) 67](#_Toc52551479)

[8.1. Read mapping 67](#_Toc52551480)

[8.2. Coverage normalization 68](#_Toc52551481)

[8.3. A large amount of the assembly displays a sequencing coverage consistent with an hemizygous state 68](#_Toc52551482)

[8.4. Detailed evaluation of gene coverage in *Lola* 69](#_Toc52551483)

[8.5. Detailed evaluation of gene coverage in the resequenced genomes 71](#_Toc52551484)

[9. Data Note 9 – Non-coding genes are also subject to Presence-Absence Variation 80](#_Toc52551485)

[9.1. Detailed evaluation of non-coding gene coverage in *Lola* and in the resequenced genomes 80](#_Toc52551486)

[10. Data Note 10 – PAV cannot be generally explained by divergence between allelic variants 89](#_Toc52551487)

[10.1. Effects of read mapping stringency on coverage estimates 89](#_Toc52551488)

[10.2. Effects of increasing allelic divergence on the coverage estimates 92](#_Toc52551489)

[11. Data Note 11 – In depth analysis of the sequencing coverage of *core* and *dispensable* genes 94](#_Toc52551490)

[11.1. Assessment of *Lola* genes encoded by hemizygous genomic regions 94](#_Toc52551491)

[11.2. Assessment of *Lola* genes encoded by homozygous genomic regions 95](#_Toc52551492)

[12. Data Note 12 – Validation of Presence-Absence Variation by PCR assays 97](#_Toc52551493)

[12.1. Primer design and experimental setup 97](#_Toc52551494)

[12.2. Results of the PCR validation assays 98](#_Toc52551495)

[12.3. PCR confirmation in familes of full-sib mussels 103](#_Toc52551496)

[13. Data Note 13 – *In silico* validation of PAV with RNA-seq data 104](#_Toc52551497)

[13.1. Experimental strategy 104](#_Toc52551498)

[13.2. Indentification of new PAV cases from RNA-sequencing data 108](#_Toc52551499)

[13.3. Characterization of new *dispensable* genes identified in the mussel transcriptome 109](#_Toc52551500)

[Data Note 14 – Construction of the mussel pan-genome 112](#_Toc52551501)

[14.1. Recursive pan-genome reassembly and decontamination strategy 112](#_Toc52551502)

[14.2. Initial pan-genome assembly results 118](#_Toc52551503)

[14.3. Decontaminated pan-genome assembly results 119](#_Toc52551504)

[14.4. Decontamination detailed report 124](#_Toc52551505)

[14.5. ITAF3: an exemple of efficient decontamination from the exogenous DNA of a mantle parasitic hydrozoan 138](#_Toc52551506)

[15. Data Note 15 – Evaluation of Presence-Absence Variation on the *dispensable* genes from the pan-genome 143](#_Toc52551507)

[15.1. Pan-genomic *dispensable* genes annotation 143](#_Toc52551508)

[15.2. Presence-Absence Variation analysis 145](#_Toc52551509)

[15.3. Functional enrichment analysis 154](#_Toc52551510)

[16. Data Note 16 –Expression levels of *core* and *dispensable* genes 159](#_Toc52551511)

[16.1. Experimental setup 159](#_Toc52551512)

[16.2. Overview of gene expression levels of *core* and *dispensable* genes 159](#_Toc52551513)

[16.3. Gene expression levels of *core* and *dispensable* genes in *Lola* 161](#_Toc52551514)

[16.4. Contribution of *dispensable* genes to transcriptional activity 163](#_Toc52551515)

[17. Data Note 17 – Structural features of PAV 165](#_Toc52551516)

[17.1. Genomic organization of *dispensable* gene clusters 165](#_Toc52551517)

[17.2. An example: mytilin K 168](#_Toc52551518)

[17.3. Coding sequence features of *core* and *dispensable* genes 170](#_Toc52551519)

[17.4. Assessment of the presence of transposable elements in the genomic regions neighboring PAV genes 175](#_Toc52551520)

[18. Data Note 18 – Functional enrichment of *dispensable* genes 176](#_Toc52551521)

[18.1. Detection of significantly over-represented annotations 176](#_Toc52551522)

[18.2. Over-represented domains 176](#_Toc52551523)

[18.3. Under-represented domains 178](#_Toc52551524)

[18.4. Gene Ontology – Cellular component annotations 178](#_Toc52551525)

[18.5. Gene Ontology – Biological process annotations 179](#_Toc52551526)

[18.6. Gene Ontology – Molecular function annotations 179](#_Toc52551527)

[19. Data Note 19 – Evolutionary considerations on *dispensable* genes 180](#_Toc52551528)

[19.1. Relationship between *dispensable* genes and gene duplication events 180](#_Toc52551529)

[19.2. Association between PAV and expanded gene clusters 184](#_Toc52551530)

[20. Data Note 20 – Taxonomically restricted genes and their relationship with PAV 189](#_Toc52551531)

[20.1. Bayesian reconstruction of Mytilida phylogeny 189](#_Toc52551532)

[20.2. Examples of taxonomically-restricted gene families 192](#_Toc52551533)

[20.3. Correlation between TRGs and PAV 192](#_Toc52551534)

[21. Data Note 21 – Key examples of PAV 193](#_Toc52551535)

[21.1. Elongation factor 1 alpha 193](#_Toc52551536)

[21.2. Mytilins 197](#_Toc52551537)

[21.3. Mytimacins 202](#_Toc52551538)

[21.4. Myticins 203](#_Toc52551539)

[21.5. Mytimycins 208](#_Toc52551540)

[21.6. Big defensins 215](#_Toc52551541)

[21.7. Myticalins 218](#_Toc52551542)

[22. Data Note 22 – Size and composition of the mussel pan-genome 221](#_Toc52551543)

[22.1. Estimates of the size of the mussel pan-genome 221](#_Toc52551544)

[22.2. Comparison with other species 222](#_Toc52551545)

[22.3. Correlation with the geographical origin 226](#_Toc52551546)

[22.4. Assessment of the possible origin of *dispensable* genes from congeneric mussel species 227](#_Toc52551547)

[23. Data Note 23 – Discussion of anomalous read mapping in male mussel gonads 230](#_Toc52551548)

[23.1. Observation of aberrant mapping profiles in male libraries obtained from the mantle tissue 230](#_Toc52551549)

[23.2. Peak coverage calibration in male mussels 233](#_Toc52551550)

[24. Data Note 24 – Recommendations for gene expression studies 239](#_Toc52551551)

[24.1. How can *dispensable* genes be efficiently managed in gene expression studies? 239](#_Toc52551552)

[24.2. On the possible collapse of the product of paralogous gene copies 241](#_Toc52551553)

[References 244](#_Toc52551554)

# 1. Data Note 1 – Sequencing and *de novo* assembly

- 1. **Sampling location**

A single *Mytilus galloprovincialis* (Mediterranean mussel) female specimen (hereby named *Lola*) was collected at Ría de Vigo (42°15'54.8"N 8°43'42.5"W, Vigo, Galicia, Spain). The sampling location is displayed in **Fig. S1**. For details, see **Additional file 2:** **Table S1**).


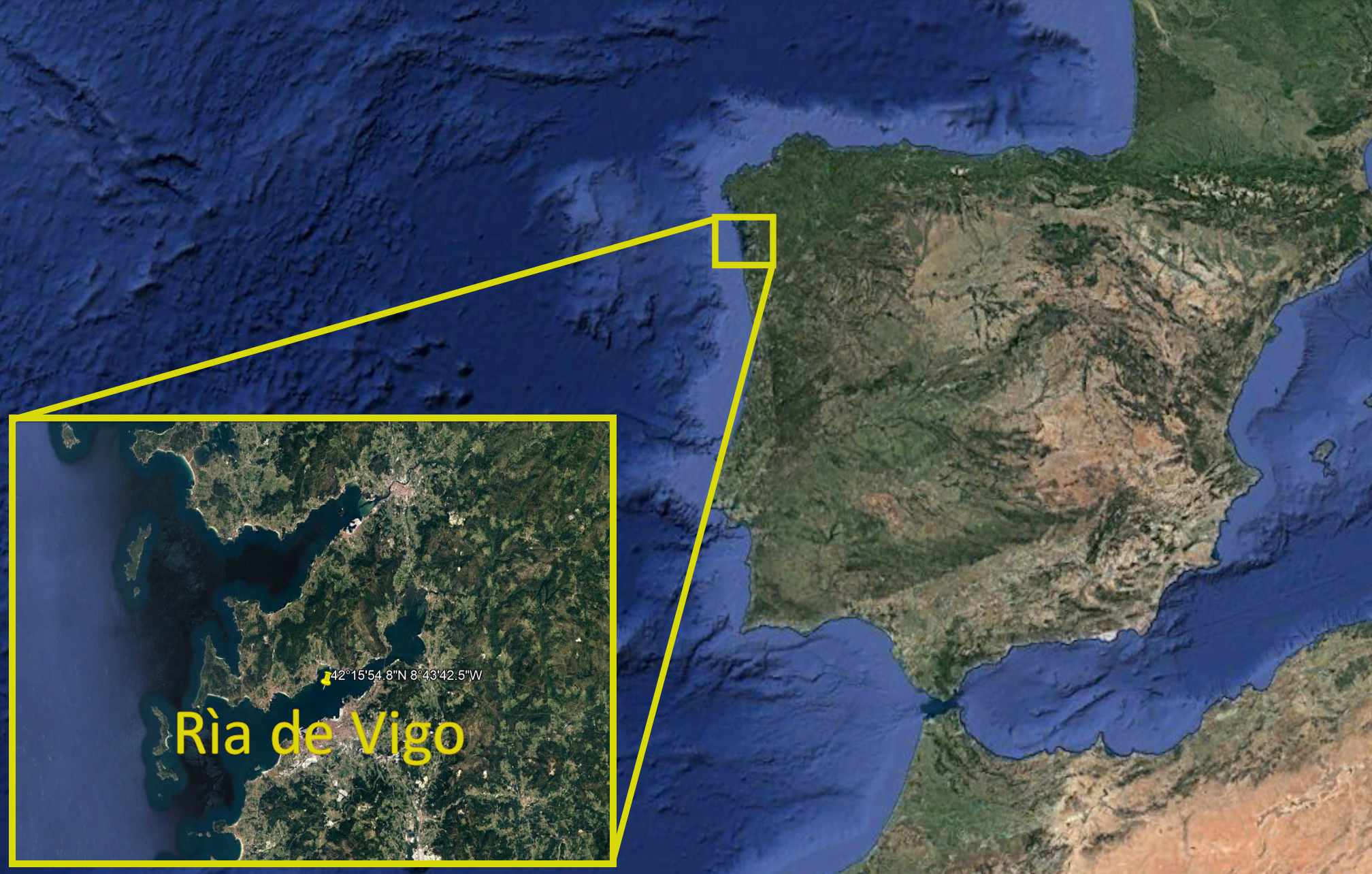


**Fig. S1**. **Sampling location of Lola, the *M. galloprovincialis* female specimen sequenced in the present study**. Geographical coordinates of the sampling site are: 42°15'54.8"N 8°43'42.5"W, Ría de Vigo, Galicia, Spain.

## Whole-genome sequencing and *de novo* assembly

Genomic DNA was extracted from the mantle tissue of *Lola* using the CTAB method and further cleaned with the QIAGEN Genomic-tip protocol (Hilden, Germany). The DNA quantity, purity and integrity were verified by electrophoresis and spectrophotometry in a 1% agarose gel and in a ND1000 (NanoDrop Technologies, Inc., DE, USA) respectively. Then, aliquots were prepared for several different library construction protocols (**Table S2**)

First, one paired-end (PE) library of 800 bp fragment size (004G_C) was prepared and sequenced on an Illumina HiSeq2000 platform. The standard Illumina protocol with minor modifications was followed for the creation of short-insert PE libraries (Illumina Inc., Cat. # PE-930-1001). In brief, 2.0 μg of genomic DNA was sheared on a Covaris™ E220, the fragmented DNA was end-repaired, adenylated and ligated to Illumina specific PE adaptors. To obtain this PE library with approximate fragment sizes of 800 bp, the DNA with adaptor-modified ends was size selected and purified using the E-gel agarose electrophoresis system (Invitrogen). The PE library was run on the HiSeq2000 (2x101 bp) according to standard Illumina operation procedures. The amount of sequence obtained for each library is summarized in **Table S2.** A total of 161.43 Gb of raw sequence (109x coverage, based on the estimated genome size of 1.48 Gb) were produced. Primary data analysis was carried out with the standard Illumina pipeline (HCS 2.0.12.0, RTA 1.17.21.3).

Second, two Mate pair (MP) libraries (3 and 5 kb fragment sizes) were constructed according to the Nextera MP preparation protocol, which leaves a linker of known sequence at the junction. Both libraries were sequenced on an Illumina HiSeq2000 platform with a 2x101 bp strategy, producing 52.38 Gb of raw sequence for the 3 kb library and 55.19 Gb of raw sequence for the 5 kb library.

Additionally, a fosmid library of 150,000 clones was constructed by Lucigen Corp (Middleton, USA), starting from a new extraction of genomic DNA, carried out from a different portion of mantle tissue from *Lola*. One-hundred fifty pools of approximately 1,000 clones per pool were made, and the purified DNA was used to prepare paired-end reads for sequencing on an Illumina HiSeq2000 platform (2x101 sequencing cycles). In addition, two independent fosmid-end (FE) libraries were constructed by Lucigen and sequenced in three lanes of a HiSeq2000 (2x101) instrument, producing 34.55 Gb of sequence, albeit with a 39.23% duplication rate due to the low complexity of the library.

Finally, genomic DNA from *Lola* was sent to Johns Hopkins University Deep Sequencing and Microarray Core and was sequenced with the SMRT PacBio technology producing 1,802,992 filtered subreads, adding up another 15.63 Gb of sequence, approximately accounting for a 10.56x genome sequence coverage. In terms of read length distribution, 50% of the reads obtained were longer than 11.11 Kb and 90% were longer than 5.44 Kb.

**Table S2.** **Output of Sequencing Libraries.**

| Library type | Read length | Fragment length | Yield (Gb) | phix error r1 (%) | phix error r2 (%) |
| --- | --- | --- | --- | --- | --- |
| 500 bp PE | 101 | 500 bp | 161.43 | 0.29 | 0.46 |
| 5 kb MP | 101 | 5 kb | 55.19 | 0.28 | 0.55 |
| 3 kb MP | 101 | 3 kb | 52.38 | 0.26 | 0.52 |
| Fosmid Ends | 101 | 40 kb | 29.95 | 0.61 | 0.7 |
| Fosmid Pools^1^ | 101 | 300 bp | 4.23 | 0.32 | 0.43 |
| PacBio^2^ | 8.668 | - | 15.63 | 14.89 | - |

^1^average values for the 150 fosmid pools

^2^Information corresponding to the pacbio subfiltered reads used in the assembly. Table reports average read length, yield and error rate. The N50 is 11,152 and N90 5,445 bp. The error rate has been estimated considering that subread accuracy is 85.11%.

### Genome assembly part 1: hybrid assembly

The assembly strategy followed the pipeline outlined in **Fig. S2** and explained in detail in the following paragraphs.


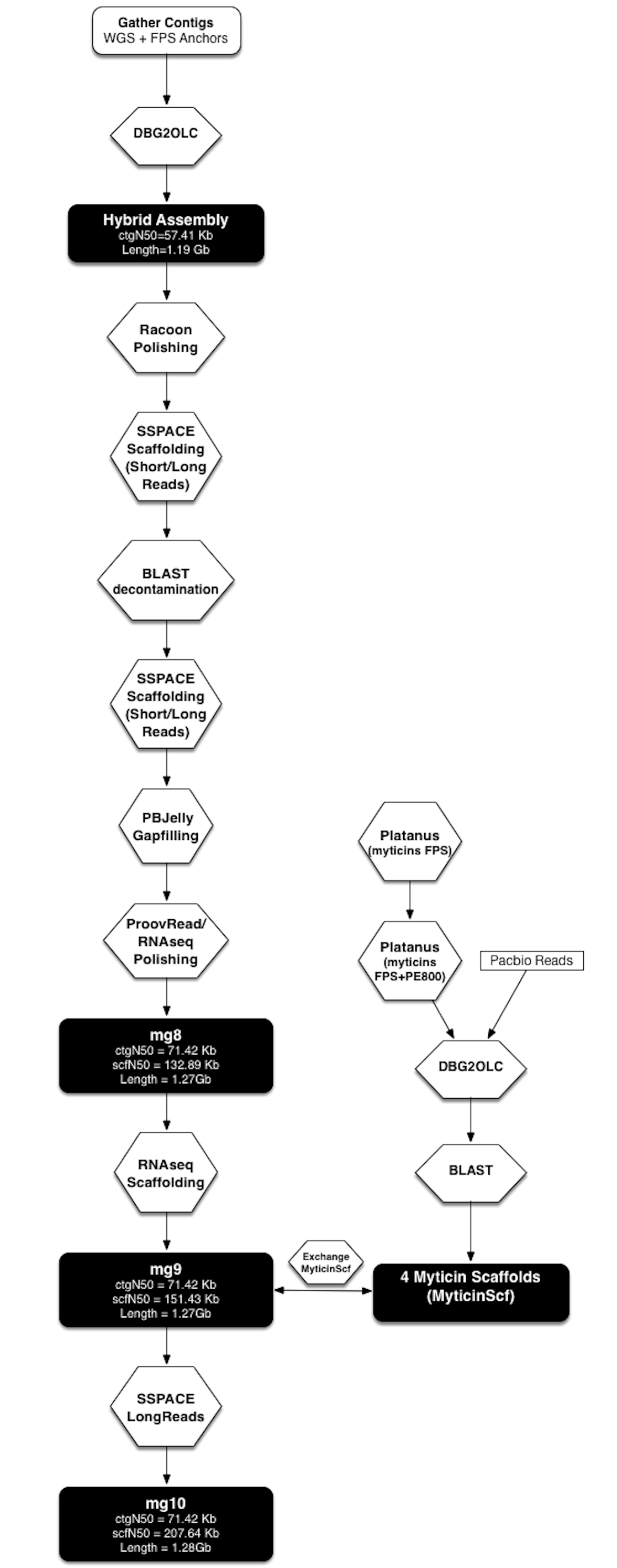


**Fig. S2: schematic overview of the hybrid genome assembly strategy applied in this study.**

**Preprocessing of sequence reads**

Post-processing of sequence reads involved detection and trimming of Illumina adapter sequences and quality trimming using the Trim Galore! wrapper script (<http://www.bioinformatics.babraham.ac.uk/projects/trim_galore/>) which employs the tool *cutadapt* [1]. The linker sequence present in the mate pair sequences was also removed with *cutadapt*. Overlapping reads derived from shorter fragments were merged using FLASH [2]. Then, all reads were filtered by mapping (gem-mapper [3], allowing up to 2% mismatches) against a contamination database that included phiX, Univec sequences and *E. coli*.

**Initial *k-mer* analyses**

An analysis of *k-mers* present in the sequence reads of the PE800 library was carried out using Jellyfish [4] to count *k-mers* of length 17. A peak in the distribution of 17-mer was observed at 75-fold coverage (**Fig. S3**). A rough estimate of genome size can be made by dividing the total number of counted *k-mers* (111,036,024,158) by the unique *k-mer* coverage (75), which results in a 1.48 Gb genome size estimate. An evident shoulder at depth 150 indicates the existence of a considerable proportion of duplicated sequences in the genome.

Accounting for sequencing error, bias, and repetitive sequence using the program *gce* [5], we obtained a more accurate estimate of 1.41 Gb. These 17-mer analyses depicted a complex, repetitive and highly heterozygous genome, in fact the *k-mer*-individual heterozygous ratio was about 0.0750 and for the *k-mer* species this value reached 0.0298.

**Fig. S3. 17-mer analysis of the sequenced reference genome**. All 17-mers in the PE800 library were counted and the number of distinct 17-mers (*k-mer* species) for each depth from 1 to 200 are shown in this plot. Two principal peaks are detected: the peak on the right (depth 75) corresponds to unique homozygous sequences. The high peak on the left (depth 37) corresponds to heterozygous sequences. The periodic, barely visible waves to the right correspond to multi-copy repetitive sequences in the genome. The high peak at very low depth is caused by sequencing errors.

**Building non-redundant contigs**

Initial attempts following a similar strategy to the one used for the olive (*Olea europaea*) genome [6] produced an assembly with inflated total genome length and evidences of artefactual duplications (large gene clusters of low-heterozygosity likely to be un-collapsed alleles or homologous haplotype stretches). To avoid this bias, we decided to use the unitigs from the fosmid pools to build the “anchors” for the hybrid assembly (**Fig. S2**). These unitigs are highly contiguous sequences from a haploid segment of the genome and the only source of duplication would be due to the presence of two different haplotypes from the same genomic region in two or more clones. The concatenated unitigs of the 150 fosmid pools represented 5.15 Gb of sequence with N50=3499 bp and N90=189 bp (see **Fig. S2**). We used ASM (Frias L, Ribeca P: ASM scripts are publicly available at <https://github.com/lfrias81/anchor-asm/tree/master/wrapper>) to merge the overlapping unitigs and obtain contigs or consensus sequences from the same region. One round of merging was applied using a range of anchors between 250 and 62 bp with a spacing of 50 bp. Divergence was set to 8% with a maximum-edit distance of the 10%, disabling repeat resolution and choosing the longest contig as consensus. The merging of different haplotypes produced a contig assembly (FPS) comprising 1.06 Gb of sequence and N50 of 1.7 Kb.

In order to include genomic sequences absent from the fosmid pool data we mapped the PE library PE800 with the gem-mapper [3] (allowing 4% of mismatches and 8% edit distance) to the initial 5.15 Gb constituted fosmid pools unitigs. This relaxed mapping allowed the detection of reads that matched either to one or another allele from the same locus or genomic region, before merging. Afterwards, all the unmapped whole-genome shotgun reads were assembled with ABySS [7] with k=87 to produce unitigs. This complementary assembly was also merged using ASM to collapse haplotype blocks coming from the same locus. The resulting whole-genome sequence (WGS) assembly had a contig N50 of 995 bp and comprised 90.35 Mb that were not contained in the fosmid-pools.

Finally, both sets of contigs, the FPS and WGS, were gathered into a single assembly that was 1.15 Gb long and had an N50=1.61 Kb (see **Additional file 2:** **Table S3**).

**Hybrid assembly**

Hybrid assembly methods integrate short and long reads to obtain both accurate and more contiguous reference genome assemblies. Here we used DBG2OLC [8], an assembler that combines algorithmic strategies from the de Bruijn graph and Overlap-Layout-Consensus methods. First, it is very important to construct accurate contigs using a de Bruijn graph. These contigs will serve as anchors to align to the long reads. As stated before, we built contigs from non-redundant unitigs (built with ABySS [7] and merged with ASM (<https://github.com/lfrias81/anchor-asm/tree/master/wrapper>)) to serve as accurate anchors for DBG2OLC. These anchors were aligned to the 10.6 x PacBio Reads to obtain an assembly using the following parameters: k=17 KmerCovTh=2 MinOverlap=20 AdaptiveTh=0.002 and RemoveChimera option on. The final hybrid assembly comprised 1.19 Gb of sequence free of gaps and contig N50=57.41 Kb (**Additional file 2:** **Table S3**). In the other hand, the gene completeness estimated with CEGMA v. 2.5 [9] was low, with 60.08% Complete and 83.06% Partial genes found (**Additional file 2:** **Table S4**).

The different versions of the genome assembly will be hereafter named “mgX”, where X is the number of the assembly version.

### Genome assembly part 2: polishing and scaffolding (mg8)

**Polishing the assembly with PE reads**

The low gene completeness observed in the hybrid assembly was mainly due to low sequence quality coming from the noisy PacBio reads. Therefore, we further tried to improve the nucleotide sequence of these long contigs. Given the evenness of coverage of the PE800 library across the genome, we decided to use this library for the nucleotide-level polishing. For this purpose, we used the pipeline Raccoon (<https://github.com/lukud/raccoon->). After several tests, we decided to use only one iteration of polishing as further rounds tended to introduce new indels, often breaking the open reading frames. After the Raccoon polishing, the assembly showed a 7% increase in CEGMA statistics, with 68.15% Complete and 87.10% Partial genes found (**Additional file 2:** **Table S4**).

**Scaffolding with all Illumina libraries**

Another effect of polishing is that the increased accuracy of the contig sequence will improve the paired mapping and connection of the contigs with the whole-genome shotgun libraries. For such purpose we used SSPACEv3.0 [10]. For this first round of scaffolding we decided to avoid spurious connections among contigs with low sequence quality. Therefore, we used GEM with parameters m=0.025 and e=0.05 and applied some filters to detect *unique mappings with no subdominant match* to accommodate for allelic variation*,* see the example below:

zcat reads/lib800_2x101_C236NACXX_8_0.interleaved.01.clean.interleaved.fastq.gz | gem-mapper -I unsspaced.gem --mismatch-alphabet ACTGN -q offset-33 -m 0.025 -e 0.05 -T3 -s 1 --fast-mapping=0 2> gem_logs/gem_map.lib800_2x101_C236NACXX_8_0.interleaved.01.clean.interleaved.err | gt.filter -t 1 --reduce-to-unique-strata 1 | gt.filter -t 1 --reduce-to-max-maps 1 | scripts/gem2tab_interleaved.pl - | gawk '$1 != $4' > fastq_tabs/gem_map.lib800_2x101_C236NACXX_8_0.interleaved.01.clean.interleaved.ta

The data employed came from all the sequenced libraries (PE800 2x100, MP3k, MP5k and the FE library). Scaffolding was carried out with parameters –k 10 -a 0.6 using SSPACEv3.0 [10]. This assembly showed a scaffold N50=75.46 Kb and a total length of 1.20 Gb, just 21 Mb below the expected genome length (1.41G).

**Scaffolding with PacBio reads**

The new scaffolds obtained with SSPACEv3.0, could be further connected using complementary long read information. Thus, we used SSPACE LongRead [11] to scaffold the previous assembly using the filtered PacBio subreads. As a result, the scaffold contiguity was almost doubled, reaching an N50 of 148.23 Kb.

**Rigorous decontamination of the assembly**

Although our initial decontamination was stringent enough to remove known contaminants (almost exact matches with <2% mismatches) such as PhiX, it was less efficient in detecting possible contaminants sequences displaying a lower degree of sequence similarity with those included in our contaminant database. Contaminant sequences can be embedded in the assembly, preventing the scaffolding of the real genomic sequence. Additionally, as mussels are filter-feeding organisms, the sequenced sample was susceptible of containing bacterial contaminants originally present in the water column. For these reasons, we constructed a more comprehensive database of contaminants. First, we detected the presence of contaminants in the reads using KRAKEN [12]. This program detected sequences corresponding to *Alteromonas macleodii* (0.06%)*,* *Mannheimia haemolytica* (0.01%) and Human herpesvirus 7 (0.01%) in the Illumina reads. Despite the low level of contaminating sequences, we added these contaminants to our contaminant database and BLASTed them against our assembly. Upon the detection with BLASTN [13,14] and removal of contaminated sequences, the assembly itself was broken into contigs and re-scaffolded (**Fig. S2** and **Additional file 2:** **Table S3**).

**Re-Scaffolding with all Illumina libraries**

The decontaminated contigs were re-scaffolded with SSPACEv3.0 [10] using all the Illumina sequencing libraries (PE800 2x100, MP3k, MP5k and the fosmid-end library) based on equally restrictive mappings as before. However, in this case we required a minimum number of links to create a scaffold (parameter k) of 15 and –a=0.6.

**Re-Scaffolding with PacBio reads**

The assembly resulting from the previous step was then re-scaffolded with SSPACELongRead [11] using the Pacbio reads and default parameters. The output assembly showed a contig N50 equal to 57.5 Kb, and a scaffold N50 equal to 142.48 Kb. It contained a total of 38.57 Mb in gaps, of which 239 were greater than 9 Kb.

**Filling large gaps with PacBio reads**

The assembly held large gaps between contigs connected using mate-pair (MP) and FE libraries. In particular, the latter were 20-40 Kb long. For this reason we used the program PBJelly [15], distributed in PBSuite_v15.8.24, to close the longest gaps in the assembly. As result of this step, the contig N50 increased to 68.5 Kb, scaffold N50 147.43 Kb. This assembly is referred as mg5. Noticeably, the total sequence in gaps dropped to 20.76 Mb and just 137 of them were greater than 9 Kb. This means that PBJelly closed 57.32% of the larger gaps in the assembly.

**Polishing with Proovread**

Despite of the considerable reduction of gaps and the subsequent increase in contig N50, the gene completeness estimated by CEGMA was 64.52% Complete and 83.06% Partial. In fact, these figures are lower than after our first polishing round with the Raccoon pipeline. In order to polish the assembly, we tried an additional round of Raccoon but the CEGMA statistics were even worse. As an alternative approach, we used all the unitigs from the fosmid pool assemblies that were longer than 1 Kb and polished the mg5 assembly using proovread [16]. The corrected assembly (mg7) gained sequence quality and the CEGMA statistics increased to 69.76% Complete and 86.29% Partial.

**Polishing the assembly with RNA-seq data**

We tried to polish the coding part of the genome in the assembly using RNA-seq information. As most of the sequence in genes is conserved by purifying selection we decided to use RNA-seq data from a different individual used in another study [17] and from data available at NCBI to include the “reference” representation of the exons in this genome. More precisely, we used RNA-seq data from gill, mantle, hemocytes and muscle [18].

We followed the guidelines of GATK [19] for Calling Variants in RNA-seq (<https://software.broadinstitute.org/gatk/documentation/article.php?id=3891>). All the reads were mapped with STAR [20] using the *2-pass* method, a first pass to identify the junctions and a second to align the RNA-seq data accounting for these junctions. Once the reads were mapped, we applied the *split and trim* step, which splits reads into exon segments and hard-clip any sequences overhanging into the intronic regions. After this, base qualities were reassigned and we performed realignments around indels. Finally, we called the variants using GATK’s *HaplotypeCaller* and filtered them with the recommended RNA-seq settings. The exact settings used for variant calling and variant filtration (VCF) are shown below:

java -Xmx4g -Djava.io.tmpdir=$TMPDIR -jar /apps/GATK/3.6/GenomeAnalysisTK.jar -T HaplotypeCaller -R mg7.fa -I all_rnaseq_vs_mg7.bam **-dontUseSoftClippedBases -stand_call_conf 20.0 -stand_emit_conf 20.0** -o all_rnaseq_vs_mg7

java -Xmx4g -Djava.io.tmpdir=$TMPDIR -jar /apps/GATK/3.6/GenomeAnalysisTK.jar -T VariantFiltration -R mg7.fa -V all_rnaseq_vs_mg7.**vcf -window 35 -cluster 3 -filterName FS -filter “FS > 30.0” -filterName QD -filter “QD < 2.0”** -o all_rnaseq_vs_mg7.filtered.vcf

In addition, the resulting VCF (with SNVs and indels) was also filtered for sites that contained at least 10 reads in one of the samples (i.e., tissues). We assigned a consensus genotype to each variable position, weighting towards the most frequent allele. First, we accounted for the sequencing depth of the alternative allele respect to the total depth across all samples. Second, whenever the alternate allele was in more than 70% of the reads, we assigned the genotype of that position as homozygous alternate (1/1). In the end, the final VCF containing all sites classified as homozygous alternate was the input to produce a new reference assembly with the GATK’s *FastaAlternateReferenceMaker* program*.*

This correction produced an assembly named mg8, with a mild gain in CEGMA, showing 70.16% Complete and 86.29% Partial gene models.

### Genome assembly part 3: scaffolding with RNA-seq data (mg9)

The software AGOUTI v0.2.4 [21] was run in order to improve the assembly and annotation (see below) with transcriptome data. After running Agouti, some scaffolds were joined and we ended up with the final mg9 assembly of 13,748 scaffolds and 1.27 Gb total length. The contig N50 of this assembly was 71.42 kb and the scaffold N50, 151.43 Kb. Ninety percent of the assembly was contained in 8,772 scaffolds of 43.4 kb or larger. Moreover, gene completeness was determined using CEGMA, which found 70.16% complete genes (86.29% at least partially present) of out 248 *core* eukaryotic genes. In addition, BUSCO estimated 69% complete genes and 8.5% fragmented genes. Among the complete genes, only a 13% of them seems to be duplicated. Statistics for all major stages of the assembly process are given in **Additional file 2:** **Table S3** and **S4**.

To obtain the protein-coding gene annotation for the mg9 assembly, we transferred the MGAL8A gene models (see **Data Note 2**) to their new coordinates in the mg9 assembly. Only 370 genes changed in this new version, as they were collapsed with other genes by Agouti. The protein-coding gene annotation derived from this step, MGAL9C, had 67,468 genes and 89,974 transcripts that encoded 85,682 protein products.

### Genome assembly part 4: refinement of the myticin gene cluster (mg10)

After, examining the scaffolds containing the myticin genes in the *mg*9 assembly we identified several issues, for example, two scaffolds containing long inverted repeats and a 10 Kb gene with an unusually long intron. Thus, in order to improve the assembly of the genomic regions containing these important immune genes, we completed the following steps:

1. We detected the fosmi-pools containing myticins by aligning myticin complete cds from GenBank (EU088427.1 and JF990710.1) against the fosmid pools’ unitigs with BLAST. This procedure enabled the identification of significant matches in 12 fosmid pools.
2. The raw illumina reads from sequencing these 12 fosmid pools were preprocessed and filtered for contaminant sequences previously detected using KRAKEN.
3. A recent study suggested that *Platanus* [22] works well in combination with DBG2OLC [8]. Therefore, we assembled the preprocessed reads using *Platanus* (version 1.2.4) with coverage parameter -e 88 and identity for bubble crush -u 0.4, in order to account for the huge heterozygosity in the mussel (see **Data Note 6**).
4. The whole-genome PE800 library was used to detect exact unique mappings to the initial contigs built with *Platanus*.
5. A second *Platanus* assembly was obtained adding the whole-genome data mapped.
6. All the Platanus contigs were used as anchors for the PacBio reads to obtain a hybrid assembly using DBG2OLC (*myticin_dbg2olc* assembly)
7. The scaffolds containing the myticins in the *myticin_dbg2olc* assembly were detected using BLAST.
8. The new scaffolds containing myticins were replaced for their counterpart in *mg*9.
9. A round of scaffolding with long reads was performed on the new assembly. Although, there were no new connections among the myticin scaffolds, the overall contiguity increased to scaffold N50 207.64 Kb. The new assembly was named *mg10.*

### Summary of the different versions of the assembly

To simplify the interpretation of the different steps of the assembly, we here summarize the characteristucs of the main assembly versions referenced in this document:

- mg3: a complete, but highly redundant, hybridgenome assembly, obtained as described in **section 1.2.1**, using a combination of Illumina PE and FE, and PacBio seqeucning data. This assembly version was produced with DBG2OLC, followed by multiple rounds of polishing, scaffolding using both short and long reads (with SSPACE and SSPACE-LongRead), and de-contamination.
- mg4: an improved and less redundant version of mg3, obtained through the merging of overlapping unitigs, using ASM.
- mg5: an improved version of mg4, obtained with a gap-filling process, using PBJelly.
- mg7: an improved version of mg5, obtained by polishing all the unitgs with length > 1Kb from the fosmid pool with Illumina reads, using proovread.
- mg8: an improved version of mg7, obtained with a round of polishing using RNA-seq data, with GATK.
- mg9: an improved version of mg8, obtained with a round of scaffolding using RNA-seq data, using AGOUTI.
- mg10: the final reference genome assembly, obtained through the refinement of the myticin scaffold, using Platanus and DBG2OLC.

### Search for contaminant traces in the final assembly

As a final quality control, we also ran *Blobtools v1.1.1* [23,24] (<https://github.com/DRL/blobtools>) on our final assembly version: mg10. The method requires three different inputs a target fasta file to be inspected, the reads aligned to the genome and the megablast best hits of the genome. First, our target was the mg10 assembly. Second, we used the BWAmem alignments of the reads from the PE800 library (obtained from the *Lola* mantle sample) against the mg10 reference assembly due their even coverage distribution across the genome. Third, we used BLASTv2.5.0 to search similarities of mg10 scaffolds against the non-redundant nucleotides database (*nt*) with *megablast*. As shown below, this was done using the recommended parameters:

blastn

-num_threads 24 \

-max_target_seqs 25 \

-culling_limit 2 \

-evalue 1e-25 \

-outfmt "6 qseqid staxids bitscore std sscinames sskingdoms stitle" \

-task megablast \

-query mg10.scaffolds.fa \

-db nt

By using *Blobtools v1.1,* we generated the BlobDB database using the three files above, and produced tables and plots for the following taxonomic ranks: Phylum, Family, Genus and Species. Consistently with the mapping rate of the PE800 library (93.09%), Blobtools classified 91.83% of the reads as mapped and 8.17% as unmapped. The ReadCovPlot (Fig. S4) shows that 91.37% of the reads (99.53% of the mapped ones) belong to the Phylum Mollusca with less than 0.5% of them suspicious of contamination. In fact, 0.4% of these reads are sub-threshold matches classified as ‘no-hit’.


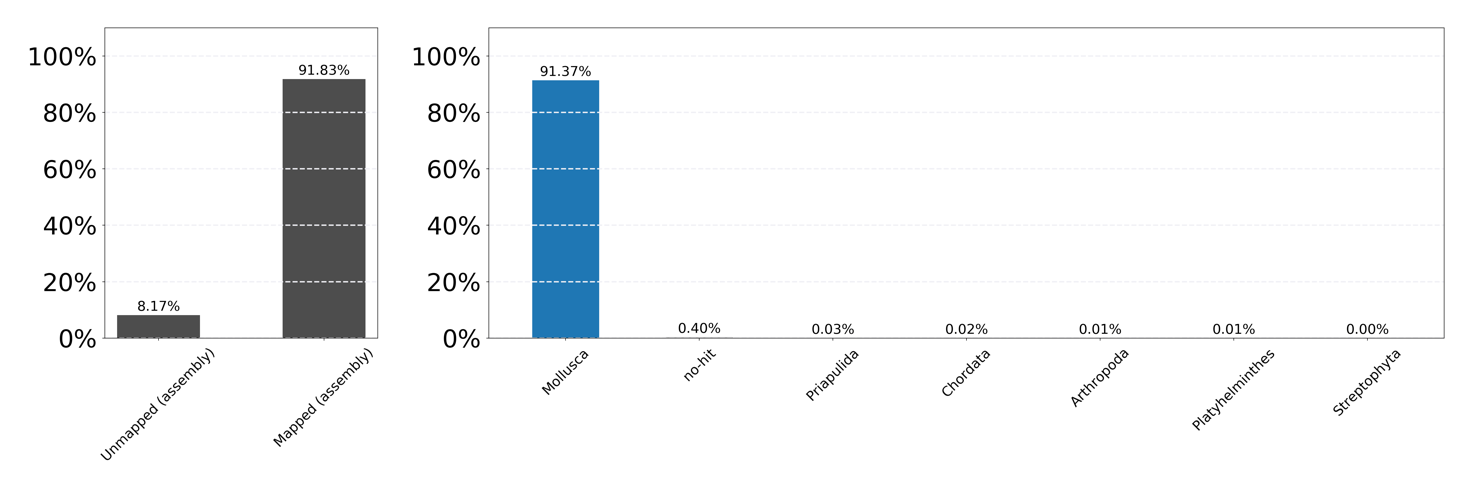


**Fig. S4. ReadCovPlot at the phylum level**. The graph reports the results obtained for the reference genome assembly mg10.

This result suggests negligible contamination in mg10. In addition, a closer glance to the %GC indicates that most of the scaffolds distribute around 32.18% and the non-mollusca cumulative sequence (span) of about 7Mb, representing 0.5% of mg10 (see **Fig. S5**):


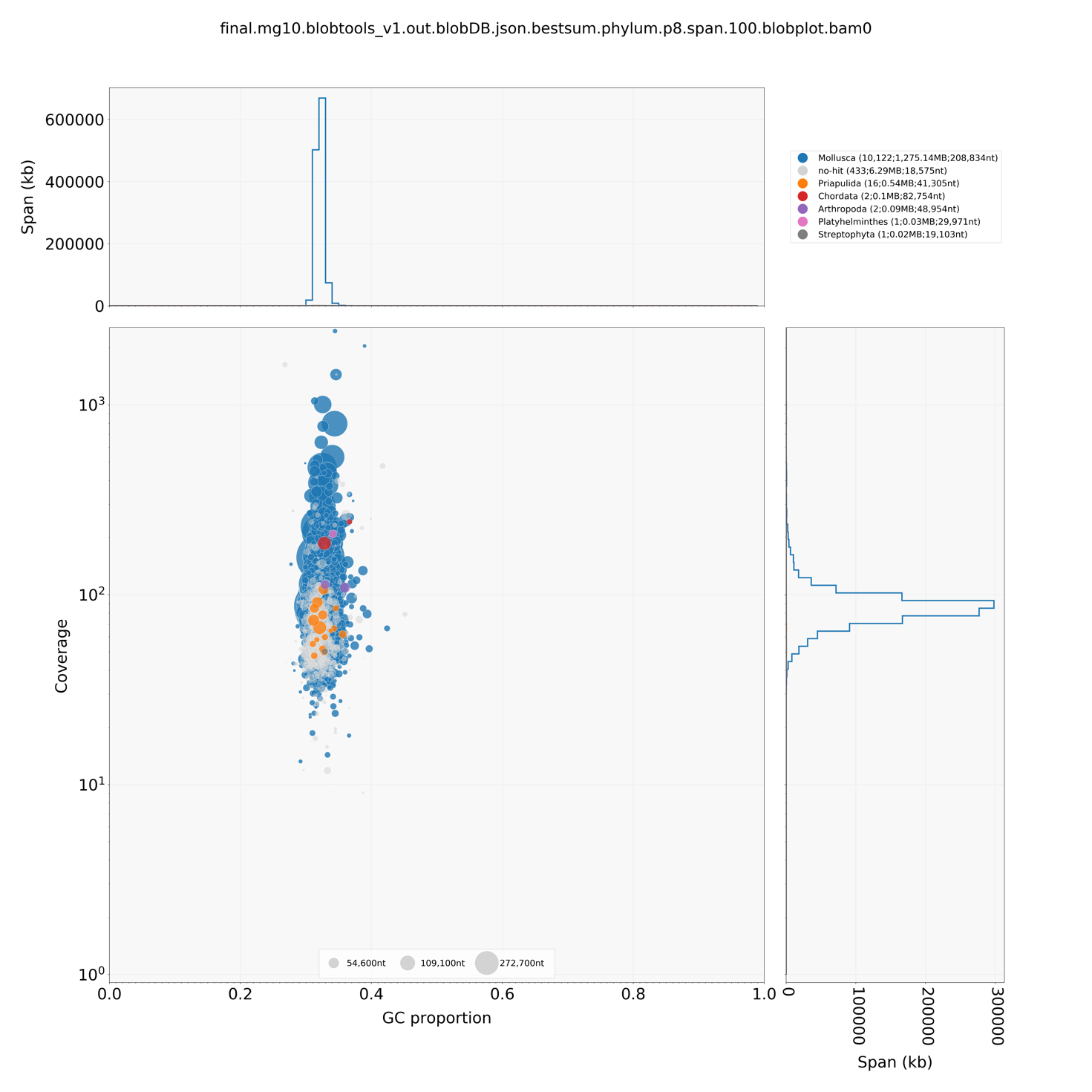


**Fig. S5. Sequencing coverage and GC content of the scaffolds included in the mg10 reference genome assembly**. Note the narrow range of GC distribution of all scaffolds. Coverage is highly influenced by the presence of repeats.

However, we cannot rule out the presence of a marginal contamination by exogenous DNA from other species without considering the lower taxonomic ranks, closer to the species level (**Figures S6**, panels A, B and C). These results clearly pointed out that the overwhelming majority of the assembled sequence belongs to *Mytilus galloprovincialis*. A total of 0.4% are sub-threshold matches (‘no-hit’) and a tiny amount (0.06%, span=1 MB) matches the marine worm (*Priapulus caudatus*), with the rest remain flagged as ‘unresolved/other’. The matches to other species within the same genus (*M. chilensis, M. edulis* and *M. californianus*) can be interpreted due to shared homology and underrepresentation of certain stretches of the genome in the current nt database at NCBI. The case of *Priapulus caudatus* is likely a false positive as it has circumpolar distribution, well out of the range were samples have been collected. Indeed, a closer look to the best hits found with megablast were PREDICTED mRNAs (i.e., computationally-assisted predictions from the genome assembly), with a bitscore lower than 660 and identities below 83.4% (**Additional file 2:** **Table S6**).


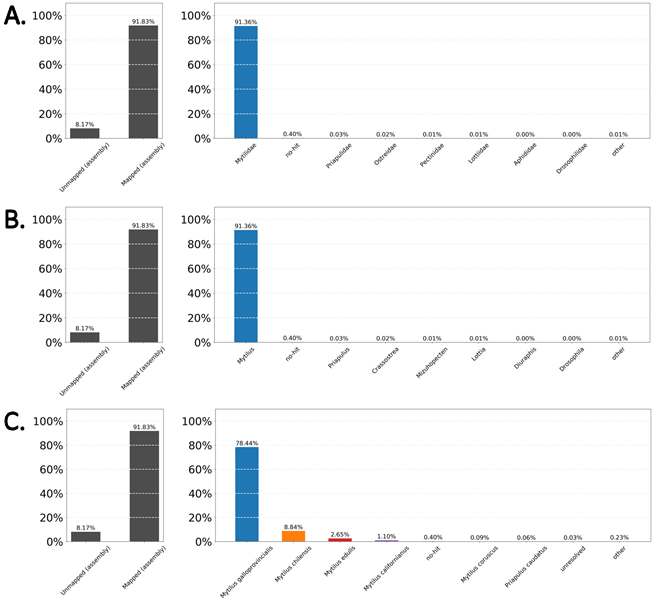


**Fig. S6. ReadCovPlot at the family (A), genus (B) and species (C) level**. The graphs report the results obtained for the reference genome assembly mg10.

In conclusion, we did not find traces of additional contamination from exogenous sources in the reference genome assembly mg10. If present, contigs linked with contamination should be present in negligible amounts, which are not expected to introduce any bias in the gene presence-absence analyses carried out (see **Data Note 8 and 9**).

## Genome assembly evaluation

Our assembly strategy aimed for a haploid or 1n reference that was valid to make inferences based on the coverage and therefore study duplications, gene expansions and gene presence-absence variation (PAV) based on re-mapping.

### Assessment of gene completeness and duplication

The mg10 assembly showed a low level of duplicated genes, as estimated with BUSCO v.3 [25] using 843 genes conserved in metazoans, compared to previous versions of the assembly. In fact, compared to mg4, it had 8.5% instead of 23% duplicated genes. Unfortunately, this was done at the cost of including about 5% less single-copy genes. A more discouraging estimate was the 9.67% decrease in Partial genes detected with CEGMA [9]. However, compared with BUSCO, CEGMA uses a more reduced gene set of 248 ultra-conserved genes. In conclusion, the gene set of mg10 might be slightly less comprehensive compared to previous assembly versions, but it removed most duplication issues.

### The *k-mer* spectra supports a better collapse of heterozygous sequences

In order to evaluate the level of artefactual duplications present in the different assemblies we used the Kmer Analysis Toolkit [26]. This program was used to obtain a stacked histogram based on the 27-mer matrix of the assembled genome and the PE800 library (as its reads are evenly distributed across the genome). Typically, these plots are used to compare a Jellyfish hash produced from a read set to a Jellyfish hash produced from an assembly. We plotted the stacked histogram for three different assembly versions (**Fig. S7**). First, this procedure was applied to mg3, a previous version of the assembly that contained a high level of artefactual duplications. Second, this was applied to mg4, an assembly version that was the product of a self-collapse of mg3 using ASM [6] to overcome this problem, but failed. Finally, this approach was applied to our reference genome (mg10), aimed at representing one unitig per haplotype and along the long-read data.

We plotted the distribution of depth for each distinct *k-mer* (**Fig. S7**). As for the Jellyfish plot (**Fig. S3**), we observed a main homozygous peak, accompanied by a heterozygous peak at half of this depth. The plots show the amount of distinct *k-mers* absent (0x class, in black), as well as the copy number variation present within the assembly. The absent elements are sequences that have not been assembled well (or have low sequence quality producing altered versions of the *k-mer*) in the assemblies. As stated in the Supplementary Figure 8 of the KAT publication [26], a good assembly will report a single haplotype so that half of the bubbles in the heterozygous peak are absent. When the collapse of alleles fails, then both haplotypes are present in the heterozygous part and the homozygous *k-mers* around the bubble are duplicated in the assembly. Therefore, uncollapsed alleles end up as artefactual duplications that are present twice and have a similar depth to the homozygous 27-mers (2x class, see the violet areas above the peak of higher depth).

A comparison of these three plots shows that mg3 and mg4 assemblies failed to represent a single haplotype per heterozygous region, while mg10 did a better job. In fact, almost half of the heterozygous peak is represented by just one distinct *k-mer*, and the level of artefactual duplications was reduced considerably from the reference assembly. However, this was done at the cost of having represented a small portion of the homozygous *k-mers*. Although this could be still due to noisy sequence from the PacBio reads present in the assembly, it is also a trade-off for collapsing the genome.

In summary, mg10 is a fairly complete reference sequence of *M. galloprovincialis* that we believe to be appropriate for the analyses presented here and the inferences based on coverage.

**
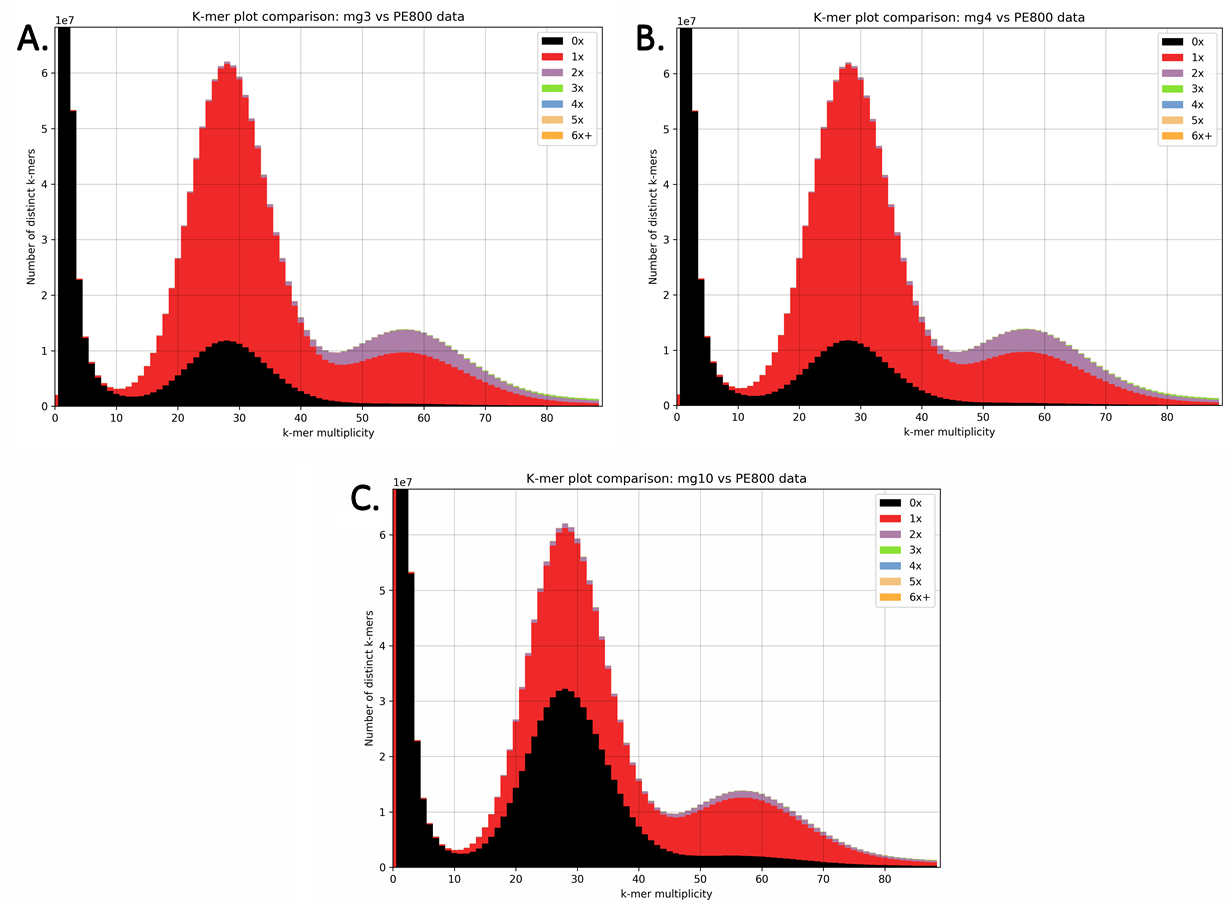
**

**Fig. S7.** **KAT stacked histograms.** A stacked histogram based on the 27-mer matrix of the assembled genome and the PE800 library. Typically, this is used to compare a Jellyfish hash produced from a read set to a Jellyfish hash produced from an assembly. The plot shows the amount of distinct *K-mers* absent (0x class, in black), as well as the copy number variation present within the assembly (1x to 6x, in color). Uncollapsed alleles end up as artefactual duplications that are present twice in the assembly (2x class, in violet) with similar depth to homozygous 27-mers. (A) KAT stacked histogram of mg3 (B) stacked KAT stacked histogram of mg4; a self-collapsed version of mg3. (C) KAT stacked histogram of mg10.

### Comparative overview with previous *de novo* assembly efforts

We and others have already produced *de novo* assemblies of the *M. galloprovincialis* genome [17,27]. However, these efforts were entirely based on Illumina short reads and resulted in genomic sequences suffering from extreme fragmentation, in spite of a global assembly size in line with expectations (about 1.6 Gb in the study by Nguyen *et al.* and 1.5 Gb in the study by Murgarella *et al.*). In both cases, the total number of assembled contigs (organized in scaffolds only by Murgarella *et al.*) exceeded 1 million (reaching 2.3 million in Nguyen *et al.*), with very low N50 assembly values (2.9 and 1 Kb, respectively. The genome assembly we present here, through the use of a hybrid approach considering both long (PacBio) and short (Illumina PE and MP) reads, greatly exceeds the quality of these two previous assemblies (**Fig. S8**). Despite a slightly lower total assembly size (discussed in detail above), mg10 is contained in just 10,577 scaffolds, with a N50 statistics higher than 207 Kb.

**
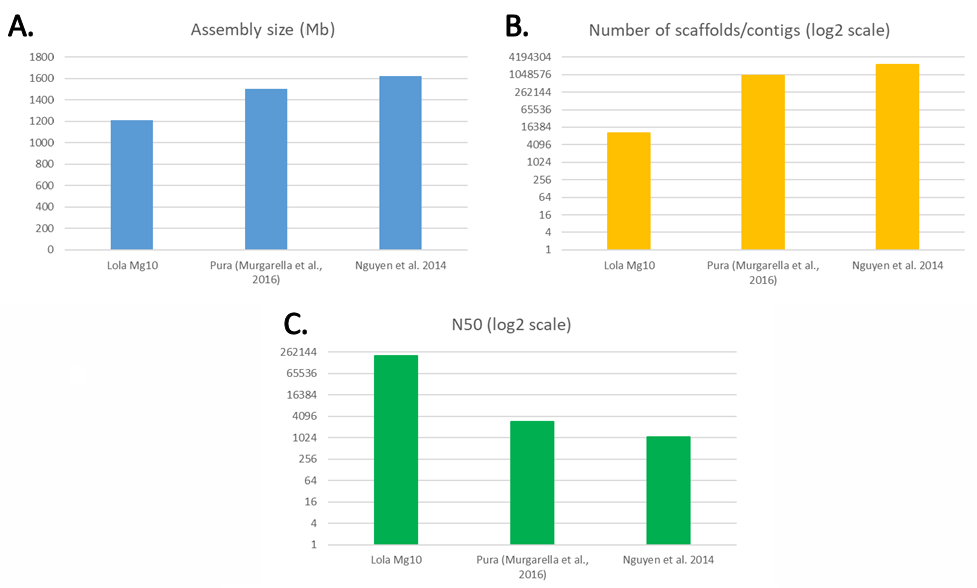
**

**Fig. S8. Comparison of assembly statistics.** (A) total assembly size (Mb), (B) number of contigs/scaffolds and (C) assembly N50 for *Lola* mg10 (this study), and previous efforts from Murgarella *et al.* [17] and Nguyen *et al.* [27]. Note that number of contigs/scaffolds and N50 are plotted in a log_2_ scale. The mg10 genome assembly greatly outperforms previous attempts.

# 2. Data Note 2 – Genome annotation

## 2.1. Generation of consensus gene models

To annotate the mussel genome, we obtained consensus gene models by combining transcript alignments, protein alignments and gene predictions. The annotation pipeline was run on the mg8 version of the genome and gene coordinates were transferred accordingly to the final version, mg10. This was possible because the differences between the two versions of the assembly are minor in terms of new sequence, as it will be detailed in the following paragraphs.

### 2.1.1. Transcript alignment

Transcripts for assembly with PASA (PASA v 2.0.2) [28] were obtained as follows. First, RNA-seq reads obtained in previous studies were downloaded from the NCBI SRA archive [18] and aligned to the *M. galloprovincialis* assembly *mg8* with STAR (v-2.5.1b). Transcript models were subsequently generated using Stringtie v1.0.4, resulting in 617,565 transcript models, which were then added to the PASA database. In addition, we collected 41,242 *M. galloprovincialis* mRNAs present in NCBI (data retrieved on December 3rd, 2014). All of the above transcripts were then assembled on the genome by PASA, resulting in 643,809 PASA assembled transcripts.

### 2.1.2. Protein alignment

We aligned all the bivalve proteins present in Uniprot (data retrieved on December 11th, 2014) to the mussel genome with SPALN v2.2.2 [29] resulting in 319,043 CDS alignments.

### 2.1.3. Repeat finding

Before performing *ab initio* gene prediction, the genome needs to be masked for repetitive elements, especially transposons. An adequate repeat library did not exist yet for mussel, so we constructed one using elements found by the program RepeatModeler with additional repeats found by MITE-hunter as well as oyster repeats. The repeat library was searched for non-transposable element proteins belonging to large protein families that were erroneously classified as repetitive elements and removed. The type and number of repeats identified is summarized in **Table S8**. The repeat library is available upon request.

**Table S8**. Repetitive elements present in the *M. galloprovincialis* mg10 assembly.

| **Class of repeat** | **Number of repeats** | **Total nucleotides** | **Percentage of the genome** |
| --- | --- | --- | --- |
| **Retrotransposons** | 266,574 | 81,193,011 | 6.33 |
| SINE | 10,200 | 2,445,118 | 0.19 |
| LINE | 239,279 | 73,206,166 | 5.71 |
| LTR | 17,095 | 5,541,727 | 0.43 |
| **DNA TEs** | 377,648 | 109,722,070 | 8.56 |
| CMC | 3,897 | 794,652 | 0.06 |
| hAT | 46,509 | 9,603,071 | 0.75 |
| Maverick | 1,965 | 890,504 | 0.07 |
| TcMar | 18,404 | 5,513,928 | 0.43 |
| Mule-MUDR | 3,612 | 724,938 | 0.06 |
| PIF-Harbinger | 26,907 | 5,897,250 | 0.46 |
| Helitron | 143,256 | 51,733,774 | 4.03 |
| Other DNA Tes | 133,098 | 34,563,953 | 2.70 |
| **Simple Repeats** | 1,917 | 666,317 | 0.05 |
| **Satellite** | 31,891 | 13,785,526 | 1.08 |
| **Other** | 1,584,624 | 363,358,778 | 28.34 |
| **Total** | 2,262,654 | 568,725,702 | 44.36 |

### 2.1.4. *Ab initio* gene predictions

*Ab initio* gene predictions were performed on the mg8 assembly masked for repeats found with RepeatMasker (A.F.A. Smit, R. Hubley & P. Green RepeatMasker at [http://repeatmasker.org](http://repeatmasker.org/)) v4-0-5 using the custom library created above. A 43.01% of the genome assembly was masked after this step. Low complexity repeats were left unmasked for this purpose.

Four different gene prediction programs were used: GeneID [30], GeneMark-ES [31], GlimmerHMM [31] and Augustus [32]. GeneID *ab initio* gene predictions were obtained by running GeneID v1.4 with the parameter file specific for *M. galloprovincialis* that we had previously generated by using 500 mussel genes with *bona fide* gene structures obtained from PASA. *M. galloprovincalis* protein-coding gene annotations were also obtained using the gene prediction tool Augustus v3-0-2. For this purpose, and for GlimmerHMM, we also trained the program to generate a species-specific parameter file. The other gene predictor, Genemark-ES was run in its self-training manner. The number of predicted gene models ranged from 133,199 with GlimmerHMM to 78,493 with GeneID.

GeneID, Genemark and Augustus were also used to generate predictions incorporating intron evidence, which was extracted from the RNA-seq data using STAR. Those canonical junctions overlapping *ab initio* GeneID predictions, Augustus predictions or protein mappings were taken as intron evidence. For Augustus, exon information as well as intron evidence derived from the RNA-seq mappings were also included.

## 2.2. Generation of consensus CDS models

The transcript alignments, protein alignments and the *ab initio* gene models were combined into consensus CDS models using EvidenceModeler-1.1.1 (EVM). EVM was run with three different sets of weights (**Table S8**) and the resulting consensus models with the best specificity and sensitivity as determined by intersection (BEDTools [33]) with the transcript mappings were chosen for the final annotation of mg9.

**Table S8. EVM weights**

| **Type of evidence** | **Software** | **EVM weight** |
| --- | --- | --- |
| ABINITIO_PREDICTION | GeneMark | 1 |
| ABINITIO_PREDICTION | Augustus | 2 |
| ABINITIO_PREDICTION | GeneID | 2 |
| ABINITIO_PREDICTION | geneid_introns | 2 |
| ABINITIO_PREDICTION | GlimmerHMM | 2 |
| ABINITIO_PREDICTION | Augustus+ | 1 |
| ABINITIO_PREDICTION | GeneMark-ET | 2 |
| TRANSCRIPT | PASA | 8 |
| PROTEIN | SPALN | 10 |

The consensus CDS models were then updated with UTRs and alternative exons through two rounds of PASA annotation updates. A final round of quality control was performed, fixing reading frames, intron phases and removing some transcripts subjected to nonsense-mediated decay (NMD). The resulting transcripts were clustered into genes using shared splice sites or significant sequence overlap as criteria for attributing them to the same gene. Systematic identifiers with the prefix “MGAL8A” were assigned to the genes, transcripts and protein products derived from them. Support by source of evidence at the gene and exon level was determined *a posteriori* using BEDTools *intersect* and *multiinter* programs. A total of 67,608 genes, 90,558 transcripts and 86,189 proteins resulted from this step.

For details about a comparative assessment of genome completeness and integrity of predicted gene models among the different assembly versions, see **Additional file 2:** **Table S4**. For a comparative assessment of genome completeness and integrity of predicted gene models between *M. galloprovincialis* and other molluscan species, see **Data Note 3**. Statistics for all major stages of the assembly process are given in **Additional file 2:** **Table S3**.

When the mg9 version of the assembly was obtained, the annotation previously obtained for mg8 was transferred to the new assembly version. The only genes that changed in this case were the ones present in different mg8 scaffolds that were joined in the mg9 version due to RNA-seq evidence. Systematic identifiers with the prefix “MGAL9C” were assigned to the genes, transcripts and protein products derived from them. This step resulted in 67,591 genes, 90,410 transcripts and 86,063 proteins. The functional annotation pipeline was run on this version of the annotation.

## 2.3. Functional annotation of protein-coding genes

For the functional annotation of MGAL9C we used InterPro [34], KEGG [35], Blast2GO [36], SignalP [37], and NCBI CDsearch [38] databases. InterProScan v.5.19-58 [39] was used to scan though all available InterPro databases, including PANTHER, Pfam, TIGRFAM, HAMAP and SUPERFAMILY. BLASTP v.2.2.29+ search against NCBI non-redundant (NR) collection of protein sequences (release 2016-09) was used as input to the local Blast2GO software p2gpipe version 2.5.0, database update 2016-08. KEGG orthology (KO) groups were assigned by KEGG Automatic Annotation Server (KAAS) [40] using bi-directional best hit (BBH) method against a representative gene set from 32 different species, including mollusk species *Lottia gigantea* (owl limpet) [41] and *Crassostrea gigas* (Pacific oyster) [42]. KO identifiers were then used to retrieve using the KEGG REST-based API service the KEGG relevant functional annotation, KEGG release v.79.1.

A total of 67,440 (78.70%) out of 86,063 proteins had some type of annotation feature derived from one of the annotation resources used in this work. GO terms were assigned to 42,361 (49.22%) proteins. Additionally, we were capable of assigning a description (name) to 22,659 (26.33%) proteins using Blast2GO or KEGG (**Table S9**). The majority of the annotated mussel proteins had Blast hits belonging to the Mollusca clade (**Fig. S6**).

**Table S9. Functional annotation results**

| **metric** | **genes** | **proteins** |
| --- | --- | --- |
| Total number | 67,591 | 86,063 |
| Annotated | 51,138 (75.66%) | 67,440 (78.36%) |
| Interpro signatures | 49,163 (72.74%) | 64,914 (75.43%) |
| Blast2GO or KEGG definition | 14,986 (22.17%) | 22,659 (26.33%) |
| Blast2GO definition | 9,295 (13.75%) | 13,298 (15.45%) |
| KEGG definition | 11,297 (16.71%) | 17,416 (20.24%) |
| Assigned to KO groups | 11,319 (16.75%) | 17,447 (20.27%) |
| With GO terms association | 30,557 (45.21%) | 42,361 (49.22%) |
| Conserved domains signatures | 43,283 (64.04%) | 58,022 (67.41%) |
| Conserved features signatures | 18,736 (27.72%) | 25,725 (29.89%) |
| SignalP signatures | 6,899 (10.21%) | 8,649 (10.05%) |


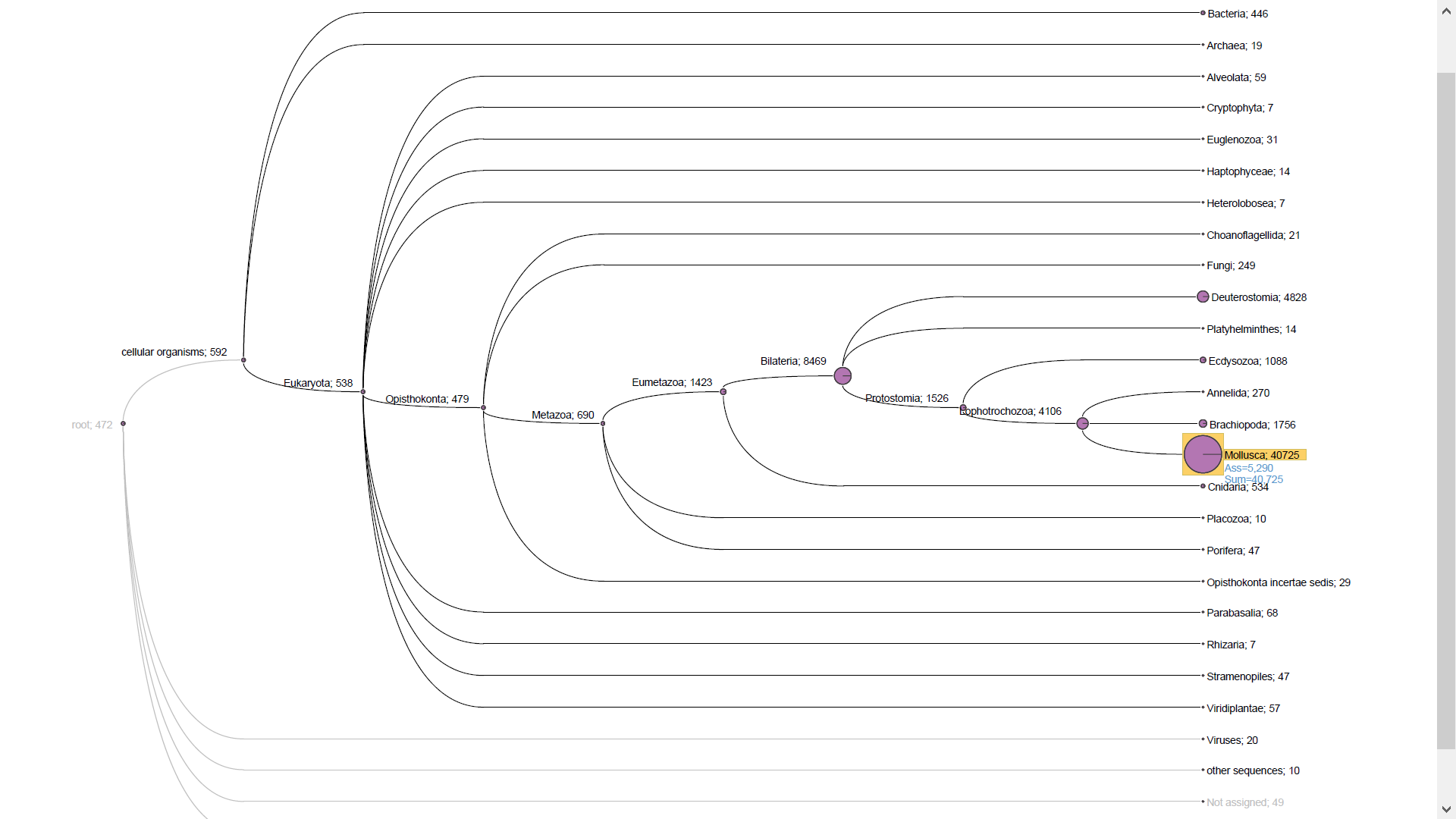


**Fig. S9. BLAST hits distribution of MGAL8A through NCBI taxa**. The diameter of the circles shown close to each taxon is proportional to the number of hits found.

## 2.4. Filtering of MGAL9C annotations

After the functional annotation, we combined these results with the source of evidence that supported each gene model and proceeded to do a filtering of those genes that were monoexonic, supported only by *ab initio* gene predictions and lacking functional annotation. Moreover, we selected the transposable element candidates and transferred them to the non-coding gene annotation, tagging them as “TE-derived” genes. In total, we removed 7,083 transcripts and moved 2,811 to the non-coding annotation. Hence, the resulting MGAL9D annotation comprised 58,125 genes, whose 79,966 transcripts encode 75,788 unique protein products.

## 2.5. Non-coding RNA annotation

We annotated non-coding RNAs (ncRNAs) on the mg9 assembly by running the following steps. First, the program *cmsearch* (v1.1), part of the Infernal package [43] was run against the RFAM database of RNA families (v12.0) [44]. Also, tRNAscan-SE (v1.23) [45] was run in order to detect the transfer RNA genes present in the genome assembly. To detect long non-coding RNAs (lncRNAs) we first selected PASA-assemblies that had not been included in the annotation of protein-coding genes, i.e., expressed genes that were not translated to protein. Those that were longer than 200 bp and whose length was not covered at least 80% by a small ncRNA were incorporated into the ncRNA annotation as lncRNAs. The resulting transcripts were clustered into genes using shared splice sites or significant sequence overlap as criteria for designation as the same gene. Systematic identifiers with the prefix “MGAL9ncA” were assigned to the genes and transcripts derived from them. A total of 73,650 ncRNA genes and 75,644 non-coding transcripts were annotated. After filtering the transposable element-like genes from the MGAL9C protein-coding gene annotation, 2,925 more genes were added to the non-coding annotation under the tag “TE-derived”. The final MGAL9ncB annotation comprised 75,973 genes, 78,569 transcripts and 102,225 exons (1.3 exons/transcript).

## 2.6. MGAL10B annotation

When the mg10 version of the assembly was obtained, the MGAL9D annotation was transferred to the new version of the assembly. However, a particular region in this assembly that cointained the myticin gene cluster (which all belonged to a single scaffold) changed significantly. Therefore, we run the same annotation process described above for the new scaffolds. Finally, we added the new genes derived from the aforementioned scaffold to the ones that resulted from transferring the previous annotation to mg10 coordinates and filtered out the genes present in the “old myticin scaffolds”. This step produced the “MGAL10A” annotation, which after some manual curation of a few genes, was updated to “MGAL10B”.  Statistics of the annotation are given in **Table S10**.

**Table S10. MGAL10B Annotation statistics**

| Number of genes | 60,302 |
| --- | --- |
| Median gene length (bp) | 4,297 |
| Number of transcripts | 83,073 |
| Median transcript length (bp) | 1,233 |
| Number of proteins | 78,736 |
| Median coding sequence length (bp) | 981 |
| Median exon length (bp) | 140 |
| Median intron length (bp) | 901 |
| Median UTR length (bp) | 368 |
| Coding GC content | 37.78% |
| Exons/transcript | 6.72 |
| Transcript/gene | 1.38 |

# 3. Data Note 3 - Comparative assessment of genome completeness

## 3.1. Genome completeness and gene model integrity evaluation

The longest protein encoded by each gene model was selected for *Lola* and for the most recent release of the publicly available genomes of the bivalves *Mytilus coruscus* [46], *Limnoperna fortunei* [47], *Bathymodiolus platifrons* [48], *Modiolus philippinarum* [48], *Crassostrea gigas* [42], *Crassostrea virginica* [49], *Pictada fucata* [50], *Mizuhopecten yessoensis* [51], Pecten *maximus* [52], *Ruditapes philippinarum* [53], *Argopecten purpuratus* [54], *Saccostrea glomerata* [55], *Sinonovacula constricta* [56], *Dreissena polymorpha* [57], *Dreissena rostriformis* [58], *Venustaconcha ellipsiformis* [59], the gastropods *Lottia gigantea* [41], *Haliotis discus* [60], *Aplysia californica* (unpublished), *Biomphalaria glabrata* [61] and the cephalopod *Octopus bimaculoides* [62].

The completeness and integrity of these gene models were assessed with BUSCO v3 [25], using the Metazoa OrthoDB v9.1 universal single copy orthologous gene set [63] as a reference. The gene models were consequently catalogued as “complete”, “duplicated”, “fragmented” or “missing”.

The results (**Fig. S10**) highlighted a rather complete picture for the *Lola* mg10 assembly, with 85% of the expected conserved genes present and complete (9% out of these were marked as duplicated), 6% as fragmented and just 9% as missing. Compared to the other three recently released Mytilidae genomes, the *M. galloprovincialis* genome annotation shows a much higher degree of completeness and integrity with respect to *L. fortunei* and *M. philippinarum*. The mussel genome shows a slightly less complete gene set compared to *B. platifrons* (a species with low heterozygosity), but in turn shows a lower number of fragmented gene models. Compared with *M. coruscus*, a congeneric species with a lower level of heterozygosity [46], *M. galloprovincialis* shows a slightly higher number of missing, a similar number of fragmented and a lower number of duplicated BUSCOs.

Overall, the quality of the mussel genome appears to be very similar to that of the Pacific oyster *C. gigas* [42] and the pearl oyster *P. fucata*, both in terms of fragmented and absent gene models, and only inferior to the much less heterozygous genome of the scallop *M. yessoensis* [51], and to the high quality genome assemblies of the oysters *C. virginica* and *S. glomerata* [49,55]. Both parameters were however inferior to four out of five available non-bivalve molluscan genomes, which can be explained be their lower size, heterozygosity and overall complexity.


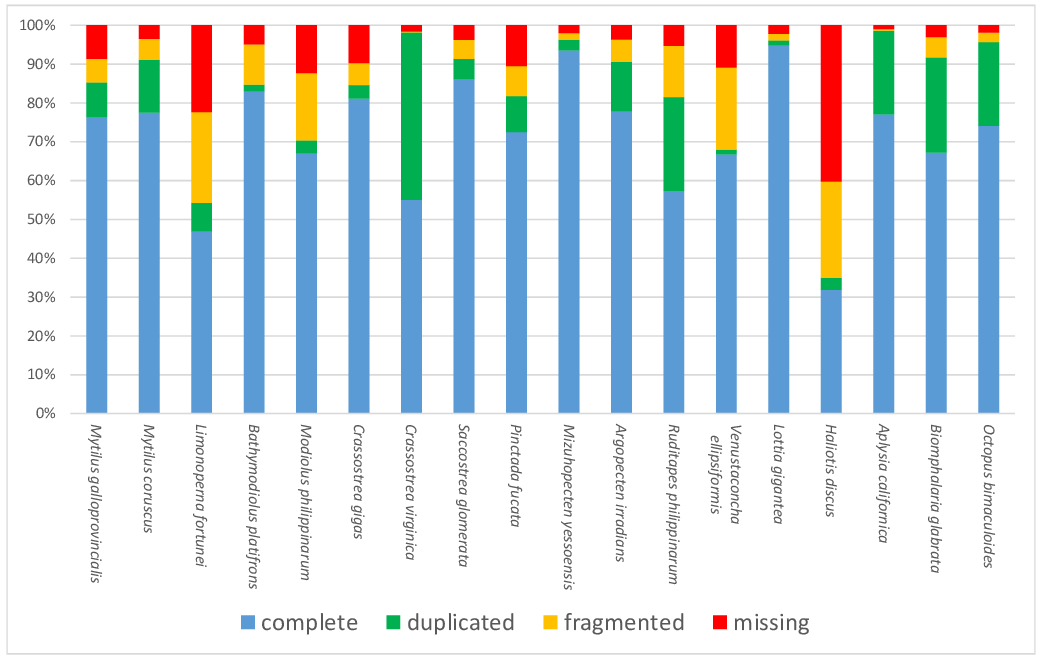


**Fig. S10. Comparative assessment of genome completeness and integrity of predicted gene models.** The evaluation was performed with BUSCO v3 using the Metazoa OrthoDB v9.1 universal single copy orthologous gene set.

## 3.2. Genome size

The global assembly size of the *Lola* genome was slightly lower than the congeneric species *M. coruscus* [46] and those of the three other available mytilid genomes (shown in red in **Fig. S11**), but higher than most of all the non-mytilid genomes sequenced to date (shown in light orange), with the exceptions of *R. philippinarum*, *D. polymorpha* and *V. ellipsiformis*, standing at a total length lower than 1 Gb. The size of the mussel genome falls well within the range of those previously sequenced in other molluscan species (shown in purple). Overall, the assembled genome size of the mussel genome is consistent with previous estimates obtained with cytogenetic studies in the same species [64,65], and falls within the ranges expected from Mytilida (observed C-values between 1.06 in *Mytilisepta keenae* [64] and 2.50 in *Lithophaga bisulcata* [62]) (**Fig. S12**).


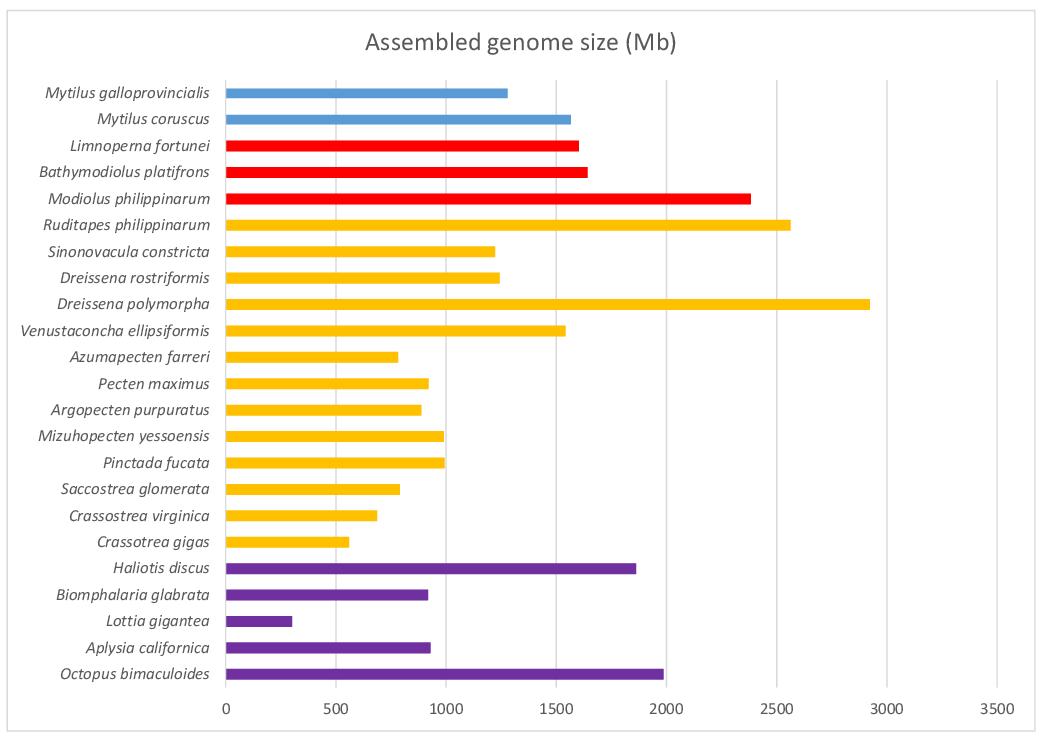


**Fig. S11**. **Comparative genome size of *M. galloprovincialis* and other molluscan genomes.**


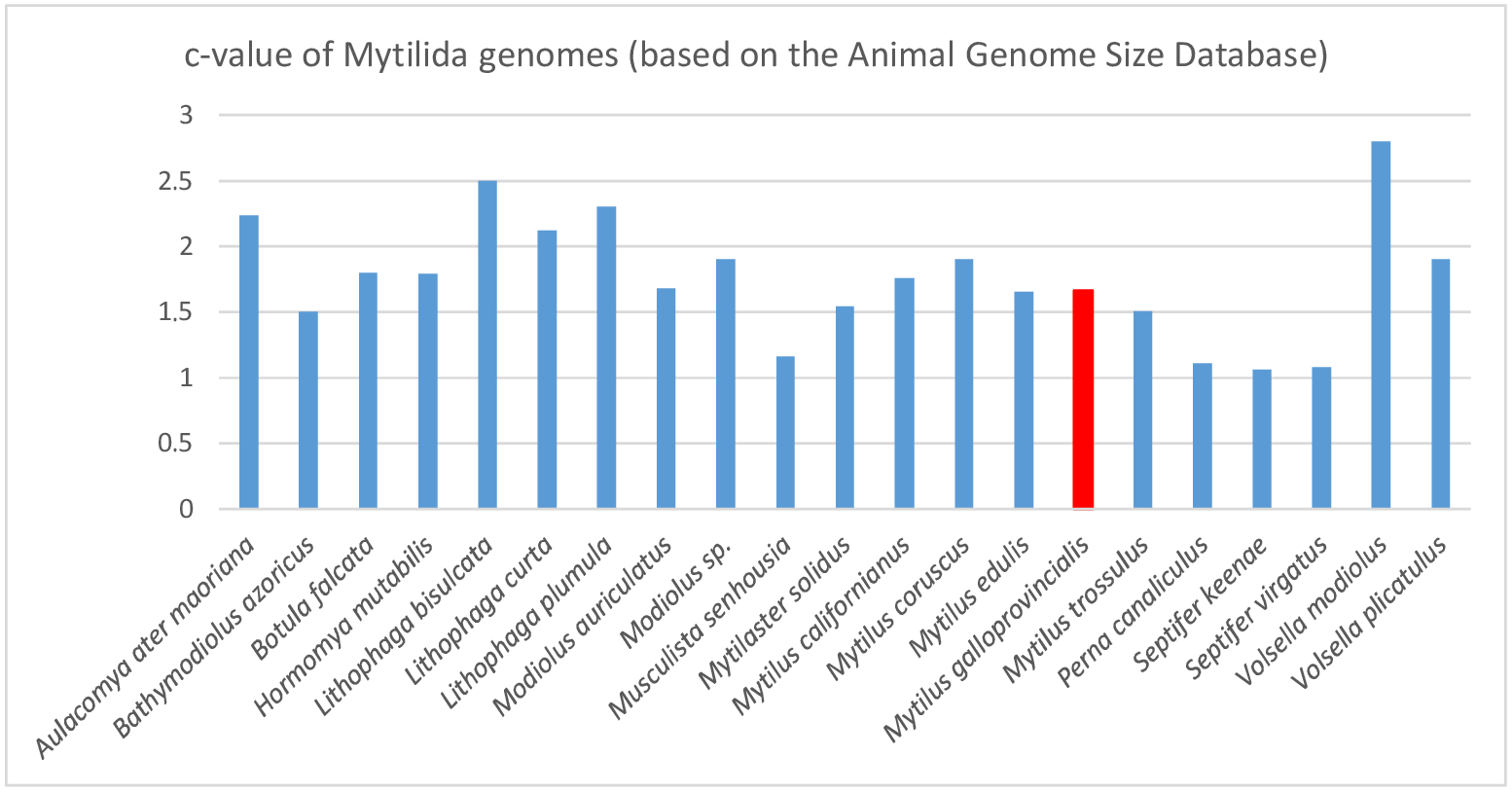


**Fig. S12**. **C-values of the genomes of Mytilidae, base on the Animal Genome Size Database data.** The values reported here represent the average of those reported for each species. *M. galloprovincialis* is evidenced in red.

## 3.3. Protein-coding gene number

The number of predicted protein-coding genes appeared to be very high compared to non-bivalve mollusks and, in general, to most metazoans (e.g. the human, zebrafish and fruit fly genomes contain 20,376, 25,591 and 13,931 genes, respectively). However, this estimate is in line with the those from the golden mussel *L. fortunei* [67], the zebra mussel D. *polymorpha* [57] and the king scallop *P. maximus* [52], and significantly lower than that of *R. philippinarum* (with over 100,000 predicted gene models) [53]. Compared with the congeneric *M. coruscus* [46] the Mediterranean mussel genome encoded roughly 30% more protein-coding genes (**Fig. S13**).


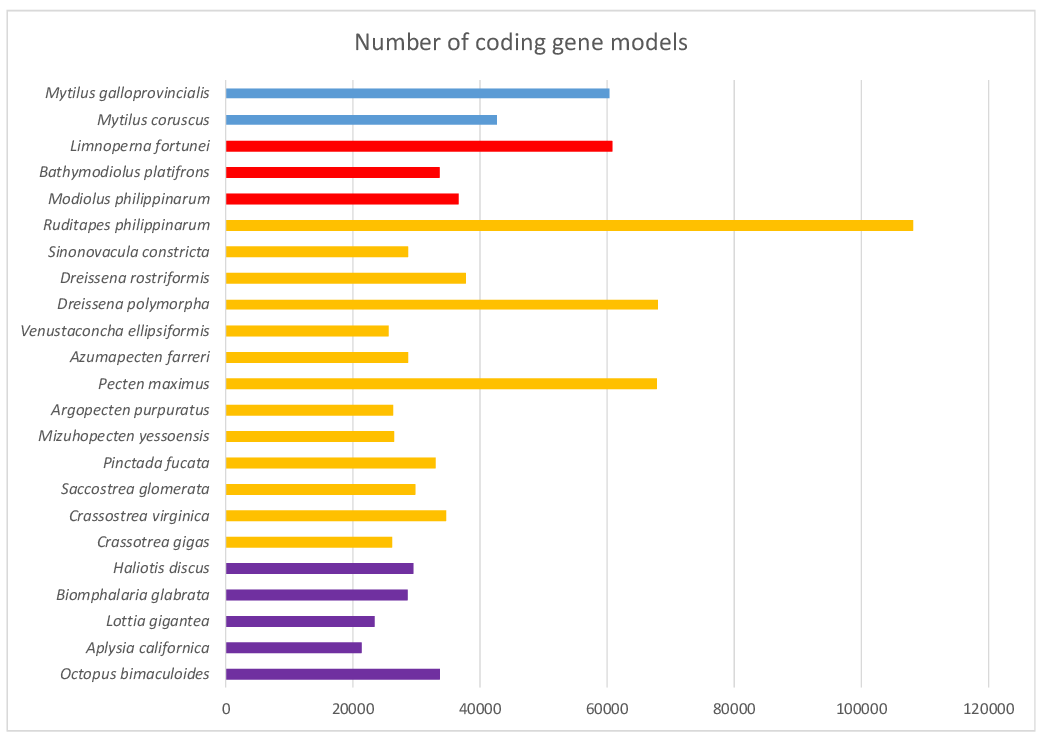


**Fig. S13. Comparative overview of the number of gene models in *M. galloprovincialis* and other molluscan genomes.**

## 3.4. Gene density

The observed gene density, close to 50 genes/Mb, also found *L. fortunei* as the closest match within Mytilida, and was in line with the values found in the smaller genes from oyster, scallops, and most gastropods (**Fig. S14**).


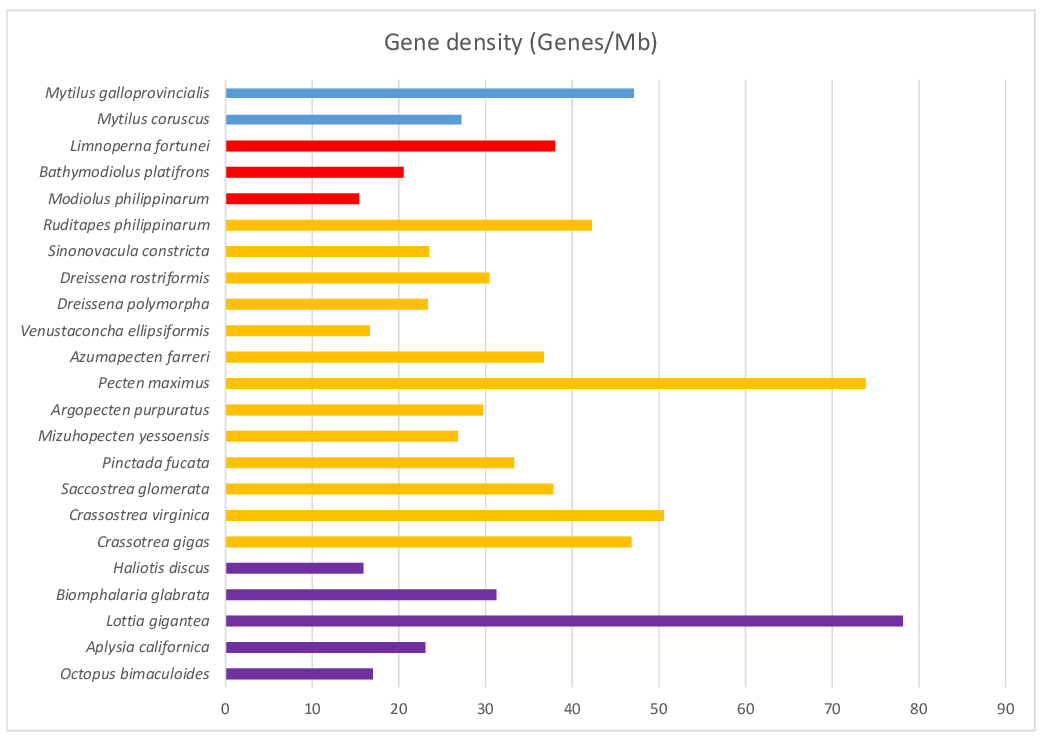


**Fig. S14. Comparative overview of the gene density (genes/Mb of assembled genome) in *M. galloprovincialis* and other molluscan genomes.**

# 4. Data Note 4 – Support of gene models by RNA-seq data

## 4.1. Transcriptomic datasets used

We mapped a total of 51 different RNA-seq datasets to the annotated *Lola* genome. This procedure was carried out with the aim to gather information about the transcriptomic support of mg10 gene models, with a greater depth compared to the preliminary analyses detailed in **Data Note 2.**

Publicly available RNA-seq data from *M. galloprovincialis* were downloaded from the NCBI SRA database (**Table S11**). In addition, digestive gland and gill transcriptomes were obtained from *Lola*, as described in detail below. Overall, these data accounted for the following tissues: digestive gland (6 samples + *Lola*), whole body (7 samples), hemocytes (28 samples), mantle (5 samples) posterior adductor muscle (2 samples) and gills (1 sample + *Lola*) [18,68–70].

**Table S11. Accession ID (NCBI SRA) and tissue of origin of the RNA-seq datasets used for this analysis.**

| **Accession** | **tissue** |
| --- | --- |
| SRX126945 | digestive gland |
| SRX126946 | digestive gland |
| SRX126947 | digestive gland |
| SRX126948 | digestive gland |
| SRX126949 | digestive gland |
| SRX126950 | digestive gland |
| SRX3198556 | whole body |
| SRX565225 | whole body |
| SRX565226 | whole body |
| SRX565227 | whole body |
| SRX565228 | whole body |
| SRX565229 | whole body |
| SRX565230 | whole body |
| SRX386628 | hemocytes |
| SRX389338 | hemocytes |
| SRX389462 | mantle |
| SRX389463 | mantle |
| SRX389464 | posterior adductor muscle |
| SRX389465 | posterior adductor muscle |
| SRX389466 | gills |
| SRR2392495 | anterior mantle |
| SRR2392762 | mid mantle |
| SRR2409049 | posterior mantle |
| SRP145077 | hemocytes (24 samples) |
| ERR4296980 | digestive gland (*Lola*) |
| ERR4296979 | gill (*Lola*) |

## 4.2. Transcriptomic support calculation

Mapping of RNA-seq reads to the reference genome was carried out with the *RNA-seq mapping* tool included in the CLC Genomics Workbench 11 (Qiagen, Hilden, Germany), setting the mapping thresholds to 0.75 (length fraction) and 0.98 (similarity fraction), upon trimming raw reads for the removal of low quality and ambiguous nucleotides (0.05 quality threshold). Reads were aligned to the reference genome (*Lola* mg10) and raw read counts for each gene were converted to Transcript Per Million (TPM) values, which enable accurate comparability both between and within samples [71]. These values were transformed by log_10_ to build the gene expression heat map shown in **Fig. S15**, which only depicts genes reaching expression values higher than 100 TPM in at least one of the 51 samples analyzed. Genes were hierarchically clustered based on Euclidean distances, calculated using the average linkage criterion.


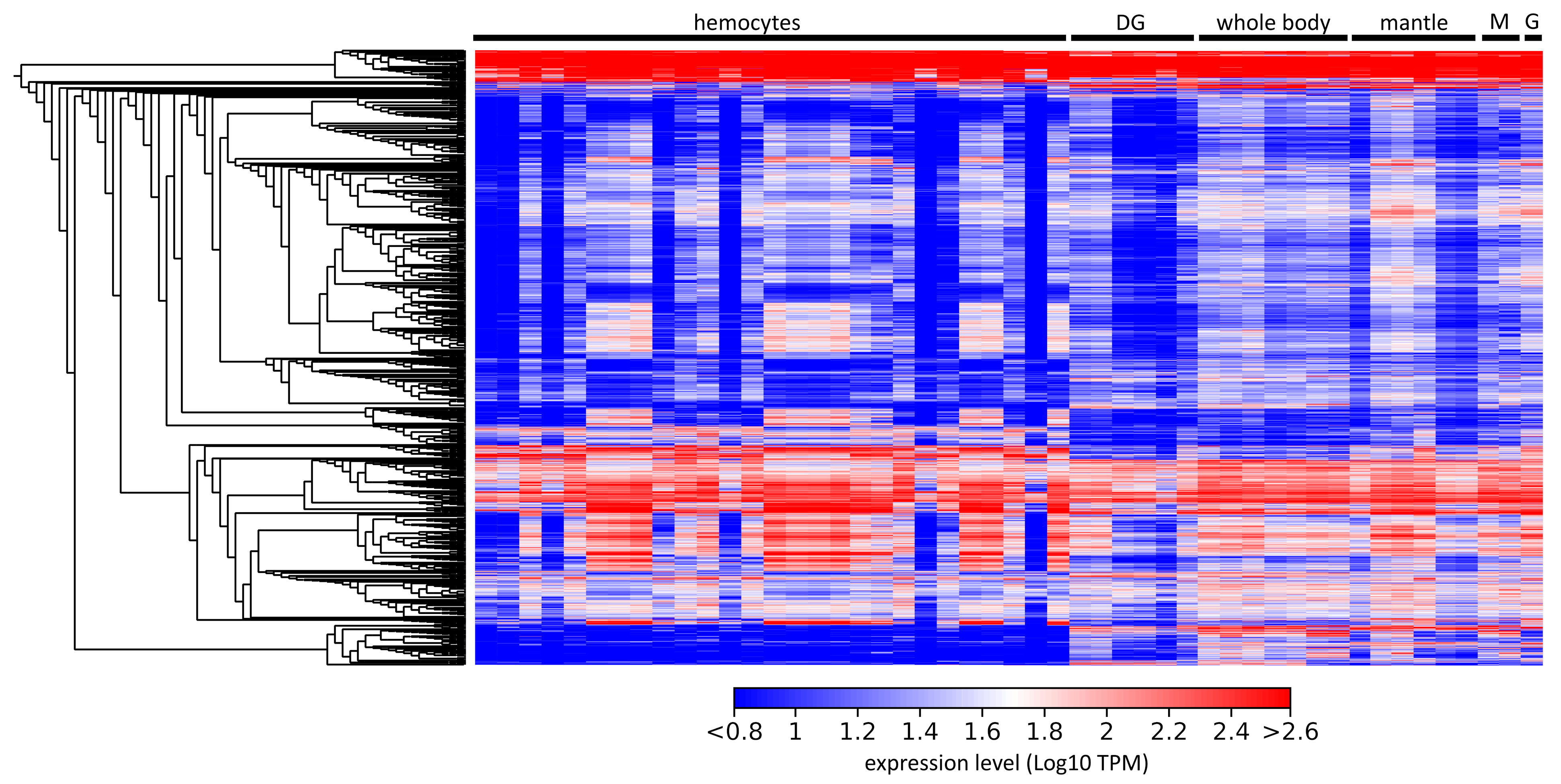


**Fig. S15**. **Heat map summarizing gene expression data from the 51 SRA datasets analyze**d (**Table S11**). Only genes attaining an expression level higher than 100 TPM in at least one sample are shown. Genes were hierarchically clustered based on Euclidean distance, calculated using an average linkage criterion. DG, M and G indicate digestive gland, posterior adductor muscle and gill tissues of mussel.

The transcriptomic support of each gene was calculated as follows:

1. Poor support: the expression level did not reach values > 1 TPM in any of the analyzed samples.
2. Mild support: the expression level reached values higher than 1 TPM, but lower than 3 TPM in at least one of the analyzed samples
3. High support: the expression level reached values higher than 3 TPM in at least one of the 51 analyzed samples

These stringent thresholds were set by keeping in mind the possibility that some of the gene models might not be expressed in the tissues available or produced under the environmental/experimental conditions used. For example, gene expression datasets for early larval stages of *M. galloprovincialis* are essentially unavailable and therefore some strictly developmentally regulated genes are expected to be missing and, therefore, classified either within the “poor support” or “mild support” categories. The same considerations are valid for sex-specific genes expressed in gonads, genes regulated by stressors, pollutants, toxins and pathogens, and *dispensable* genes (which will be discussed in detail below).

Overall, 19,001 gene models (31.66% of the total) were supported by low expression levels, not reaching the arbitrarily set threshold of 1 TPM in any of the 51 samples examined (2,440 genes showed no trace of expression). A total of 9,779 gene models (16.21% of the total) were supported by mild expression support (1 <= TPM < 3), whereas the remaining 26,444 genes (56.17% of the total= were supported by strong transcriptomic evidence (**Fig. S16**).


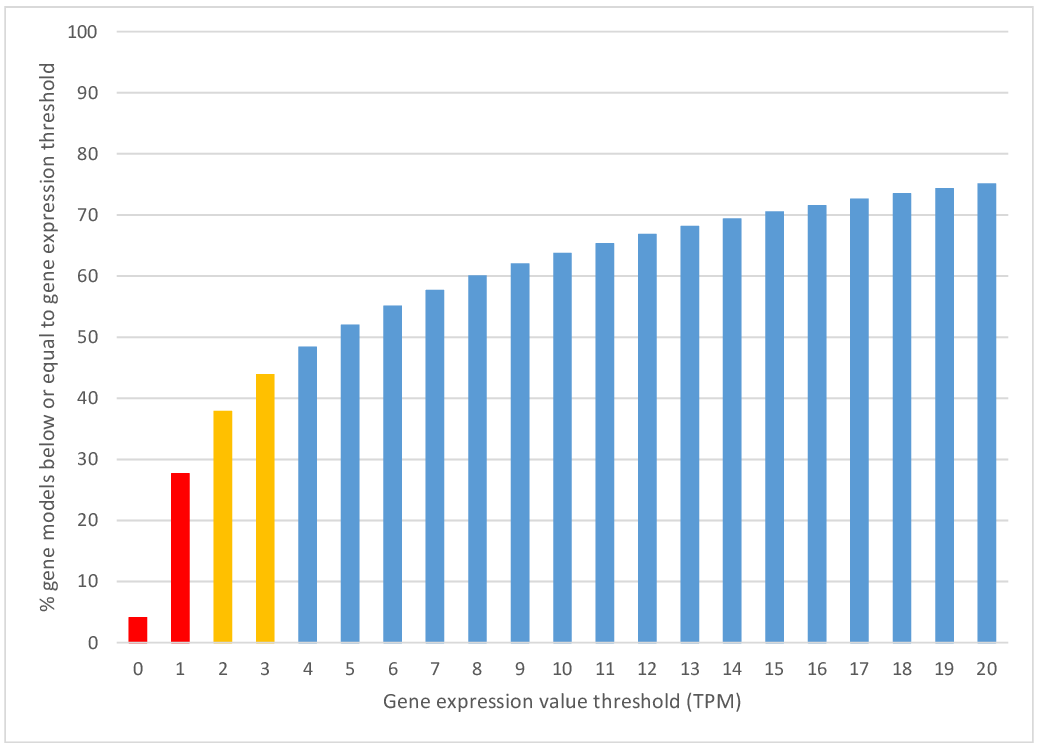


**Fig. S16**. **Percentage of genes showing expression levels below a given threshold** (considering the maximal expression level observed in the 51 analyzed samples). Genes with poor transcriptomic support (maximal TPM < 1) are shown as a red bar in the histogram. Genes with mild transcriptomic support (maximal TPM between 1 and 3) are shown by orange bars. Genes with strong transcriptomic support (maximal TPM > 3) are depicted by light blue bars in the histogram.

## 4.3. *Lola* transcriptome

*Lola* gill and digestive gland tissues were sampled, snap-frozen and maintained at -80ºC prior to RNA extraction. Total RNA isolation was carried out using TRIzol (Invitrogen, Carlsbad, USA) following the manufacturer’s protocol. Purification of RNA after DNase I treatment was performed with RNeasy mini (Qiagen, Hilden, Germany). Next, the concentration and purity of the RNA were measured using a NanoDrop ND1000 spectrophotometer (NanoDrop Technologies, Inc., DE, USA) and RNA integrity was tested on an Agilent 2100 Bioanalyzer (Agilent Technologies, Santa Clara, USA) (RIN values: Digestive gland, 7.4; Gills, 9.3) to produce cDNA libraries for Illumina sequencing.

Next-generation sequencing was performed using Illumina HiSeq™ 4000 technology in Macrogen Korea, using a 2x101 paired-end sequencing strategy, following the protocol of a TruSeq Stranded mRNA LT Sample Prep Kit (Illumina, San Diego, USA). Briefly, mRNA was extracted from total RNA using oligo (dT) magnetic beads and cleaved into short fragments using fragmentation buffer. A cDNA library compatible with the Illumina NGS technology was then prepared from the fragmented mRNA via reverse transcription, second-strand synthesis and ligation of specific adapters (paired-ends) after cDNA purification using the QIAquick PCR Purification Kit (Qiagen, Hilden, Germany). The amount of cDNA in each library was quantified through spectrofluorometric analysis using a Qubit fluorometer.

CLC Genomics Workbench, v.10.0.1 ([Qiagen,](http://www.clcbio.com) Hilden, Germany) was used for raw sequencing data trimming and *de novo* assembly. Raw reads were trimmed to remove adaptor sequences, low quality sequences (quality score limit 0.05), and residual reads shorter than 70 bp. Then, a reference global transcriptome of the two tissues was *de novo* assembled with a minimum contig length of 200 bp and RNA-seq analysis (mismatches = 2, length fraction = 0.8, similarity fraction = 0.8, and maximum hits per read = 10) was constructed. The Blast2GO software [36] was used to annotate contigs by a blastX approach against Uniprot/Swissprot database, with an e-value threshold of 10e10^-5^. Subsequently, GO terms were assigned to the identified contigs.

The main statistics of the *Lola* transcriptome assembly and annotation process are shown in **Table S12**.

**Table S12: Summary of the *Lola* transcriptomes sequencing, *de novo* assembly and annotation.**

| ***Lola* transcriptomes** | **Gill** | **Digestive gland** |
| --- | --- | --- |
| Raw reads | 133,860,870 | 105,504,942 |
| Trimmed reads | 133,427,976 (99.68%) | 105,025,499 (99.55%) |
| **Assembly** |  |  |
| Contigs | 135,969 | |
| N50 | 844 | |
| Mean length | 639 | |
| Range length | 200 - 11,582 | |
| **Annotation** |  |  |
| Contigs Blasted | 41,971 (30.87%) | |
| Contigs with GO terms assigned | 41,529 (30.54%) | |
| Contigs with KEGG pathway assigned | 9,127 (6.71%) | |

# 5. Data Note 5 – Phylome reconstruction

## 5.1. Mussel phylome reconstruction

The mussel phylome, comprising all evolutionary histories of mussel protein-coding genes was reconstructed using the PhylomeDB pipeline [72]. In brief, for each protein-coding gene in the reference mussel genome (mg10) we searched for homologs (Smith-Waterman Blast search, e-value cutoff < 1e-05, minimum contiguous overlap over the query sequence cutoff 50%) in a protein database containing the proteomes of the 16 species considered (**Table S13**). The most similar 150 homologues were aligned using three different programs (MUSCLE [73], MAFFT [74] and KALIGN [75] in forward and reverse direction. These six alignments were combined using M-COFFEE [76], and trimmed with trimAl v.1.3 [77] using a consistency cut-off of 0.16667 and a gap threshold of 0.1). Phylogenetic trees were built using a maximum likelihood approach as implemented in PhyML v3.0 [78] using the best fitting model among seven candidates (JTT, LG, WAG, Blosum62, MtREV, VT and Dayhoff). The two models best fitting the data were determined based on likelihoods of an initial Neighbor Joining tree topology and using the Akaike Information Criterion (AIC). We used four rate categories and inferred fraction of invariant positions and rate parameters from the data. All alignments and trees are available for browsing or download at PhylomeDB with the PhylomeID 787 [79].

**Table S13. List of species used in the phylome reconstruction.**

| **Taxa ID** | **Species name** | **Reference** |
| --- | --- | --- |
| 29158 | *Mytilus galloprovincialis* | this study |
| 356393 | *Limnoperna fortunei* | [67] |
| 310899 | *Modiolus philippinarum* | [48] |
| 220390 | *Bathymodiolus platifrons* | [48] |
| 6565 | *Crassostrea virginica* | NCBI* |
| 6573 | *Mizuhopecten yessoensis* | [51] |
| 50426 | *Pinctada fucata* | [50] |
| 6526 | *Biomphalaria glabrata* | [61] |
| 225164 | *Lottia gigantea* | [41] |
| 6500 | *Aplysia californica* | NCBI** |
| 37653 | *Octopus bimaculoides* | [62] |
| 115415 | *Phoronis australis* | [80] |
| 416868 | *Notospermus geniculatus* | [80] |
| 7574 | *Lingula anatina* | [81] |
| 283909 | *Capitella teleta* | [41] |
| 7227 | *Drosophila melanogaster* | Ensembl Metazoa release 25 [82] |
| 9606 | *Homo sapiens* | Ensembl release 77 [83] |

*<https://www.ncbi.nlm.nih.gov/assembly/GCF_002022765.2> **https://www.ncbi.nlm.nih.gov/assembly/683478

## 5.2. Prediction of gene duplications, and orthology/paralogy relationships

Orthology and paralogy relationships were predicted on the basis of the phylogenetic evidence collected from the mussel phylome. We used ETE v2 [84] to infer duplication and speciation relationships using a species overlap approach and a species overlap score of 0. The relative age of the inferred duplications was estimated using a phylogenetic approach that uses the information on which species diverged prior and after the duplication node. We performed Gene Ontology (GO) terms enrichment analysis with FatiGO [85] by comparing annotations of the proteins involved in a duplication at a given age against all the others encoded in the mussel genome. All orthology and paralogy relationships are available through PhylomeDB [79]. HMMER v3.1b2 [86] was used to find domains that contain homology with viral and transposable elements (based on Pfam-A.hmm domains collection). 290 proteins with such elements have been identified based on alignments with e-value < 0.01.

## 5.3. Species tree reconstruction

We selected 177 genes that had one-to-one orthologs in the 17 species, and after filtering out species specific duplications (in such cases, one of the paralogs was selected randomly), considered and concatenated their trimmed alignments, as reconstructed in the phylome. The final alignment contained 124,595 amino acid positions. We constructed a species tree using three different approaches: concatenation, gene-tree parsimony and a coalescent-based method.

First, from the concatenated aligment we reconstructed the maximum likelihood tree with PhyML v3.0 [78] using LG [78] as the model of aminoacid replacement, with four rate categories and fraction of invariant sites estimated from the alignment (**Fig. S17**). Clade support was computed using approximate Likelihood Ratio Tests and 100 bootstrap alignment replicates.

Second, we inferred a supertree (**Fig. S18**) by combining all gene trees in the phylome (54,976 trees) using a Gene Tree Parsimony approach as implemented in the dup-tree algorithm [87]. This procedure finds the species tree topology which implies the minimum number of total duplications in collection of gene family trees (i.e., the phylomes).

Finally, we obtained an estimate of the species tree with ASTRAL-III version 5.6.1 [88,89] (**Fig. S19**) based on 177 individual maximum-likelihod (ML) gene trees previously obtained with RaxML-NG [90] (<https://github.com/amkozlov/raxml-ng>), and including quartet-support. Individual ML gene trees were estimated under the best-fit model of amino acid replacement selected by modeltest-ng [91]. One hundred heuristic searches were performed in RaxML-NG using the SPR algorithm, starting from 50 random addition parsimony trees and 50 random trees.


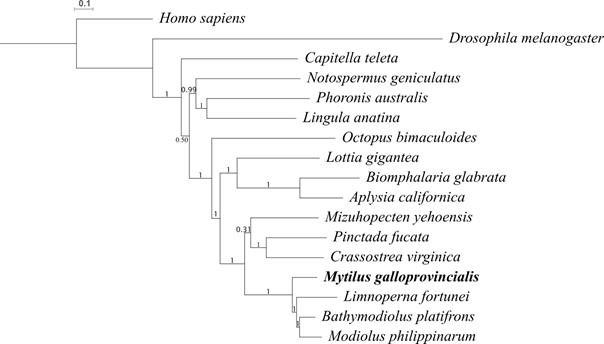


**Fig. S17. Species tree obtained from the concatenation of 177 widespread single-gene families**. Species names in bold indicate genomes that have been sequenced in this study. Numbers below each branch represent the statistical support of the node (aLRT); while numbers above each branch represent branch length. The topology agrees with the established phylogeny of mollusks, with Bivalvia as sister branch to Gastropoda; both forming a clade sister to Cephalopoda. Mollusca appears as sister branch of a clade containing Phoronida (*Phoronis*), Nemertea (*Notospermus*) and Brachiopoda (*Lingula*) with low support (0.503). Sisterhood of Brachiopoda + Phoronida is highly supported, though.


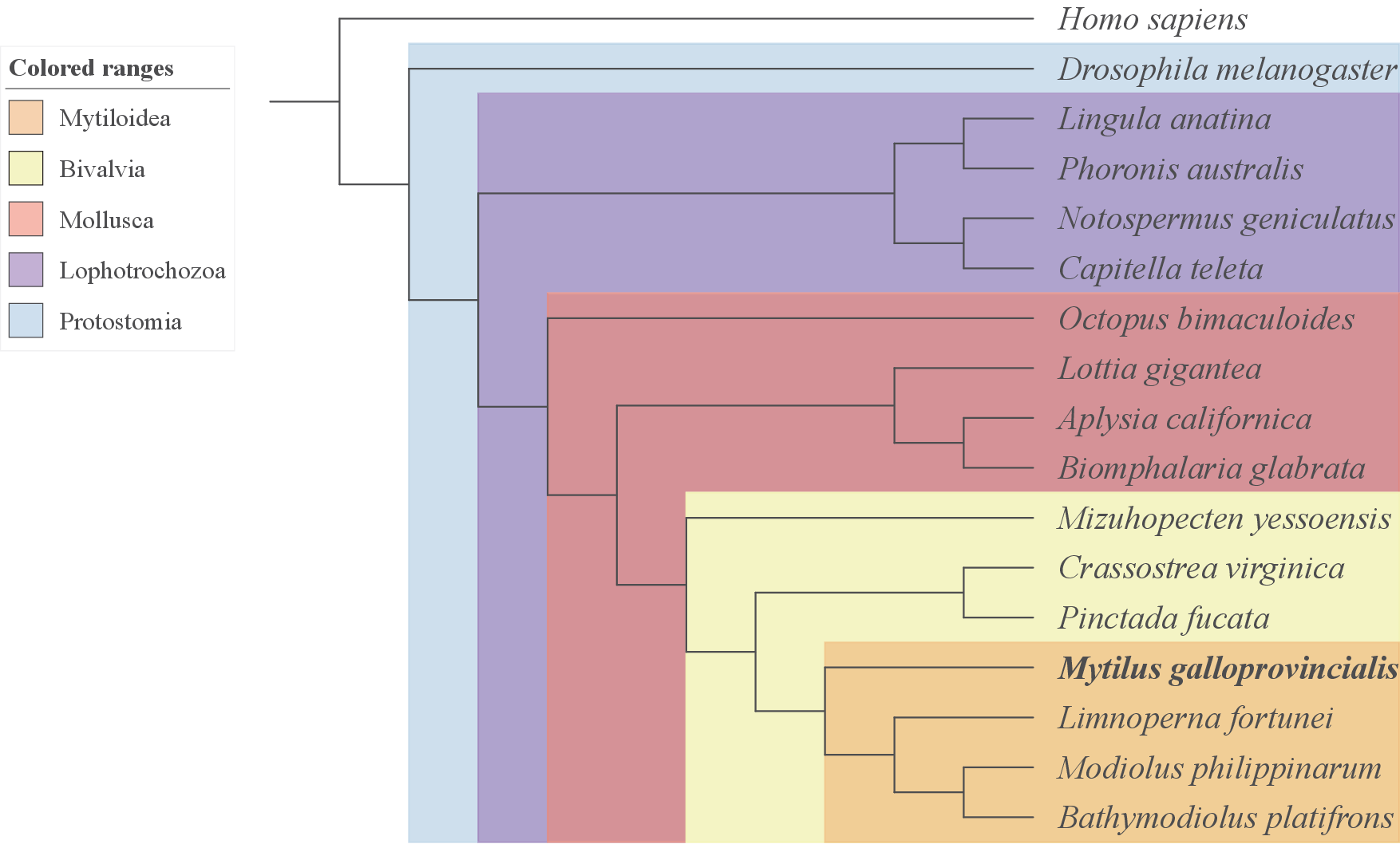


**Fig. S18. Species tree obtained with the gene tree parsimony approach**

**
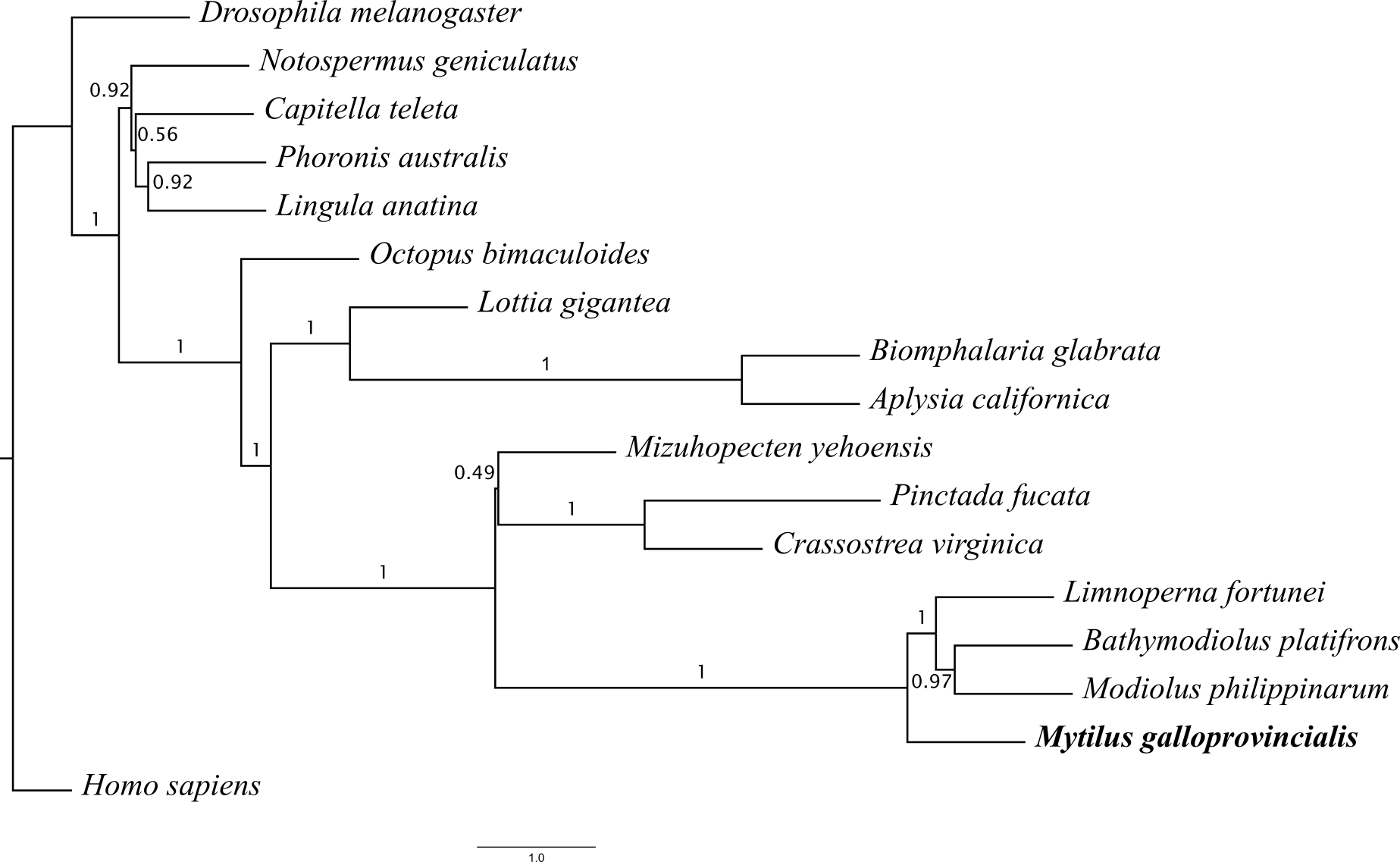
**

**Fig. S19. Species tree estimated with ASTRAL-III.** Numbers above branches represent quartet-support.

## 5.4. Lineage-specific gene family expansion events

Mytilida, represented here by *Mytilus*, *Limnoperna, Modiolus* and *Bathymodiolus*, displays an extremely high duplication rate, with 23,815 *Mytilus* genes (39.50% of the total) inferred to have been duplicated in the common ancestor of all Mytilida. Among the *Mytilus-*specific duplications, including large expansions, 35,027 proteins (58.05% of the total) have an in-paralog (i.e., resulting from a duplication that occurred in the *Mytilus* lineage since its split from the rest of Mytilida). These in-paralogs could be assigned to 10,450 *Mytilus*-specific gene expansions that contain 40,711 genes (67.47% of the total) of which 7,538 appear in more than one cluster. Most of the clusters (8,303, i.e., 79.45%) have a moderate size (2-5 in-paralogs, **Fig. S20**). Interestingly, a small fraction of clusters (286, 2.7%) contain very-large expansions (>20 in-paralogs, overall accounting for 9,643 genes, 15.98% of the genome; **Fig. S21**).


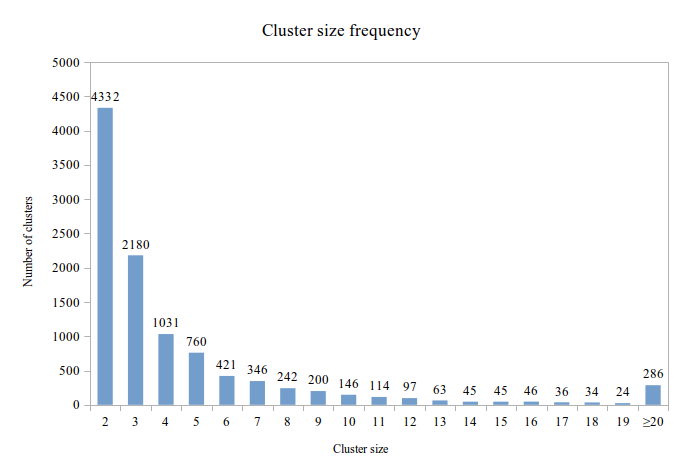


**Fig. S20. Distribution of size of in-paralog groups resulting from *Mytilus*-specific duplications**.


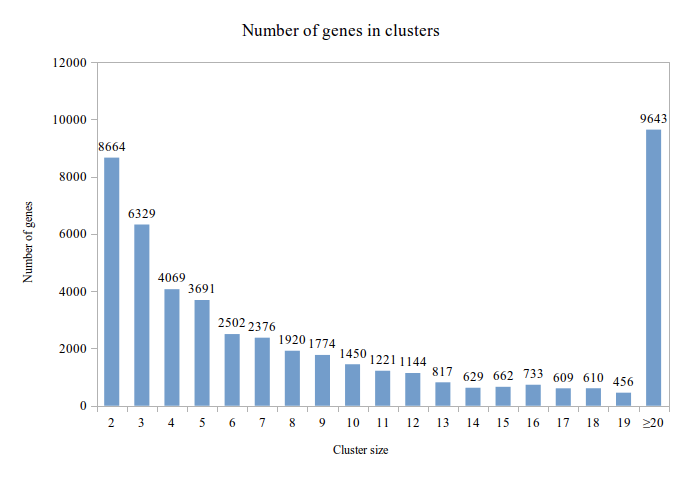


**Fig. S21. Number of genes for each cluster size groups resulting from *Mytilus*-specific duplications**.

Genes specifically duplicated in *Mytilus* are significantly enriched in various biological functions, including protein and carbohydrate binding, innate immune response, signal transduction and DNA/RNA processing (**Additional file 2:** **Table S14**). These results strongly suggest that gene expansion played a capital role in the genome evolution of Mytilida. All sampled members of this clade present a very high number of genes compared to most sequenced metazoan species, and the duplication rates of *Mytilus* suggests that this extreme dynamic nature is shared by other mytilid species (**Table S15**).

**Table S15**: Computed duplication ratios (average inferred duplications per gene lineage at that branch), after removing expansions (events involving more than five duplications in the same gene at the same branch).

| Evolutionary period | Duplication density (duplications/gene) |
| --- | --- |
| *Mytilus* specific | 1.40 |
| Mytilida | 1.28 |
| Mytilida + Pectinida | 0.05 |
| Bivalvia | 1.00 |
| Bivalvia + Gastropoda | 0.19 |
| Mollusca | 0.09 |
| Lophotrochozoa, excluding Annelida | 1.63 |
| Lophotrochozoa | 0.67 |
| Protostomia | 0.30 |
| Bilateria | 2.59 |

**Additional file 2:** **Supplementary Tables 16-33**: Enrichment of Gene Ontology terms in lineage-specific expanded gene families, with respect to the species tree displayed in **Figure 1** (see main text). Both “raw” data (corresponding to yellow circles in **Figure 1**) and data processed by removing large expansions consisting on more than 20 paralogs appearing in a single node (corresponding to yellow circles in **Figure 1**) are presented for the following taxonomical units:

1. *Mytilus* (**Table S16** and **S25**)
2. Mytilida (**Table S17**and **S26**)
3. Bivalvia (**Table S18** and **S27**)
4. Bivalvia + Gastropoda (**Table S19** and **S28**)
5. Mollusca (**Table S20** and **S29**)
6. Lophotrochozoa (excluding Annelida) (**Table S21** and **S30**)
7. Lophotrochozoa (**Table S22**and **S31**)
8. Protostomia (**Table S23** and **S32**)
9. Bilateria (**Table S24** and **S33**)

# Data Note 6 - Whole genome resequencing and heterozygosity rate estimates

## Resequencing of 14 additional mussels and re-analysis of *Pura*

Fifteen additional adult *M. galloprovincialis* specimens (**Table S1**), were collected from a commercial raft in the Ría de Vigo (Galicia, Spain, 42°15'54.8"N 8°43'42.5"W) (GAL) (**Fig. S1**) and from a different mussel population from a farming site off-shore the Goro lagoon (Northern Adriatic Sea, Italy, 44°43'22/44°43'12"N 12°19'54"/12°18'50"E) (ITA) [92] (**Fig. S22**). Three females (F) and three males (M) from each sampling site were randomly chosen to be sequenced. Sex was determined via microscopic examination of the gonadal tissue. In addition, one of the males was used as a technical replicate and two more males (one ripe, GALM6, and one post-spawned, GALM11) were also sequenced to discard genomic bias due to the gonadal state (see **Data Note 23**). Gonadal state was determined and confirmed by histology in all individuals. All individuals looked healthy at histological examination, with no evidence of morphological aberrations and presence of neoplastic tissues.


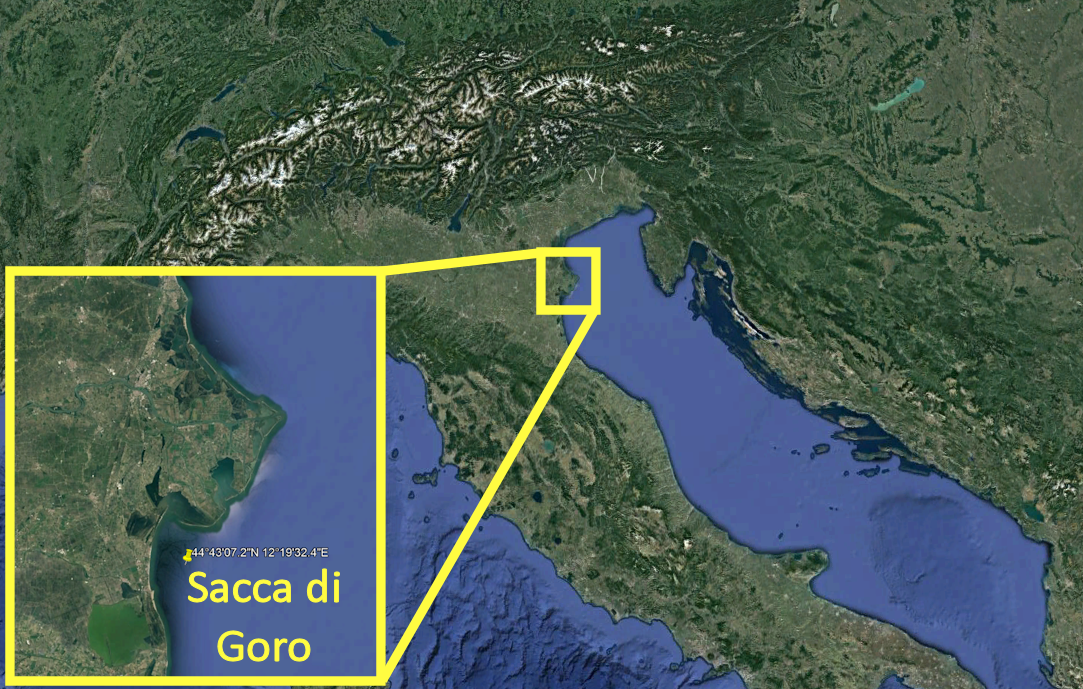


**Fig. S22. Sampling location of the six *M. galloprovincialis* Italian specimens sequenced in the present study.** Geographical coordinates of the sampling site are: (44°43'22/44°43'12"N 12°19'54"/12°18'50"E, Sacca di Goro, Italy). The sampling location for the eight Galician *M. galloprovincialis* individuals in shown in **Fig. S1**.

The selection of Galicia and the Adriatic Sea as sampling sites was motivated in part by practical reasons (i.e., accessibility to farming sites) and in part because two major different *M. galloprovincialis* lineages have been described by the use of nuclear molecular markers [93]. Gene flow between these two separate *M. galloprovincialis* clusters is partly prevented by both natural (geographical) and genetic barriers [94]. The first one, i.e., an “Atlantic clade” with higher rates of genetic introgression from *M. edulis*, is here exemplified by the population sampled at Ría de Vigo. On the other hand, the “Mediterranean clade”, a “pure” *M. galloprovincialis* lineage less subject to introgresion, is here exemplified by the population sampled at Sacca di Goro.

Mantle tissue was used to obtain genomic DNA following extraction in a Maxwell 16 LEV robot (Promega, Madison, WI, USA). Quantity and quality of DNA were assessed by a ND1000 Nanodrop (NanoDrop Technologies, Inc., DE, USA) and gel electrophoresis, to ensure the sample suitability for NGS-compatible library preparation. Genomic DNA was stored in TE buffer until the shipment to Admera Health (USA), where DNA libraries were prepared with the Kapa DNA Library preparation kit (Kapa Biosystems, Wilmington, MA) and sequenced on an Illumina HiSeq 2500 platform with a PE 2x150 nt strategy, aiming at achieving a 35X genome sequencing coverage. Raw sequencing data from the previously published paper by Murgarella *et al.* [17], targeting a single female Galician mussel named *Pura*, was also used (genomic DNA was extracted from the mantle tissue). Details about the sequencing outputs of the resequenced mussels are reported in **Additional file 2:** **Table S34.**

## Initial estimate of heterozygosity rates by *k-mer* analysis

The heterozygosity rates of the 17 mussel genomes, as well as from other available molluscan genomes were initially estimated by *k-mer* frequency analysis using GenomeScope [95]. For *M. galloprovincialis*, PE Illumina sequencing data obtained from the mantle tissue were analyzed, with the exception of the samples GALM1 and ITAM1 where, due to the aberrant profiles obtained in this tissue (see main text and **Data Note 23**), PE sequence data from gills were used instead. For the *Lola* genome, the estimate was performed based on PE sequence data from gills. For the *Pura* genome, data reported from Murgarella *et al.* was retrieved from the SRA database [17]. In all cases, *k-mer* frequencies were estimated with Jellyfish v.2.2.6 [4], based on a *k-mer* size of 17 nucleotides.

*K-mer* plots from other molluscan genomes were either kindly provided by the authors, retrieved from the supplementary material linked to the original publications, or calculated *de novo* using sequencing data obtained from the NCBI SRA database. In detail, the species selected were *Mytilus coruscus* [46], *Limnoperna fortunei* [47], *Bathyodiolus platifrons* [48], *Modiolus philippinarum* [48], *Crassostrea gigas* [42], *Pinctada fucata* [50], *Azumapecten farreri* [96], *Mizuhopecten yessoensis* [96], *Pecten maximus* [52], *Ruditapes philippinarum* [53], *Venustaconcha ellipsiformis* [59] and *Dreissena rostriformis* [58] as representative species for bivalves, and *Biomphalaria glabrata* [61] and *Octopus bimaculoides* [62] as representative species for gastropods and cephalopods, respectively.

The estimated heterozygosity rates of the 16 sequenced *M. galloprovincialis* genomes were quite uniform and similar to *Lola*, ranging from 2.76% (ITAF1) to 3.28% (ITAM3), averaging 3.07% (**Fig. S23**). The only exception was GALF1, a female specimen sampled in Galicia, which displayed a heterozygosity rate of 4.45%. Detailed *k-mer* profiles for the 16 mussel genomes are reported in **Figures S24-S27**.

All the *M. galloprovincialis* genomes showed a level of heterozygosity slightly higher than the reference genome of the invasive golden mussel *L. fortunei* (2.71%) [47] and significantly higher than its congeneric species *M. coruscus*, standing at 1.64% [46]. The two other available Mytilidae genomes showed significantly lower heterozygosity rates, as previously reported by Sun *et al.* [48]. Consistently with previous reports, bivalve mollusk genomes are highly heterozygous, with values ranging from 1.5 to 2.5%, with the exceptions of *M. yessoensis*, whose genome assembly was obtained from a highly inbred individual [51], and the freshwater mussel *Venustaconcha ellipsiformis*, which experienced severe genetic bottlenecks during its evolution due to glaciation events [59]. The level of heterozygosity in the blood fluke *B. glabrata* was approximately the half of the average level observed in *M. galloprovincialis* [61], whereas the octopus genome, as reported by Albertin *et al.*, shows a high degree of homozygosity [62].


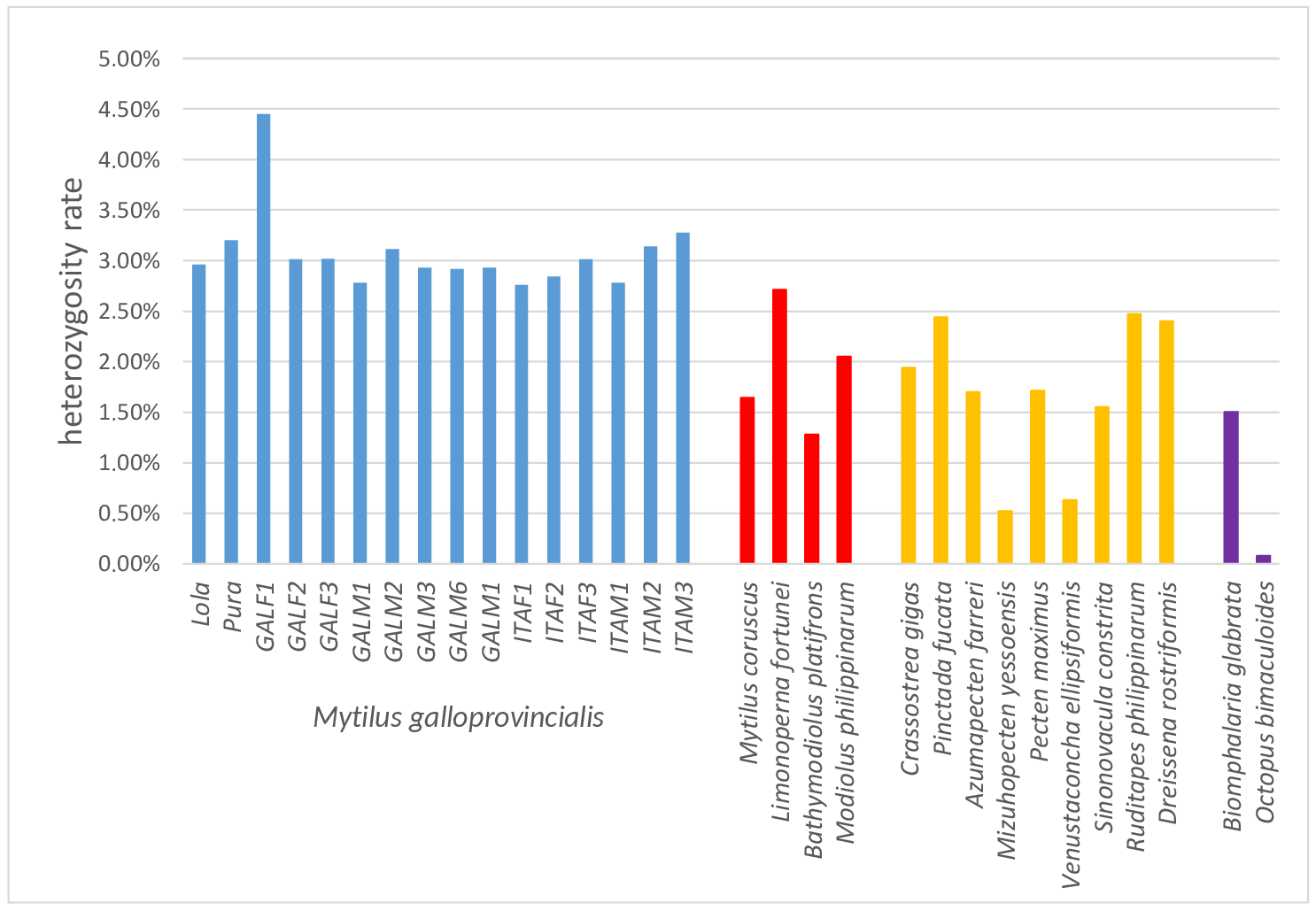


**Fig. S23**. **Comparative overview of heterozygosity levels computed based on *k-mer* frequency analysis in molluscan genomes.** The 16 *M. galloprovincialis* genomes subject of this study are indicated in light blue (for details, see **Figures S24-S27**), other Mytilida in red, non-mytilid bivalves in orange and non-bivalve mollusks in violet.


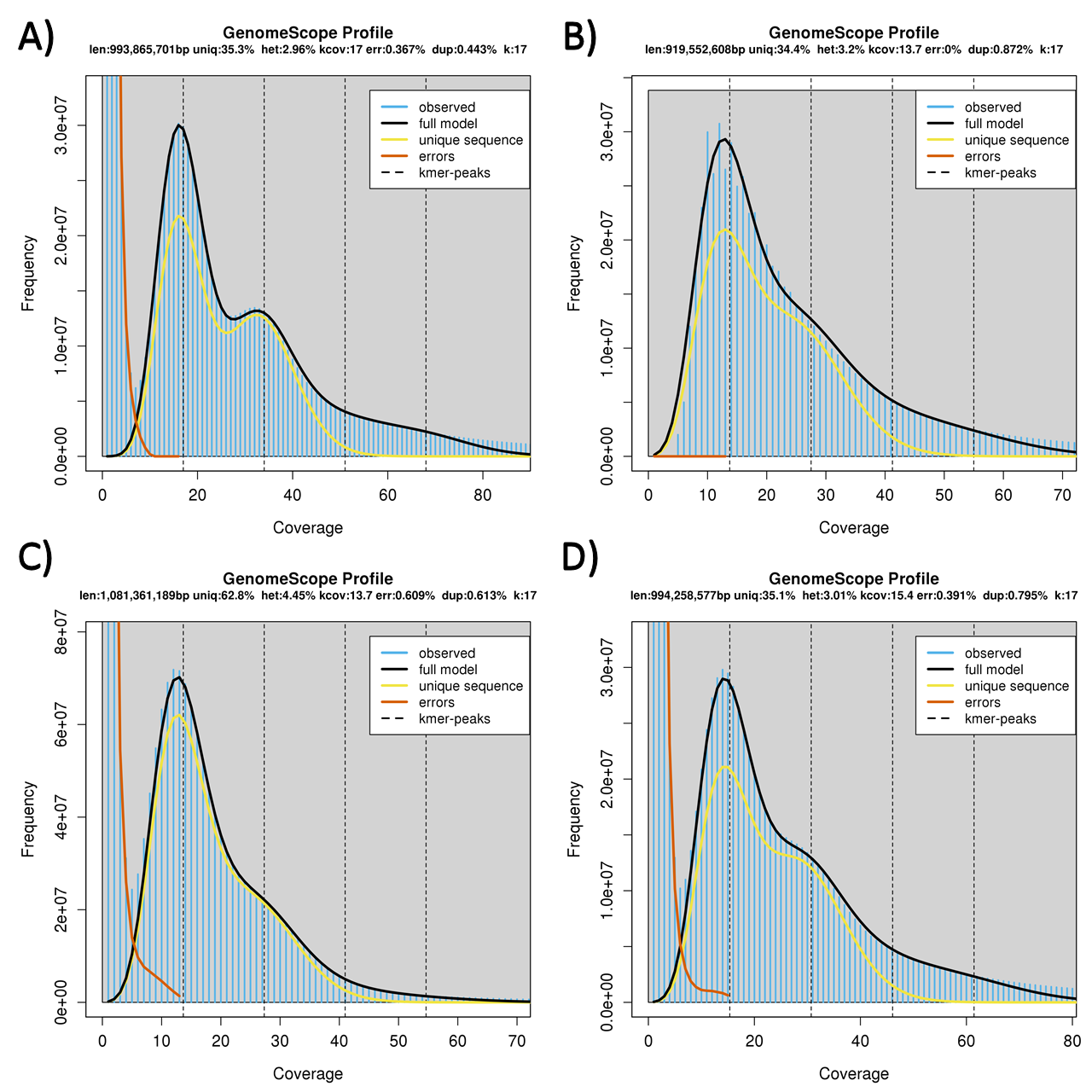


**Fig. S24**. **Heterozygosity estimates from *Lola*, *Pura*, GALF1 and GALF2 genomes.** Panel A: heterozygosity estimate from *M. galloprovincialis Lola* genome (Illumina PE library obtained from gills) obtained with GenomeScope. Panel B: heterozygosty estimate from *M. galloprovincialis Pura* genome (Illumina PE library obtained from mantle) obtained with GenomeScope. In this case, the “error peak” was manually removed from the *k-mer* table to allow the reaching of run convergence. Panel C: heterozygosity estimate from *M. galloprovincialis* GALF1 genome (Illumina PE library obtained from mantle) obtained with GenomeScope. Panel D: heterozygosty estimate from *M. galloprovincialis* GALF2 genome (Illumina PE library obtained from mantle) obtained with GenomeScope.


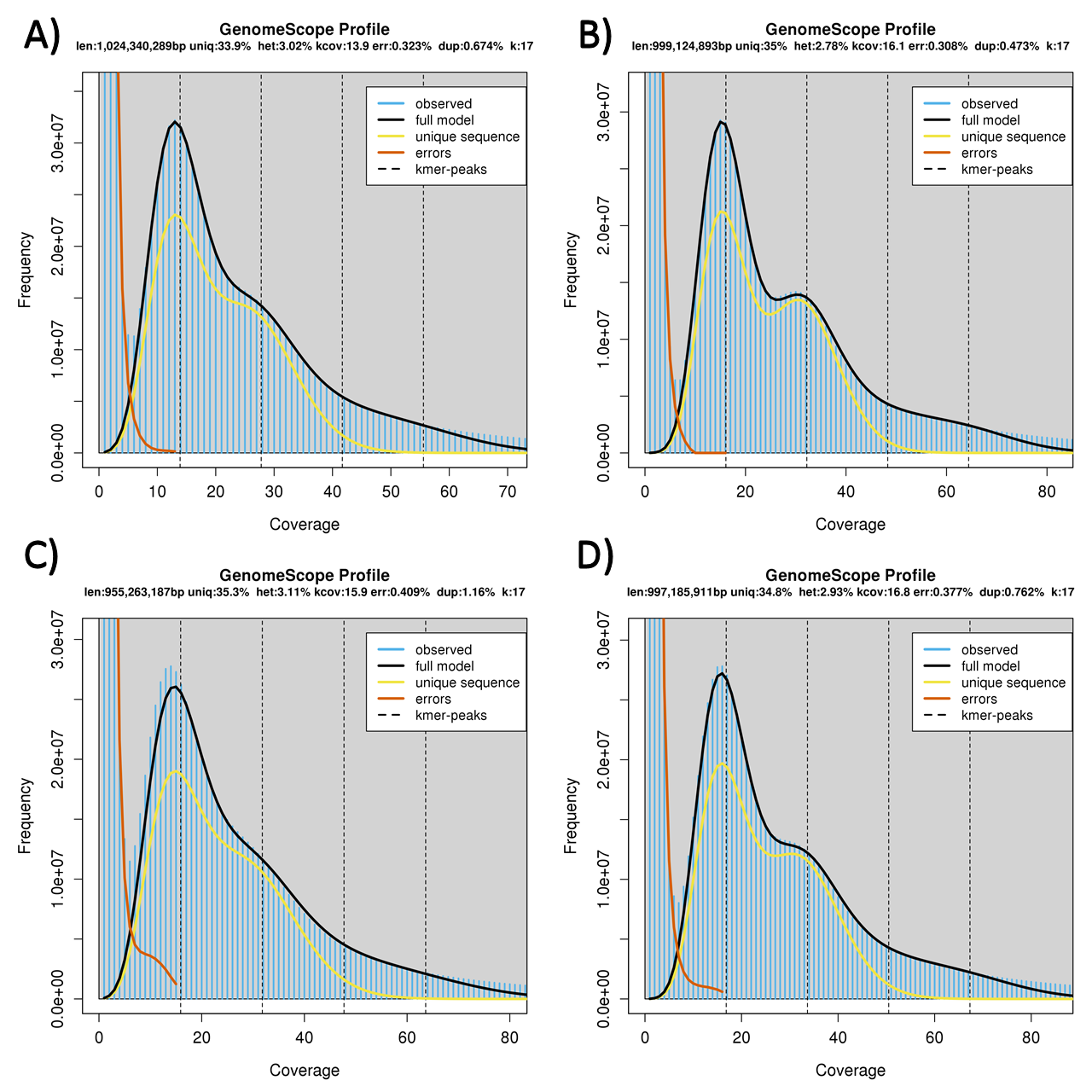


**Fig. S25**. **Heterozygosity estimates from GALF3, GALM1, GALM2 and GALM3 genomes.** Panel A: heterozygosity estimate from *M. galloprovincialis* GALF3 genome (Illumina PE library obtained from mantle) obtained with GenomeScope. Panel B: heterozygosty estimate from *M. galloprovincialis* GALM1 genome (Illumina PE library obtained from gills) obtained with GenomeScope. Panel C: heterozygosty estimate from *M. galloprovincialis* GALM2 genome (Illumina PE library obtained from mantle) obtained with GenomeScope. Panel D: heterozygosty estimate from *M. galloprovincialis* GALM3 genome (Illumina PE library obtained from mantle) obtained with GenomeScope.


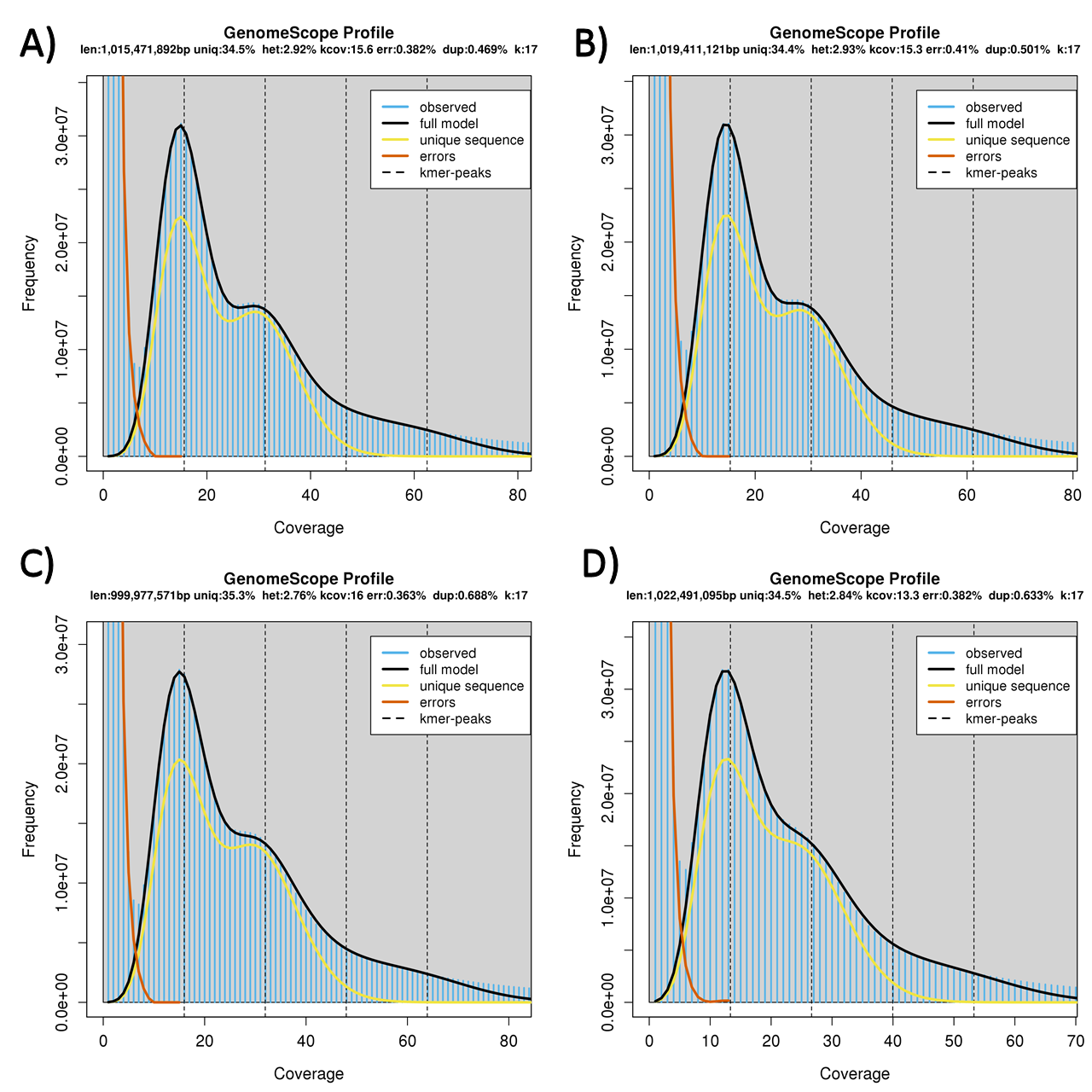


**Fig. S26**. **Heterozygosity estimates from GALM6, GALM11, ITAF1 and ITAF2 genomes.** Panel A: heterozygosity estimate from *M. galloprovincialis* GALM6 genome (Illumina PE library obtained from mantle) obtained with GenomeScope. Panel B: heterozygosty estimate from *M. galloprovincialis* GALM11 genome (Illumina PE library obtained from mantle) obtained with GenomeScope. Panel C: heterozygosty estimate from *M. galloprovincialis* ITAF1 genome (Illumina PE library obtained from mantle) obtained with GenomeScope. Panel D: heterozygosty estimate from *M. galloprovincialis* ITAF2 genome (Illumina PE library obtained from mantle) obtained with GenomeScope.


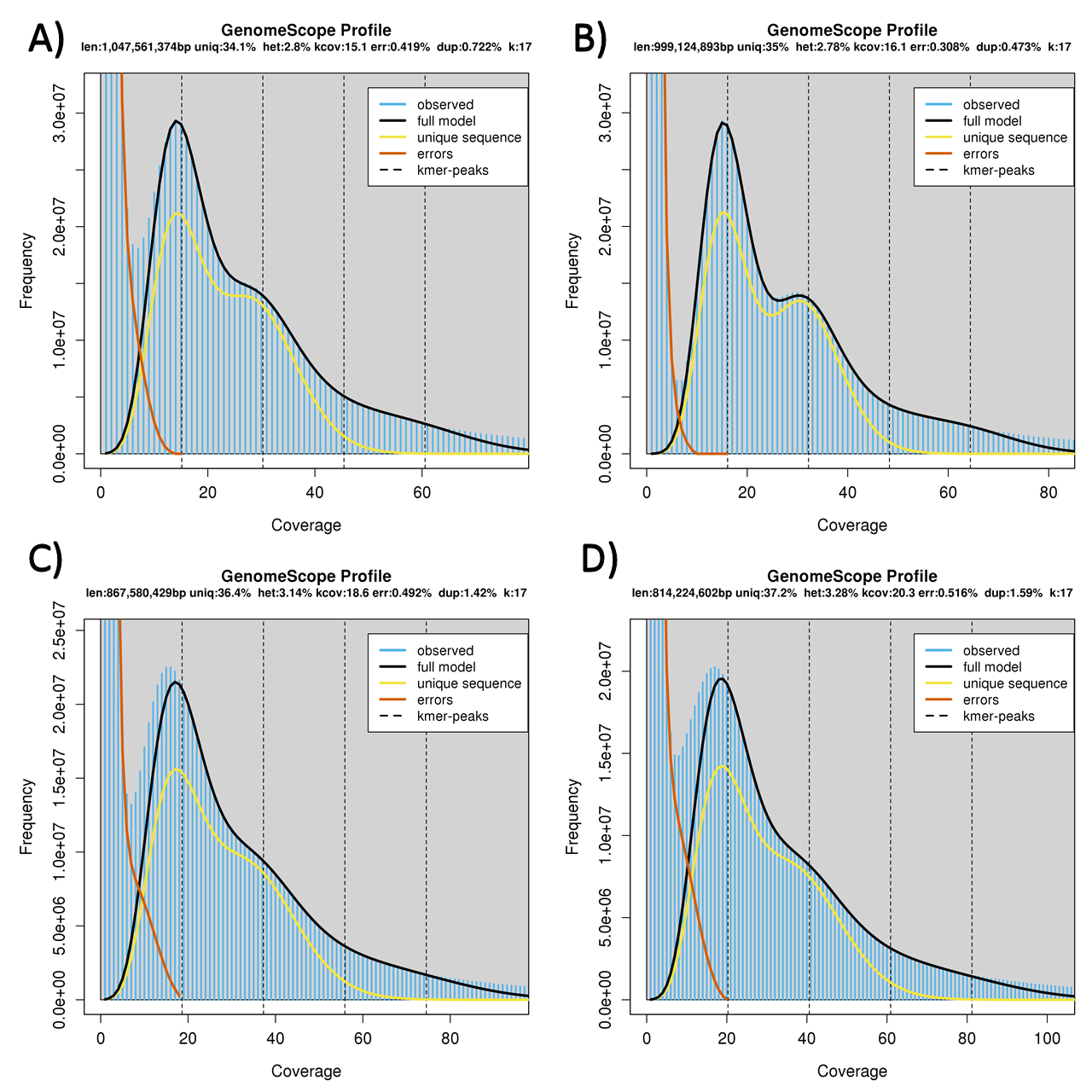


**Fig. S27**. **Heterozygosity estimates from ITAF3 and ITAM1, ITAM2 and ITAM3 genomes.** Panel A: heterozygosity estimate from *M. galloprovincialis* ITAF3 genome (Illumina PE library obtained from mantle) obtained with GenomeScope. Panel B: heterozygosty estimate from *M. galloprovincialis* ITAM1 genome (Illumina PE library obtained from gills) obtained with GenomeScope. Panel C: heterozygosty estimate from *M. galloprovincialis* ITAM2 genome (Illumina PE library obtained from mantle) obtained with GenomeScope. Panel D: heterozygosty estimate from *M. galloprovincialis* ITAM3 genome (Illumina PE library obtained from mantle) obtained with GenomeScope.

## Improved estimate of heterozygosity rates

The discovery of massive PAV in the mussel genome (see main text and **Data Note 8**) revealed that *k-mer* based approaches were unsuitable for providing a reliable estimate of the heterozygosity rates fot his particular genomic architecture. Indeed, *k-mers* derived from hemizygous genomic regions contribute to increasing the size of the “haploid peak” of coverage, thereby inflating the estimates of heterozygosity.

Therefore, we decided to use instead GATK 3.7 [19], adapting the “GATK Best Practices for Germline Short Variant Discovery [97,98] to a diploid organism with extreme levels of polymorphism and no available dbSNP for the species. For each individual sample and technical replicate (different tissues or sequencing platform), we aligned the PE reads to the reference genome (mg10) using BWA mem [99,100] (version 0.7.7), selecting the option –M. In order to verify that the differences in coverage did not bias the estimates between samples. We down-sampled the *Lola* alignment (coverage=82.92) producing a BAM file with ~33x coverage (the mean coverage of WGR samples and similar to *Pura*’s coverage), which was used for variant calling. The down-sampling was carried out using samtools view (version 1.2) [101] with options -hb -s 0.4. Duplicates in these alignments were marked using the MarkDuplicates tool from PICARD (version 1.60, <https://broadinstitute.github.io/picard/>). Then, we identified the callable sites per sample was obtained using the GATK’s CallableLoci tool, with options --minBaseQuality 10 --minMappingQuality 20, to be in concordance with the default value for these parameters in HaplotypeCaller. The variant calling was performed using the HaplotypeCaller but restricting it to the callable sites only and with options:

-dt NONE -rf BadCigar --never_trim_vcf_format_field -ploidy 2 --min_base_quality_score 10 --standard_min_confidence_threshold_for_calling 30 --emitRefConfidence GVCF and --GVCFGQBands

at Genotype Qualities 15, 20, 25, 30, 35, 40, 45, 50, 55, 60, 65, 70, 80 and 99. The resulting GVCF was used to call genotypes with the the GenotypeGVCF tool and option --never_trim_vcf_format_field. Note that we exclusively considered supported variants. That means that only those that were covered at least by 10 reads were considered and, if the sample was heterozygote for a particular site, the alternative allele needed to be supported by at least 2 reads.

After discarding all the unsupported and multi-allelic variants for each mussel, we estimated the heterozygosity rate as the total number heterozygous SNVs divided by the total number of callable sites. Finally, we identified the sites that were callable in all samples and replicates by intersecting the callable intervals with BEDOPS version 2.0.0a [102], selected the SNP variants falling inside these regions (using GATK’s SelectVariants) and calculated the heterozygosity rate again.

Using this method, we estimated a mean heterozygosity of 1.58% (s.d. = 0.16%) (**Fig. S28**). *Lola* shows a different heterozygosity rate (~1.2%) in all replicates. This likely due to the assembly of haploid stretches in this individual that are both callable and homozygous, depleted in variants, and that reduce the heterozygosity rate. A total of 312 Mb sequence was identified to be callable in all the resequencing samples and replicates, simultaneously. This corresponds with 22.12% of the estimated genome size and 24.5% of the assembly size (mg10). The heterozygosity for this fraction of the genome does not differ dramatically between the assembled individual (1.8%) and the other mussels (1.66%-2.05%) (with the exceptions of the GALM1 and ITAM1 mantle, that present skewed mapping profiles, see **Data Note 23**). This also supports the hypothesis that we have assembled haploid regions of *Lola* and yields a less biased estimate for the species. The average heterozygosity rate of the mussel genomes studied here is 1.73%, with a standard deviation of 0.24%.


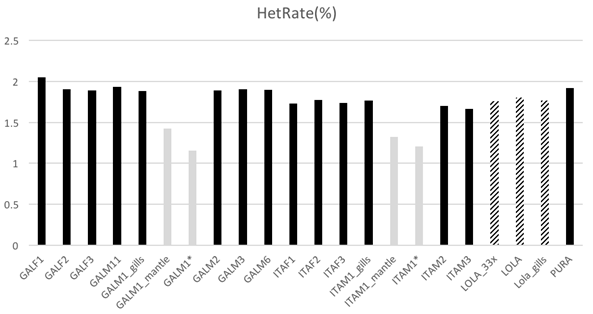


**Fig. S28: Improved heterozygosity rates estimate of the mussel genome.** Estimated for *Lola* and all resequenced individuals, as explained in section 6.3.*technical replicates of the GALM1 and ITAM1 mantle samples.

# 7. Data Note 7 – Assessment of genetic introgression from congeneric species

## 7.1. Overview of molecular markers used for the assessment of genetic introgression and methodology used

*M. galloprovincialis* is part of the *M. edulis* species complex, together with *M. edulis* and *M. trossulus*, but distinct from the congeneric species *M. californianus* and *M. coruscus*. The three species are potentially interfertile and, in spite of the presence of partially characterized barriers to gene flow, they create “hybrid zones” in certain geographic locations where the species are sympatric [103–105], with the most extensively studied being the north-Westerns coast of France, including the Bay of Biscay, Brittany and Normandy [106]. A number of studies have shown that genetic introgression is widespread in mussel populations across the globe [107], highlighting the need to consider the complex evolutionary history of mussel populations and secondary contacts, possibly also linked to aquaculture practices, in explaining the observation of outlier loci in the evaluation of population molecular markers. Due to the widespread genetic introgression within the *M. edulis* species complex, no single genetic marker can be considered as diagnostic for *M. galloprovincialis*, although a number of studies have previously identified some targets, usually analyzed with PCR approaches, that can be informative, especially when they are considered in combination.

Namely, among nuclear markers, the Glu-5’ fragment of the polyphenolic adhesive protein gene has been previously shown to be useful to discriminate between the three species in the *M. edulis* species complex due to length polymorphisms [108,109]. Similarly, the intron 1 of the *mac-1* actin gene displays length polymorphism which enables species discrimination [110–112], even though in this case, unlike Glu-5’, where just three amplicon sizes can be obtained, the patterns of band length are much more complex. A third nuclear locus which has been successfully used for species discrimination is EFbis, corresponding to 1/1.3 kb of genomic region which includes 3 exons (second, third and fourth) and 2 introns of the EF1A1 gene, which is also characterized by length polymorphisms [113,114]. Mitochondrial DNA is also frequently used for population genetics studies, usually limited to the female-type sequence, which is present in all individuals regardless of the sex. In particular mitochondrial molecular markers, including 16S mitochondrial rRNA and COI, have been previously used, together with nuclear markers, to track the evolutionary history of mussel populations [115–117].

Exploiting previously published data and validated haplotypes for *M. galloprovincialis*, *M. edulis* and *M. trossulus*, we inspected whether *Lola*, *Pura* and the 14 resequenced mussel genomes displayed sequences consistent with their identification as part of a “pure” *M. galloprovincialis* lineage, or whether any clear evidence of hybridization with congeneric species existed. The presence of each allele for the target loci was inspected by *in silico* PCR based on the alignment of forward and reverse primers with *de novo* assembled genomes, inferring the size of the amplicons that would be expected to be obtained by PCR. The absence of non-detected alleles and dubious situations (e.g. partially assembled genomic regions) were resolved by back-mapping raw reads to the most closely related allele available and *de novo* reassembly, performed recursively, until the assembly gaps were solved. Sequencing primers were retrieved from relevant publications (see details below) and, in case of difficulties in the retrieval of complete genomic regions due to gaps in *de novo* assemblies, a BLASTn strategy [118] was also implemented, using custom e-value thresholds set on a case-by-case basis.

## 7.2. Polyphenolic adhesive protein gene Glu-5’ fragment polymorphisms

First, the analysis of length polymorphisms of the Glu-5’ fragment of the polyphenolic adhesive protein gene (**Table S35**) revealed high genetic homogeneity across all genomes, which were clearly homozygous for the *M. galloprovincialis* allele, expected to produce an amplicon of 126 nucleotides. As a unique exception, GALF1 displayed two distinct alleles, with the second one matching the size previously reported for the *M. edulis* allele (180 nt), indication of either genetic introgression from *M. edulis* (frequently reported in mussel populations across the North Western coasts of Europe), or possibly identifying GALF1 as a F1 hybrid between *M. edulis* and *M. galloprovincialis*. This analysis excluded the possibility that *M. trossulus* alleles were present in any of the resequenced genomes.

**Table S35.** Results of *in silico* PCR for the Glu-5’ fragment of polyphenolic adhesive protein gene. All genomes, with the single exception of GALF1, resulted to be homozygous for the *M. galloprovincialis* allele (expected to produce a 126nt long amplicon).

| **Genome** | **Amplicon size (I)** | **Amplicon size (II)** | **Diagnosis** |
| --- | --- | --- | --- |
| *Lola* | 126 | / | *Mytilus galloprovincialis* |
| *Pura* | 126 | / | *Mytilus galloprovincialis* |
| GALF1 | 126 | 180 | *Mytilus galloprovincialis/Mytilus edulis* |
| GALF2 | 126 | / | *Mytilus galloprovincialis* |
| GALF3 | 126 | / | *Mytilus galloprovincialis* |
| GALM1 | 126 | / | *Mytilus galloprovincialis* |
| GALM2 | 126 | / | *Mytilus galloprovincialis* |
| GALM3 | 126 | / | *Mytilus galloprovincialis* |
| GALM6 | 126 | / | *Mytilus galloprovincialis* |
| GAML11 | 126 | / | *Mytilus galloprovincialis* |
| ITAF1 | 126 | / | *Mytilus galloprovincialis* |
| ITAF2 | 126 | / | *Mytilus galloprovincialis* |
| ITAF3 | 126 | / | *Mytilus galloprovincialis* |
| ITAM1 | 126 | / | *Mytilus galloprovincialis* |
| ITAM2 | 126 | / | *Mytilus galloprovincialis* |
| ITAM3 | 126 | / | *Mytilus galloprovincialis* |

## 7.3. EF1bis locus polymorphisms

We then moved to the analysis of the EF1bis locus. In this case, due to the longest size of the amplicon and the high sequence similarity of some allelic variants, we could only retrieve partial sequences from some genomes, which were however informative enough to guarantee a good alignment with the validated, reference sequences. The only exception was one of the two alleles found in GALF1, which was too short to be considered informative for phylogenetic inference. A number of genomes (*Pura*, GALF2, GALM1, GALM2, GALM6, GALM11, ITAM1, ITAM2, ITAM3) showed a single assembled allelic variant, indicating either homozygosity for the locus, or high similarity between the two variants, which did not enable their assembly as separate sequences. A total of 64 individual sequences were retrieved from the NCBI database, referring to *M. galloprovincialis* individuals from the Atlantic Ocean, *M. edulis* individuals from the Bay of Biscay and form the North Sea, as well as *M. trossulus* samples [119]. The GenBank accession numbers for the sequences analyzed are: EU684206-EU684212, EU684181-EU684205, EU684213-EU684228, EU684168-EU684180.

The nucleotide sequences were aligned with MUSCLE [73] and the resulting multiple sequence alignment file was used to construct a neighbor-joining (NJ) phylogenetic tree [120] with the CLC Genomics Workbench, using 100 bootstrap replicates. The tree is presented, for a convenient graphical representation, as a cladogram in **Fig. S29**. The NJ clearly depicts three main clades, corresponding to *M. edulis*, *M. galloprovincialis* and *M. trossulus* (represented at the root of the tree).


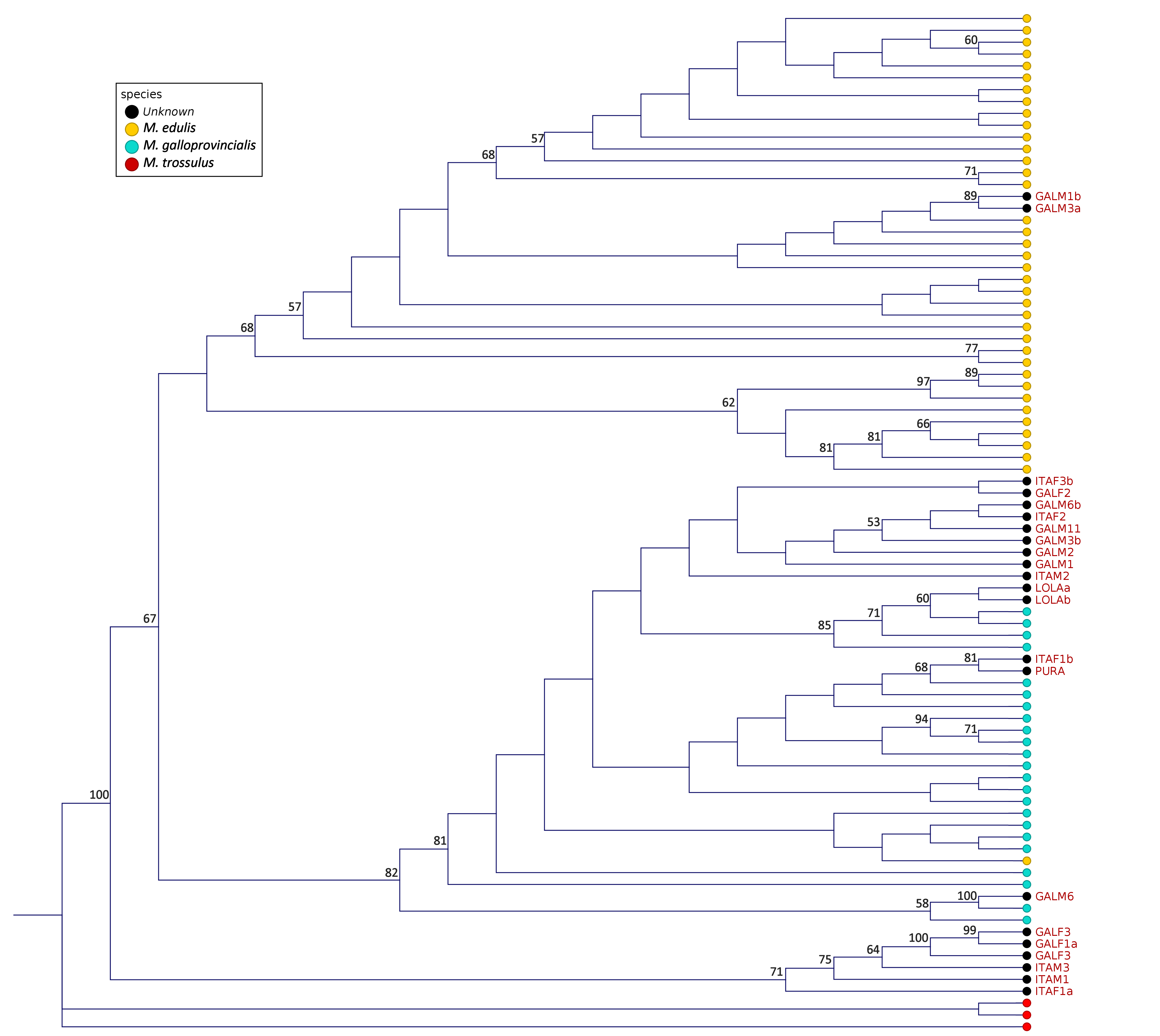


**Fig. S29**. **Cladogram depicting the EFbis gene tree of the 16 resequenced mussel genomes**, compared to previously published and validated sequences from *M. galloprovincialis* (light blue), *M. edulis* (light orange) and *M. trossulus* (red). The tree has been rooted on the *M. trossulus* clade. Significant bootstrap support values (i.e., values >= 50) are shown close to each node. Nodes without values attached are poorly supported (i.e., bootstrap values < 50).

However, while the *M. trossulus* (bootstrap = 100) and the *M. galloprovincialis* (bootstrap = 82) clades were quite well supported, the bootstrap support for the basal node of the *M. edulis* clade was quite low, and only higher-order nodes reached significant, albeit not very high, support. Overall, no sequence obtained from the resequenced individuals clustered within the *M. trossulus* clade, and only two sequences (GALM1b and GALM3a) were grouped with the alleles from *M. edulis*. However, both individuals also displayed a second allelic variant which clustered within the main *M. galloprovincialis* clade, indicating heterozygosity and probably genetic introgression of the *M. edulis*, which is supported by their homozygosity for the *M. galloprovincialis* allele at the Glu-5’ locus, as previously demonstrated (**Table S35**). Most of the sequences showed a remarkable similarity, being clustered close to each other within the clade comprising all the other available sequences of *M. galloprovincialis* specimens sampled in the Atlantic Ocean. However, a few variants (GALF1a, GALF3a, GALF3b, ITAF1a, ITAM1 and ITAM3) grouped in a distinct clade (bootstrap support = 71), which did not show significant similarity with any of the sequences previously reported [119], possibly identifying a second clade of *M. galloprovincialis* haplotypes most commonly found in the Mediterranean Sea (all the validated *M. galloprovincialis* sequence included in the analysis were obtained from Atlantic populations).

## 7.4. Mitochondrial DNA molecular markers

Finally, keeping in mind the unusual mode of doubly uniparental inheritance of mitochondrial (mt) DNA typical of mussels [121,122], we investigated the sequences of 16S rRNA from the female-type mitochondrial genome (found in all individuals, regardless of the sex), discarding information related to male-type mitochondrial DNA (see **Fig. S30** for the organization of the F-type mitochondrial genome in *Lola*). In detail, based on the results of previous studies [115], we analyzed the sequence of 16S mitochondrial rRNA, which is expected to enable a more efficient discrimination between *M. edulis* and *M. galloprovincialis* haplotypes compared to COI, which gives rise to a large heterogeneous clade of mixed alleles from both *M. galloprovincialis* and *M. edulis*. However, based on literature data, both markers allow an efficient discrimination with the more divergent mitochondrial haplotypes from *M. trossulus*.

**
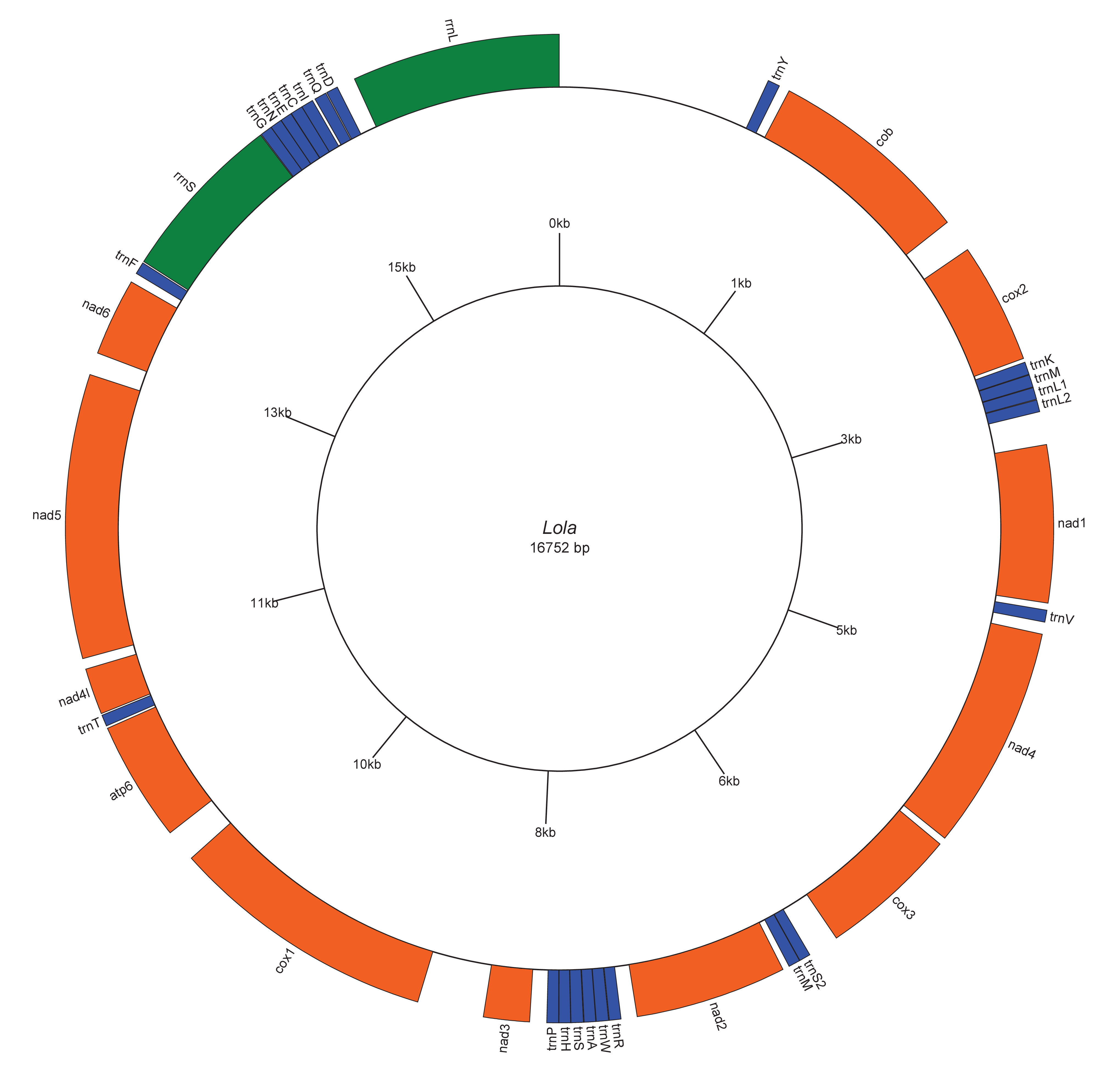
Fig. S30**. **Circularized *Lola* F-type mitochondrial DNA, displaying annotated tRNA genes (blue), proten-coding genes (red) and rRNA genes (green).**

The resulting mt DNA sequences were aligned with those reported in a previous study from Gerard *et al.* [115] using MUSCLE [73], and a NJ tree was constructed with the CLC Genomics Workbench using 100 bootstrap replicates. In addition, both 16S and COI sequences were subjected to a BLASTn analysis against the nr/nt database to check for potential perfect matches with previously published mitochondrial haplotypes of *Mytilus* spp. The combination of these results (i.e., identity with previously taxonomically assigned entries, shown in **Table S36**, validated hits and tree clustering of 16S rRNA sequences, shown in **Fig. S31**) was used to assess the likelihood that each of the resequenced mussel genomes pertained to a “pure” mussel lineage or was somehow subjected to introgression from the congeneric species *M. edulis* and *M. trossulus*. In the interpretation of the results, it needs to be considered that the relatively short size of the multiple sequence alignment (388 nucleotides) did not allow a complete phylogenetic sorting of all sequences, leading to the generation of many poorly supported clusters (**Fig. S31**).

**Table S36**: Occurrences of perfect matches between the mitochondrial 16S rRNA and COI sequences identified in the resequenced mussel genomes and those deposited in public sequence databases. The inferred origin of the F-type mitochondrial DNA for each individual is reported.

| **Genome** | closest *Mytilus galloprovincialis* hit | | **diagnosis** |
| --- | --- | --- | --- |
|  | **16S rRNA** | **COI** |  |
| *Lola* | 100.00% | 99.84% | *M. galloprovincialis* |
| *Pura* | 97.59% | 100.00% | *M. galloprovincialis* |
| GALF1 | 100.00% | 99.22% | *M. galloprovincialis* |
| GALF2 | 100.00% | 99.53% | *M. galloprovincialis* |
| GALF3 | 99.76% | 99.53% | probable *M. galloprovincialis* |
| GALM1 | 99.52% | 98.91% | probable *M. edulis* |
| GALM2 | 99.76% | 98.91% | *M. edulis* |
| GALM3 | 99.37% | 99.84% | probable *M. galloprovincialis* |
| GALM6 | 99.71% | 99.84% | probable *M. galloprovincialis* |
| GALM11 | 100.00% | 100.00% | *M. galloprovincialis* |
| ITAF1 | 99.76% | 99.22% | *M. edulis* |
| ITAF2 | 100.00% | 99.84% | *M. galloprovincialis* |
| ITAF3 | 100.00% | 100.00% | *M. galloprovincialis* |
| ITAM1 | 100.00% | 99.84% | *M. galloprovincialis* |
| ITAM2 | 100.00% | 100.00% | *M. galloprovincialis* |
| ITAM3 | 100.00% | 100.00% | *M. galloprovincialis* |

Overall, although not all the sequences identified showed perfect matches with previously characterized mitochondrial sequences (**Table S36**), 13 out of 16 genomes (including *Lola*) were characterized by mitochondrial markers consistent with a *M. galloprovinc*ialis origin. Two of the genomes (ITAF1 and GALM2) possessed a haplotype identical to some of those previously described for *M. edulis*. A third one (GALM1) also clustered within the *M. edulis* 16S rRNA clade (**Fig. S31**). However, it needs to be pointed out that, while the external *M. trossulus* clade was highly supported, the basal node of the *M. edulis* clade was not supported by a high bootstrap value and therefore the results reported here need to be carefully evaluated. Introgression of mtDNA haplotypes in mussel populations is thought to occur frequently in geographic regions of contact [124,125] and these three genomes have been characterized as being homozygous for the *M. galloprovincialis* allele at the Glu-5’ and EFbis nuclear loci (with the exception of GALM1, heterozygous for *M. galloprovincialis*/*M. edulis* at EFbis).

Consequently, the data we have collected support the identification of all the resequenced genomes as *M. galloprovincialis* specimens presenting a degree of genetic introgression of *M. edulis* alleles compatible with the ranges currently expected from natural populations from Galicia and Italy.


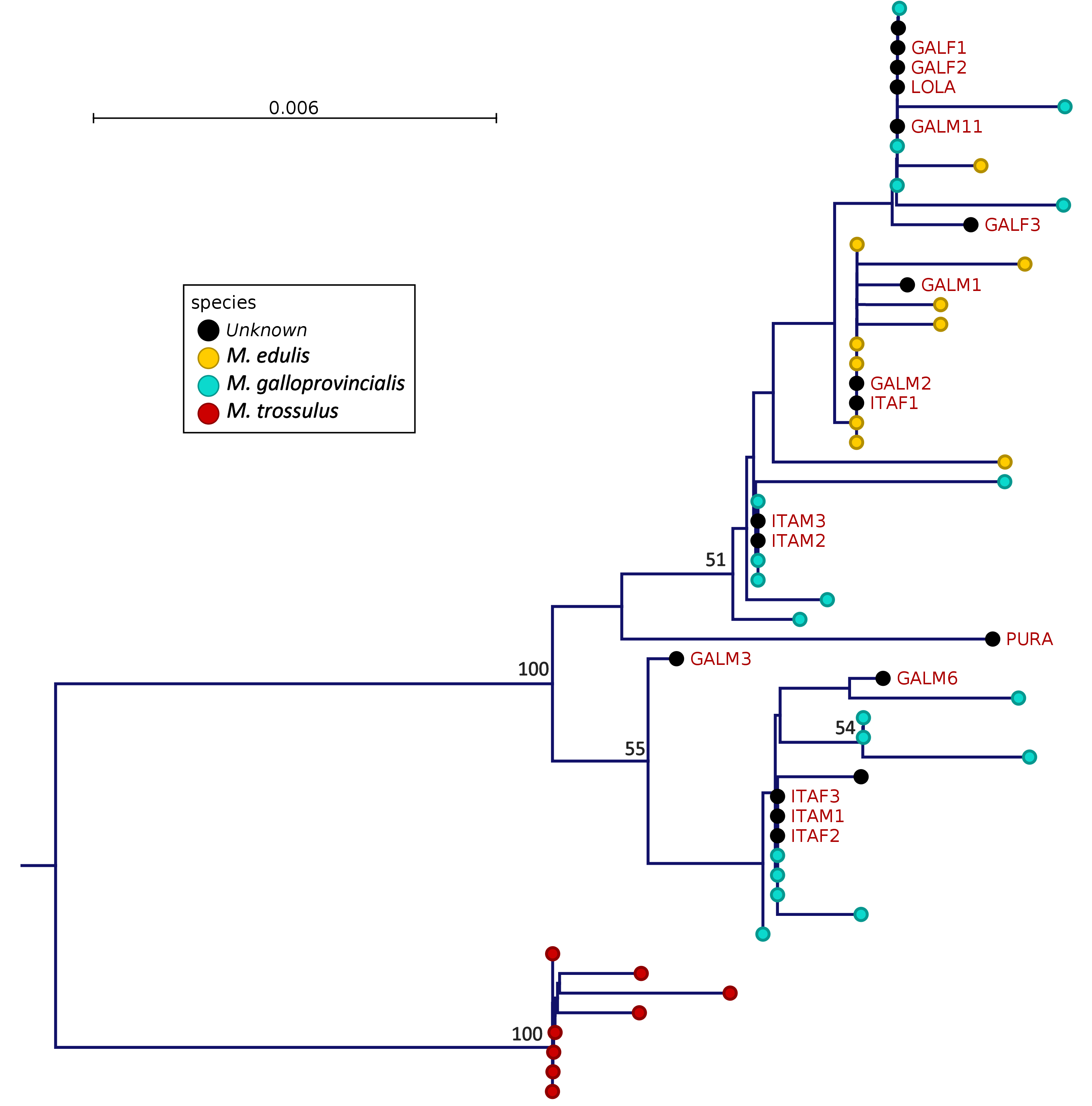


**Fig. S31**. **NJ tree depicting the mitochondrial 16S rRNA gene tree of the 16 resequenced mussel genomes, c**ompared to previously published and validated sequences from *M. galloprovincialis* (light blue). *M. edulis* (light orange) and *M. trossulus* (red). The tree has been rooted on the *M. trossulus* clade. Significant bootstrap support values (i.e., values >= 50) are shown close to each node. Nodes without values attached are poorly supported (i.e., bootstrap values < 50).

# Data Note 8 – Presence-Absence Variation (PAV)

## 8.1. Read mapping

We assessed the read coverage of each protein-coding gene annotated in the *Lola* mg10 reference assembly across resequenced genomes, including the previously sequenced *Pura* [17]. Details about the geographical origin and sex of individuals, as well as the tissue of origin are provided in **Table S1,** and details about the sequencing output from each individual are reported in **Table S34**.

All Illumina read datasets were independently mapped to the *Lola* reference assembly (mg10) using BWA mem (v0.7.15) [99]. Mappings were performed using the -M parameter, to mark shorter split hits as secondary. In this manner, alignments corresponding to chimeric reads were excluded. Following initial testing with a mapping quality (MQ) >= 60, the mappings were filtered by selecting alignments with MQ >=0 in order to tolerate multi-mappings (i.e., the alignment of reads with similar scores on different genomic positions). This choice was made to enable reads originated from closely related paralogous genes to be aligned on multiple locations, avoiding cases where a given genomic region would have been artificially assigned a coverage = 0.

Then, using BEDtools [33], mapping data was extracted from the bam files using the coordinates of the longest transcript per each of the non-redundant genes annotated. In this way, only reads mapping on exons were considered, and reads potentially mapping to repetitive elements located within introns, a possible source of background noise, were therefore discarded.

The coverage per gene was calculated as the average read coverage per base within the coding region only (corresponding to the longest annotated transcript per gene). Coverage estimates were subsequently normalized on the estimated haploid genome size (see details below), and each gene was categorized, for each individual resequenced genome and Illumina dataset, as follows:

(i) putatively absent, if the coverage, normalized on the haploid peak of coverage was lower than 0.25

(ii) putatively present, if the coverage, normalized on the haploid peak of coverage was >= 0.25

Although these thresholds were arbitrarily set, we choose to use a strict limit to call “absence” (0.25), to put a major focus on the identification with high confidence of putatively absent genes, at the cost of the detection of some false positives.

## 8.2. Coverage normalization

The detection of the main (homozygous) peak of coverage was performed manually, by the visual inspection of plots summarizing read mapping densities for all the libraries obtained from females. Due to the unusual distributions observed in males (see details in **Data Note 23**), the homozygous peak was detected as follows: a subset of 4,277 genes displaying a “highly stable” mapping rate within the homozygous peak in female mussels (average normalized coverage between 1.95 and 2.05, with a standard deviation lower than 0.2) was identified. The average coverage of such stable genes was calculated in male libraries and the average value obtained for each genome was used as a reference for normalization.

## 8.3. A large amount of the assembly displays a sequencing coverage consistent with an hemizygous state

The first observation gathered from the inspection of the per-base coverage graph of *Lola* (PE reads obtained from mantle) was that a large fraction of the genome assembly shows a haploid (1n) coverage, exactly in line with half of expectations (~41.5X vs ~83X), suggesting the massive presence of genomic regions found in an hemizygous state (**Fig. S32**). Estimates of the area included in the “hemizygous peak” of coverage indicated that it nearly matched the area corresponding to the “homozygous peak” (=2n) of coverage (plus the area with multiple mappings). Overall, the haploid peak of coverage accounted for 36.78% of the *Lola* genome assembly (excluding regions with a very low coverage. i.e., <13X). As the mapping parameters used were rather stringent, these results might have been somewhat consistent with the extreme heterozygosity rate of the mussel genome (see **Data Note 6**) and the widespread presence of SNPs, especially in non-coding regions, which are expected to be subjected to lower evolutionary constraints.


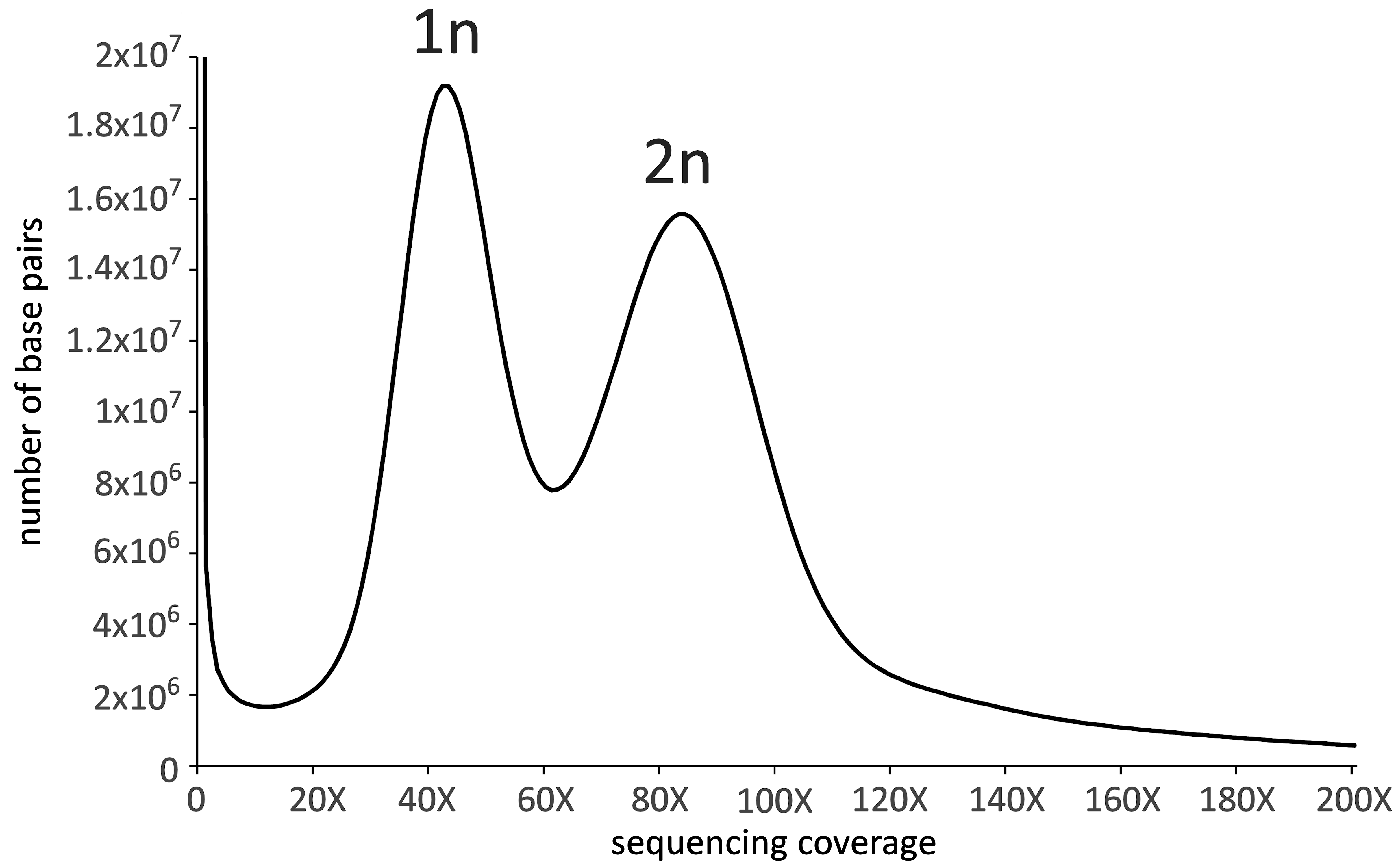


**Fig. S32**. **Summary of the coverage of the *Lola* assembly at a single base scale**. The coverage was evaluated by the mapping of Illumina PE reads obtained from the mantle tissue. Two main peaks of coverage are clearly visible, corresponding to the hemizygous (1n, ~41.5X) and homozygous (2n, ~83X) peaks of coverage based on genome size estimates. The peak located at 0 indicates approximately 70 Mb of genome assembly which did not achieve any mapping based on Q >= 60.

## 8.4. Detailed evaluation of gene coverage in *Lola*

The analysis was therefore extended at the gene level, to *Lola* and to all resequenced genomes, as described above. *Lola* sequencing data from mantle and gills were independently analyzed, providing highly correlated results (**Figures S33**) (R^2^ = 0.917; p-value < 0.0000001) (**Fig. S34**). This confirmed the reliability of this approach, supporting the use of read mapping data and inferred coverages as a proxy for detecting PAV and estimating the hemizygous/homozygous coverage of the genes annotated in mussel genomes.

The detailed analysis of the mapping peaks revealed that the bimodal distribution observed at the nucleotide scale was maintained at the gene level, even though the relative size of the hemizygous peak, compared with the homozygous peak, was reduced, consistently with the localization of a relevant amount of sequence variation within non-coding genomic regions. The bimodal distribution of mapping peaks still revealed that an important fraction of the protein-coding genes annotated in *Lola* was present with a single allele in the genome, or that the allelic variant present in the homologous chromosome was divergent enough to prevent the cross-mapping of reads (see **Data Note10**). In detail, the genes predicted to fall within the hemizygous peak of coverage (i.e., with normalized coverage < 1.5) were 21,400 (35.47% of the total) and 20,696 (34.30% of the total) in mantle and gills, respectively. Overall, roughly one third of the protein-coding genes of the mussel genome displayed a coverage level consistent with the presence of a single gene allele in the diploid genome, pointing out such genes were encoded by widespread genomic regions subject to hemizygosity.


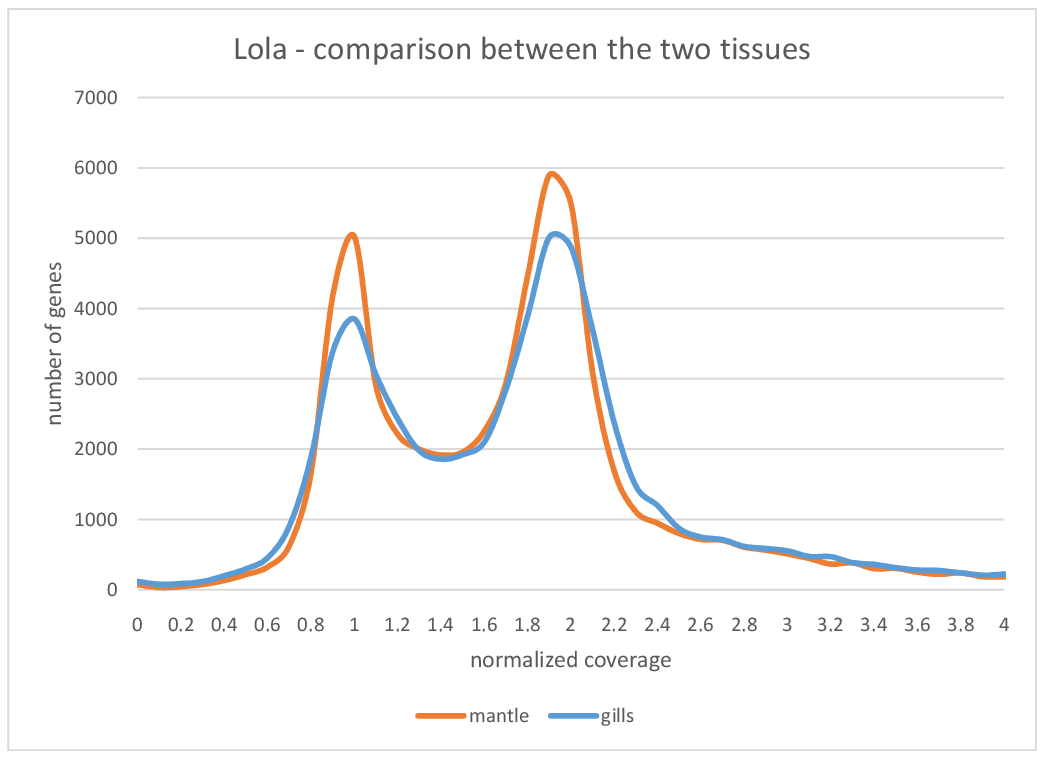


**Fig. S33**. **Comparative overview of per gene coverage of *Lola* mg10 genes.** Coverage was normalized on the expected haploid mussel genome size, calculated based on the mapping of Illumina PE library obtained from gills and mantle tissue in the same individual.


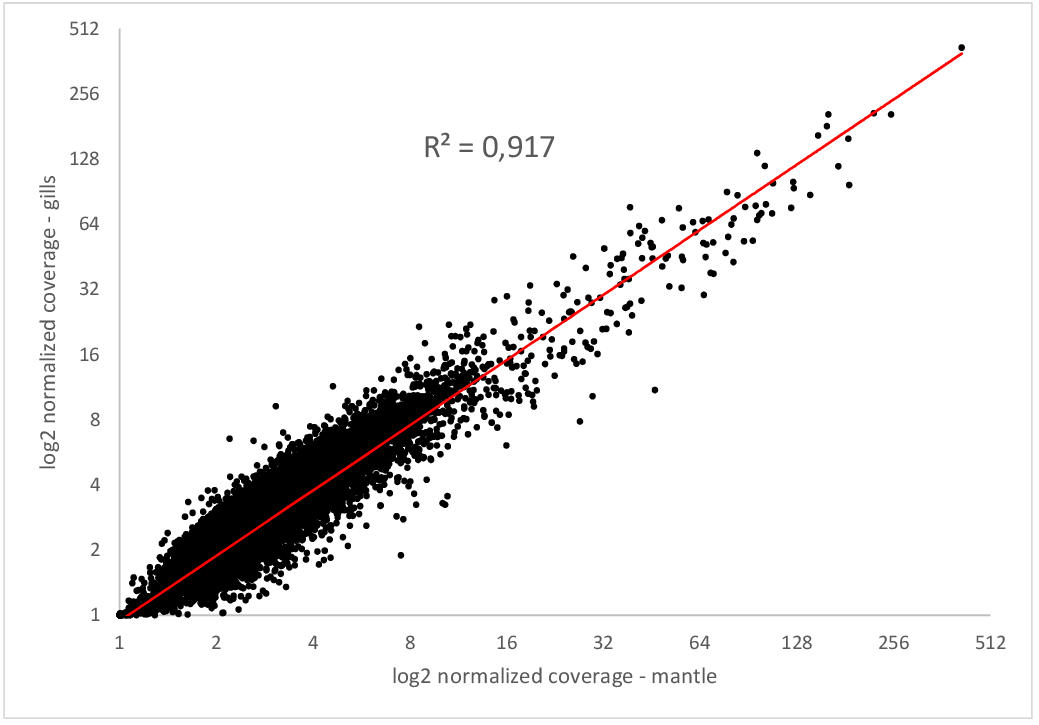


**Fig. S34**. **Correlation between “per gene coverage” data**. Coverage was normalized on the expected haploid mussel genome size), estimated from the mapping of Illumina PE libraries generated from *Lola* gills and mantle Illumina PE libraries. The data series obtained from the two tissues are highly correlated (R^2^ = 0.917. linear regression analysis p-value < 0.000001).

## 8.5. Detailed evaluation of gene coverage in the resequenced genomes

Mapping data obtained from the resequenced genomes immediately revealed an unexpected trend, highlighting some similarities, as well as some striking differences with respect *Lola*. First, a main peak corresponding to the coverage expected for genomic region found in an homozygous state was always observable. Still, compared to *Lola*, the size of the secondary hemizygous peak was much reduced. At the same time, however, the number of genes displaying extremely low normalized coverage (i.e., lower than 0.25 and consistent with gene absence) increased (**Figures S35-S40**, also see main text). Similar profiles were obtained for the genomes of all resequenced female mussels, including *Pura* [17] and most male mussels, with a few notable exceptions, which is discussed in detail in **Data Note 23**. The calculation of the mapping profiles from male mussels required a calibration procedure, which is also described in detail in **Data Note 23**.


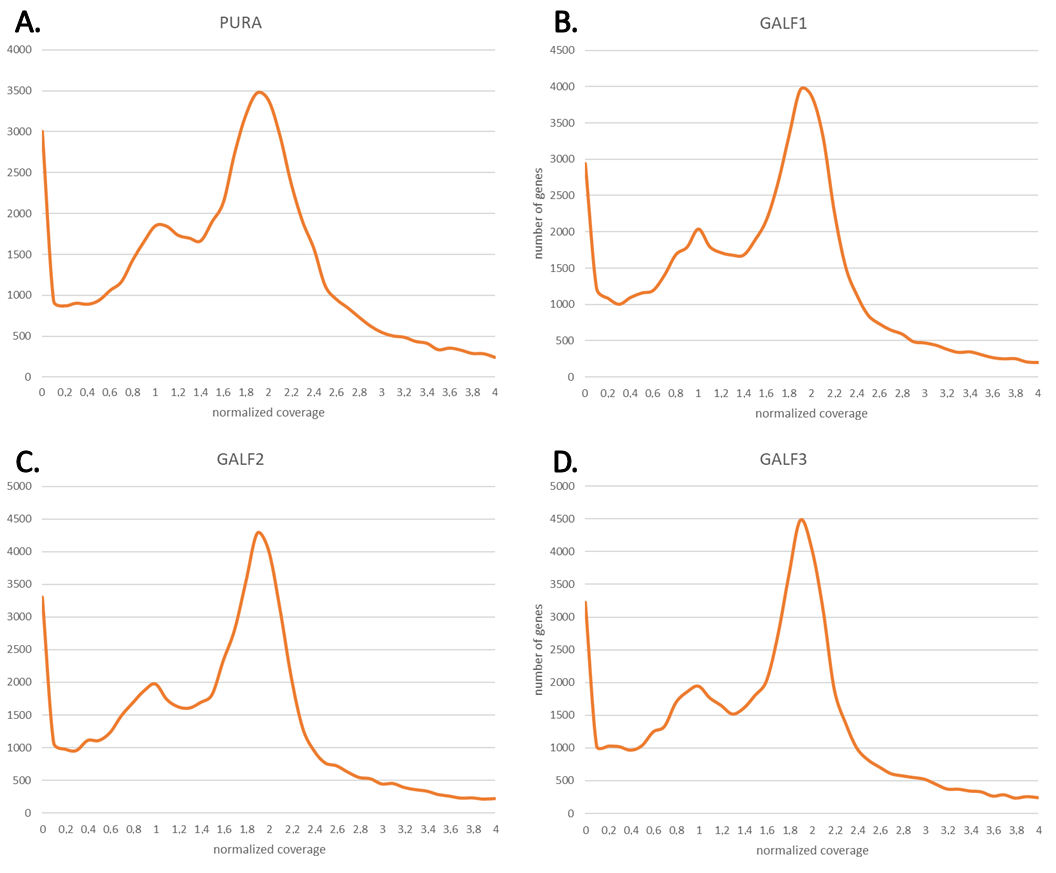


**Fig. S35. Per gene coverage of *Lola* mg10 genes for *Pura* and GALF1-3.** Coverage was normalized on the expected haploid mussel genome size, calculated based on the mapping of Illumina PE libraries obtained from the mantle tissue of (A) *Pura*, (B) GALF1, (C) GALF2 and (D) GALF3.


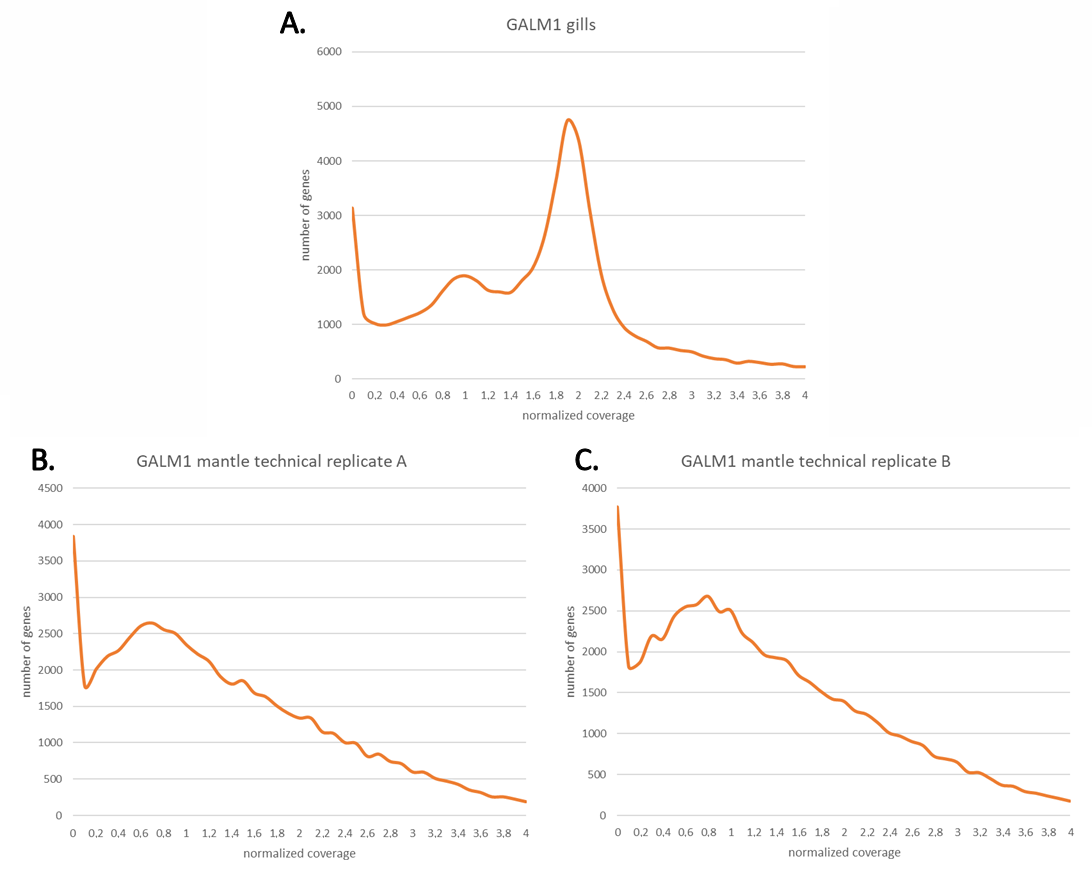


**Fig. S36. Per gene coverage of *Lola* mg10 genes for GALM1.** Coverage normalized on the expected haploid mussel genome size, calculated based on the mapping of Illumina PE libraries obtained from (A) the gills and mantle (B-C) tissues of GALM1. B and C represent two technical replicates.


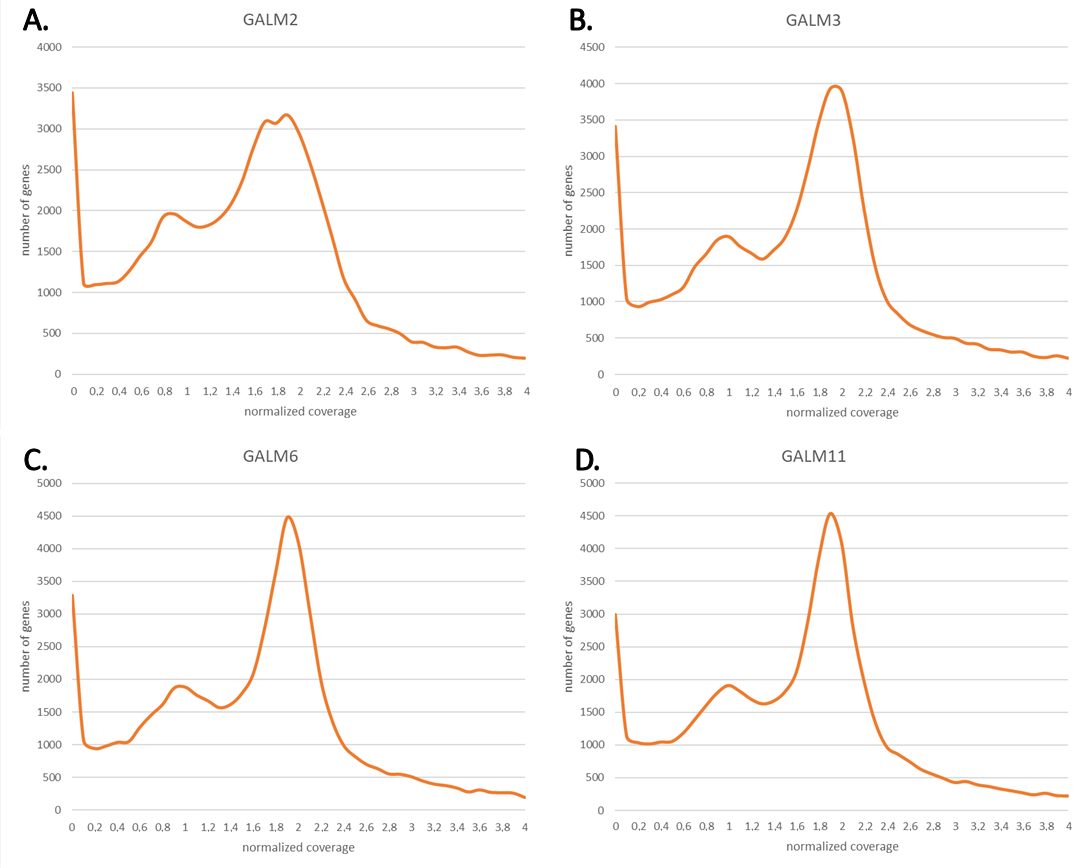


**Fig. S37. Per gene coverage of *Lola* mg10 genes for GALM2, GALM3, GALM6 and GALM11.** Coverage was normalized on the expected haploid mussel genome size, calculated based on the mapping of Illumina PE libraries obtained from the mantle tissue of (A) GALM2, (B) GALM3, (C) GALM6, (D) GALM11.


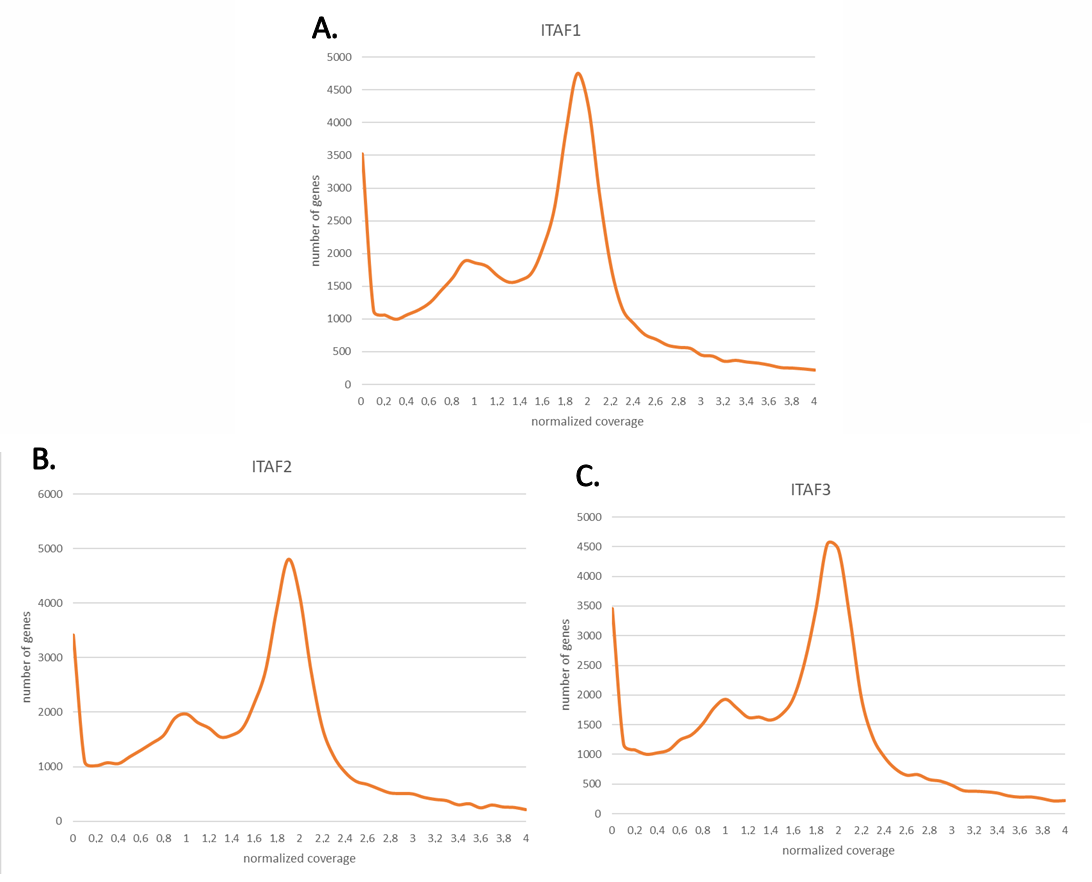


**Fig. S38**. **Per gene coverage of *Lola* mg10 genes for ITAF1-ITAF3**. Coverage was normalized on the expected haploid mussel genome size, calculated based on the mapping of Illumina PE libraries obtained from the mantle tissue of (A) ITAF1, (B) ITAF2 and (C) ITAF3.


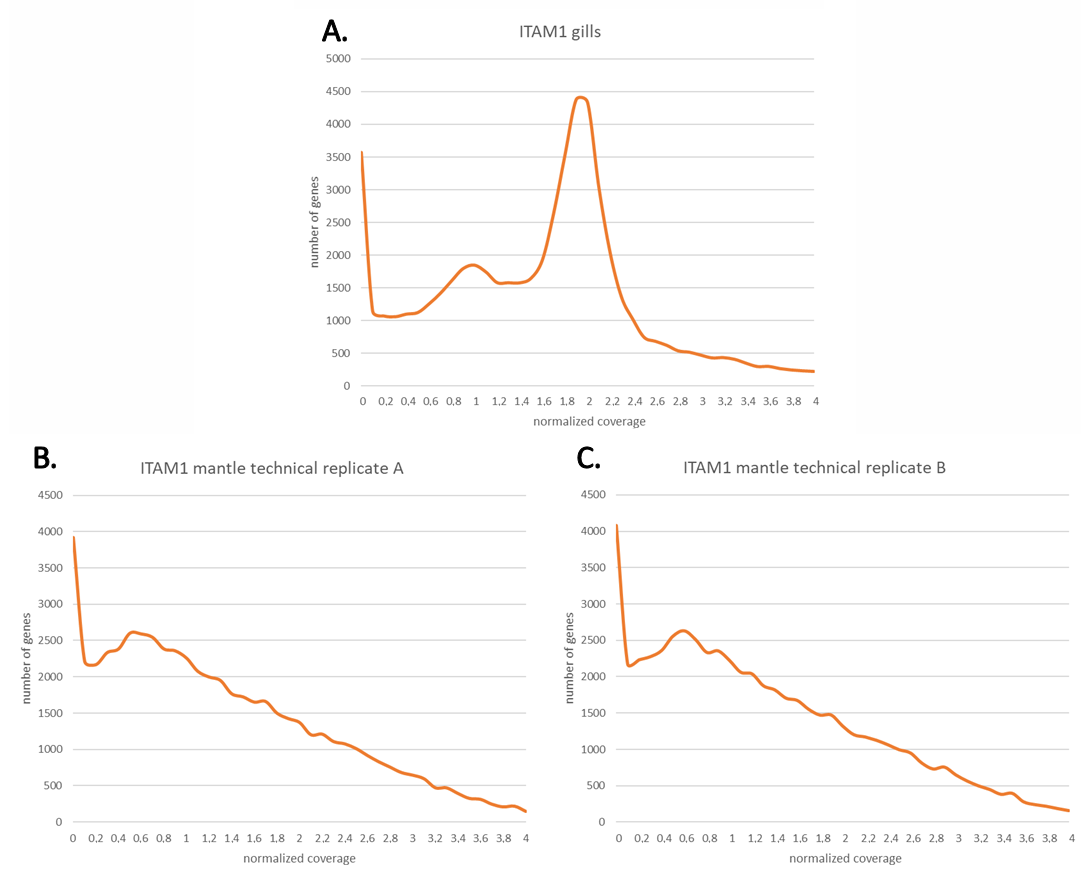


**Fig. S39. Per gene coverage of *Lola* mg10 genes for ITAM1**. Coverage was normalized on the expected haploid mussel genome size, calculated based on the mapping of Illumina PE libraries obtained from (A) the gills and mantle (B-C) tissues of ITAM1. B and C represent two technical replicates.


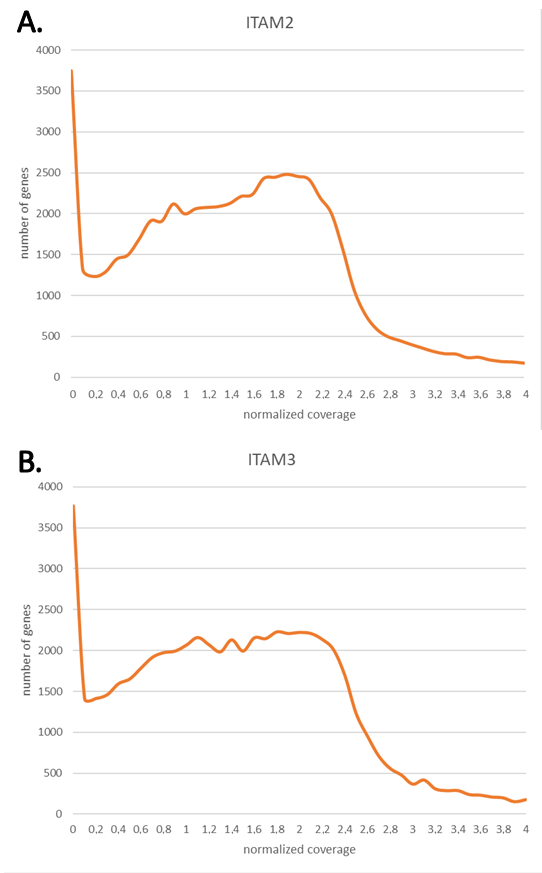


**Fig. S40**. **Per gene coverage of *Lola* mg10 genes for ITAM2 and ITAM3.** Coverage was normalized on the expected haploid mussel genome size, calculated based on the mapping of Illumina PE libraries obtained from the mantle tissue of (A) ITAM2 and (B) ITAM3.

This analysis enabled a detailed investigation of presence-absence variation and resulted in the detection of a significant fraction of genes subjected to presence-absence variation (PAV) in all the analyzed genomes. The estimates of the number of absent genes (normalized average coverage < 0.25) are summarized in **Fig. S41**. These ranged from a minimum of 4,234 (7.02% of the total) in *Pura* to a maximum of 5,667 in ITAM3 (9.39% of the total). On average, 4,829 genes, accounting for 8% of *Lola* protein-coding genes, were absent in each of the resequenced genomes. However, while the lack of a number of genes was shared by different mussel genomes, on many occasions a given gene was uniquely lacking in one out of the 14 resequenced genomes (+ *Pura*). Overall, the occurrence of PAV for all genes is reported in **Fig. S42**. This analysis revealed a *core* set consisting of 45,518 genes which were present in all genomes, as opposed to a relatively large set of 14,570 genes (24.15% of the total) which were absent in at least one of the resequenced genomes and which were therefore labeled as *dispensable* genes. *Dispensable* genes were apparently characterized by markedly variable frequencies of occurrence, as 3,765 of them (6.27% of the total) were found in but one all mussel genomes and, on the opposite extreme, 304 genes (0.51% of the total) were exclusively found in *Lola* (**Fig. S42**).


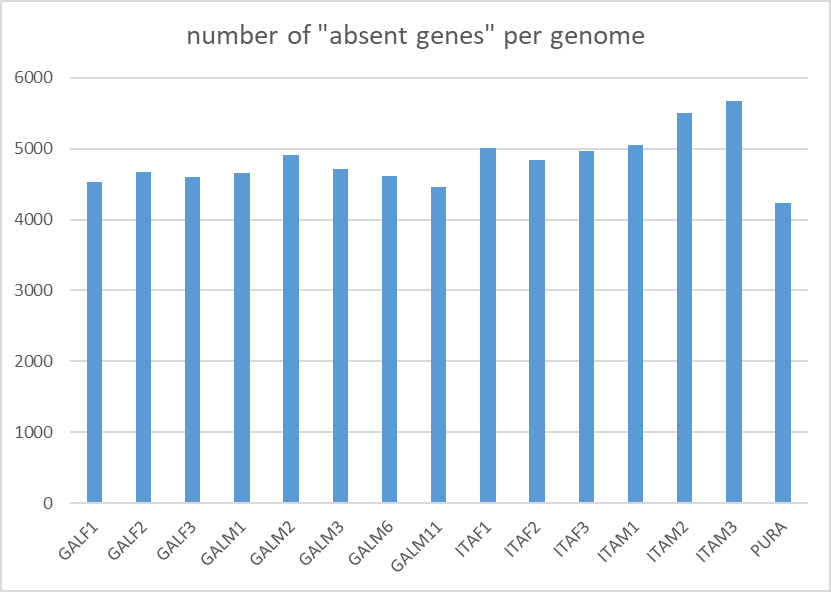


**Fig. S41**.**Number of genes absent in each of the resequenced genomes**. Absent genes were defined as those displaying a normalized coverage lower than 0.25.


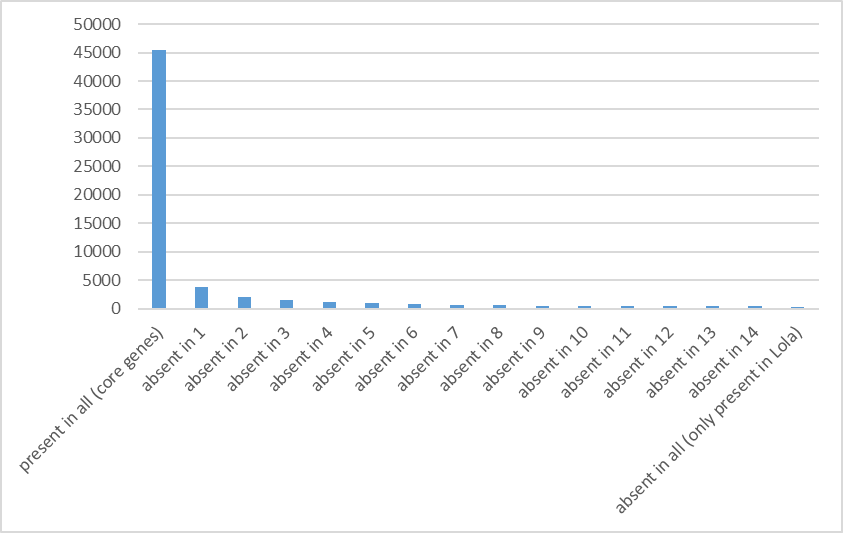


**Fig. S42. Occurrence of PAV in the 14 resequenced mussel genomes, plus *Pura***. Data detailed in **Table S37** below.

**Table S37**.Summary of presence-absence variation (PAV) of *Lola* genes in the 14 resequenced genomes (plus *Pura*). The absolute number and the percentage compared to the total are indicated. Genes marked as “present in all” are considered as *core* genes which were detected in all the 16 sequenced mussel genomes. Genes classified in the other categories are classified as *dispensable* genes, present in different frequency in mussel populations. Genes marked as “absent in all” were not detected neither in any of the 14 resequenced genomes nor in *Pura* (i.e., they were only present in *Lola*).

| **category** | **count** | **% of total** |
| --- | --- | --- |
| present in all (*core* genes) | 45.518 | 75.75 |
| absent in 1 | 3.765 | 6.27 |
| absent in 2 | 2.078 | 3.46 |
| absent in 3 | 1.449 | 2.41 |
| absent in 4 | 1.152 | 1.92 |
| absent in 5 | 950 | 1.58 |
| absent in 6 | 759 | 1.26 |
| absent in 7 | 720 | 1.20 |
| absent in 8 | 560 | 0.93 |
| absent in 9 | 539 | 0.90 |
| absent in 10 | 495 | 0.82 |
| absent in 11 | 479 | 0.80 |
| absent in 12 | 464 | 0.77 |
| absent in 13 | 440 | 0.73 |
| absent in 14 | 416 | 0.69 |
| absent in all (only present in *Lola*) | 304 | 0.51 |

# Data Note 9 – Non-coding genes are also subject to Presence-Absence Variation

## Detailed evaluation of non-coding gene coverage in *Lola* and in the resequenced genomes

The same analyses carried out for protein-coding genes outlined in section 8.5 were extended to the 73,097 non-coding genes annotated in mg10. The criteria used for labeling a gene as “present” or “absent” were identical to those described above, and made use of arbitrary coverage thresholds (i.e., 0.25 of the expected “hemizygous coverage”) and calibration. The results obtained closely mirrored those previously described for protein-coding genes, pointing out that the PAV phenomenon is not restricted to genes with protein-coding potential only, but it also affects larger genomic regions embedding non-coding genes and, possibly, relatively large inter-genic regions (as the base-by-base coverage analysis strongly hints to, see **Fig. S32**). In *Lola*, the sequencing coverage of non-coding genes clearly followed a two-peaked distribution, similar to the one previously evidenced for protein-coding genes (**Fig. S43**, see **Fig. S33** for comparison), highlighting the presence of a relevant number of non-coding genes in hemizygous genomic regions, in addition to the main group of non-coding genes found within the homozygous peak of coverage. As in the case of protein-coding genes, the coverage distributions observed in the two tissues were highly correlated (R^2^ = 0.848, linear regression analysis p-value < 0.00001), ruling out the possibility of this distribution being an artefact (**Fig. S44**). A significant difference between the profiles obtained for protein-coding and non-coding genes stands in the fact that the latter graphs show a curve which is more “leaning” towards higher coverages and larger peaks, which we believe might be explained by the following factors:

1. A relatively high fraction of non-coding genes was inferred to be present with multiple copies, possibly due to the inclusion of several active transposable elements (see **Data Note 2**). In *Lola*, just 2.15% protein-coding genes were inferred to be present with more than two alleles in the diploid genome (i.e., had a coverage higher than 2.5X the expected coverage of a single-copy gene). On the other hand, this number was 8.5 times higher (i.e., 18.56%) for non-coding genes. This may obviously lead to significant read cross-mapping among paralogous genes.
2. Compared with protein-coding genes, non-coding genes are expected to be subject to weaker selective constraints, allowing a higher rate of sequence variation among allelic variants of the same gene, consequently determining much larger fluctuations in read mapping rates.


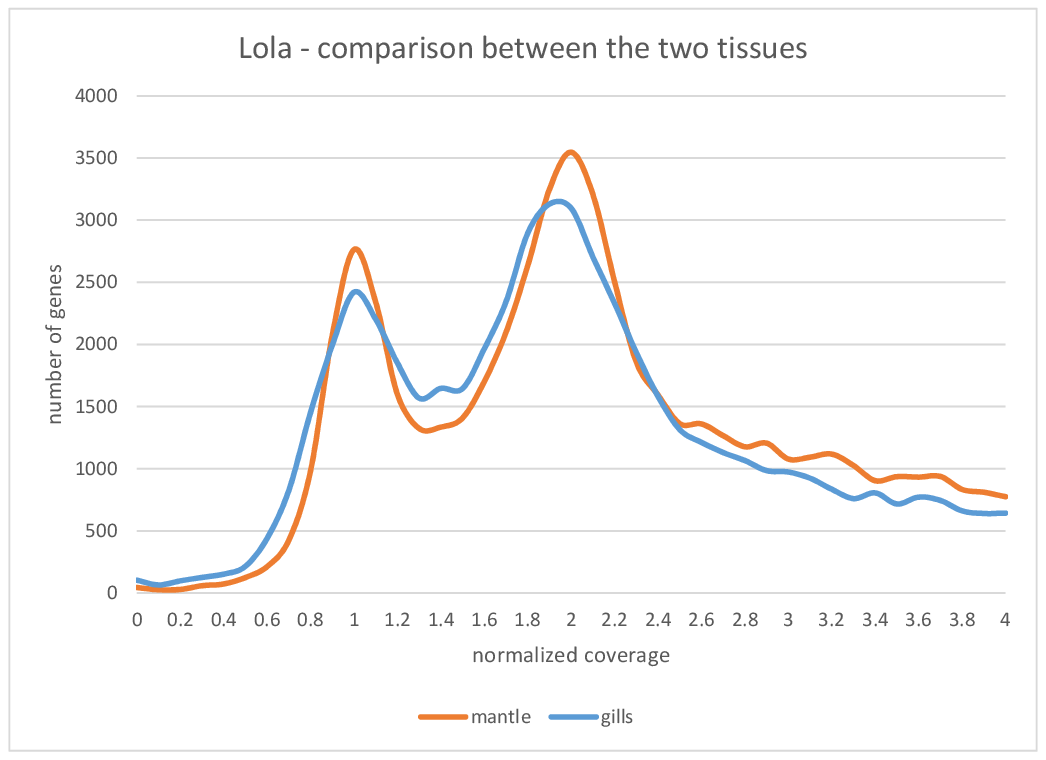


**Fig. S43**. **Comparative overview of per gene coverage of *Lola* mg10 genes, with reference to non-coding genes.** Coverage was normalized on the expected haploid mussel genome size, calculated based on the mapping of Illumina PE library obtained from gills and mantle tissue in the same individual.


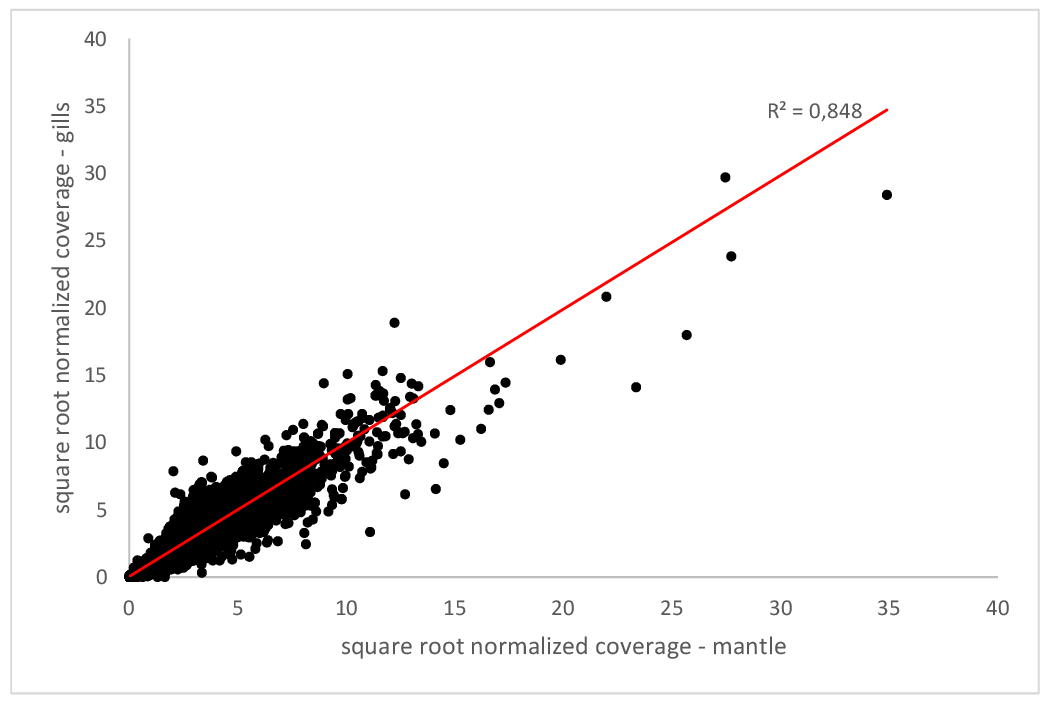


**Fig. S44**. **Correlation between per gene coverage data in the mantle and gills samples of *Lola*. Coverage is referred to non-coding genes**. Coverage was normalized on the expected haploid mussel genome size), estimated from the mapping of Illumina PE libraries generated from *Lola* gills and mantle Illumina PE libraries, and further square root-transformed. The data series obtained from the two libraries are highly correlated (R2 = 0.848. linear regression analysis p-value < 0.00001).

The coverage profiles obtained for the 15 resequenced genomes also closely matched those observed for protein-coding genes, with the two main peaks being evident in most cases, even though in this case the hemizygous peak was often just visible as a “shoulder” and the main homozygous peak was broader. As explained above, this may be explained by the combination of a higher rate of cross-mapping and a lower degree of sequence conservation among allelic variants, compared with protein-coding genes. In any case, the number of genes displaying extremely low normalized coverage (i.e., lower than 0.25 and consistent with gene absence) was remarkable in all genomes (**Figures S45-S48**). As previously noted for protein-coding genes, the few samples that displayed an “anomalous” graph (i.e., mostly ITAM2 and ITAM3) will be extensively discussed in **Data Note 23**, which also describes the calibration procedure utilized to generate these graphs. Therefore, for the sake of simplicity, the following graphs will only display the coverage graphs obtained from the gill tissue of GALM1 and ITAF1, disregarding the data obtained from the DNA extraction and sequencing performed from the mantle of the same individual.


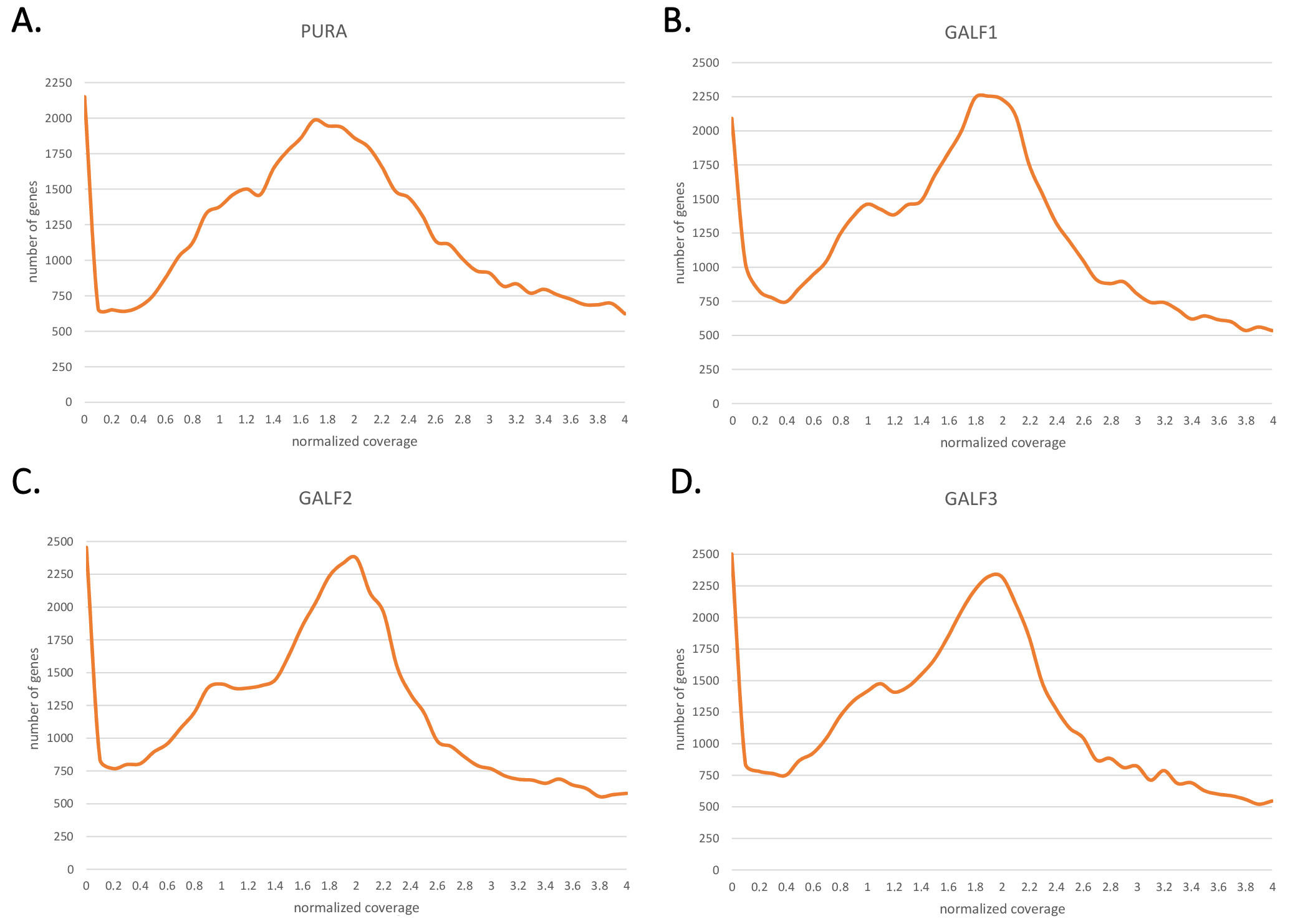


**Fig. S45. Per gene coverage of *Lola* mg10 non-coding genes for *Pura* and GALF1-3.** Coverage was normalized on the expected haploid mussel genome size, calculated based on the mapping of Illumina PE libraries obtained from the mantle tissue of (A) *Pura*, (B) GALF1, (C) GALF2 and (D) GALF3.


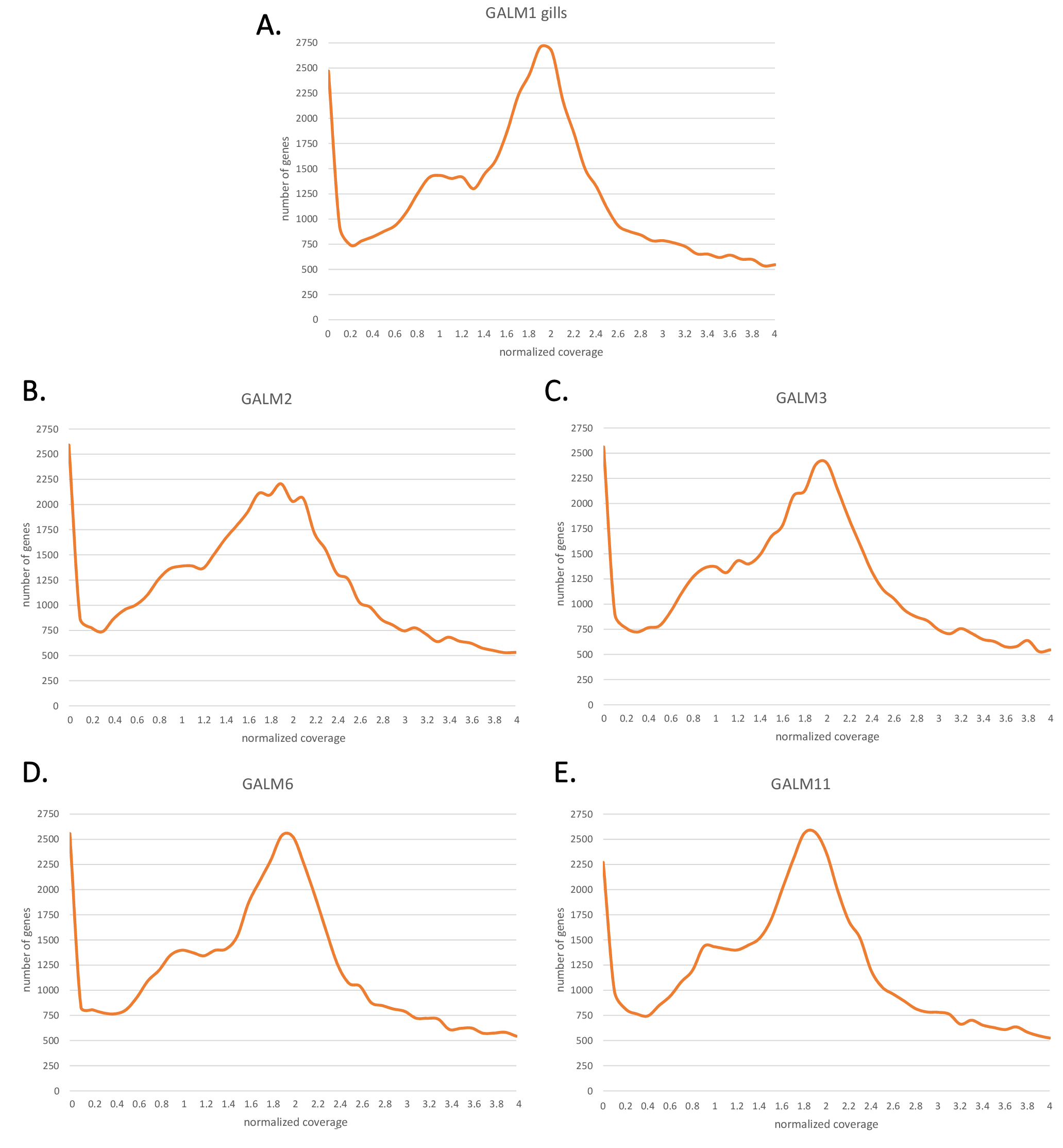


**Fig. S46. Per gene coverage of *Lola* mg10 non-coding genes for GALM1, GALM2, GALM3, GALM6 and GALM11.** Coverage was normalized on the expected haploid mussel genome size, calculated based on the mapping of Illumina PE libraries obtained from the gill tissue of (A) GALM1, and from the mantle tissue of (B) GALM2, (C) GALM3, (D) GALM6, (E) GALM11.


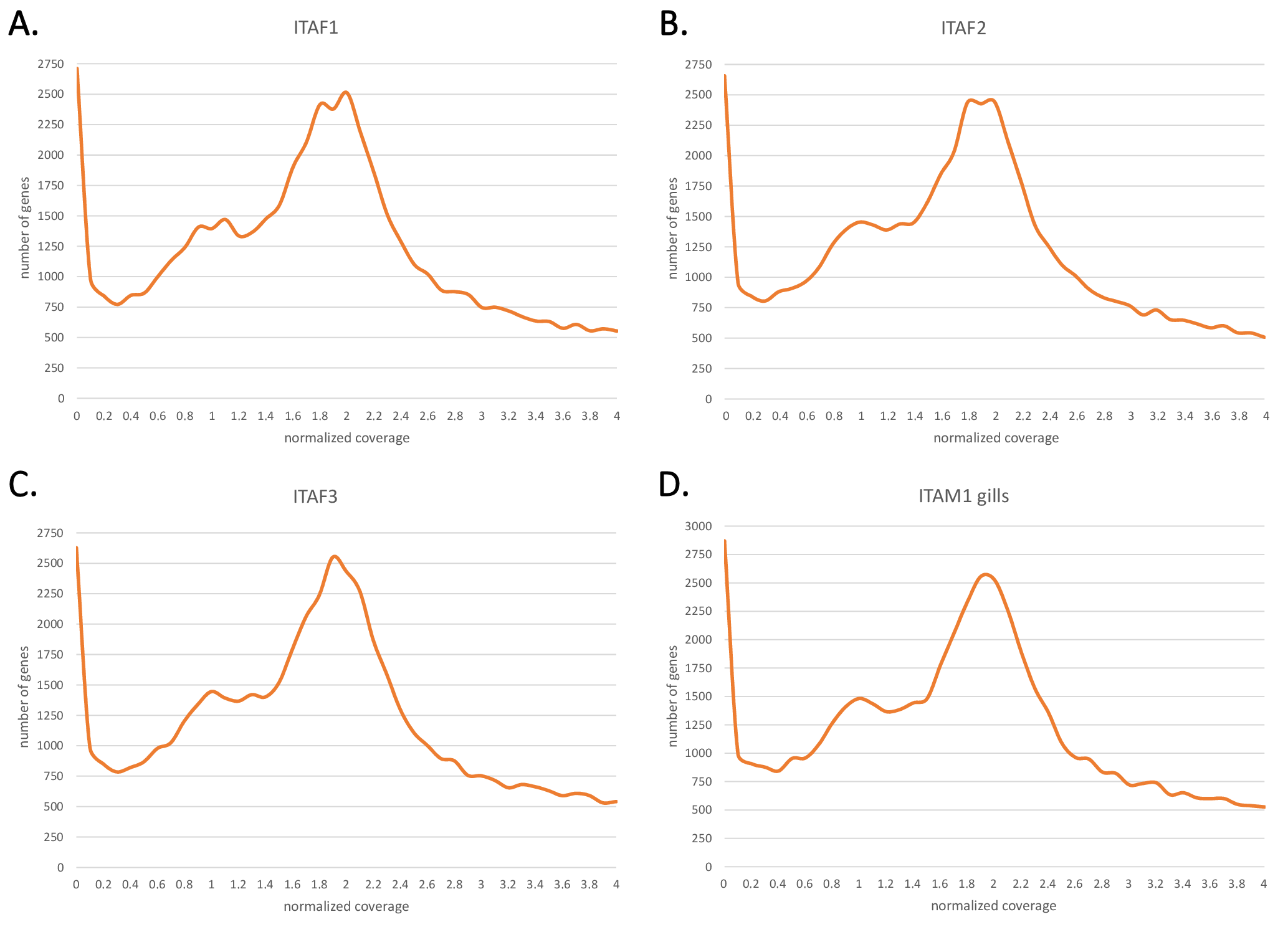


**Fig. S47**. **Per gene coverage of *Lola* mg10 genes for ITAF1, ITAF2, ITAF3 and ITAM1**. Coverage was normalized on the expected haploid mussel genome size, calculated based on the mapping of Illumina PE libraries obtained from the mantle tissue of (A) ITAF1, (B) ITAF2 and (C) ITAF3, and from the gill tissue of (D) ITAM1.


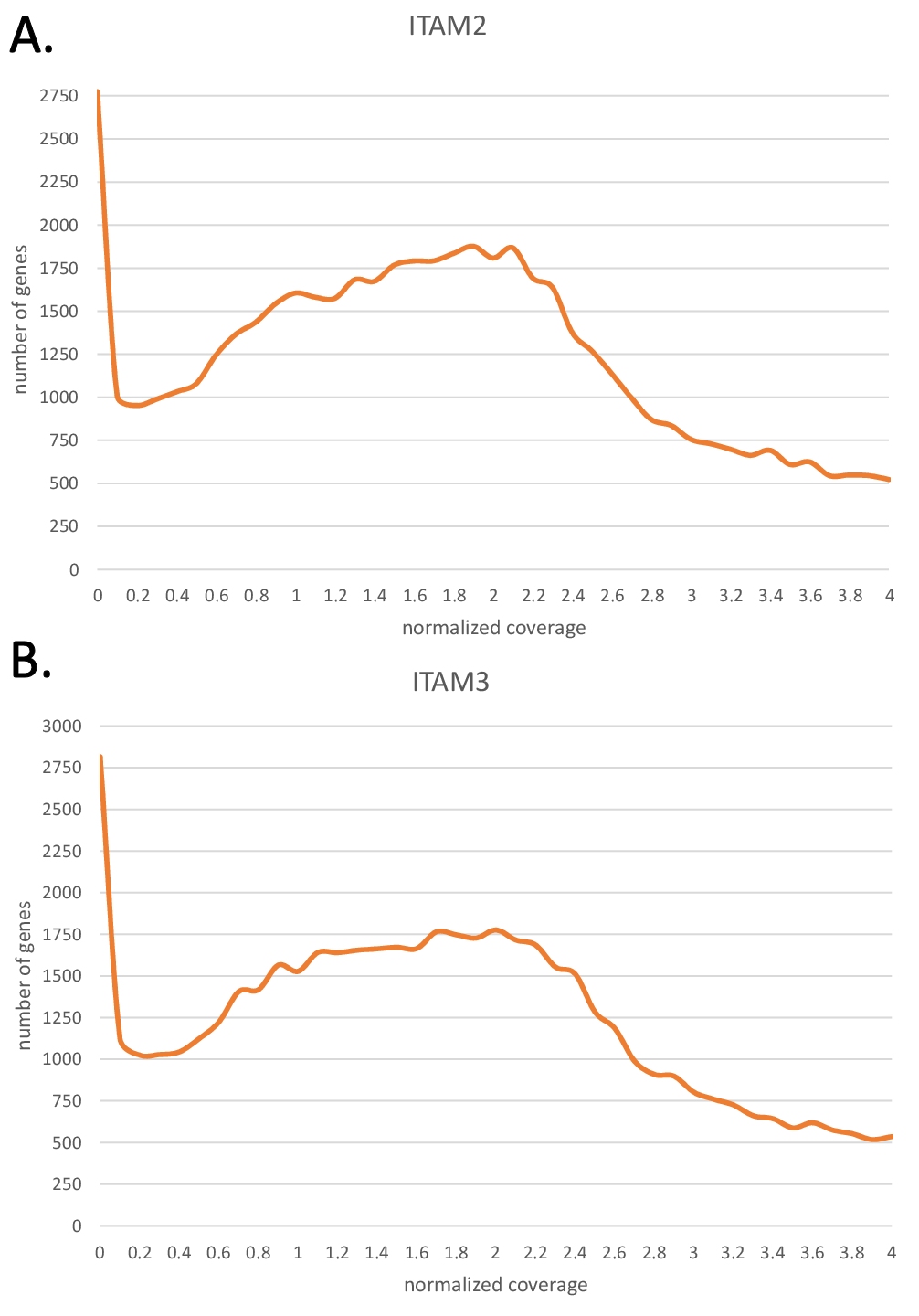


**Fig. S48**. **Per gene coverage of *Lola* mg10 non-coding genes for ITAM2 and ITAM3.** Coverage was normalized on the expected haploid mussel genome size, calculated based on the mapping of Illumina PE libraries obtained from the mantle tissue of (A) ITAM2 and (B) ITAM3.

Overall, a significant number of non-coding genes were subject to PAV in all the analyzed genomes. The estimates of the number of absent genes (normalized average coverage < 0.25) are summarized in **Fig. S49**. These ranged from a minimum of 3,229 (4.42% of the total) in *Pura* to a maximum of 4,311 in ITAM3 (5.90% of the total). On average, 3,744 genes, accounting for 5.12% of all *Lola* non-coding genes, were absent in the resequenced genomes. This fraction was somewhat lower compared to the one observed for protein-coding genes (i.e., 8%, see **section 8.5**), in line with our observation that several non-coding genes are present with multiple copies and, hence, less subject to the PAV phenomenon.

Overall, the occurrence of PAV for all non-coding genes in the 14 resequenced individuals (+*Pura*) is reported in **Fig. S50**. Based on the outcome of this analysis, the *core* set of non-coding genes included 60,883 genes which were present in all genomes, whereas the *dispensable* gene set was proportionally much smaller than that of protein-coding genes, including 12,214 genes (16.71% of the total). As in the case of protein-coding genes, *dispensable* non-coding genes had a very variable frequency of occurrence: in this case, 3,335 (27.30% of the total) *dispensable* genes were found in all but one mussel genomes. On the other hand, 144 *dispensable* non-coding genes (1.18% of the total) were exclusively found in *Lola* (**Table S38**).


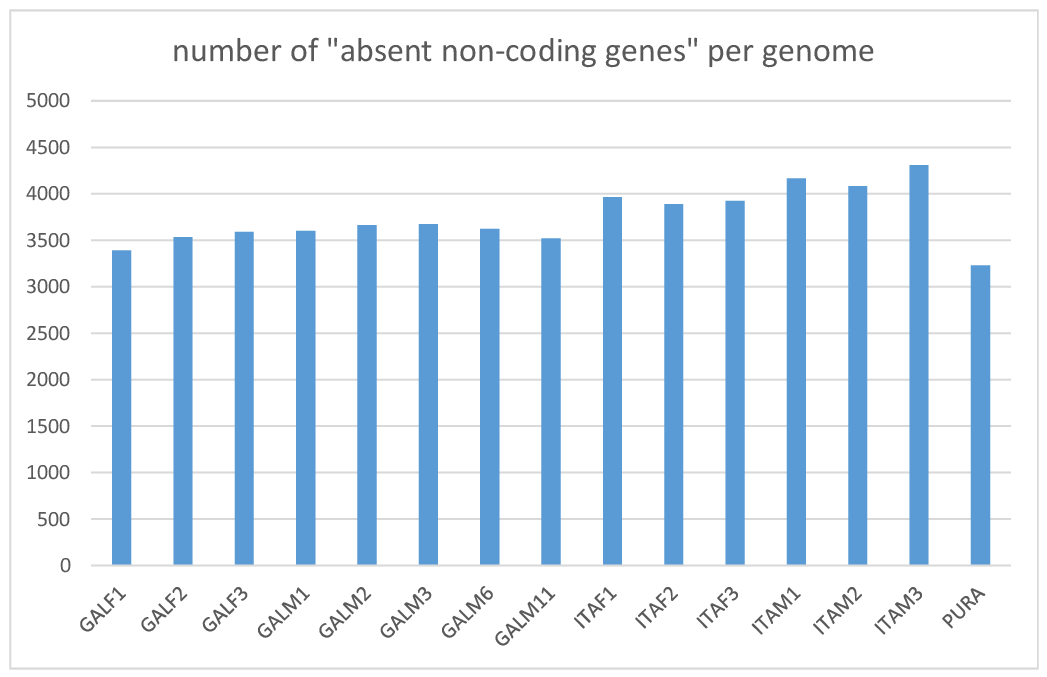


**Fig. S49**. **Number of non-coding genes absent in each of the resequenced genomes**. Absent genes were defined as those displaying a normalized coverage lower than 0.25.


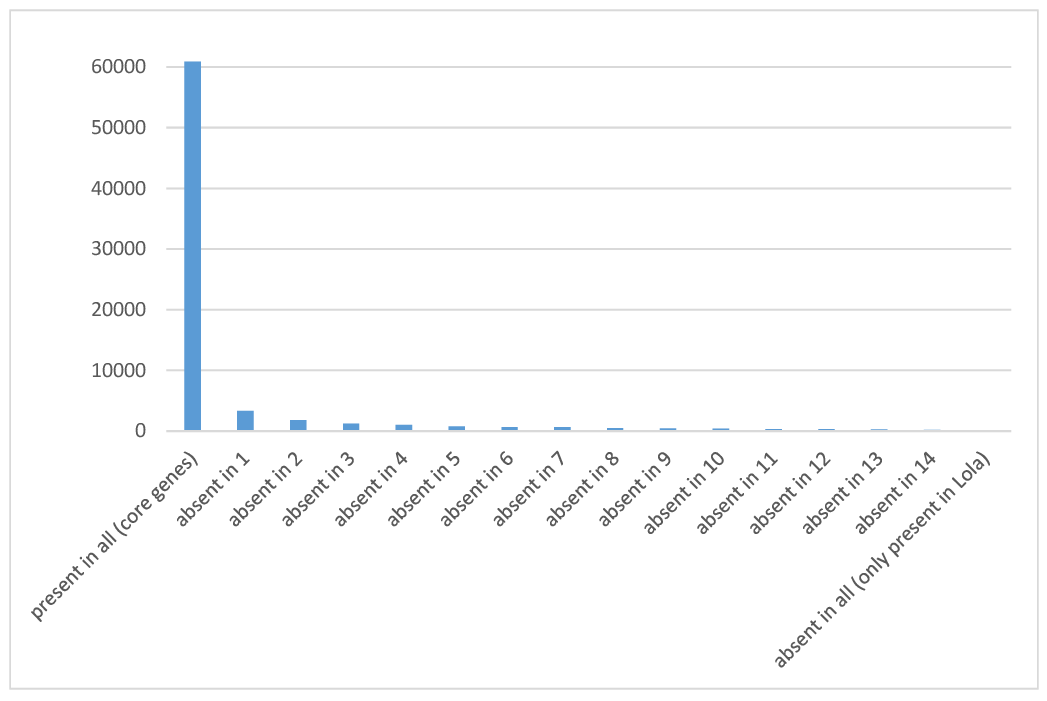


**Fig. S50. Occurrence of PAV in the 14 resequenced mussel genomes, plus *Pura***. Note that the Y axis reports numbers in a log_2_ scale. Data are detailed in **Table S38** below.

**Table S38**. Summary of presence-absence variation (PAV) of *Lola* non-coding genes in the 14 resequenced genomes (plus *Pura*). The absolute number and the percentage compared to the total are indicated. Genes marked as “present in all” are considered as *core* genes which were detected in all the 16 sequenced mussel genomes. Genes classified in the other categories are classified as *dispensable* genes, present in different frequency in mussel populations. Genes marked as “absent in all” were not detected neither in any of the 14 resequenced genomes nor in *Pura* (i.e., they were only present in *Lola*).

| **category** | **count** | **% of total** |
| --- | --- | --- |
| present in all (*core* genes) | 60,883 | 83.29 |
| absent in 1 | 3,335 | 4.56 |
| absent in 2 | 1,791 | 2.45 |
| absent in 3 | 1,256 | 1.72 |
| absent in 4 | 1,044 | 1.43 |
| absent in 5 | 805 | 1.10 |
| absent in 6 | 651 | 0.89 |
| absent in 7 | 637 | 0.87 |
| absent in 8 | 509 | 0.70 |
| absent in 9 | 450 | 0.62 |
| absent in 10 | 416 | 0.57 |
| absent in 11 | 351 | 0.48 |
| absent in 12 | 324 | 0.44 |
| absent in 13 | 296 | 0.40 |
| absent in 14 | 205 | 0.28 |
| absent in all (only present in *Lola*) | 144 | 0.20 |

# Data Note 10 – PAV cannot be generally explained by divergence between allelic variants

## Effects of read mapping stringency on coverage estimates

We evaluated whether the hemizygous peak of coverage could be explained by the divergence between allelic variants and, in that case, what would be the expected degree of pairwise divergence between two sequences to enable the generation of the observed mapping profiles. This analysis was carried out using real data obtained from *Lola* (gills paired-end Illumina sequencing data) and a subset of 4,896 genes displaying a “hemizygous normalized coverage” (i.e., 1n +/- 0.25) in this genome (once again based on gills PE Illumina sequencing data). We mapped the sequencing reads to the coding region (of the longest transcript) of each gene, using different mapping stringencies, with the *map reads to contigs* tool included in the CLC Genomics Workbench 11 (Qiagen, Hilden, Germany). Namely, decreasing *similarity fraction* (SF) values were used, i.e., 0.98, 0.96, 0.94, 0.9, 0.85 and 0.8, thereby tolerating 2, 4.6, 10, 15 and 20% sequence polymorphisms in the alignment between reads and the reference genome. The *length fraction* parameter was always kept at 0.5 to enable the mapping of reads located on exon/intron junctions.

Theoretically, under the hypothesis that reads originated from divergent alleles are only mapped on one out of the two variants using stringent parameters (i.e., SF = 0.98), the progressive decrease of stringency should also enable the non-specific mapping of such reads to the other variant. The results revealed that the shift between the hemizygous and the homozygous peak could be explained by inter-allelic divergence only for a minor fraction of *Lola* genes (**Fig. S51**). Namely, while a generalized shift towards the homozygous peak of coverage could be observed with decreased mapping stringency (due to the increased occurrence of non-specific mappings), a large number of genes remained within the hemizygous peak. In detail, nearly half of the tested genes maintained a similar coverage even when a divergence as high as 20% was allowed (**Fig. S52**). At the same time, only a relatively minor fraction of genes shifted to the homozygous peak (28% when SF=0.8) (**Fig. S53**). It also needs to be taken into account that the amount of mapped reads increased enormously for many genes (exceeding the coverage expected for a single-copy gene found in a homozygous state, **Fig. S54**), indicating the massive cross-mapping of reads originated from paralogous gene copies. This was already visible, to some extent, with a relatively little decrease of stringency, but assumed massive proportions for divergence >10% (**Fig. S53**). In detail, the progressive lowering of the stringency parameter induced the following changes:

-SF = 0.98: all genes fell within the hemizygous peak

-SF = 0.96: the large majority of genes (88.58%) remained within the hemizygous peak, while 9.44% moved to the homozygous peak.

SF = 0.94: the trend continued, with 80.92% genes remaining within the hemizygous peak and 14.62% moving to the homozygous peak.

SF = 0.9: 65.5% genes remained in the hemizygous peak, whereas 22.71% moved to the homozygous peak. Here, a significant number of genes (11.76%) displayed coverages higher than 2n, due to non-specific cross-mapping.

SF = 0.85: 52.72% genes maintained a hemizygous coverage, 26% genes shifted to the homozygous peak and 20.22% genes shifted to higher coverages.

SF = 0.8: 44.93% genes maintained a hemizygous coverage, 28.06% shifted to the homozygous peak and 27% genes shifted to higher coverages.

Overall, our experiment suggests that allelic divergence may explain the phenomenon of PAV just for a minor fraction of the annotated mussel genes (no more than 15% and 25%, assuming an average allelic divergence at the nucleotide level in the order of 5 and 10%, respectively). Higher levels of allelic divergence, in the order of 20%, would still not be able to explain PAV for about 45% of the genes falling within the hemizygous peak of coverage (i.e., those allegedly present with a single allele in the reference *Lola* genome).


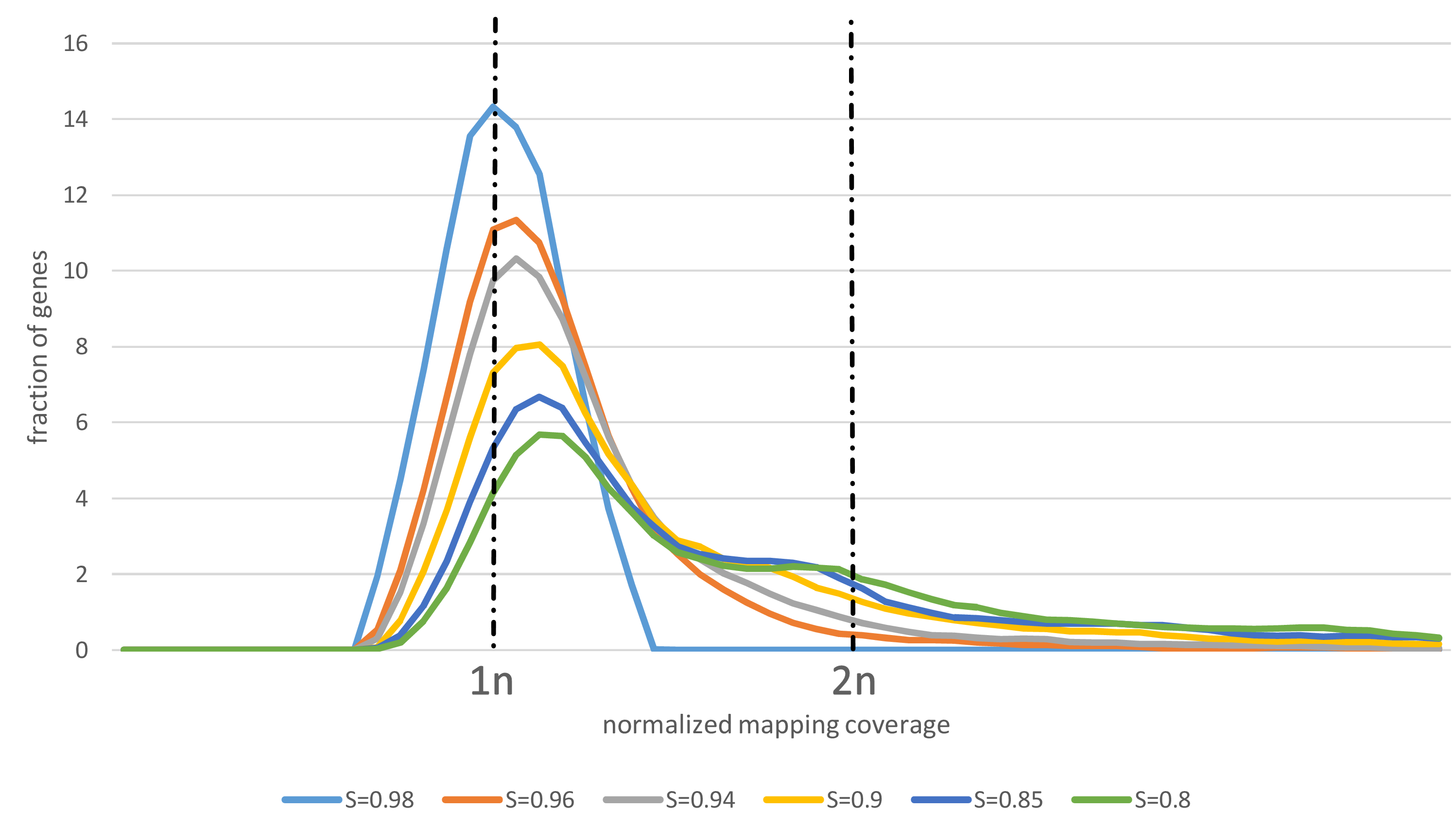


**Fig. S51**. **Effect of decreasing mapping stringency on the read mapping profiles of 4,896 selected genes from the *Lola* mg10 assembly.** The graphs depict the coverage, normalized on the expected haploid genome size, obtained from the mapping of the gills Illumina paired-end library. S = similarity fraction parameter (i.e., minimum allowed % of identify between the read and the reference gene). 1n = one allele found in the genome, i.e., hemizygous state; 2n = 2 alleles found in the genome, i.e., homozygous state.


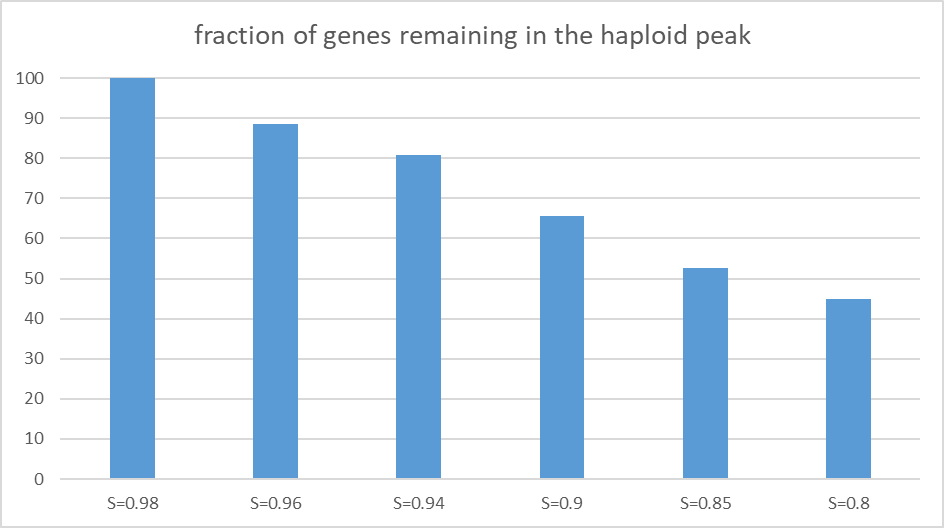


**Fig. S52**. **Fraction of genes maintaining a coverage consistent with the presence of a single allele in the reference genome (i.e., genes found in a hemizygous state)**. That is, remaining within the hemizygous peak, with decreasing mapping stringency (see **Fig. S40**).


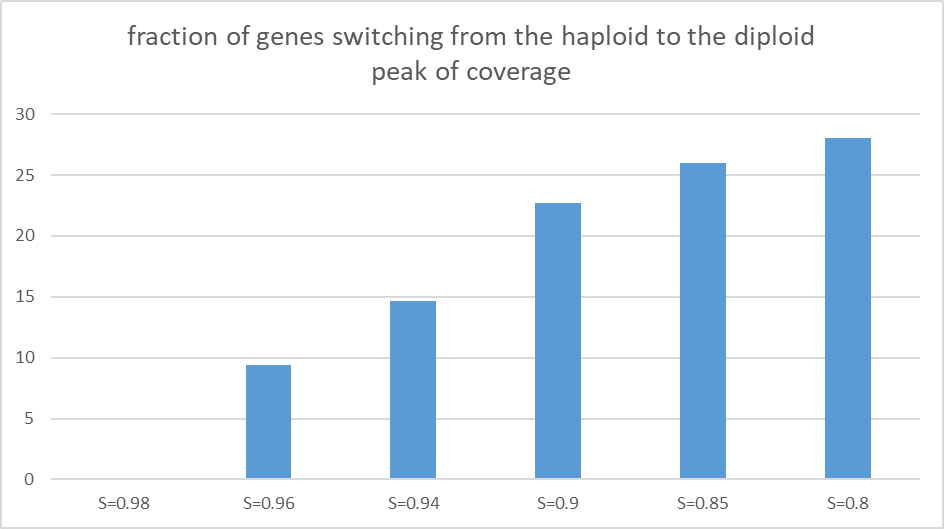


**Fig. S53**. **Fraction of genes that switched from the hemizygous to the homozygous peak of coverage with decreasing mapping stringency** (see **Fig. S40**).


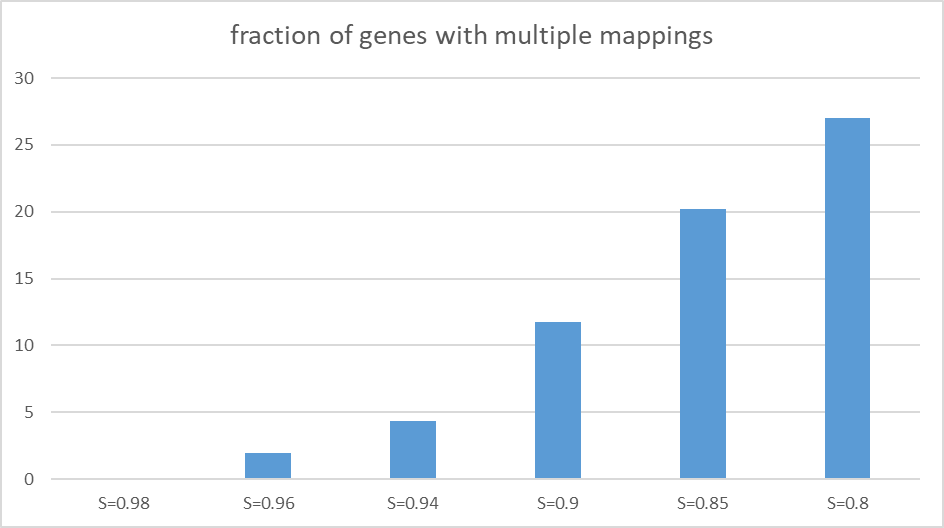


**Fig. S54**. **Fraction of genes that with decreasing mapping stringency acquired multiple mappings.** i.e., showed a normalized read coverage higher than what would have been expected for a single copy gene found in a homozygous state).

## Effects of increasing allelic divergence on the coverage estimates

Next, we simulated *in silico* how read mapping would be affected by the increasing divergence between allelic variants in a randomly generated target genomic region of 10 Kb of length. In detail, PE sequencing reads of fixed length (150 nt) and depth (60X) were generated from the target genomic region, as well as from the homologous region from a virtually generated allelic variant, using an in-house developed script. The allelic variant sequence was generated by specifying an increasing chance of mutation for each nucleotide (from 0 to 0.25, with intervals of 0.01). In summary, different read sets were generated simulating a degree of allelic divergence between 0 and 25%. Reads were generated in random positions to obtain an overall sequencing coverage equal to 60X. Each read set was subsequently mapped to the target sequence with BWA, using the parameters detailed in **Data Note 8** for the identification of *dispensable* genes, and the number of reads mapped was calculated from the obtained BAM files. Ten independent simulations were run for each mutation probability, re-generating allelic variants and reads *de novo* for each simulation.

The aim of this test was to assess what threshold level of divergence between allelic variants could have possibly generated the bimodal distribution of normalized coverage graphs described in detail in **Data Note 8**. Mapping rates were computed and plotted in **Fig. S55**. The results clearly point out that no appreciable drop in coverage is detectable for levels of divergence <10%. Divergence levels as high as 15% roughly caused a 20% decrease in coverage, not yet compatible with the creation of the hemizygous peak of coverage observed in mussel genomes (**Figure 2A** and **Figures S35-S40**). This was, on the other hand, achieved for divergence levels > 20% (with a 40% decrease in coverage), to the point that the complete shift of coverage from the homozygous to the hemizygous peak could be attained for divergence levels close to 25%, which do not seem compatible, on such a large scale, with the expected levels of nucleotide sequence divergence between allelic variants of the same gene in a metazoan species.


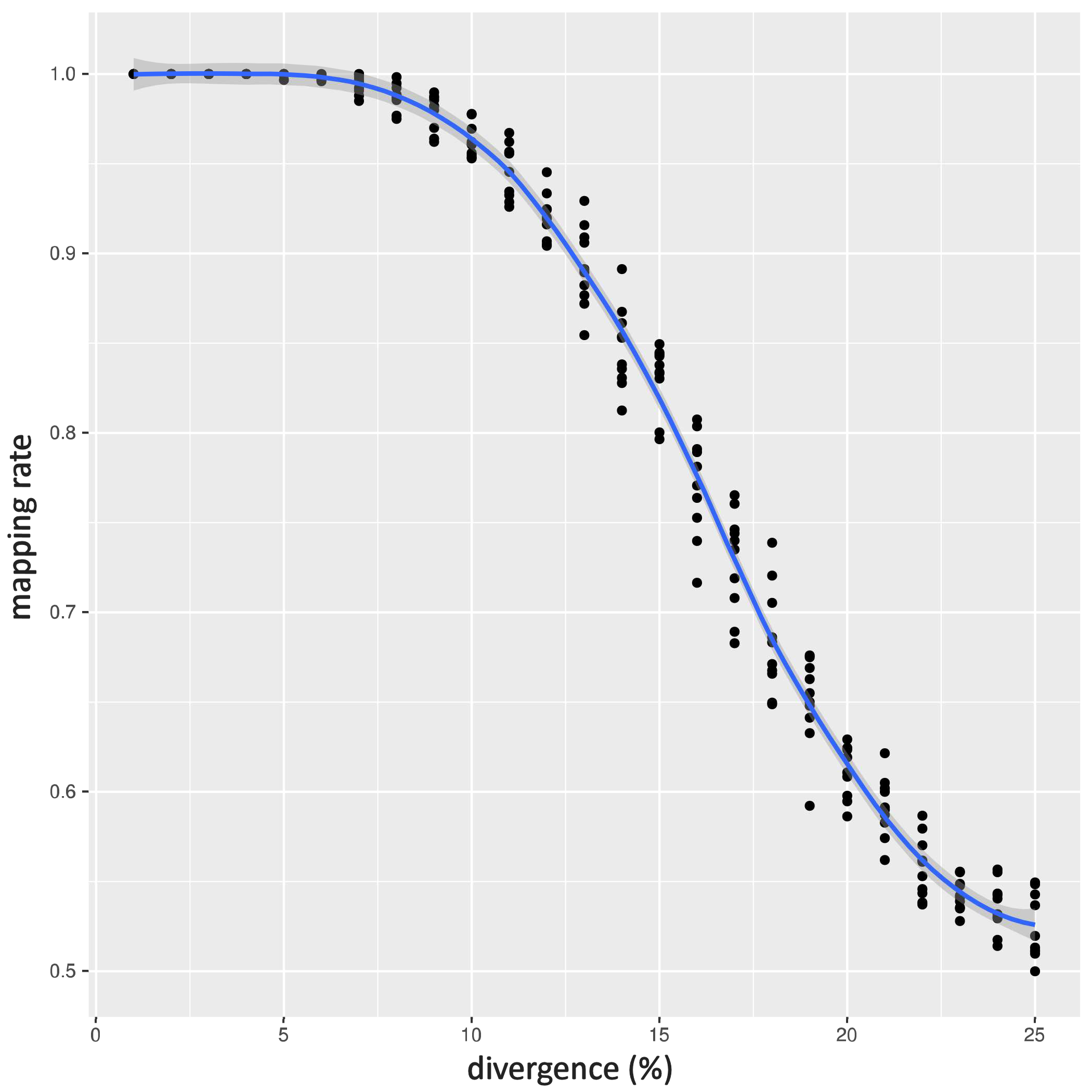


**Fig. S55**. **Results of mapping simulation**. Reads were generated using an in-house developed script from a randomly built genomic region of 10 Kb and from the allelic variant, tolerating increasing amounts of variation between the two sequences (from 0 to 25%). Read sets were mapped to the target region with BWA using the same parameters used for PAV assessment in mussel genomes and the mapping rates were computed for 10 replicates for each step of inter-allelic divergence increase (0.01).

# Data Note 11 – In depth analysis of the sequencing coverage of *core* and *dispensable* genes

## Assessment of *Lola* genes encoded by hemizygous genomic regions

One of the main questions that arises from the observation of a high number of *dispensable* genes is whether any of the genes that in *Lola* appeared to be encoded by hemizygous genomic regions might be subject to PAV in other mussels. The availability of massive sequencing data from the 15 additional genomes (14 resequenced in this study, plus *Pura*) provided a unique opportunity to investigate this issue. In detail, we evaluated the sequencing coverage of two subsets of genes: these were selected based on their localization, well within the hemizygous peak or within the homozygous peak in *Lola* (normalized coverage between 0.75 and 1.25, or between 1.75 and 2.25, respectively) according to the mapping of both the mantle and gills library). First, the large majority of the 12,212 selected genes encoded by hemizygous regions in *Lola* (12,143, i.e., 99.43%) were present in a single allele in at least one of the resequenced genomes, and that 7,084 (58%) were subject to PAV in at least one genome. Consequently, just a tiny portion of such genes (69, 0.57%) were detected as displaying a coverage consistent with the presence of two alleles (in the diploid genome). When individual genomes were considered, we found that a rather uniform percentage of the genes encoded by hemizygous genomic regions in *Lola* (23.23% on average) were absent. More than half of such genes were inferred to be encoded by hemizygous genomic sequence (58.50%) and less than one fifth (18.27%) displayed a coverage consistent with the presence of two alleles (i.e., they were inferred to be encoded by homozygous genomic regions) (**Fig. S56**).


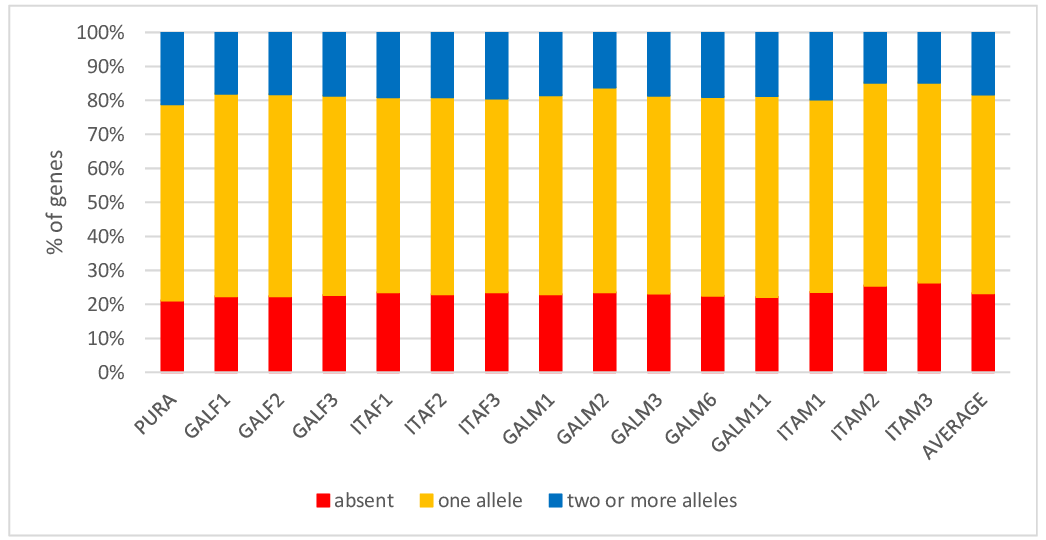


**Fig. S56**. **Summary of the coverage of *Lola* genes present in hemizygous regions in the 14 resequenced mussel genomes (plus *Pura*)**. Genes were categorized as absent (normalized coverage < 0.25), present with one allele (normalized coverage comprised between 0.25 and 1.5) and present with two or more alleles (normalized coverage > 1.5).

## Assessment of *Lola* genes encoded by homozygous genomic regions

A similar analysis was carried out on a subset of 18,470 *Lola* genes displaying homozygous coverage (i.e., falling within the expected peak of mapping), and revealed strikingly different results (**Fig. S57**). Actually, just 8.21% of these were detected as being subject to PAV in at least one of the resequenced genomes, whereas the fraction of genes displaying a coverage compatible with the presence of two alleles in all resequenced genomes was estimated to be in the range of 51.74%, in spite of some complications in the calculations related to the unusual coverages of male mussel genomes (see **Data Note 23**). Overall, the coverage analysis of the genes that displayed homozygous coverage in *Lola* in individual resequenced genomes pointed out their widespread presence with two alleles (85.46% of cases, on average). Such genes were only seldom found in association with hemizygous genomic regions (12.59% of cases, although this number was somewhat boosted by the male mussel outliers), and they were very rarely found to be subject to PAV (e.g. 1.95% of cases).


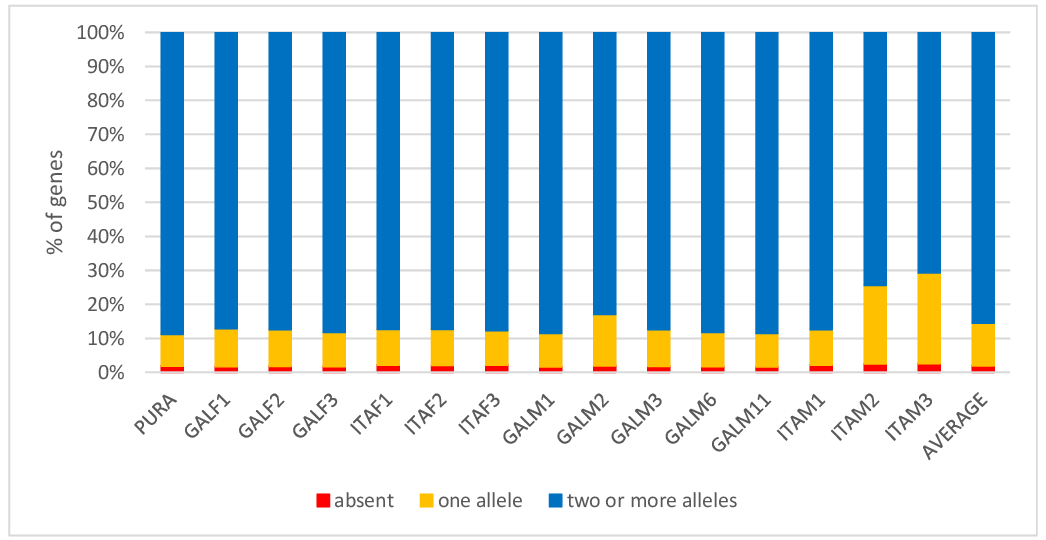


**Fig. S57. Summary of the coverage of a subset of genes present in two copies in *Lola* (i.e., those falling within the omozygous peak of coverage) in the 14 resequenced mussel genomes (plus *Pura*)**. Genes were categorized as absent (normalized coverage < 0.25), present with one allele (normalized coverage comprised between 0.25 and 1.5) and present with two or more alleles (normalized coverage > 1.5).

# Data Note 12 – Validation of Presence-Absence Variation by PCR assays

## Primer design and experimental setup

The PAV phenomenon was confirmed with PCR on 13 mussel genomes: *Lola*, GALF1, GALF2, GALF3, GALM1, GALM2, GALM3, ITAF1, ITAF2, ITAF3, ITAM1, ITAM2 and ITAM3. In detail, the presence-absence of amplification bands, deriving from genomic DNA extracted from the gills and mantle tissues, was assessed on 1% agarose gel (stained with RedSafe, Chembio Ltd., Rickmansworth, UK) for 12 selected *dispensable* gene targets, expected to produce discordant PCR results in case of PAV, and 5 *core* genes (**Table S39**). Primers were designed with Primer3plus, aiming at the amplification of a region of 100-400 nucleotides in length, contained within the same exon, to avoid possible issues related to the inclusion of intronic repetitive sequence which might have prevented optimal amplification. Forward and reverse primers contained, whenever necessary, degenerate bases to enable the pairing even in case of SNPs. These were analyzed, on a case-by-case basis, with an *in silico* analysis (i.e., reads were mapped to the target genomic regions and SNPs were called).

The PCR reaction mix contained 0.5 μl of gDNA (previously diluted to an 80 ng/μl concentration), 6 μl DreamTaq master mix (Thermo Fisher Scientific), 6 μl primer mix (10 μM) and 5 μ ultrapure water. The following PCR conditions were used:

Initial- denaturation: 95ºC - 5 min. followed by 30 cycles of

95ºC – 30s

55ºC – 30s

72ºC – 30s

And a final elongation step at 72ºC for 7min

For this evaluation we selected five *core* genes predicted to be present with high confidence in all resequenced genomes (the observed normalized coverage was close to 2). Some of the *dispensable* genes tested were chosen based on their high (i.e., mytilectin-2 and STING-1) or low (e.g., myticalin B1 and homeobox) frequency of occurrence. One target *dispensable* gene was also chosen based on its exclusive presence in *Lola* (i.e., NOD-like receptor). Mytilin K was selected due to its absence in *Lola*. EF1a-bis, a *dispensable* non-coding gene paralogous to EF1a, was additionally selected. Finally, the three E3 ubiquitin ligase genes were chosen due to their association within the same genomic scaffold, as a part of a PAV block (see **Data Note 17**). Note that, based on the strict decontamination process described in **Data Note 1.2.6**, all the *dispensable* genes selected for this analysis are highly unlikely to derive from exogenous contamination. Similarly, the pan-genomic contig including the mytilin K gene passed all the filtering steps included in the recursive pan-genome reassembly process described in **Data Note 14.3**. This observation, together with the taxonomically-restricted nature of the mytilin gene family (see **Data Note 20**), also allow us to exclude the possibility that mytilin K is a product of exogenous contamination.

**Table S39.** List of primers designed to assess the PAV hypothesis in mussels.

| **Oligo name** | **Forward primer (5' to 3')** | **Reverse primer (5' to 3')** | **Amplicon size** |
| --- | --- | --- | --- |
| ***Dispensable* genes** | | | |
| Myticalin B1 | GACATCGACAATGGGGACTT | GTAACGCGCGTCCATATGAT | 195 bp |
| Mytilin K | TTCCTGCAGACAGGTTGCTA | TTCAGTGGCACAACGTTCAC | 124 bp |
| Mytilectin-2 | TCCATCCCTACATGGGACAT | GGGTGYATTATTTTGCCACT | 151 bp |
| STING1 | HGCTGCCTCARAACAATGYA | TCAGTGCTTCYGGAATGTGY | 176 bp |
| NOD-like receptor | TCTCCATTGTTTGCAGCTTG | CAGTGGTGAATCTCCGACCT | 207 bp |
| Homeobox | AGCCTGGCACAACAGCTAAT | ATGCGAGTCTTGGTCCGATA | 234 bp |
| Acth receptor | GCCGATGGTTAACGGAACTA | GAACTGTCCCTGCTTCTTGC | 227 bp |
| ecto-5'-nucleotidase | GAGGCGGATGTTTTAATGGA | TTCCATTCTCGCACGTTAAA | 184 bp |
| E3 ubiquitin ligase 1 | TGGTGGGAATGTGTTCAAGA | CACGGACCGAAAGAAATGTT | 221 bp |
| E3 ubiquitin ligase 2 | GTAGCAAGATTTGCCGAAGC | CAGCGGTGGTGATGATATTG | 237 bp |
| E3 ubiquitin ligase 3 | AAACGTTCGTGGGTTTTCAG | TTCGGCTTCTGCAATTCTTT | 325 bp |
| EF1a-bis | GTGGGATACAATCCGAAAGC | TCAAGAGCCTGAAGCAAGGT | 158 bp |
| ***Core* genes** | | | |
| tRNA wybutosine-synt 4 | TTGTGTTCACTGAAGGATGGA | CAGTTTTTCTTGATGCACCATT | 241 bp |
| CD109 | CTTATTCGCTCAGCACAGACA | ATTCAATTTCGAGTCCAGGWA | 150 bp |
| Coiled-coil & C2 dom-cont 2A | ACGACCAAAACCATCTAGGC | TGCTGAACAAATGATGATGAGA | 188 bp |
| Cation-indep mannose-6-P | GAGAGCCATGCCATAGTGGT | GGACAGGCATATCTGGTTGG | 151 bp |
| EF1a | GCTTTTGTHCCAATTTCTGG | ACGGAGAGCTTTGTCTGTRG | 177 bp |

## Results of the PCR validation assays

The results of the validation experiments are presented in **Additional file 2:** **Table S40** and the corresponding agarose gels are shown in **Figures S58**-**S62**. The presence of the five *core* genes was confirmed in all the tested mussel genomes, including the reference *Lola* genome. Despite the occasional presence as faint bands (see details below), the *dispensable* nature of the 12 genes putatively subjected to PAV could be confirmed in 11 out of 12 cases. In summary, a clearly visible band of the expected molecular weight (**Table S39**) could be identified in the genomes of the mussels where presence had been previously predicted *in silico* and, on the other hand, no band was visible in genomes displaying a read coverage close or equal to zero for any given target gene.

In general, a gene was considered as present whenever a clear band of the expected molecular weight could be identified in at least one out of the two gDNAs tested for each individual (extracted either from mantle or from gills). However, as mentioned above, we could observe a variable range of band intensities, which were in some cases barely visible, in particular for the STING1 gene (**Fig. S58-S59**). This observation may depend on the presence of multiple SNPs in the target regions for amplification (e.g., the forward and reverse primers for STING-1 contained three and two degenerate positions, respectively).

Overall, the only case where no concordance was observed between PCR results and *in silico* prediction was the Acth receptor gene. While the amplification of this sequence was only expected to occur in *Lola*, we could obtain an amplification product of the expected size in all genomes. Although primers were specifically designed to avoid the occurrence of undesired cross-amplification based on the genomic information available, at the present time we cannot exclude that the bands observed are the product of non-specific amplification.

An extended version of **Figure 3A** (see main text) in shown in **Fig. S62**.


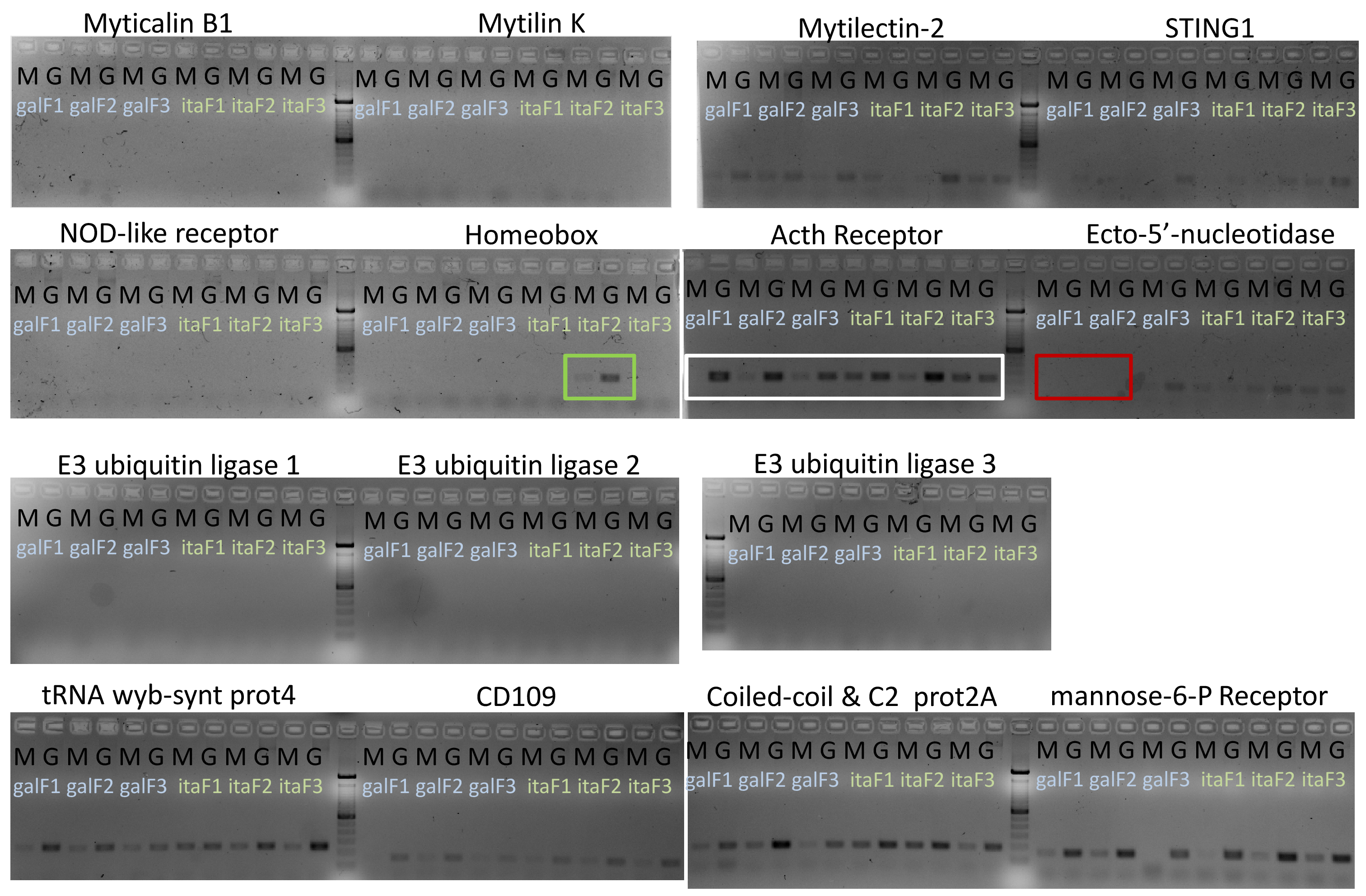


**Fig. S58**. **Confirmation of presence-absence variation by PCR in the 6 tested female mussel individuals**. The predicted outcomes by *in silico* analysis are reported in **Additional file 2:** **Table S40**. The green box highlights the presence of amplification bands for the homeobox gene in ITAF2. The white box indicates the unexpected presence of amplification bands in all female mussels for the Acth receptor gene. The red box indicates the absence of amplification in GALF1 and GALF2 for the ecto-5’ nucleotidase gene. Besides the four *core* genes displayed in the bottom line, also mytilectin-2 and STING1 were amplified in all genomes (although with some faint bands for STING1). No amplification was obtained for myticalin B1, mytilin K, NOD-like receptor and E3 ubiquitin ligase 1, 2 and 3. M: DNA extracted from mantle; G: DNA extracted from gills.


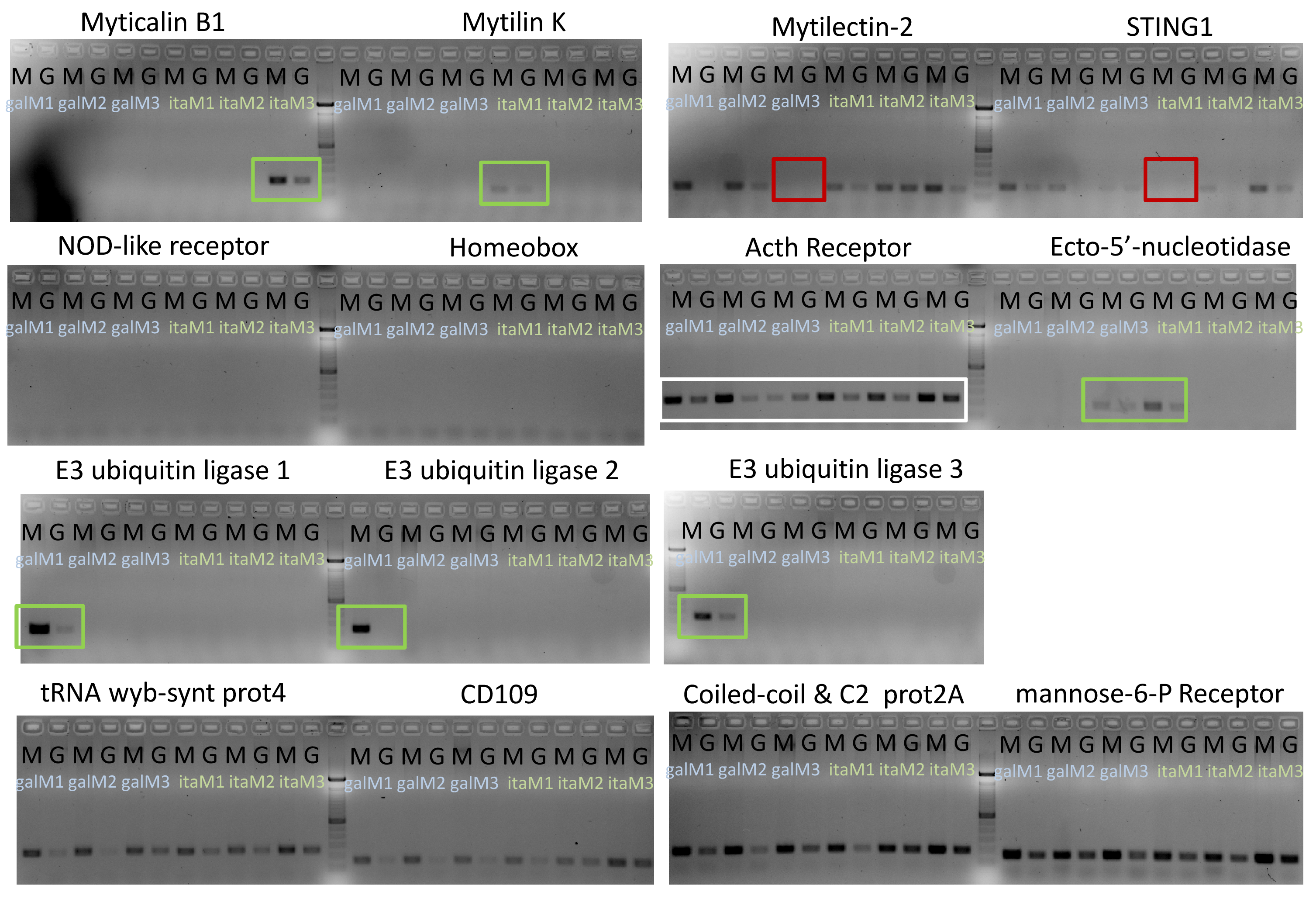


**Fig. S58**. **Confirmation of presence-absence variation by PCR in the 6 tested male mussel individuals**. The predicted outcomes by *in silico* analysis are reported in **Additional file 2:** **Table S40**. The green boxes highlight the presence of amplification bands for the myticalin B1 gene in ITAM3, the mytilin K gene in ITAM1, the ecto-5’ nucleotidase gene in GALM3 and ITAM1, and the E3 ubiquitin ligase genes 1, 2 and 3 in GALM1. The white box indicates the unexpected presence of bands of amplification in all male mussels for the Acth receptor gene. The red boxes indicate the absence of mytilectin-2 in GALM3 and STING-1 in ITAM1. The four *core* genes displayed in the bottom line were amplified in all genomes. No amplification was obtained for NOD-like receptor and homeobox. M: DNA extracted from mantle; G: DNA extracted from gills.


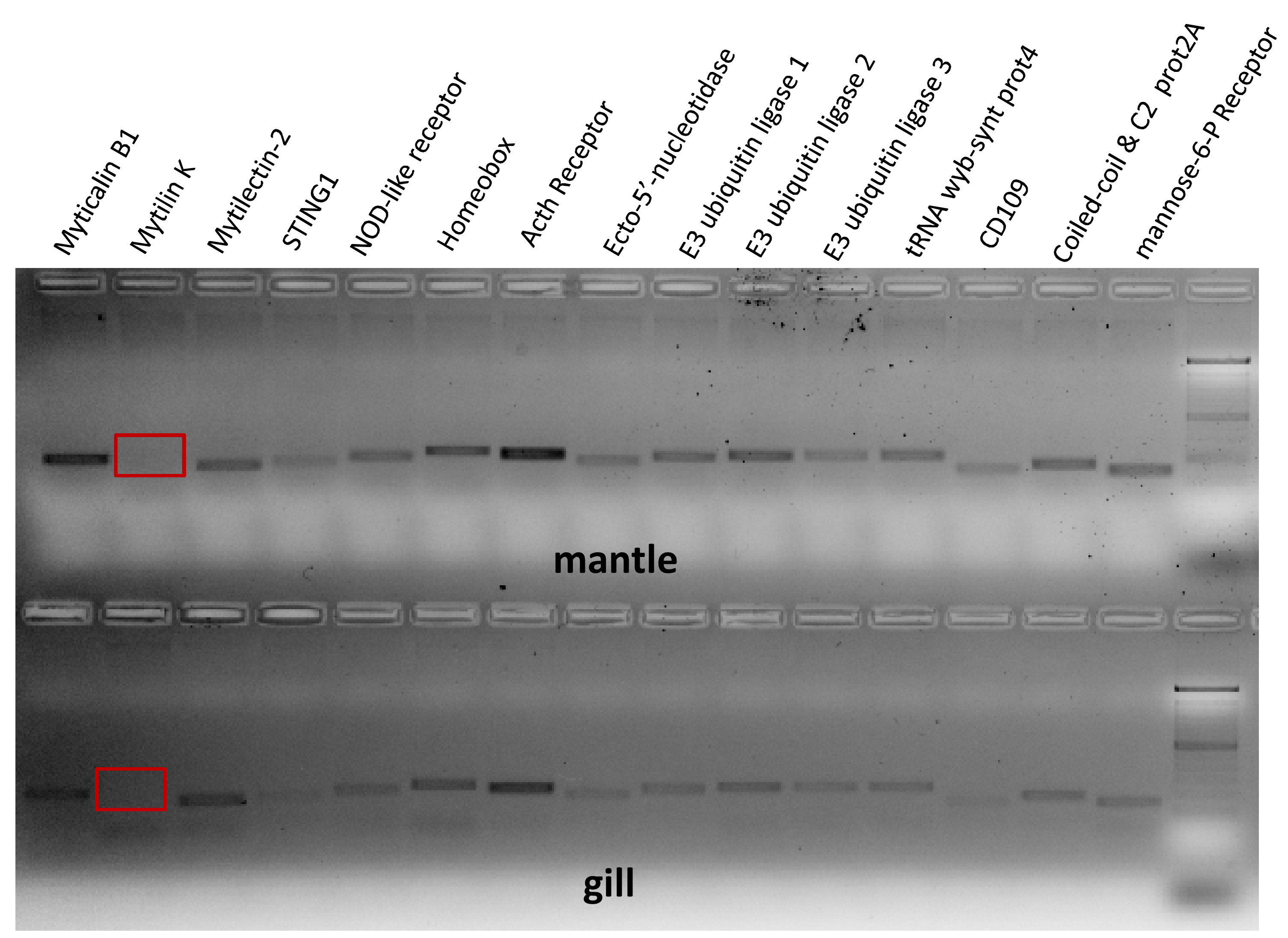


**Fig. S59**. **Confirmation of presence-absence variation by PCR in *Lola*.** Amplification products could be obtained, as expected, in all cases and from the genomic DNA extracted from both tissues, with the exception of mytilin K, a sequence identified from RNA-sequencing data and predicted to be absent in the reference genome.


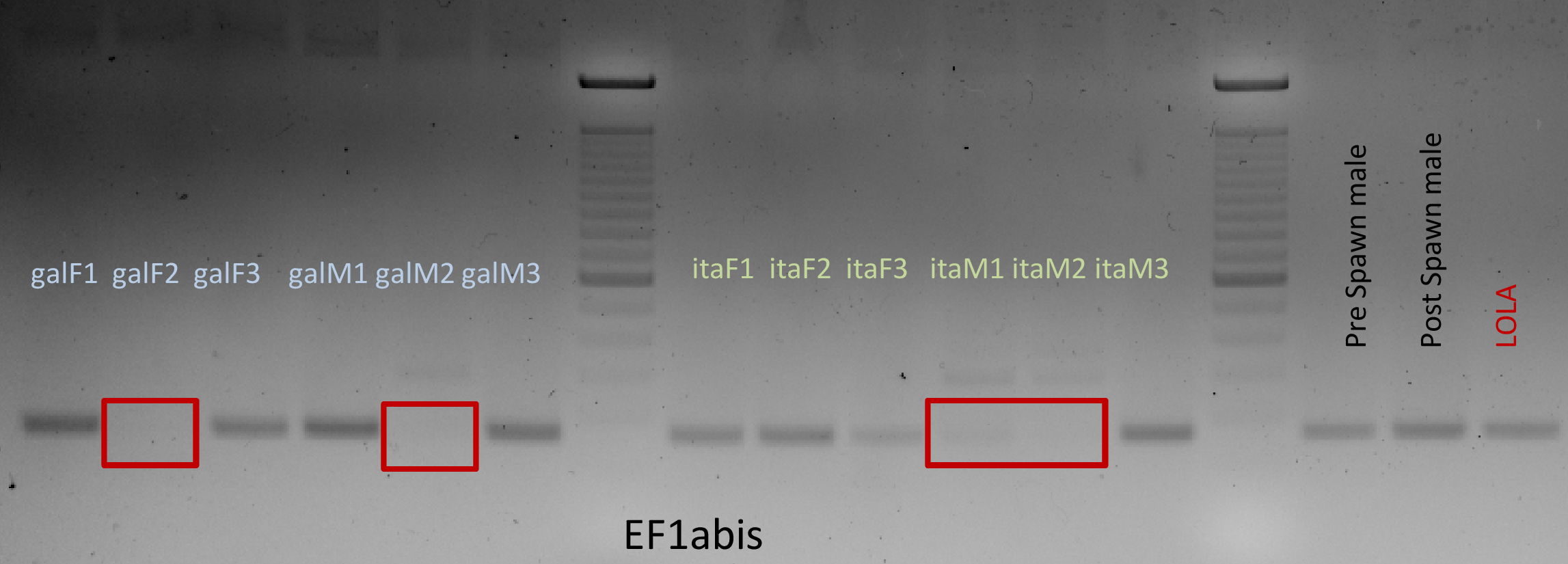


**Fig. S60**. **Confirmation of presence-absence variation by PCR for the elongation factor 1 alpha-bis pseudogene.** The predicted outcomes by *in silico* analysis are reported in **Additional file 2:** **Table S40**. Missed amplification cases are marked by a red box. Pre-spawn and post-spawn male mussels are GALM6 and GALM11, respectively.


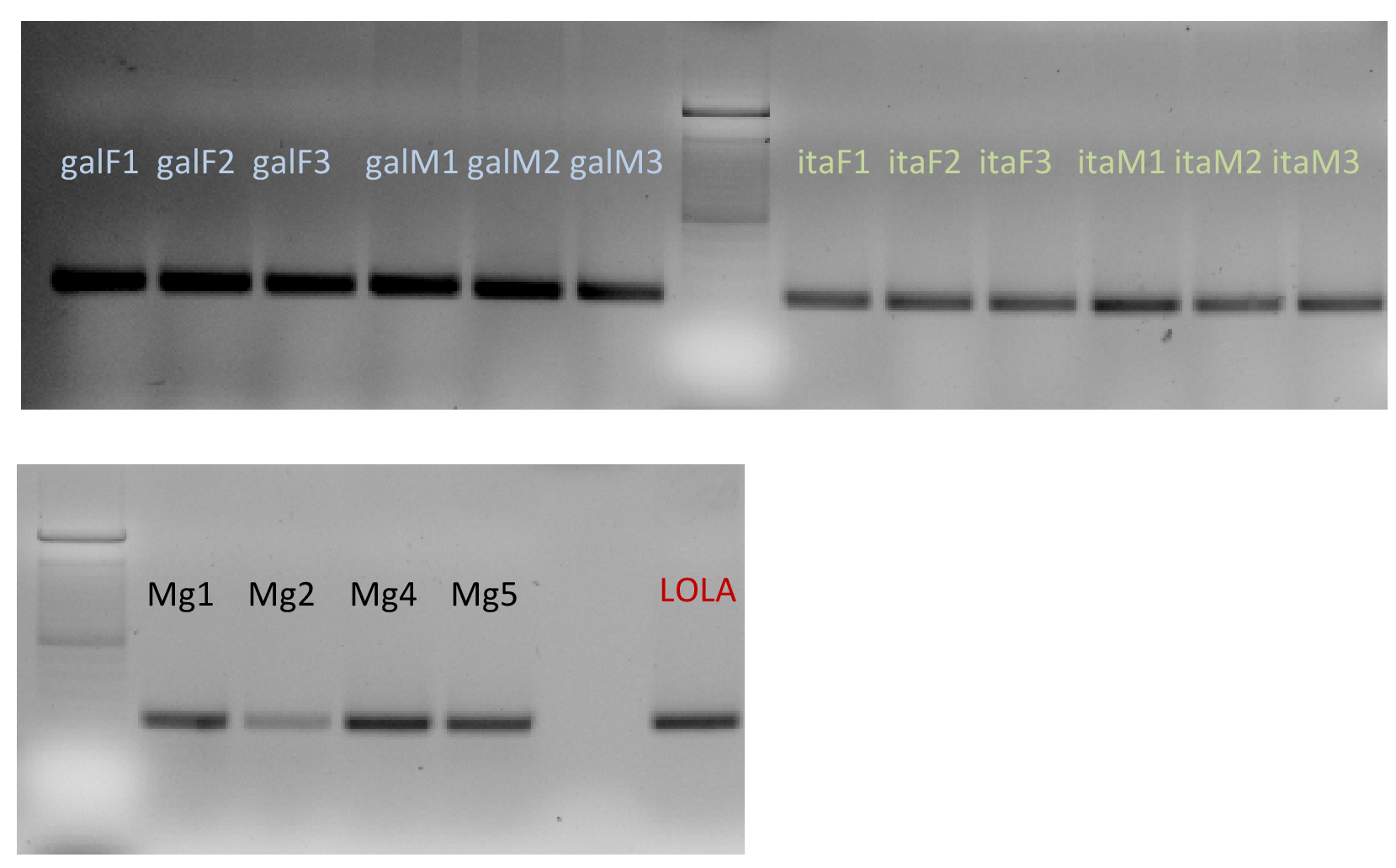


**Fig. S61**. **Confirmation of presence by PCR for the elongation factor 1 alpha- gene**. As expected, an amplification product could be obtained in all cases. Mg1, Mg2, Mg4 and Mg5 represent the genomic DNA extracted from four additional Galician individuals.


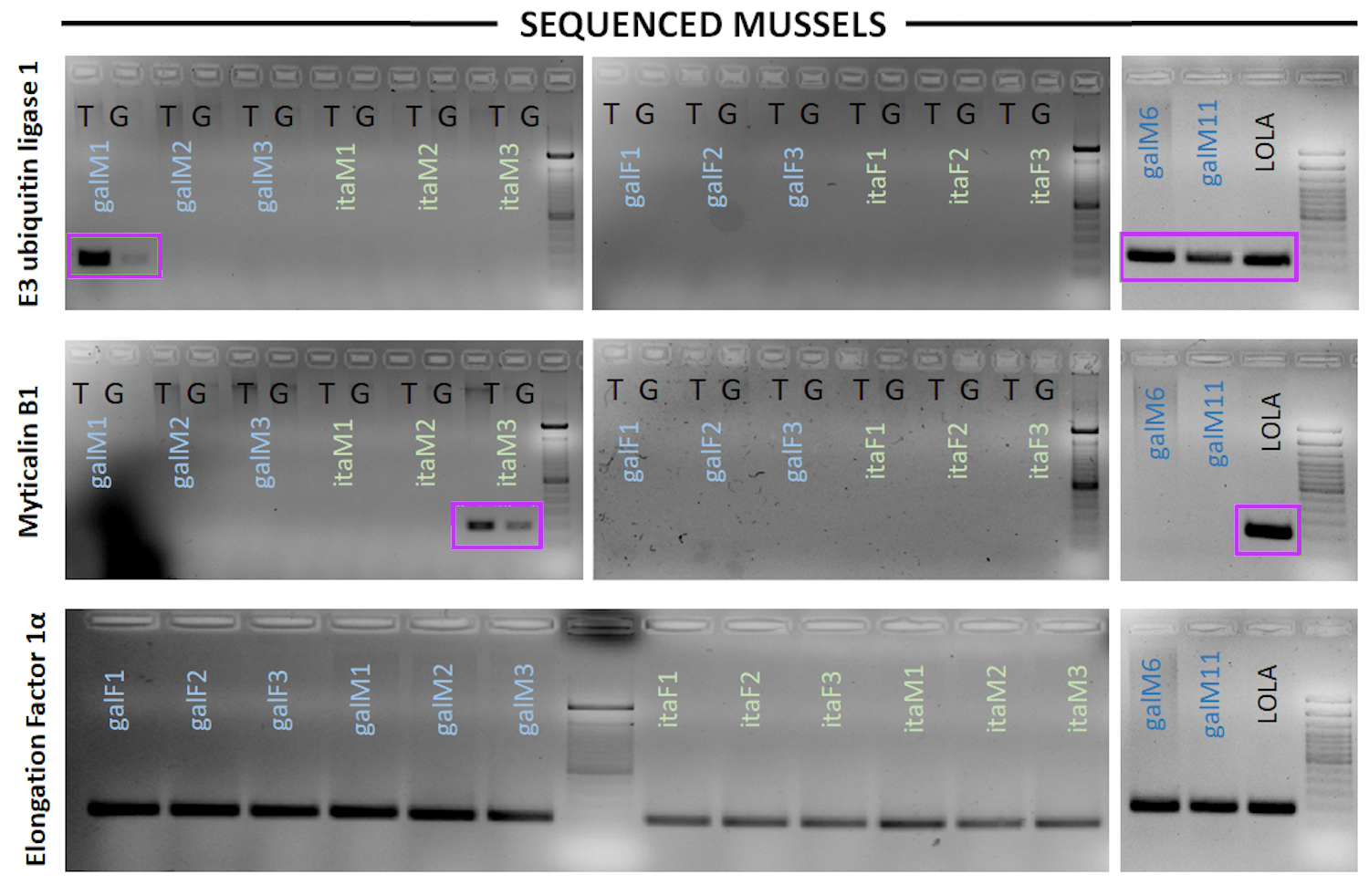


**Fig. S62**. **Validation of the presence-absence variation phenomenon by PCR.** This analysis was carried out on the genomic DNA extracted from the mantle (T) or gills (G) of the 14 mussel individuals subjected to whole genome resequencing. In *Lola*, GALM6 and GALM11, genomic DNA was extracted from the mantle tissue only. One *core* gene (elongation factor 1 alpha) and two *dispensable* genes (E3 ubiquitin ligase 1 and myticalin B1) were tested. To aid the visualization of positive results, samples where the amplification band was interpreted as an evidence of presence are marked with a purple box.

## PCR confirmation in familes of full-sib mussels

To obtain experimental support for the PAV phenomena, three of the tested genes (i.e., elongation factor 1 alpha, myticalin B1 and E3 ubiquitin ligase 1) were also checked in three different families of full-sib mussels, XEC19, XEC20 and XEC21. These families were produced after induced spawning of a single male and a single female per family in the aquaria facilities of the Institute of Marine Research (CSIC, Vigo, Spain). The two parents, as well as three siblings (size <1 cm) of each family were randomly chosen and DNA was extracted from the whole body using a Maxwell 16 LEV robot (Promega, Madison, WI, USA). We could confirm that the PAV phenomenon is present not only between families but also within them as shown in **Figure 3B** in the main text. While the housekeeping *core* gene elongation factor 1 alpha was identified in all individuals, and the *dispensable* gene myticalin B1 was absent in all individuals, the E3 ubiquitin ligase 1 gene displays the following presence-absence pattern:

-XEC 19: present in both parents and two out of three siblings (#33 and #37).

-XEC20: present in the mother and in a single sibling (#22)

-XEC21: present in the father and in a single sibling (#32)

An extended version of **Figure 3B** (see main text) is presented in **Fig. S63**.

**
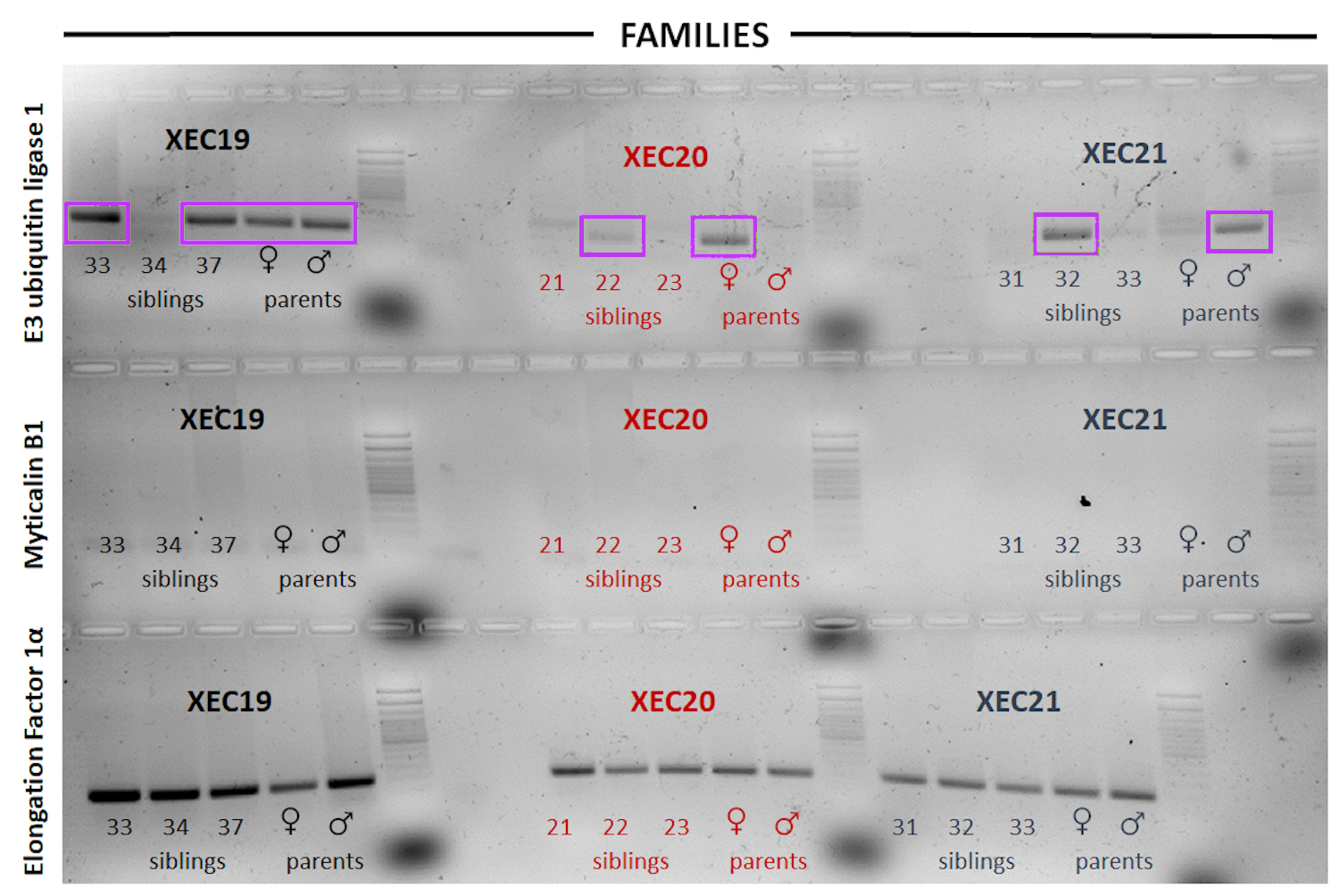
**

**Fig. S63**. **Observation of the presence-absence variation phenomenon by PCR carried out in 3 full-sib mussels obtained from a controlled cross.** Parents were also tested, and their sex is indicated by ♂ and ♀, respectively. One *core* gene (elongation factor 1 alpha) and two *dispensable* genes (E3 ubiquitin ligase 1 and myticalin B1) were tested. To aid the visualization of positive results, samples where the amplification band was interpreted as an evidence of presence are marked with a purple box.

# Data Note 13 – *In silico* validation of PAV with RNA-seq data

## Experimental strategy

An alternative approach was undertaken to confirm the PAV phenomenon in *M. galloprovincialis.* The observation that a high number of the genes annotated in the *Lola* reference genome were inferred to be present in just a few of the other resequenced individuals (see **Data Note 8**) suggests that several *dispensable* genes might be present at low frequencies in mussel populations. Consequently, the mussel “pan-genome” might be significantly larger (see **Data Note 15** and **Data Note 22**), including several *dispensable* genes missing from the *Lola* genome, but present in some of the 14 resequenced genomes (plus *Pura*). The fragmented nature of the *de novo* assembled genomes (see **Table S41**), due to the use of short reads only (Illumina paired-end), prevented a validation approach based on the full re-annotation of these genome re-assemblies and a comprehensive comparative analysis of annotated genes between *Lola, Pura* and the 14 resequenced genomes. To this end, we used a recursive re-assembly strategy which was specifically focused on the genomic regions not included in *Lola*, that enabled to significantly extend the reference genome assembly, by including a large number of “*dispensable* scaffolds”. These were annotated, characterized and analyzed in detail, as reported in **Data Note 14**.

However, we also used an alternative approach based on the analysis of RNA-seq data, which we report here as a further confirmation of the PAV phenomenon. As a matter of fact, assembled RNA-sequencing data offers a good opportunity to obtain information about expressed (and therefore likely to be functional) genes in the absence of a reference genome. Our strategy aimed at recovering the full-length transcripts encoded by *dispensable* genes missing in *Lola* by exploiting publicly available transcriptome data, as described in detail below.

**Table S41.** Statistics of *de novo* assembled resequenced mussel genomes.

| **Genome** | **Number of assembled scaffolds** | **Assembly N50** | **Total assembly size (bp)** | **Longest scaffold (bp)** |
| --- | --- | --- | --- | --- |
| *Pura* | 1,002,335 | 2,931 | 1,500,151,125 | 67,529 |
| GALF1 | 1,207,581 | 2,094 | 1,414,363,425 | 34,056 |
| GALF2 | 1,050,673 | 2,541 | 1,460,633,183 | 41,273 |
| GALF3 | 1,091,538 | 2,286 | 1,415,299,402 | 28,770 |
| GALM1 | 771,555 | 2,891 | 1,381,884,540 | 51,918 |
| GALM2 | 824,596 | 2,703 | 1,397,518,003 | 39,775 |
| GALM3 | 824,141 | 2,657 | 1,383,917,076 | 38,700 |
| GALM6 | 805,159 | 2,738 | 1,379,802,939 | 56,794 |
| GALM11 | 828,758 | 2,551 | 1,345,681,404 | 37,254 |
| ITAF1 | 807,390 | 2,628 | 1,340,353,299 | 46,680 |
| ITAF2 | 816,426 | 2,612 | 1,348,400,053 | 42,999 |
| ITAF3 | 889,380 | 2,612 | 1,471,731,338 | 40,040 |
| ITAM1 | 783,993 | 2,791 | 1,362,999,611 | 59,413 |
| ITAM2 | 834,967 | 2,410 | 1,300,393,004 | 35,402 |
| ITAM3 | 838,011 | 2,395 | 1,288,717,683 | 51,767 |

First, we recovered 17 RNA-seq datasets from the NCBI SRA database (**Table S42**), obtained from different tissues of mussels (*M. galloprovincialis*) sampled from different geographical locations. Upon trimming, NGS data were mapped to the *Lola* genome (mg3 assembly) using the *large gapped mapping* tool included in the CLC Genomics Workbench 11 (Qiagen, Hilden, Germany) and setting both length and similarity fraction parameters to 0.9. In this case, the mg3 assembly was used as a reference instead of the mg10 final assembly to include heterozygous genomic regions which had been discarded during the assembly refinement set (see **Data Note 1**). The use of this specific tool allowed the mapping of “spliced” reads to the reference assembly even in the absence of the annotated corresponding gene and *de facto* allowing the collection of reads that could not be mapped to the reference genome due to insufficient sequence similarity. We expected unmapped reads to be possibly derived from different classes of expressed RNAs:

1. Exogenous contaminants, i.e., microzooplankton and microphytoplankton acquired by filter feeding or present in the residual seawater in contact with soft tissues and inevitably present, at low amounts, in tissue homogenates. This group might also include RNA viruses [129] and transcripts produced by symbiotic or parasitic protozoans and bacteria. In this case, we expected the unmapped reads to generate (upon *de novo* assembly) full-length or partial transcripts with no significant match in any of the 14 resequenced genomes (and *Pura*).
2. Expressed mRNAs corresponding to genes present in *Lola*, but encoded by unassembled genomic regions. In this case, we expected the unmapped reads to generate (upon *de novo* assembly) full-length or partial transcripts whose presence in *Lola* could be easily recognized by the back-mapping of Illumina libraries obtained from *Lola* genomic DNA.
3. Expressed mRNAs with local regions of divergence compared to reference genome. This might be explained by allelic variants, copy number variation, introgression from alleles of other species of the *M. edulis* species complex, and inter-individual and inter-population sequence variation. In this case, we expected the unmapped reads to generate (upon *de novo* assembly), in the large majority of cases, partial fragmented transcripts.
4. Real PAV cases. In this case, we expected the sequence of the *dispensable* gene to be sufficiently divergent from the *Lola* reference genome to avoid the possibility of non-specific cross-mapping, based on the mapping parameters described below. Upon *de novo* assembly, the unmapped reads would be expected to generate either full-length or partial fragmented transcripts, depending on the expression level of the given transcript in the RNA-seq dataset. The assembled full-length transcripts might fall into two categories:
5. Transcripts encoded by genes present in one or more of the resequenced genomes, which could be potentially assessed by the back-mapping of Illumina reads generated from the genomic DNA of each of the 14 resequenced specimens (plus *Pura*).
6. Transcripts encoded by genes absent in all the 14 resequenced genomes (plus *Pura*), i.e., *dispensable* genes present with very low frequency in mussel populations or characteristics of populations distantly related to Galician and Adriatic mussels, and therefore virtually indistinguishable from the case (i) described above.

The analysis was carried out as follows. First, unmapped reads, accounting for 8-19% of the total, depending on the dataset considered (**Table S42**), were collected and *de novo* assembled with the CLC Genomics Workbench 11 assembler (*word size* and *bubble size* parameters were automatically set) allowing a minimum contig length of 300 nucleotides. Protein-coding transcripts were predicted with TransDecoder v.5.01 (<https://github.com/TransDecoder/>), based on a minimum ORF length of 100 codons, and only those marked as “complete”, and thereby likely corresponding to full-length transcripts, were retained for further analysis.

**Table S42**. Summary of RNA-seq datasets mapped against *Lola* mg3 scaffolds.

| **RNA-seq sample ID** | **Tissue** | **No. of reads** | **% of unmapped reads** |
| --- | --- | --- | --- |
| SRR1046119 | gills | 53,334,944 | 12 |
| SRR442031 | digestive gland | 6,573,164 | 15 |
| SRR442032 | digestive gland | 17,752,288 | 15 |
| SRR442033 | digestive gland | 15,768,008 | 14 |
| SRR442034 | digestive gland | 1,694,546 | 19 |
| SRR442035 | digestive gland | 13,304,384 | 19 |
| SRR442036 | digestive gland | 8,907,432 | 11 |
| SRR1045900 | haemolymph | 54,782,140 | 10 |
| SRR1042397 | haemolymph | 52,587,588 | 9 |
| SRR1046117 | muscle | 55,013,474 | 8 |
| SRR1046118 | muscle | 52,113,290 | 9 |
| SRR1046115 | mantle | 5,4102,310 | 11 |
| SRR1046116 | mantle | 51,957,010 | 9 |
| SRR2409049 | mantle | 47,280,786 | 11 |
| SRR2392762 | mantle | 39,799,900 | 11 |
| SRR2392495 | mantle | 48,827,998 | 11 |

Overall, the *de novo* assembly of total 65M unmapped reads obtained from the 17 RNA-seq datasets generated 79,186 contigs (N50= 406 bp; average length=406 bp), that resulted in the prediction of 24,181 ORFs. Out of these, only 1,729 ORFs were marked as “complete” and further characterized. This step permitted to remove transcript sequences likely originated by the case (iii) described above or otherwise poorly expressed.

To exclude “case (ii) sequences”, i.e., the possibility that unmapped reads pertain to genomic regions present in *Lola*, but unassembled, the *Lola* Illumina paired-end sequencing library obtained from gills genomic DNA was mapped to the 1,729 complete ORF sequences, using the following mapping parameters: *length fraction* 0.5, *similarity fraction* 0.98. In this case we applied a somewhat relaxed *length fraction* mapping parameter to allow the mapping of reads originated from intron/exon junctions, due to the lack of intron information in RNA-seq-derived NGS data. All ORFs displaying a coverage compatible with the presence of at least one allele in *Lola* (i.e., those with a coverage higher than 9.5%, meaning those with a normalized coverage on the expected hemizygous coverage peak higher than 0.25) were discarded. The final set that was subjected to analysis comprised 1,286 full-length ORFs, either pertaining to “case (i)” (i.e., exogenous contaminants) or to “case (iv)” (i.e., real PAV cases) sequences. Trimmed reads obtained from the 14 resequenced genomes (plus *Pura*) were similarly mapped to these target sequences using the same parameters outlined above (*length fraction* 0.5. *similarity fraction* 0.98) and the resulting coverages were normalized on the expected hemizygous coverage peak of each genome to infer “absent genes” (normalized average coverage < 0.25) and “present genes” (average coverage >= 0.25).

## Indentification of new PAV cases from RNA-sequencing data

As a result of the mapping, 720 ORFs (56%) were absent in all the resequenced genomes, thereby representing either exogenous contaminants (case (i)) or real *dispensable* “rare” *dispensable* genes (case (iv)b) (**Fig. S54**). All these sequences were not considered for further characterization as a precautionary measure, because the two possibilities could not be discriminated with confidence. The remaining 556 sequences, corresponding to the case (iv)a, can be reasonably considered as the product *of bona fide* *dispensable* genes. In detail, the vast majority of the full-length transcripts identified could be detected in a low number of genomes, either one, two or three in 70% of the cases (**Fig. S64**), indicating that the assembled transcripts were, for the most part, encoded by “rare” *dispensable* genes. A considerable amount of the 720 full-length transcripts of uncertain nature described above (case (iv)b) might be real PAVs as well. This number of sequences present in a higher number of genomes progressively decreased, to the point that no transcript was found to be absent only in *Lola*.


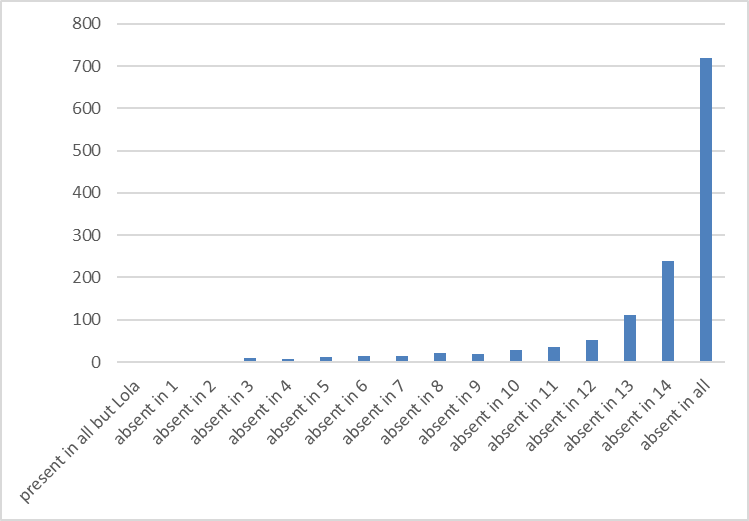


**Fig. S64**. **Summary of presence-absence status of the 1,286 transcripts with a complete ORF (derived from the *de novo* assembly of RNA-seq data and absent in *Lola*) in the 14 resequenced genomes (plus *Pura*)**.

## Characterization of new *dispensable* genes identified in the mussel transcriptome

As a further confirmation of the identification of genuine *dispensable* genes, we performed an annotation of Pfam conserved domains (based on a cut-off E-value = 0.01) of the proteins encoded by the 556 “case (iv)a” sequences. Coherently with the observations gathered from the analysis of *dispensable* mussel genes at whole-genome level (see **Data Note 18**) and at the pan-genome level (see **Data Note 15**), the most abundant protein domains were AIG1, C1q, Scavenger receptor cysteine-rich domain, ankyrin 2, Interferon-inducible GTPase and BIR, i.e., domains which confirm the over-representation of the corresponding domain-containing gene families in the PAV gene set (**Table S43**). For comparison, four of these domains (C1q, AIG1, ankyrin 2 and SRCR) were the most abundant domains also in the full set of full-length transcripts missing in *Lola* (case (i) + case (iv)), further suggesting that a significant number of case (iv)b sequences might exist (**Table S44**).

**Table S43**. Top 12 most abundant Pfam domains in the set of *dispensable* genes (case (iv)a) identified by RNA-seq data analysis.

| **PFAM domain** | **PFAM code** | **Number of sequences identified** |
| --- | --- | --- |
| AIG1 | PF04548 | 29 |
| C1q | PF00386 | 19 |
| SRCR | PF00530 | 18 |
| Ank_2 | PF12796 | 8 |
| IIGP | PF05049 | 6 |
| MIEAP | PF16026 | 6 |
| BIR | PF00653 | 5 |
| Tox-ART-HYD1 | PF15633 | 5 |
| Fibrinogen_C | PF00147 | 4 |
| I-set | PF07679 | 4 |
| Lectin_C | PF00059 | 4 |
| Mab-21 | PF03281 | 4 |

**Table S44**. Top 6 most abundant Pfam domains in the set of genes of uncertain nature (case (i) + case (iv)) identified by RNA-seq data analysis.

| **PFAM domain** | **PFAM code** | **Number of sequences identified** |
| --- | --- | --- |
| Ank_2 | PF12796 | 34 |
| AIG1 | PF04548 | 56 |
| C1q | PF00386 | 35 |
| SRCR | PF00530 | 22 |
| WD40 | PF00400 | 21 |
| Collagen | PF01391 | 11 |

We used the binary presence-absence matrix for the set of the 556 type (iv)a sequences to generate a phylogenetic tree for the genomes of the 14 resequenced individuals (plus *Pura*) with MrBayes v3.2 [127]. The MCMC analysis was run for 300.000 generations, until reaching the convergence of two independent analyses with 4 chains each (evaluated by the reaching of an effective sample size > 200 for all the estimated parameters and average standard deviation of split frequencies < 0.05). The inferred tree topology showed a weak population structure (i.e., the genomes do not tend to group according to their geographical origins) (**Fig. S65**), less evident than that calculated based on the full complement of *dispensable* genes identified in *Lola* and resequenced genomes (**Data Note 22.4**), but much stronger than the structure obtained from the newly annotated genes found in pan-genomic contigs (**Data Note 14**). In particular, the position of *Pura*, GALF1 and GALM2 could not be assessed with certainty, as these genomes were placed in an unresolved node at the base of the clade comprising all the genomes from Adriatic mussels, organized in a star-like fashion, with the exception of ITAM1, which oddly clustered close to GALM6 and GALM11. We might argue that the low number of *dispensable* genes analyzed in this case (556) is not sufficient to provide a reliable reconstruction of the weak geographical correlation of presence-absence patterns that could be detected by the use of the full set of *dispensable* genes.


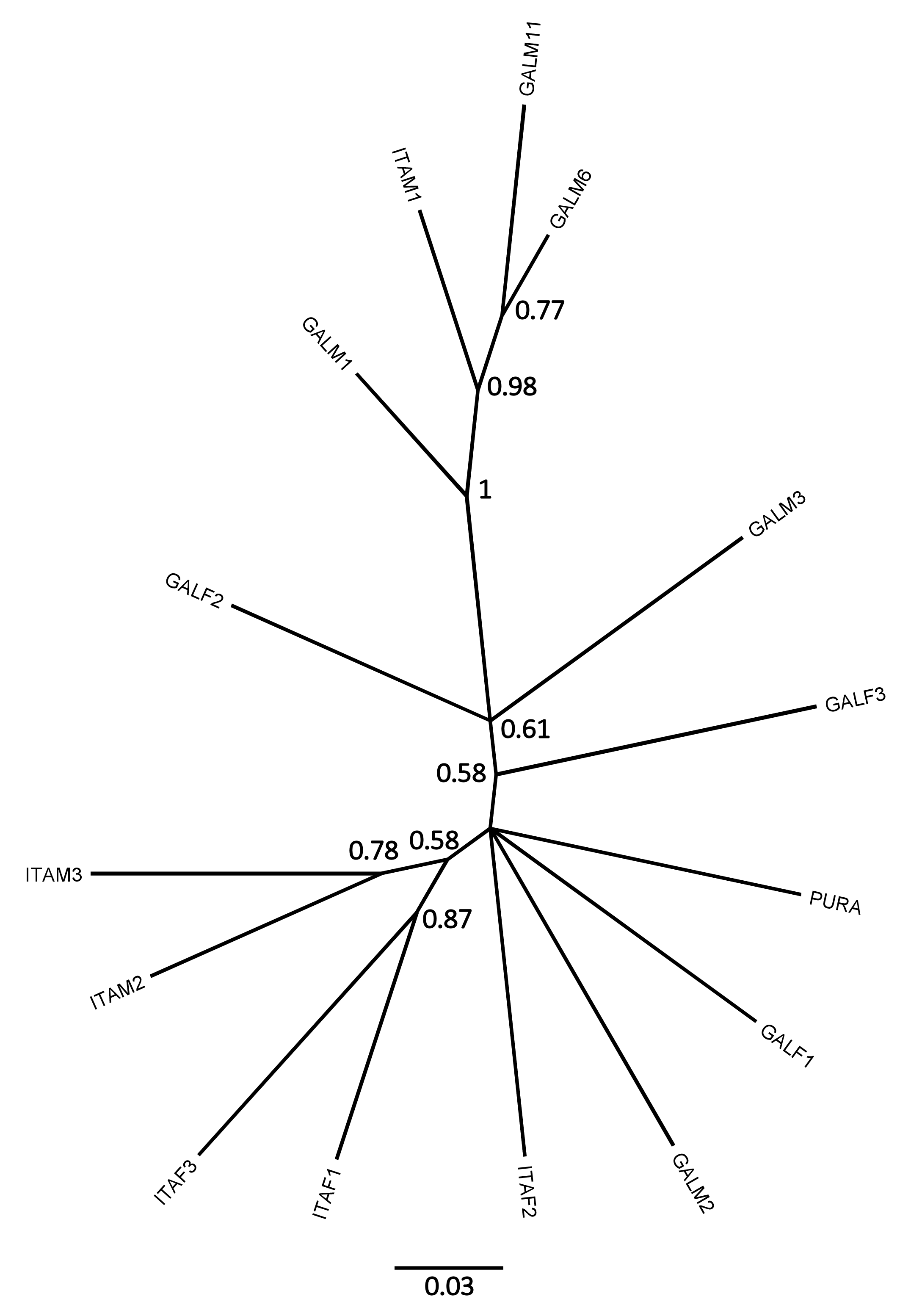


**Fig. S65**. **Bayesian phylogeny of the 14 resequenced mussel genomes (plus *Pura*) based on a binary presence-absence matrix calculated for the 556 *bona fide dispensable* gene sequences identified in RNA-seq datasets.**

# Data Note 14 – Construction of the mussel pan-genome

## Recursive pan-genome reassembly and decontamination strategy

Trimmed sequencing reads for each resequenced genome (see **Table S34**) were mapped against the *Lola* genome assembly, using the mg3 version, as it included all the uncollapsed allelic variants removed in the steps that led to the assembly of the mg10 reference genome (see **Data Note 1**). This mapping strategy was similar to the one described above for the recovery of unmapped RNA-seq data (see **Data Note 13**). The mapping was performed with the *map reads to contigs* tool included in the CLC Genomics Workbench v.20 (Qiagen, Hilden, Germany), setting the length fraction and similarity fraction parameters to 0.75 and 0.9, respectively. Unmapped PE reads were collected and *de novo* assembled with the *de novo assembly* tool, setting the *word size* and *bubble size* parameters to “automatic” and a minimum contig length of 1000 bp.

This process was performed recursively, so that the reads obtained from each genome were mapped against the growing pan-genome sequence dataset, which comprised the *Lola* mg3 scaffolds plus the newly obtained contigs from each new genome analyzed (**Fig. S66**).


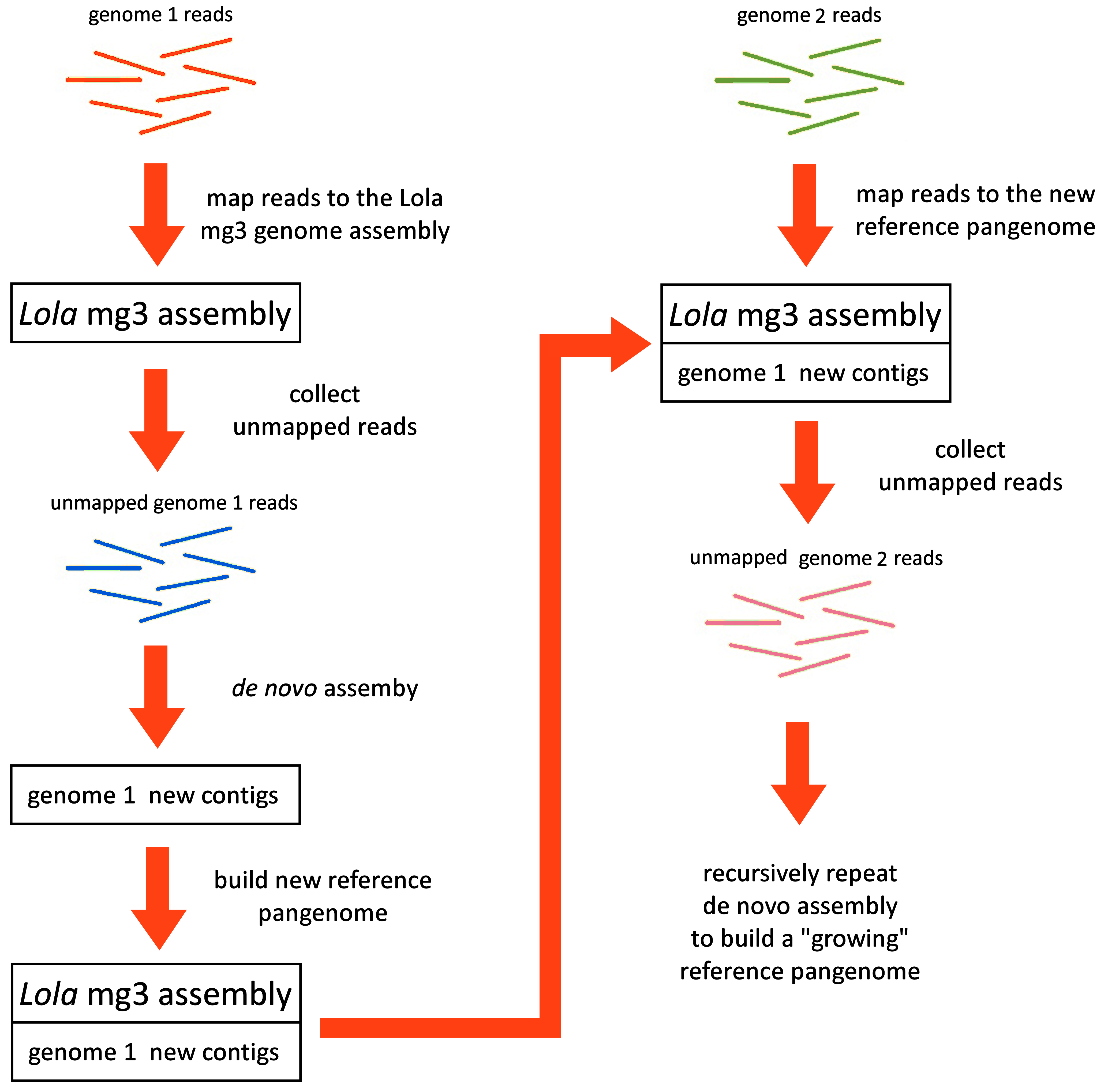


**Fig. S66: Schematic representation of the recursive pan-genome re-assembly process used in this study.**

The genomes were analyzed in the following order: (i) GALF1; (ii) GALF2; (iii) GALF3; (iv) *Pura*; (v) GALM1; (vi) GALM2; (vii) GALM3; (viii) GALM6); (ix) GALM11; (x) ITAF1; (xi) ITAF2; (xii) ITAF3; (xiii) ITAM1; (xiv) ITAM2; (xv) ITAM3. With the exception of GALM1 and ITAM1, the two samples with the most skewed distribution of read coverage (see **Data Note 23**), the reads used were obtained from the sequencing of genomic DNA extracted from the mantle tissue. For GALM1 and ITAM1, reads obtained from the sequencing of genomic DNA extracted from gills were used instead. BAM files with mappings were built for all the assembled contigs.

All the *de novo* assembled contigs underwent a strict filtering process, aimed at removing putative contaminants, which may have either resulted from the sequencing of exogenous genomic material acquired from the marine environment or from user contamination during the phases of library preparation and sequencing, a common issue in the analysis of unmapped data in high throughput sequencing [128]. In detail, the following steps were carried out:

1. All the contigs displaying a particularly low median sequencing coverage (i.e., < 65% of the expected coverage of a *dispensable* gene present with a single allele in any diploid genome, i.e., in a region subject to hemizygosity) were discarded, as they were interpreted as the product of possible low-input exogenous DNA contamination. The sequencing coverage was calculated based on the mapping of the paired-end reads from the genome each contigs was obtained from (e.g., GALF1 reads were mapped to the contigs *de novo* assembled using GALF1 sequencing data). Note that, due to the possible presence of repeats in these regions (which on the other hand were not expected to be found within the exons of *dispensable* genes), the threshold was based on the median, not on the average, coverage.
2. All the contigs displaying a particularly high median sequencing coverage (i.e., > 135% of the expected coverage of a *dispensable* gene present with a single allele in any diploid genome, i.e., in a region subject to hemizygosity) were discarded, as they were interpreted as the product of possible high-input exogenous DNA contamination.
3. All the contigs displaying a GC content falling within the lower and upper 2.5% of the distribution of GC content observed in *Lola*’s scaffolds (i.e., <28,75% or >34,11%) were discarded, as they were considered to be unlikely to derive from *M. galloprovincialis*. GC content was calculated with the EMBOSS *geecee* tool [129].
4. All contigs were screened for the presence of foreign ITS and COI sequences, which might indicate the presence of exogenous contamination. The detected ITS and COI sequences were BLASTed against the NCBI nr database to identify the possible sources of contamination and build a custom contaminant database that was subsequently used to screen all contigs.

In detail, the following sources of contamination were detected, and the following actions were undertaken:

- *Homo sapiens*, possibly derived from operator contamination during animal handling, DNA extraction and library preparation. The most recent release of the human genome (GRCh38.p13) was downloaded from Ensembl and added to the custom contaminant database.

- *Mus musculus*, most likely derived from contamination at the sequencing center occurred during library preparation. The most recent release of the mouse genome (GRCm38.p6) was downloaded from Ensembl and added to the custom contaminant database.

- *Drosophila nasuta*, most likely derived from contamination at the sequencing center occurred during library preparation. The most recent release of the genome [130] (ASM222288v1) was downloaded from NCBI genomes and added to the custom contaminant database.

- An unknown beetle species related with *Popillia japonica*, most likely derived from contamination at the sequencing center occurred during library preparation. The most recent release of the genome (GSC_JBeet_1.0) was downloaded from NCBI genomes and added to the custom contaminant database.

- An unknown fungal ascomycete species related with *Metarhizium anisopliae*, most likely derived from contamination at the sequencing center occurred during library preparation. The most recent release of the genome [131] (MAN_1.0) was downloaded from NCBI genomes and added to the custom contaminant database.

- *Eugymnanthea inquilina*, an hydrozoan species known to sometimes infest *M. galloprovincialis*, living attached to the mantle tissue [132]. Since no genome was available for this species, the genome of the phylogenetically most closely related species available, i.e., *Clytia hemisphaerica*, was selected. This genome was downloaded from the data repository supplied by the authors (web address <http://marimba.obs-vlfr.fr/node/237572> ) [133] and added to the custom contaminant database.

- An unknown cnidarian species closely related with *Montipora capitata*, most likely linked with environmental contamination from larvae present in sea water. The most recent release of the genome [134] (Mcap_UHH_1.1) was downloaded from NCBI genomes and added to the custom contaminant database.

All the contigs passing the filtering steps 1, 2, 3 and 4 were BLASTed against the custom database described above, which also included *Lola* mg3 scaffolds, with a *word size* set to 12 and an e-value threshold of 1E-10. All contigs displaying a non-mussel related best blast hit were flagged as likely contaminants and discarded.

All the contigs that did not display any significant match with *Lola* mg3 were flagged as suspicious and discarded from further analysis. This final step was included in the pipeline as a “precautionary measure” to avoid the inclusion of possible contaminants that might have been missed by the previous steps. This strategy was based on the empirical observation that most *dispensable* genes of *Lola* (and their flanking genomic regions) found significant BLASTn matches against the genome assemblies of the individuals where such *dispensable* genes were absent. Such BLAST hits, uslaly characterized by HSPs with a relatively low homology, but significant e-values, is in line with the observation that genes subject to PAV very often result from massive gene family expansion events (see **Data Note 5**).

1. The novelty of the assembled contigs compared with the genome of *Lola* was checked by re-mapping the reads obtained from the sequencing of the genomic DNA extracted from the gills of *Lola*. This procedure, performed under stringent conditions with the CLC Genomics Workbench v.20 (length fraction = 0.75, similarity fraction = 0.9), ensured the detection of small genomic regions which had not been successfully assembled by the strategy described in detail in **Data Note 1**. The median sequencing coverage resulting from paired-end reads was calculated for all contigs (excluding scaffolded regions, i.e., “N stretches”), and the thresholds used for PAV detection (see **Data Note 8**) were applied to detect and remove contigs representing genomic regions present in *Lola*. In detail, all the contigs achieving a median coverage > 9,25X (i.e., 25% of the expected coverage of a *dispensable* gene present in hemizygous genomic regions>) were discarded.
2. As a final step, to ensure that no residual contamination was present, contigs were classified, using the *k-mer* decomposition strategy of Kraken2 [135], against the whole NCBI nt/nr database, setting a confidence threshold of 0.1, meaning that for taxonomy calling at least 10% of the *k-mers* contained in each contig had to be assigned to a taxonomical rank.

The filtering/decontamination pipeline is detailed in **Fig. S67** below.


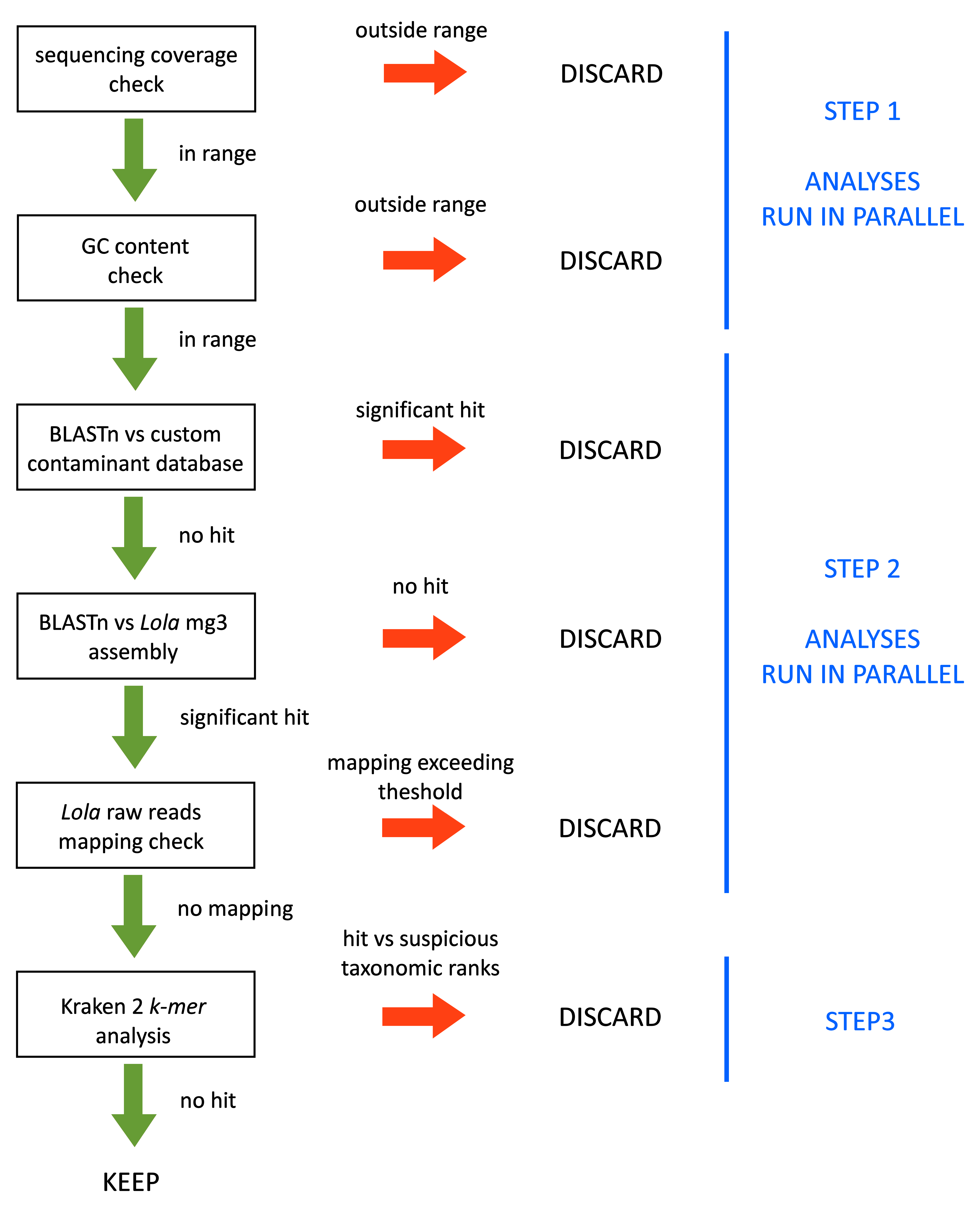


**Fig. S67: Schematic representation of the filtering/decontamination pipeline used to refine the *de novo* re-assembly of mussel pan-genomic contigs.**

## Initial pan-genome assembly results

The recursive pan-genome reassembly process initially led to the generation of 453,284 contigs, with a mean length of 2047.02 base pairs, accounting for a total length of > 900 Mb (927,879,413 bp) (**Table S44**). Due to the recursive reassembly strategy used, these contigs would represent the genomic DNA not present neither in the *Lola* mg10 reference assembly, nor in the previous mg3 version, which included a large fraction of sequence derived from the alternative haplotype of the same individual.

As expected from the progressive inclusion of a lower amount of novel genomic sequence data in the later recursive steps of the process, in general the number of contigs obtained and the total number of newly assembled nucleotides decreased along with the inclusion of the genomes from new individuals (e.g., 70,671 contigs in the first individual analyzed, GALF1, vs 11,926 contigs in the last individual analyzed, ITAM3). On the other hand, the average contig length remained quite stable and close to 2 Kb, indicating that the quality of the assembled contigs was mostly dependent on intrinsic properties of the mussel genome, such as the high repeat content (see **Data Note 2**) and on the sequencing strategy used for these individuals (i.e., only paired-end Illumina reads were available.

Two notable exceptions to this “decreasing” trend were apparent:

1. *Pura*, which showed a lower than expected number of contigs (22,787), a lower than expected assembly size (40,177,497bp), and a lower than expected average contig length (1,763.18 bp).
2. ITAF3, which displayed a much higher than expected number of contigs (54,493), a very large assembly size (147,125,795 bp) and a higher than expected average contig length (2,699.90 bp).

While the metrics obtained from *Pura* could be reasonably explained by the fact that this was the only individual that was subject to a different sequencing strategy (i.e., with shorter Illumina reads due to its inclusion in a previous work [17], with the consequent lower ability to produce the *de novo* assembly of long contigs), the unexpected results obtained for ITAF3 did not find any apparent justification in the sequencing strategy, nor in any particular known feature of this sample.

The factors underlying the large size of the assembly obtained from ITAF3 will be explored in detail in **section 14.5**, where we will demonstrate that this sample was contaminated with DNA from a parasitic hydrozoan.

**Table S44. Initial (pre-decontamination) pan-genome *de novo* recursive reassembly statistics.**

| **individual** | **number of *de novo* assembled contigs*** | **total number of assembled nucleotides** | **average contig length** |
| --- | --- | --- | --- |
| GALF1 | 70,671 | 145,090,250 | 2,053.04 |
| GALF2 | 51,714 | 108,410,851 | 2,096.35 |
| GALF3 | 39,895 | 80,115,443 | 2,008.16 |
| *Pura* | 22,787 | 40,177,497 | 1,763.18 |
| GALM1 | 33,969 | 68,434,718 | 2,014.62 |
| GALM2 | 29,043 | 55,394,375 | 1,907.32 |
| GALM3 | 26,131 | 50,437,478 | 1,930.18 |
| GALM6 | 23,345 | 44,303,835 | 1,897.79 |
| GALM11 | 19,665 | 37,043,143 | 1,883.71 |
| ITAF1 | 21,495 | 41,036,273 | 1,909.11 |
| ITAF2 | 18,271 | 34,483,354 | 1,887.33 |
| ITAF3 | 54,493 | 147,125,795 | 2,699.90 |
| ITAM1 | 16,995 | 31,766,768 | 1,869.18 |
| ITAM2 | 12,884 | 22,944,589 | 1,780.86 |
| ITAM3 | 11,926 | 21,115,044 | 1,770.51 |
| total | 453,284 | 927,879,413 | 2,047.02 |

*based on the recursive reassembly strategy described in detail in section 14.1.

## Decontaminated pan-genome assembly results

The decontamination/filtering process (**Fig. S67**) led to a significant improvement of the quality of the mussel *de novo* recursive pan-genome reassembly, with the removal of a high number of low quality contigs with suspicious origin or derived from exogenous sources. On average, a rather uniform percentage of the contigs present in the original assembly and originated from each individual, i.e., ~35%, were removed (**Fig. S68**). The only outlier was ITAF3 which, as noted above (**Table S44**) displayed an unexpectedly high number of assembled contigs. In this genome, the percentage of discarded contigs reached 82.02%.


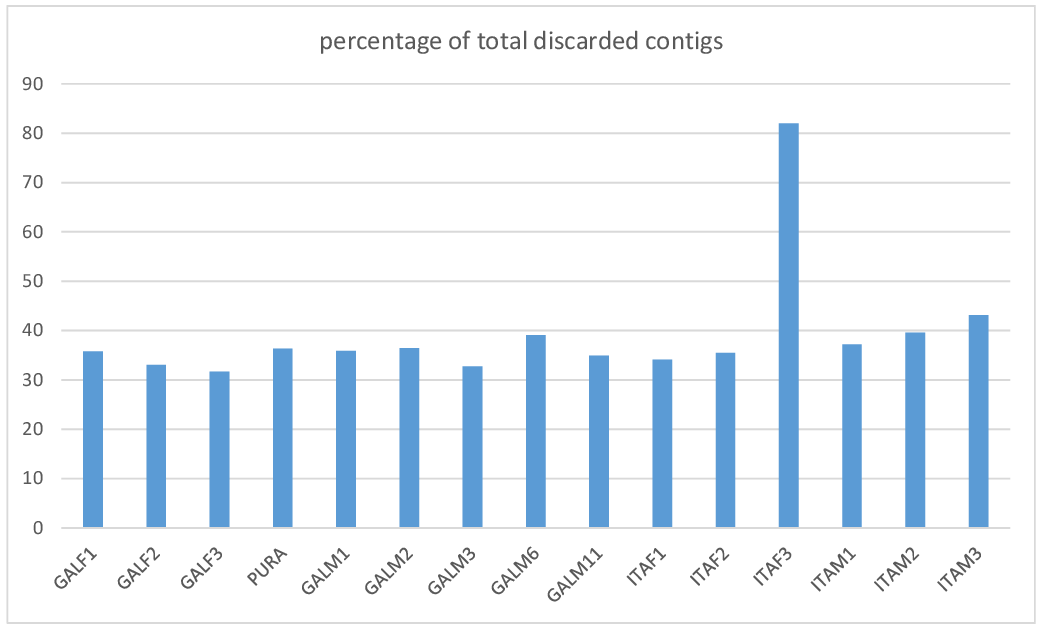


**Fig. S68**. **Fraction of recursively reassembled pan-genomic contigs that were discarded as a result of the decontamination pipeline explained in detail above and summarized in Fig. S67.** A breakdown of the reasons behind their removal is presented in **Figures S72-S76**.

Overall, the total number of contigs retained was 267,538, with an average length of 2,163.21 bp, accounting for a total size of the pan-genome reassembly of nearly 580 Mb (578,741,168 bp) (**Table S45**). Compared with the original unfiltered pan-genome assembly, the decontaminated assembly displayed a much more evident linear progressive decrease both in the number of assembled contigs and size of the assembly, with the complete removal of the issue previously evidenced for the ITAF3 genome. On the other hand, *Pura* continued to display lower than expected numbers, most likely due to the shorter length of the reads generated with the sequencing of this individual in particular. The slight increase in the average contigs size might be explained by the removal of several low quality contigs characterized by low coverage.

The median size of the contigs included in the final version of the reassembly was 1,738 base pairs, indicating that a large fraction of genomic regions characterized by hemizygosity are relatively small and probably do not have protein-coding potential. The longest contig was 25,985 bp, which is in line with the information about structural variants associated with hemizygosity in *Lola* (see **Data Note 17**) and indicates that *dispensable* genomic regions can be potentially quite large (387 contigs exceeded the size of 10 Kb).

It needs to be remarked that the 580 Mb of extra sequence assembled here represent genomic material which was assigned with high confidence to *M. galloprovincialis* (see the detailed discussion below), but which was not present in the reference genome. Based on the observations reported in **Data Notes 8** and **9**, these genomic regions can be considered as *dispensable* and may or may not include protein-coding genes (this aspect will be explored in detail in **Data Note 15**). This approach *de facto* allowed to extend the mussel genome assembly from the reference individual to the 15 additional resequenced individuals, with some technical limitations, mostly linked with the unavailability of long-range sequencing data (i.e., PacBio SMRT reads). Nevertheless, we believe that the metrics of this accessory assembly, in light of the stringent decontamination procedure we used, may provide a reasonable estimate of the true amount of *dispensable* genomic DNA sequence associated with these individuals.

We further need to remark that:

1. Due to technical reasons, we only focused on assembled contigs with >1 Kb size. Hence, smaller genomic regions subject to hemizygosity might have been excluded from the final version of the reassembly.
2. Our filtering/decontamination approach was extremely stringent, both in terms of GC content and median coverage thresholds. Hence, a number of “real” *M. galloprovincialis* contigs might have been discarded due to an excess of caution.

Consequently, we believe that the total size of the pan-genome assembly we present is far from being an overestimate, and it on the contrary most likely represents an underestimate of the actual size of the mussel pan-genome.

In addition, it needs to be highlighted that these estimates were only based on a limited number of individuals from two independent populations. Nevertheless, the recursive reassembly of the 15^th^ individual still led to the generation of >10 Mb additional sequence data. We might expect that the inclusion of additional individuals from other populations would eventually lead to a significant increase of the size of the mussel pan-genome. This particular aspect will be investigated in **Data Note 22**.

Overall, the cumulative pan-genome size dropped, upon decontamination, by 37.63%, while considering the total number of nucleotides excluding the reference genome (**Fig. S69**), and by 12.42%, while including the reference genome (**Fig. S70**). Note the evident “shoulder” in the graph reported in **Fig. S70**, which was linked with the removal of a high number of contigs from ITAF3, consistently with the data reported in **Fig. S68**.

**Table S45. Final (post-decontamination) pan-genome *de novo* assembly statistics.**

| **individual** | **number of *de novo* assembled contigs*** | **total number of assembled nucleotides** | **average contig length** |
| --- | --- | --- | --- |
| GALF1 | 45,368 | 100,589,717 | 2,217.20 |
| GALF2 | 34,618 | 79,357,465 | 2,292.38 |
| GALF3 | 27,247 | 58,944,385 | 2,163.33 |
| *Pura* | 14,511 | 28,061,408 | 1,933.80 |
| GALM1 | 21,774 | 48,641,563 | 2,233.93 |
| GALM2 | 18,450 | 38,874,465 | 2,107.02 |
| GALM3 | 17,583 | 36,943,793 | 2,101.11 |
| GALM6 | 14,227 | 34,109,304 | 2,397.51 |
| GALM11 | 12,768 | 26,525,079 | 2,077.47 |
| ITAF1 | 14,169 | 29,716,845 | 2,097.31 |
| ITAF2 | 11,787 | 24,569,450 | 2,084.45 |
| ITAF3 | 9,797 | 21,078,121 | 2,151.49 |
| ITAM1 | 10,670 | 22,313,713 | 2,091.26 |
| ITAM2 | 7,786 | 15,467,275 | 1,986.55 |
| ITAM3 | 6,783 | 13,548,585 | 1,997.43 |
| total | 267,538 | 578,741,168 | 2,163.21 |

*based on the recursive reassembly strategy described in detail in **section 14.1**.

**
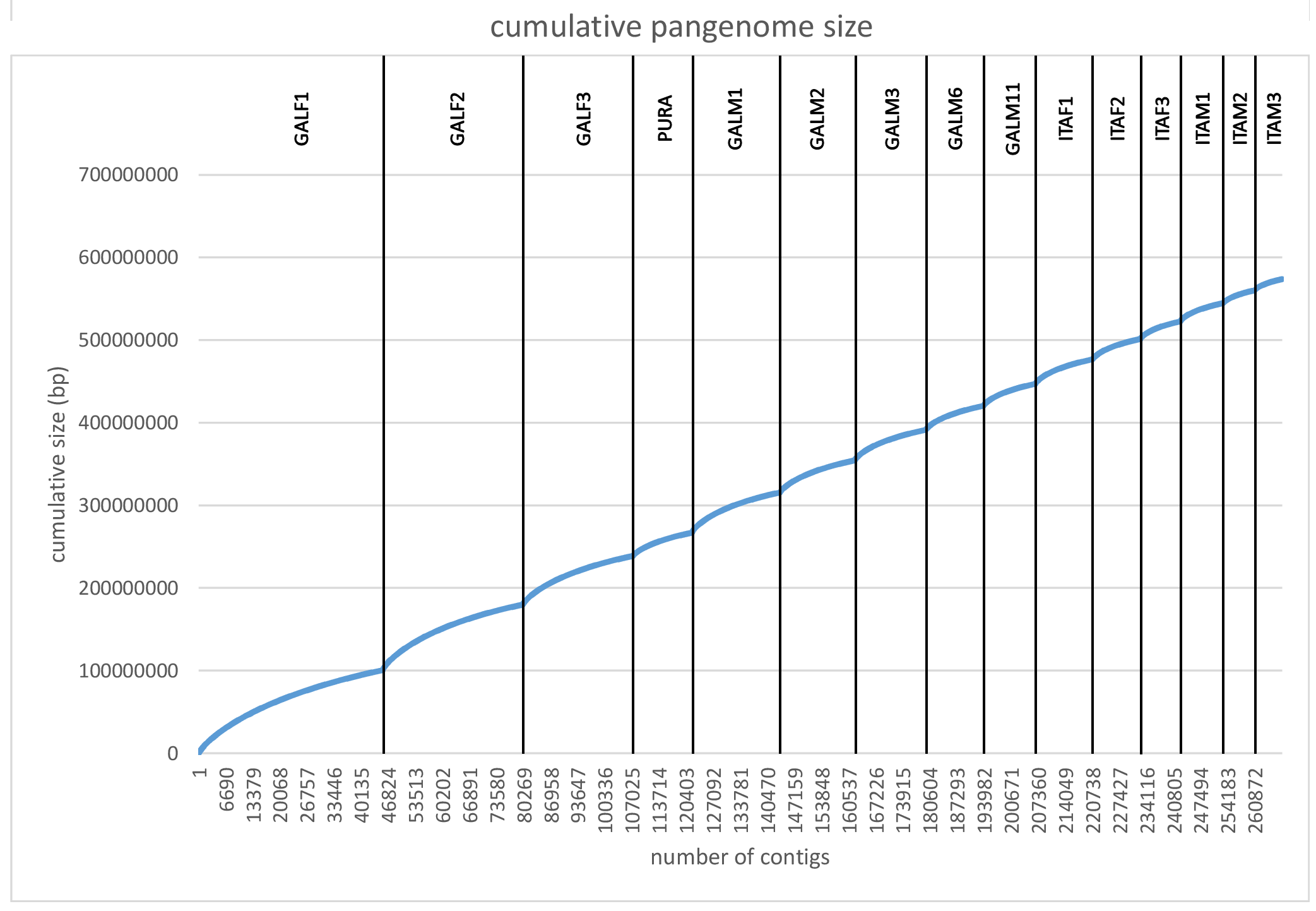
**

**Fig. S69**. **Cumulative size of the mussel pan-genome, based on the pan-genome recursive reassembly process, before and after the filtering/decontamination step.** Note: compared with the following graph, this one excludes *Lola* and also re-orders the accessory contigs obtained from each individual from the longest to the shortest one.


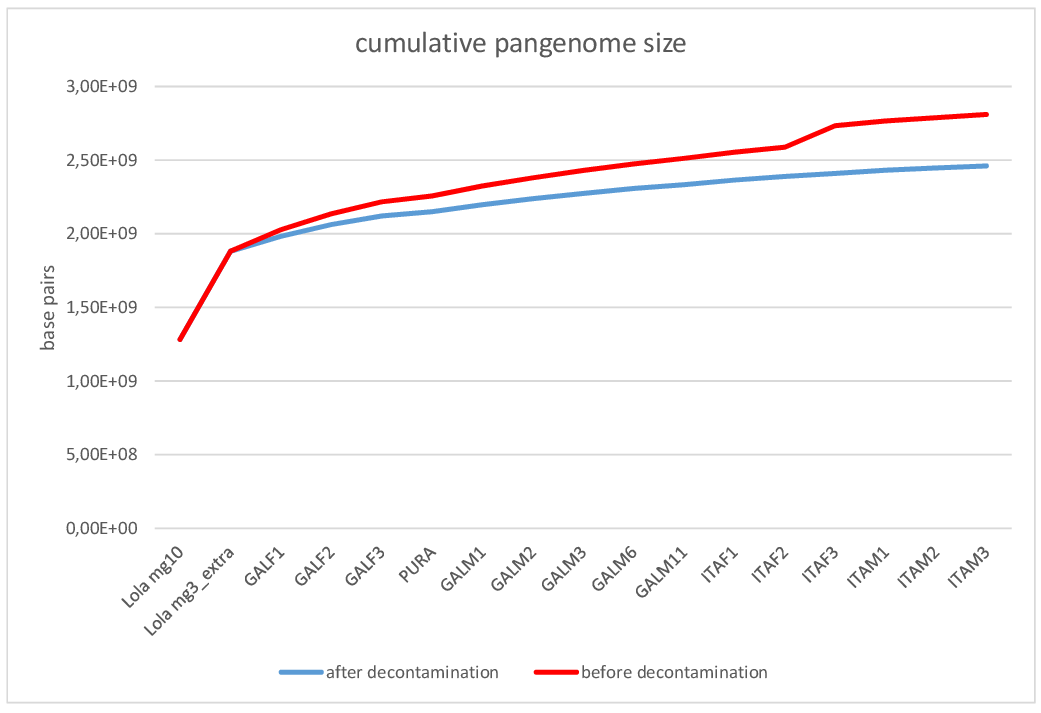


**Fig. S70**. **Cumulative size of the mussel pan-genome, based on the pan-genome recursive reassembly process, before and after the filtering/decontamination step.** Note: this graph also includes the reference mg10 assembly of *Lola*, plus the heterozygous regions assembled in the previous mg3 version and later discarded with the aim to obtain a haploid reference.

The total number of contigs assembled also dropped significantly, from 453,284 to 267,538, which corresponds to a decrease of 40.98% (**Fig. S71**). A remarkable number of the total contigs discarded (i.e., 44,696, 24% of the total) derived from the assembly of ITAF3 sequencing data, as clearly visible from the “shoulder” visible in **Fig. S71** below. This is consistent with the previously evidenced anomalies linked with this sample (see **Table S44**).


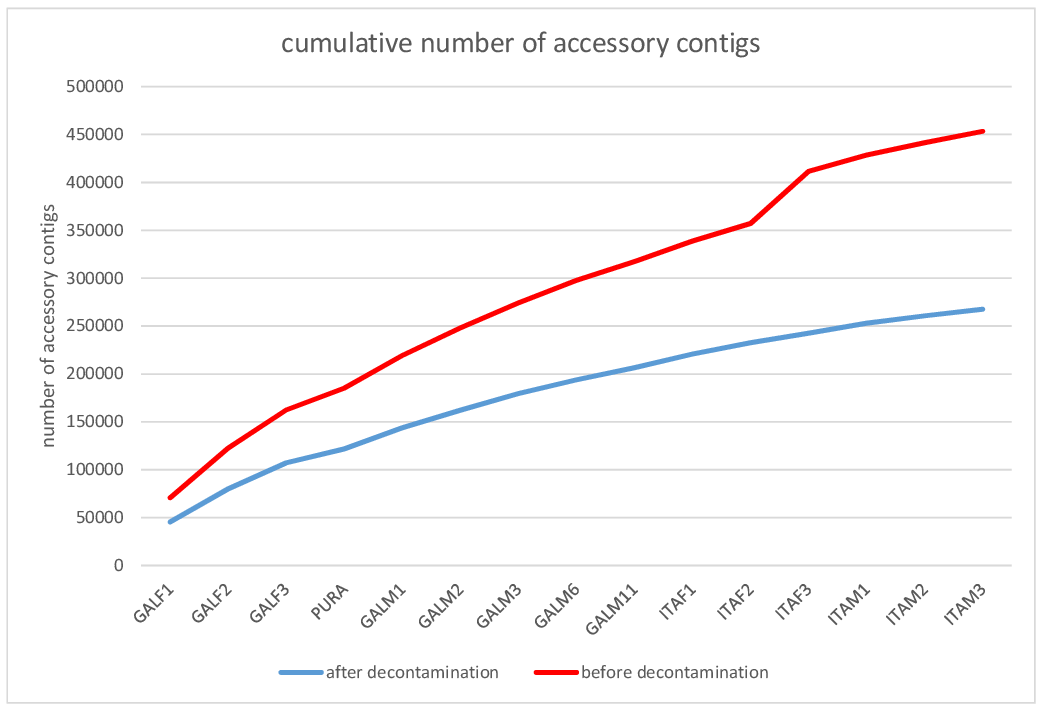


**Fig. S71**. **Cumulative number of accessory contigs included in the mussel pan-genome, based on the pan-genome recursive reassembly data, before and after the filtering/decontamination step.**

## Decontamination detailed report

This section reports the detailed results of the decontamination/filtering process (**Fig. S67**). Please note that, unless otherwise stated, the numbers reported below are not additive, meaning that the contigs flagged as suspicious due to one metric may have been also simultaneously flagged for other reasons (e.g., high GC content AND low sequencing coverage.

On average, 9.17% of the contigs assembled in each individual were flagged as likely contaminants due to low GC content (**Fig. S72**). The different individuals showed relatively uniform results, with *Pura* being the one with the highest proportion of flagged contigs (14.27%) and ITAF3 being the one with the lowest (5.90%).


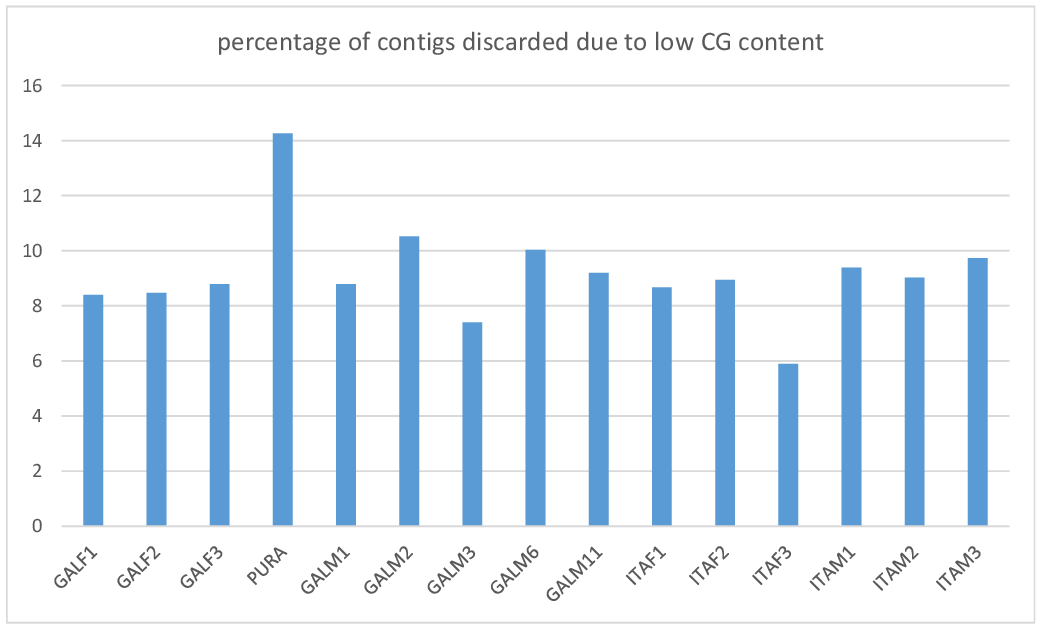


**Fig. S72**. **Fraction of re-assembled pan-genomic contigs flagged as contaminant/suspect due to low GC content.** Note that these numbers are not additive to those reported in the following graphs (e.g. contigs may have been flagged multiple times due to different warnings).

On average, 9.45% of the contigs assembled in each individual were flagged due to high GC content (**Fig. S73**), which was similar to the fraction of contigs flagged due to low GC content (**Fig. S72**). In this case however, distinct individuals were characterized by marked differences: in particular ITAF3 displayed a very high value (15.42%), as opposed to *Pura*, which showed a fraction of flagged contigs lower than the other individuals (6.66%). In general, the data obtained from ITAF3 pointed out a significant bias towards a group of contigs characterized by high GC content, whereas *Pura* displayed a similar bias towards a group of contigs characterized by low GC content.


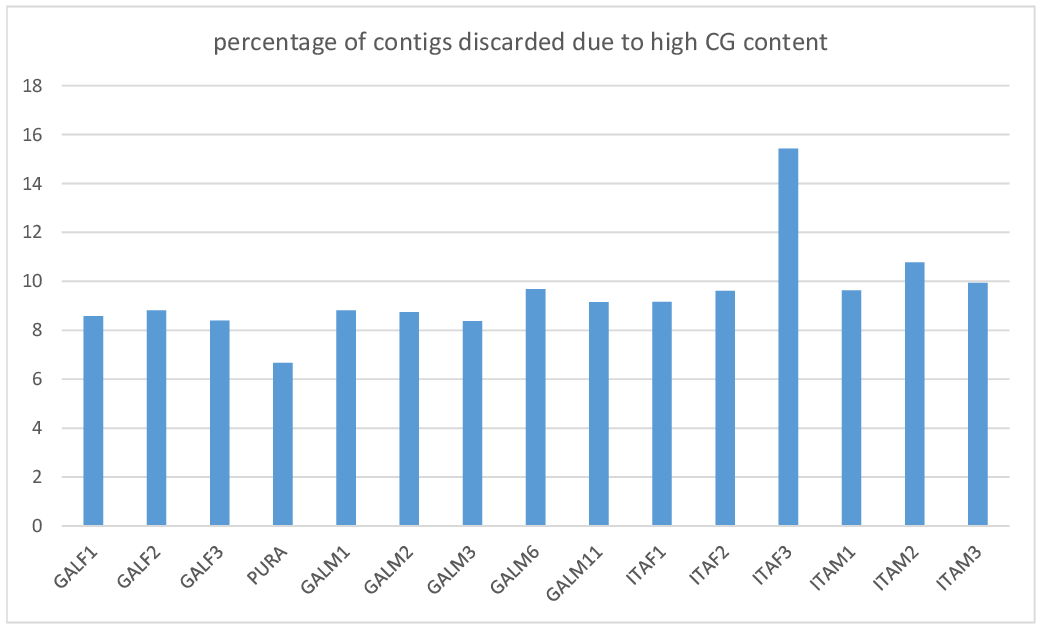


**Fig. S73**. **Fraction of re-assembled pan-genomic contigs flagged as contaminant/suspect due to high GC content.** Note that these numbers are not additive to those reported in the following graphs (e.g. contigs may have been flagged multiple times due to different warnings).

The fraction of contigs flagged due to low median sequencing coverage largely varied from individual to individual (**Fig. S74**). ITAF3 emerged, once again, as an outlier, with 68.04% contigs showing a lower coverage than expectations. This result, combined with the GC content analysis reported above, revealed that the *de novo* reassembly of ITAF3 sequencing data led to the generation of a high number of contigs with a lower coverage and higher GC content than it would have been expected from *M. galloprovincialis*. These contigs were marked as “suspect” and further characterized (see below). *Pura* also included a significant fraction of contigs with low coverage (15.09%), like the two male mussel individuals ITAM2 and ITAM3. This observation can be explained, for these two male genomes, by the highly skewed distribution of read mapping (see **Data Note 23**).

The three female individuals GALF1, GALF2 and GALF3 were on the other hand, the individuals with the lower fraction of flagged contigs (<6%).


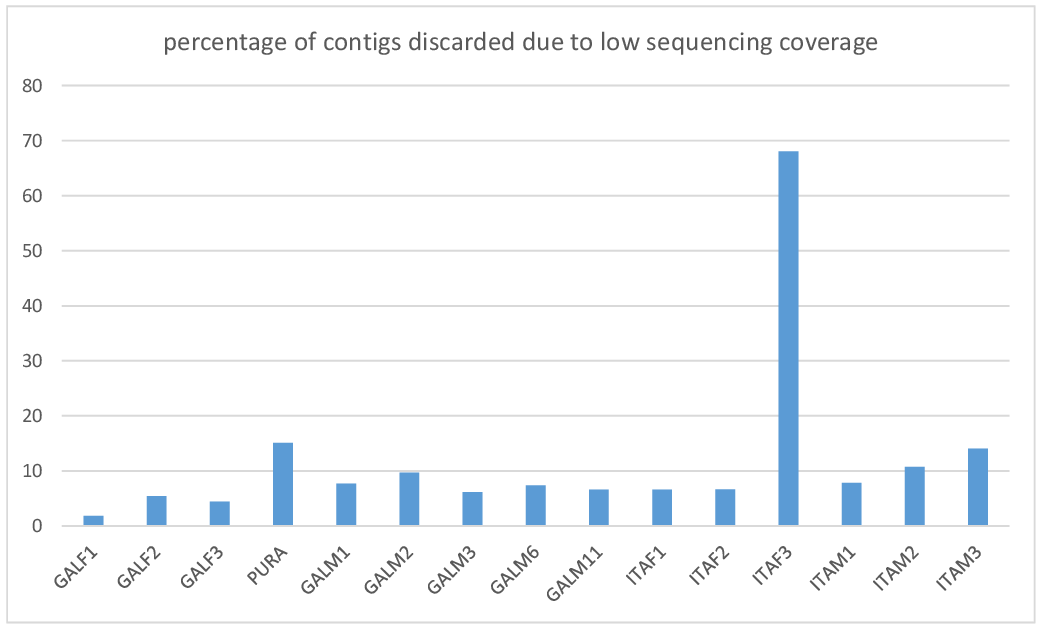


**Fig. S74**. **Fraction of re-assembled pan-genomic contigs flagged as contaminant/suspect due to low sequencing coverage.** Note that these numbers are not additive to those reported in the following graphs (e.g. contigs may have been flagged multiple times due to different warnings).

In stark contrast with the results reported above for the contigs with low coverage, only a very few contigs displayed a median coverage that exceeded the arbitrary upper threshold. In detail, just 0.27% of the assembled contigs per individual were discarded (**Fig. S75**). No contig was flagged as suspect in GALF3 on one extreme, whereas 0.60% contigs were flagged as suspect in *Pura*.


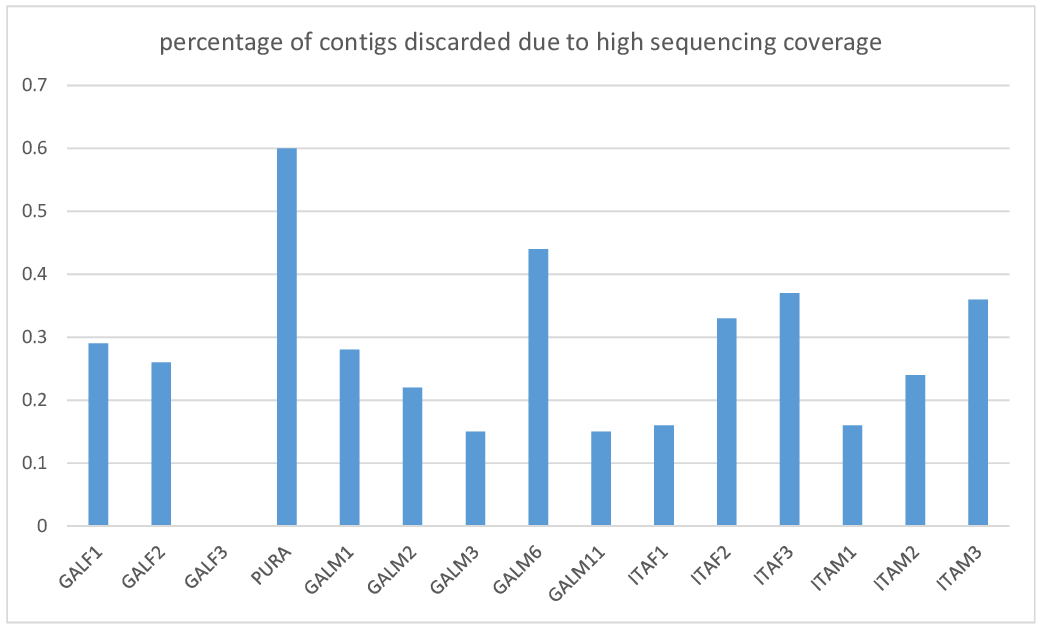


**Fig. S75**. **Fraction of reassembled pan-genomic contigs flagged as contaminant/suspect due to high sequencing coverage.** Note that these numbers are not additive to those reported in the following graphs (e.g. contigs may have been flagged multiple times due to different warnings).

The residual fraction of contigs discarded either due to suspect or inconclusive BLASTn hits or due to their likely presence in unassembled regions of the *Lola* reference genome was quite uniform for all but two genomes, i.e., *Pura* and ITAF3, which, as described above, were the only two to display significant variantions compared with the others in terms of GC content and sequencing coverage (**Fig. S76**). Note that these steps were not performed in parallel with the evaluation of GC content and sequencing coverage, so the numbers of discarded contigs in this particular case are not additive to those reported in the previous graphs (**Fig. S67**). The average fraction of contigs discarded in each individual was 8.93%, but this number was much lower in ITAF3 (i.e., 4.40%) and *Pura* (0.54%), indicating that the previous filtering steps based on GC content and sequencing coverage had already been very effective in the removal of possible contaminants.


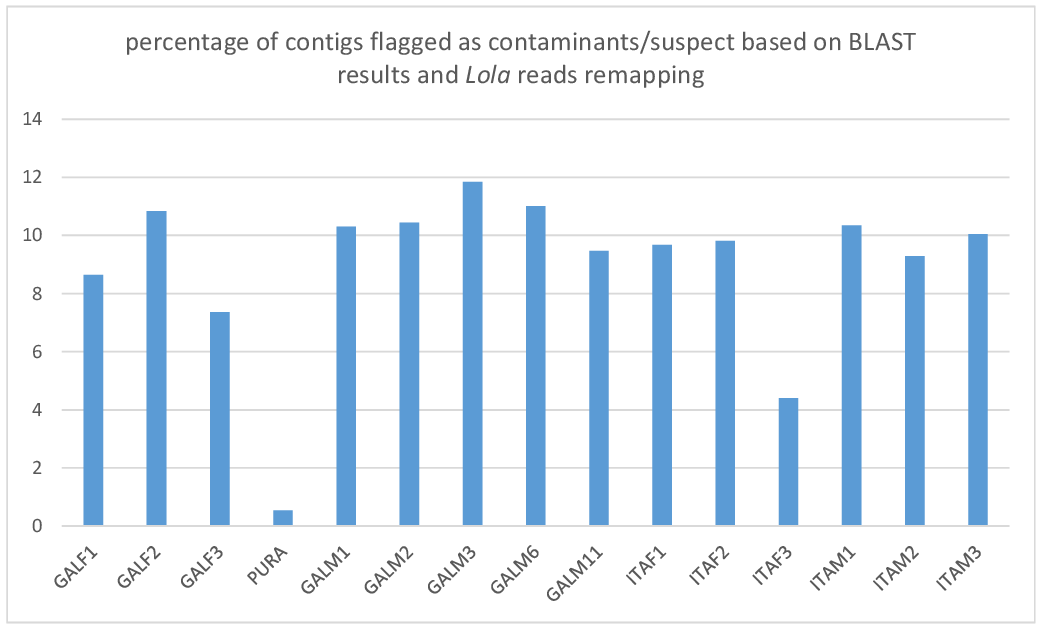


**Fig. S76**. **Fraction of re-assembled pan-genomic contigs flagged as contaminant/suspect either based on BLAST results or on the mapping of reads from the *Lola* genome.** Note that these numbers are additive to those reported in the previous graphs (**Fig. S72– S75**).

The final step of decontamination/filtering was, run with KRAKEN 2, led to the detection of no further “suspect” contigs. Out of the 267,538 contigs analyzed, only 32 could be classified within a known taxonomical rank, 23 of which at the genus level. Of such 23 contigs, 22 were classified within the *Mytilus* genus and 1 as *Danio rerio*. The other contigs were classified with less taxonomic resolution, as reported in **Table S46**. Higher taxonomic ranks had lower classification confidence, since the classification of different *k-mers* along the entire contig was so diverse that all taxonomies were collapsed to the lowest common rank. Such contigs should be considered “unclassified” due to the low classification consensus.

Based on the results reported above, none of the aforementioned contigs were discarded. This was justified by the low confidence of detection (i.e., just a small fraction of the *k-mers* of each contig could be classified), which might be interpreted either as a classification error, as the presence of very short real contaminant fragments, or as the presence of very short nucleotide sequence stretches with high similarity due to chance, and not due to homology.

**Table S46. Summary of the KRAKEN 2 analysis of the contigs included in the final pan-genome assembly.**

| **contig name** | **classified taxon** | **classification confidence*** | **total confidence*** |
| --- | --- | --- | --- |
| GALF1_extra_0031656 | *Mytilus galloprovincialis* (taxid 29158) | 0.12 | 0.13 |
| GALF1_extra_0036470 | Protostomia (taxid 33317) | 0.00 | 0.10 |
| GALF1_extra_0037141 | *Mytilus galloprovincialis* (taxid 29158) | 0.11 | 0.12 |
| GALF1_extra_0051918 | *Mytilus* (taxid 6548) | 0.02 | 0.22 |
| GALF1_extra_0054072 | *Mytilus edulis* (taxid 6550) | 0.10 | 0.16 |
| GALF1_extra_0057706 | root (taxid 1) | 0.00 | 0.10 |
| GALF1_extra_0060155 | *Mytilus galloprovincialis* (taxid 29158) | 0.17 | 0.18 |
| GALF1_extra_0060279 | *Mytilus californianus* (taxid 6549) | 0.12 | 0.13 |
| GALF1_extra_0064782 | *Mytilus edulis* (taxid 6550) | 0.13 | 0.13 |
| GALF1_extra_0066152 | *Mytilus galloprovincialis* (taxid 29158) | 0.22 | 0.22 |
| GALF1_extra_0070312 | *Mytilus galloprovincialis* (taxid 29158) | 0.13 | 0.14 |
| GALF1_extra_0070449 | *Mytilus galloprovincialis* (taxid 29158) | 0.13 | 0.17 |
| GALF2_extra_0011122 | cellular organisms (taxid 131567) | 0.00 | 0.14 |
| GALF2_extra_0026106 | Mytilus galloprovincialis (taxid 29158) | 0.16 | 0.17 |
| GALF2_extra_0036300 | Eukaryota (taxid 2759) | 0.00 | 0.17 |
| GALF2_extra_0048508 | *Mytilus galloprovincialis* (taxid 29158) | 0.16 | 0.19 |
| GALF3_extra_0030902 | Danio rerio (taxid 7955) | 0.10 | 0.13 |
| PURA_extra_0002728 | *Mytilus* (taxid 6548) | 0.32 | 0.89 |
| PURA_extra_0008996 | *Mytilus galloprovincialis* (taxid 29158) | 0.41 | 0.50 |
| PURA_extra_0011319 | *Mytilus edulis* (taxid 6550) | 0.18 | 0.49 |
| GALM1_extra_0030109 | cellular organisms (taxid 131567) | 0.00 | 0.10 |
| GALM1_extra_0030191 | Eukaryota (taxid 2759) | 0.08 | 0.15 |
| GALM2_extra_0025454 | *Mytilus galloprovincialis* (taxid 29158) | 0.24 | 0.24 |
| GALM3_extra_0016282 | *Mytilus* (taxid 6548) | 0.01 | 0.11 |
| GALM6_extra_0005895 | *Mytilus galloprovincialis* (taxid 29158) | 0.15 | 0.15 |
| GALM6_extra_0012516 | Eumetazoa (taxid 6072) | 0.00 | 0.10 |
| ITAF1_extra_0002077 | *Mytilus galloprovincialis* (taxid 29158) | 0.26 | 0.27 |
| ITAF1_extra_0006336 | *Mytilus galloprovincialis* (taxid 29158) | 0.20 | 0.20 |
| ITAF1_extra_0019696 | *Mytilus galloprovincialis* (taxid 29158) | 0.11 | 0.17 |
| ITAF2_extra_0009723 | *Mytilus galloprovincialis* (taxid 29158) | 0.10 | 0.11 |
| ITAM2_extra_0001711 | cellular organisms (taxid 131567) | 0.02 | 0.10 |
| ITAM2_extra_0011866 | cellular organisms (taxid 131567) | 0.02 | 0.10 |

*the total confidence is defined as the fraction of *k-mers* contained in a given contig mapped to known taxonomical units. The classification confidence is defined as the fraction of *k-mers* contained in a given contig mapped to the the top scoring species.

**Figures S77-S91** schematically display the main features of the *de novo* recursively reassembled pan-genomic contigs, before and after the filtering/decontamination process, as density heat maps. The graphs correlate GC content (Y axis) with the median sequencing coverage (X axis). Consistently with the results reported and discussed above, in spite of the removal of a conspicuous number of contigs, none of the resequenced individuals displayed highly significant distribution shifts, with the exception of ITAF3 (**Fig. S88**), which will be discussed in detail in the next section.


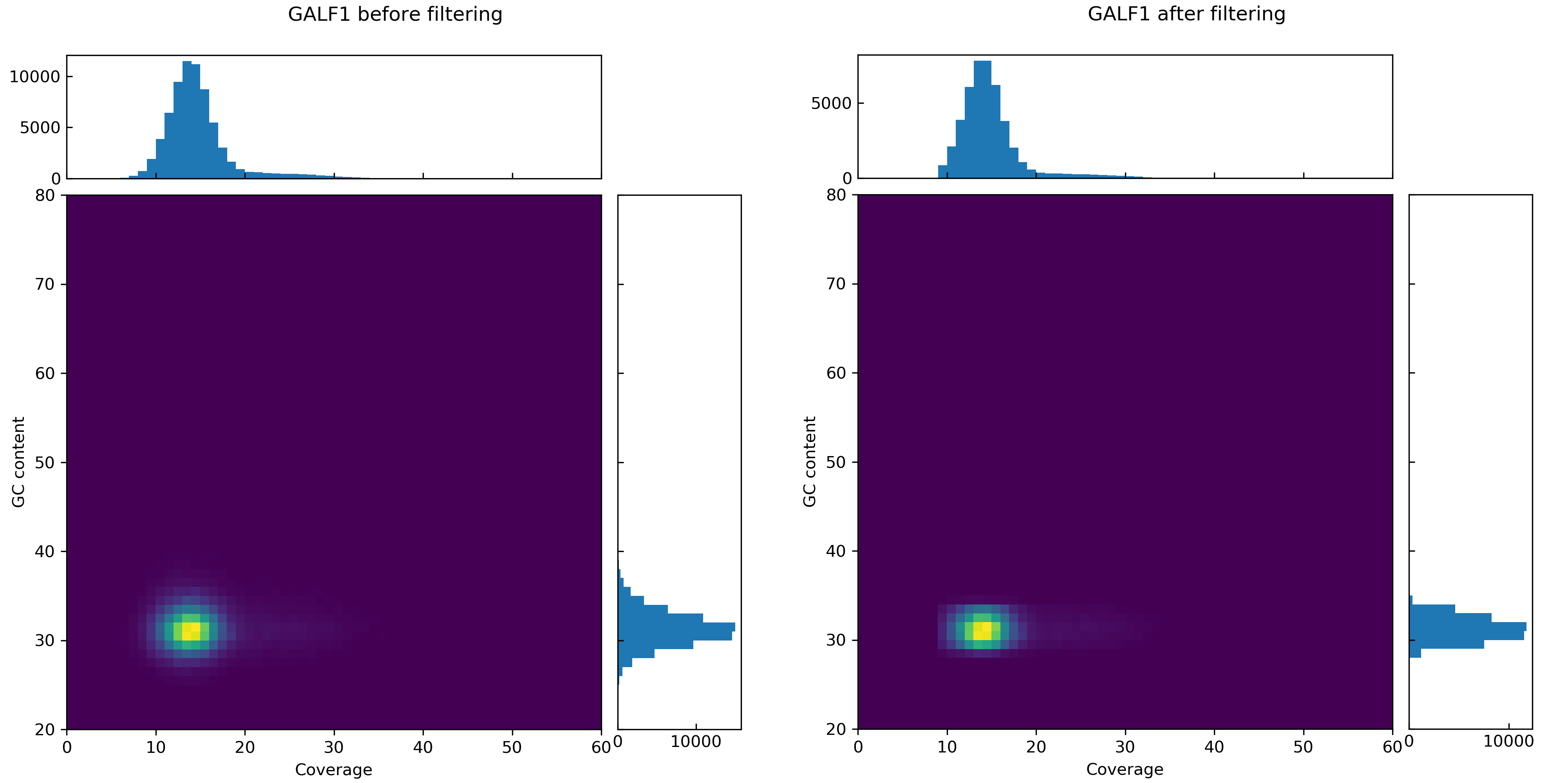


**Fig. S77**. **GC content and sequencing coverage of GALF1 contigs, before and after the decontamination/filtering process.** The contig distribution is shown as a density heat map, whose interpretation is simplified by the histograms found at the top and the side of the graphs.


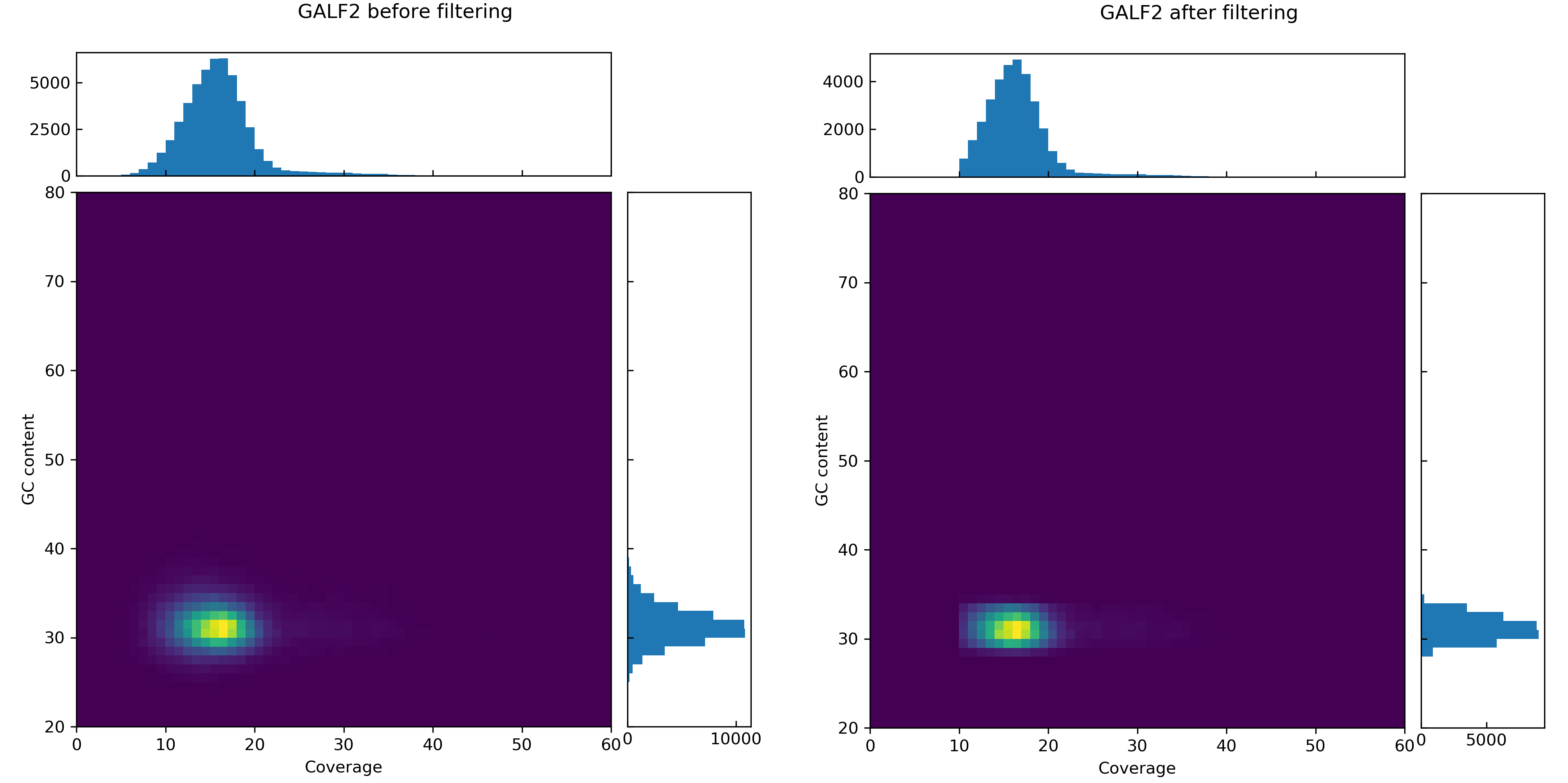


**Fig. S78**. **GC content and sequencing coverage of GALF2 contigs, before and after the decontamination/filtering process.** The contig distribution is shown as a density heat map, whose interpretation is simplified by the histograms found at the top and the side of the graphs.


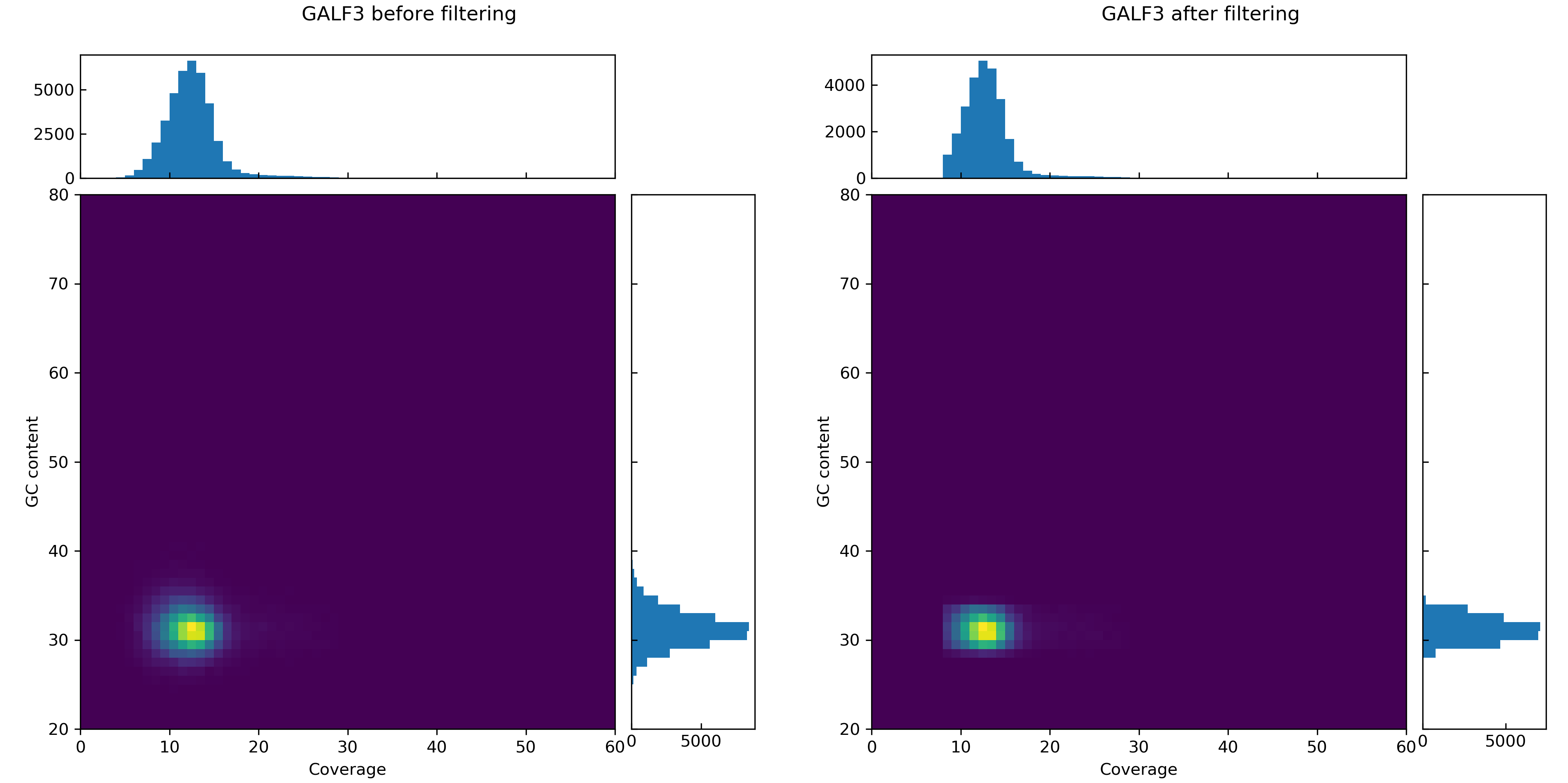


**Fig. S79**. **GC content and sequencing coverage of GALF3 contigs, before and after the decontamination/filtering process.** The contig distribution is shown as a density heat map, whose interpretation is simplified by the histograms found at the top and the side of the graphs.


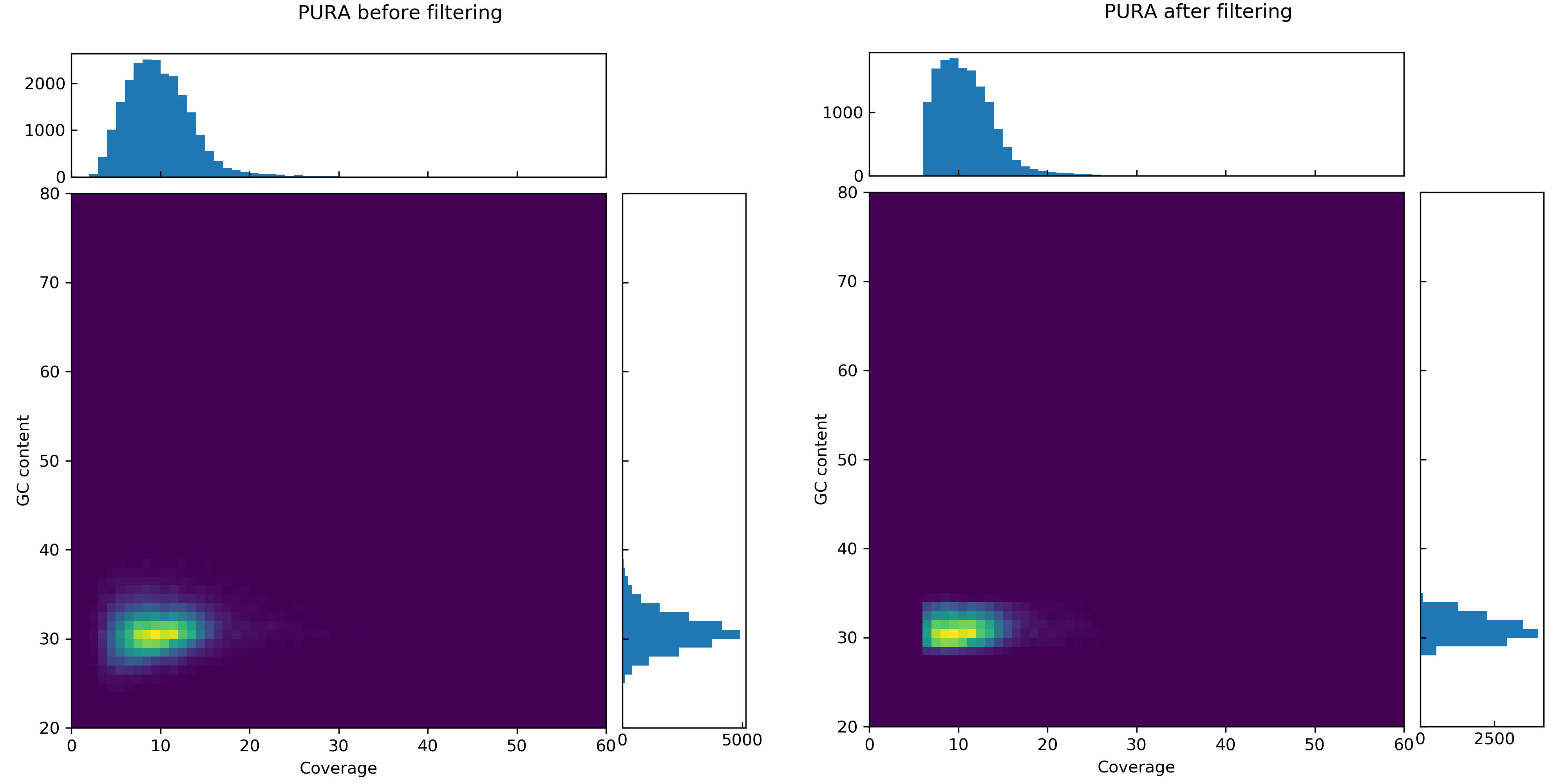


**Fig. S80**. **GC content and sequencing coverage of *Pura* contigs, before and after the decontamination/filtering process.** The contig distribution is shown as a density heat map, whose interpretation is simplified by the histograms found at the top and the side of the graphs.


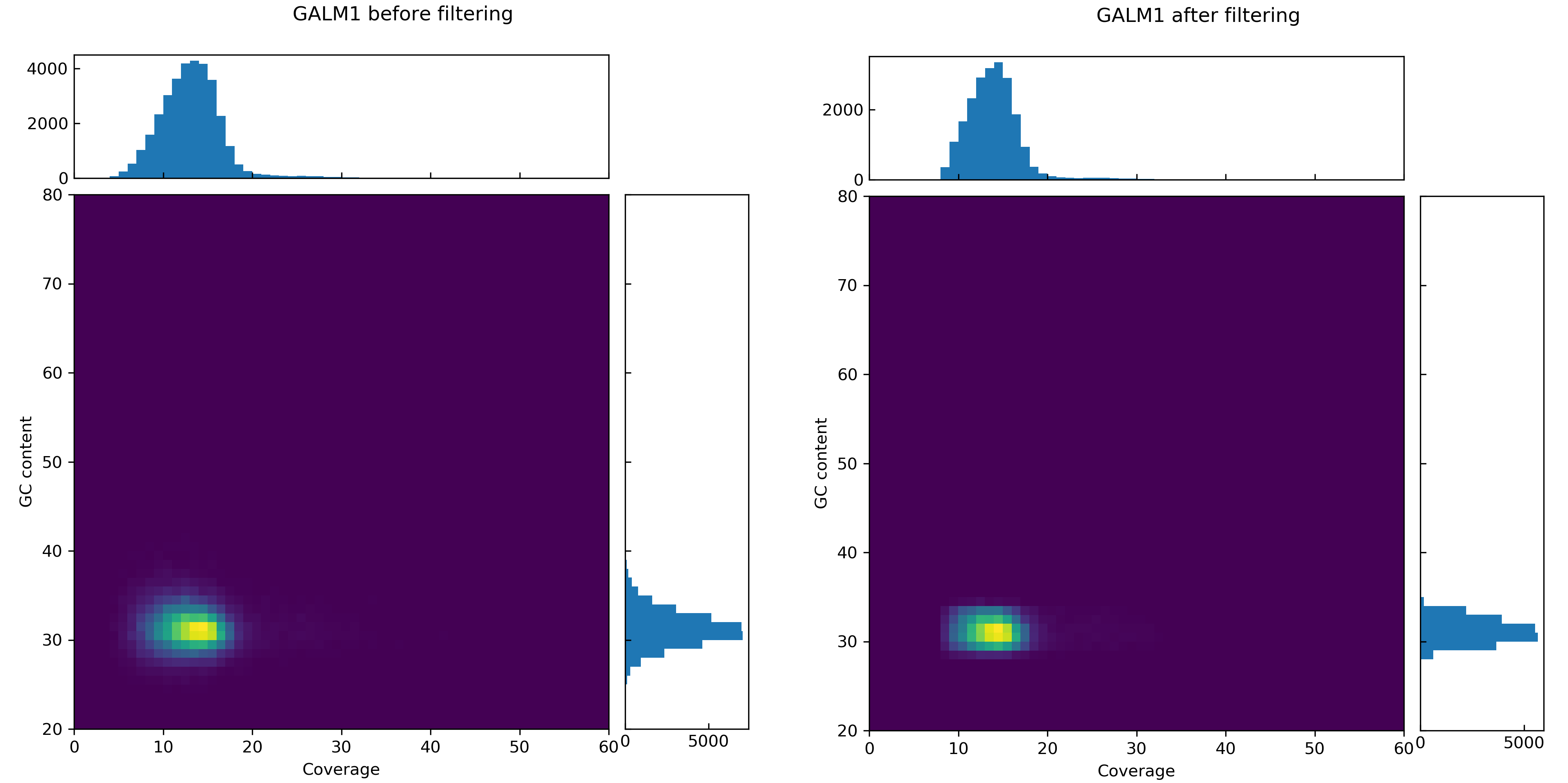


**Fig. S81**. **GC content and sequencing coverage of GALM1 contigs, before and after the decontamination/filtering process.** The contig distribution is shown as a density heat map, whose interpretation is simplified by the histograms found at the top and the side of the graphs. Note: this graph was obtained using the sequencing output obtained from the gills sample.


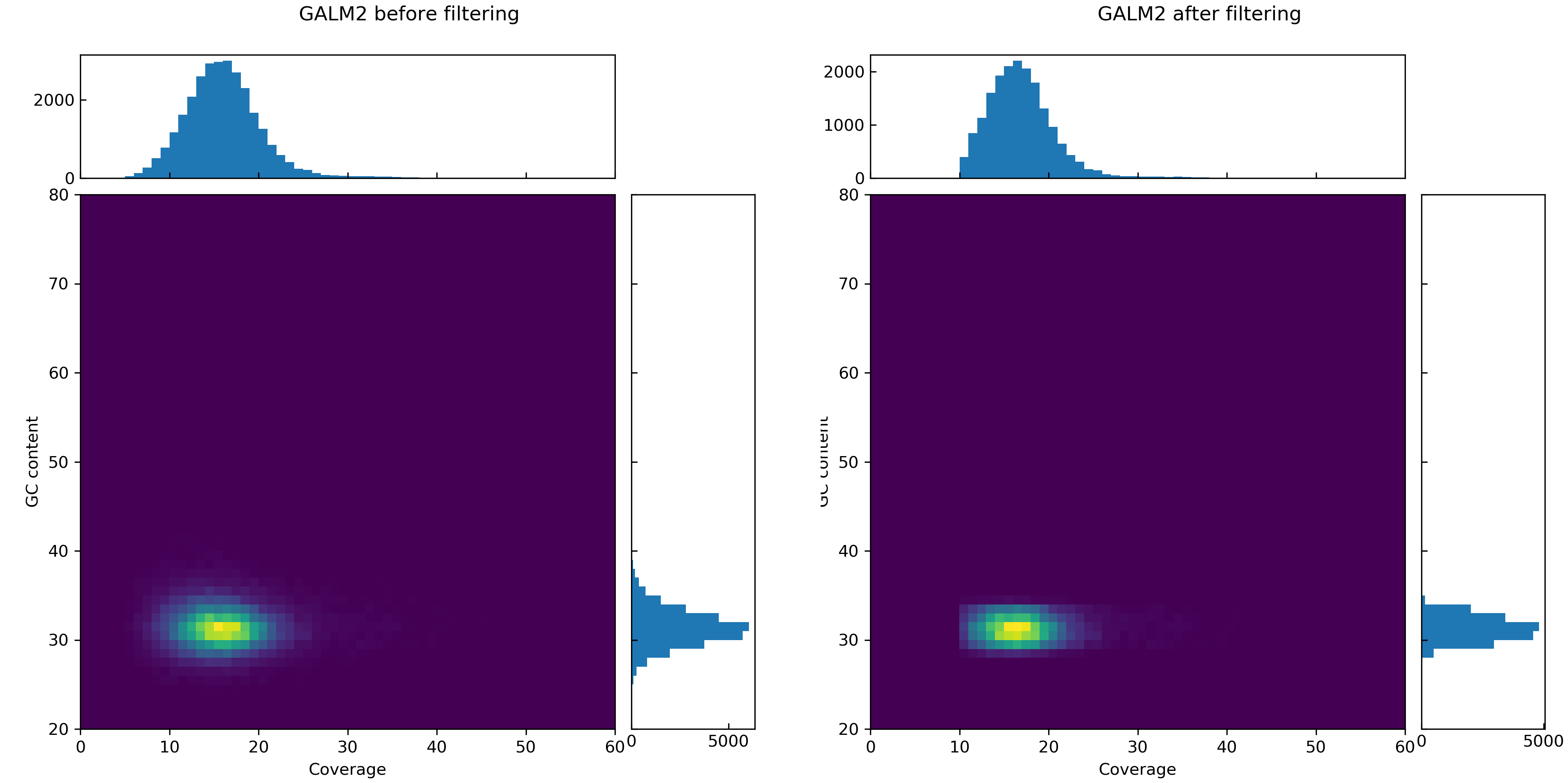


**Fig. S82**. **GC content and sequencing coverage of GALM2 contigs, before and after the decontamination/filtering process.** The contig distribution is shown as a density heat map, whose interpretation is simplified by the histograms found at the top and the side of the graphs.


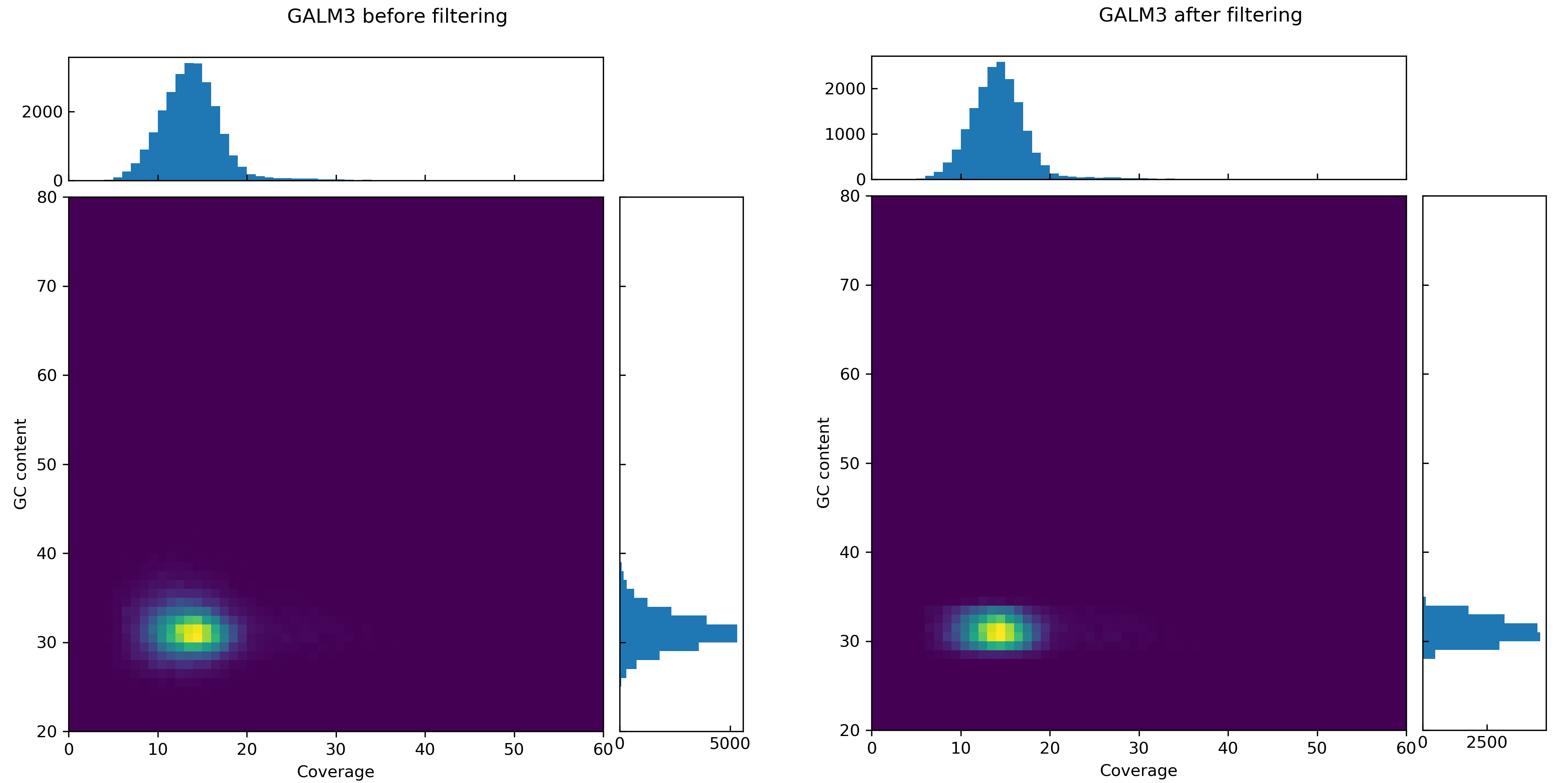


**Fig. S83**. **GC content and sequencing coverage of GALM3 contigs, before and after the decontamination/filtering process.** The contig distribution is shown as a density heat map, whose interpretation is simplified by the histograms found at the top and the side of the graphs.


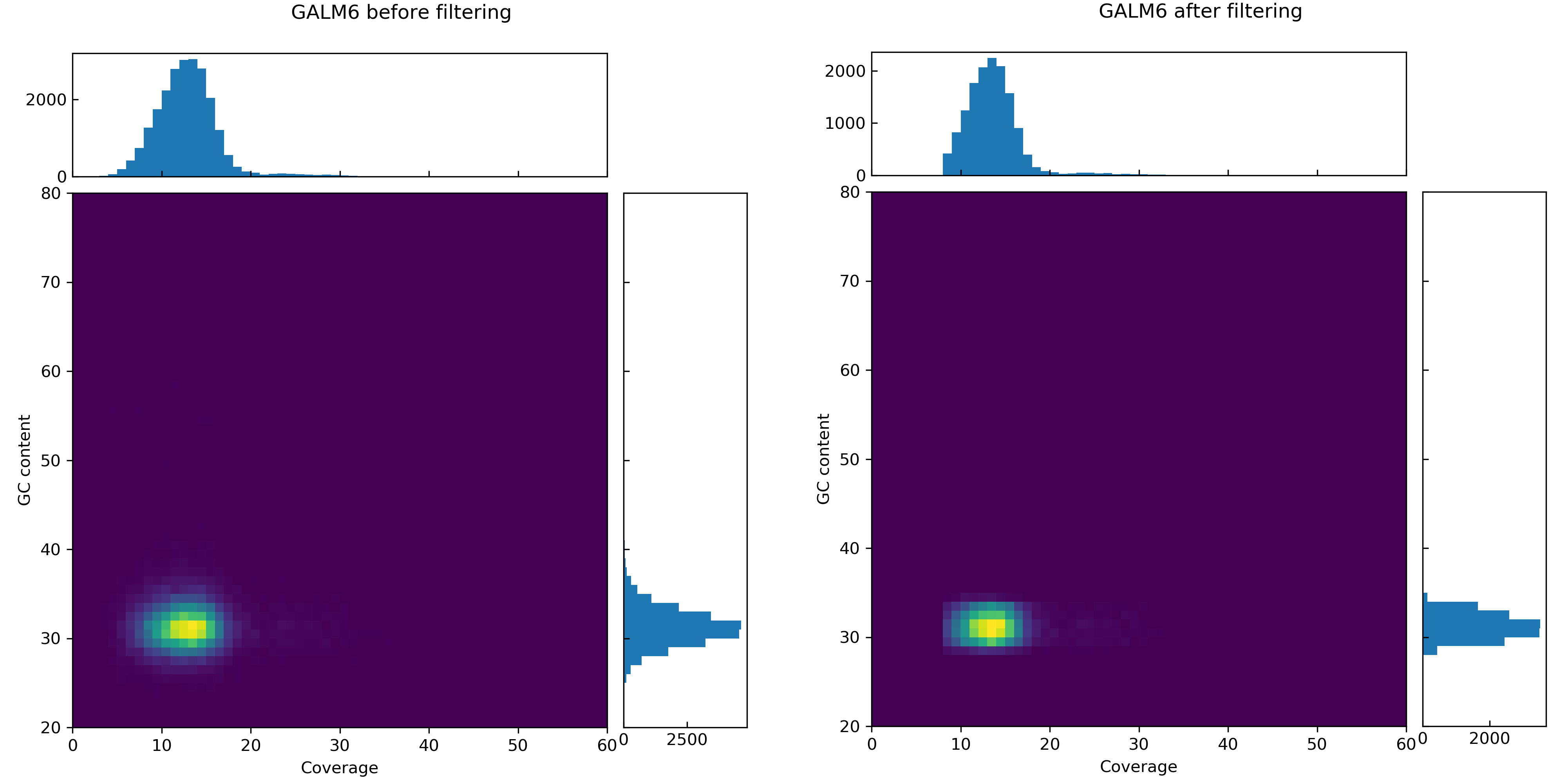


**Fig. S84**. **GC content and sequencing coverage of GALM6 contigs, before and after the decontamination/filtering process.** The contig distribution is shown as a density heat map, whose interpretation is simplified by the histograms found at the top and the side of the graphs.


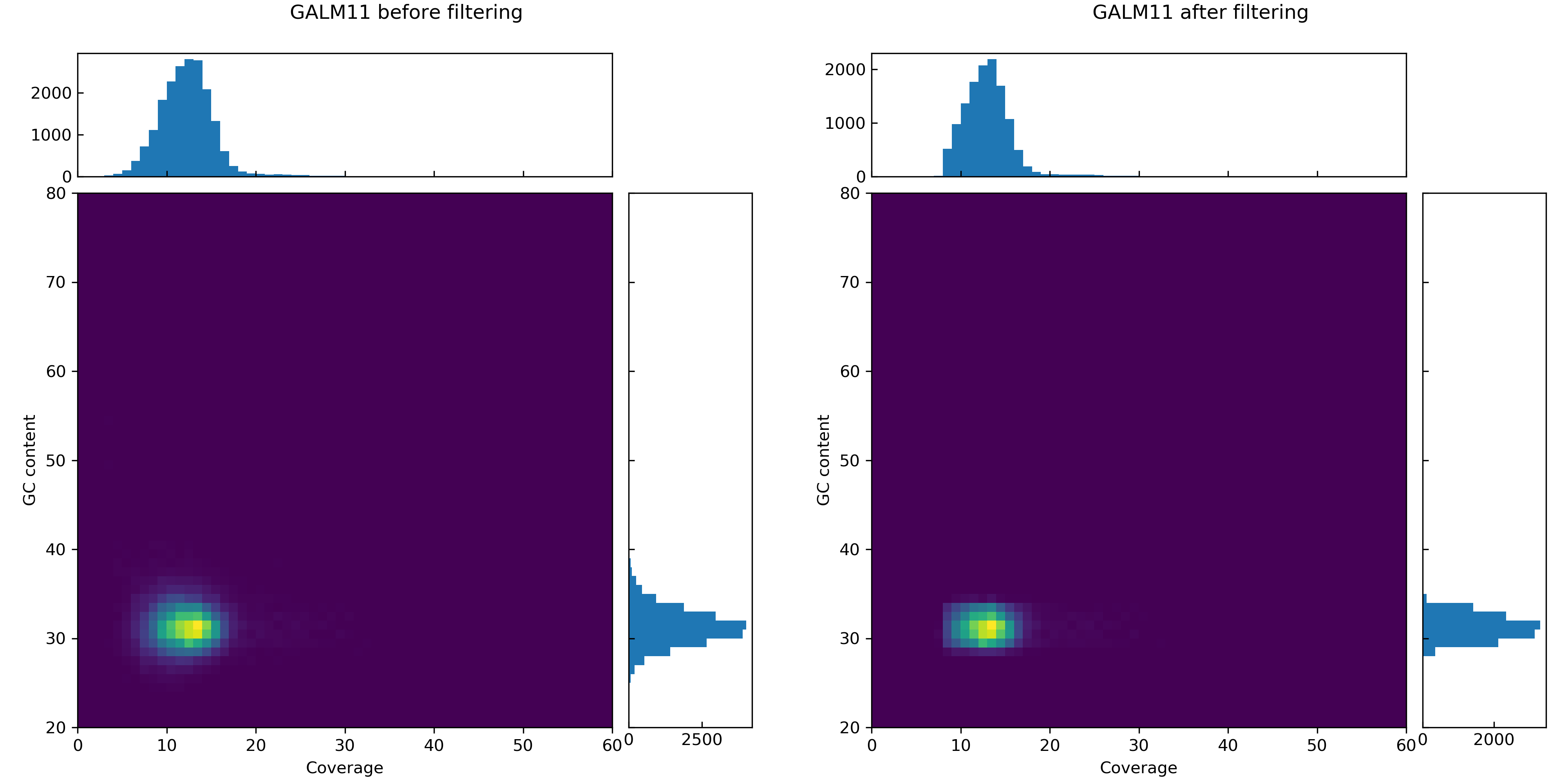


**Fig. S85**. **GC content and sequencing coverage of GALM11 contigs, before and after the decontamination/filtering process.** The contig distribution is shown as a density heat map, whose interpretation is simplified by the histograms found at the top and the side of the graphs.


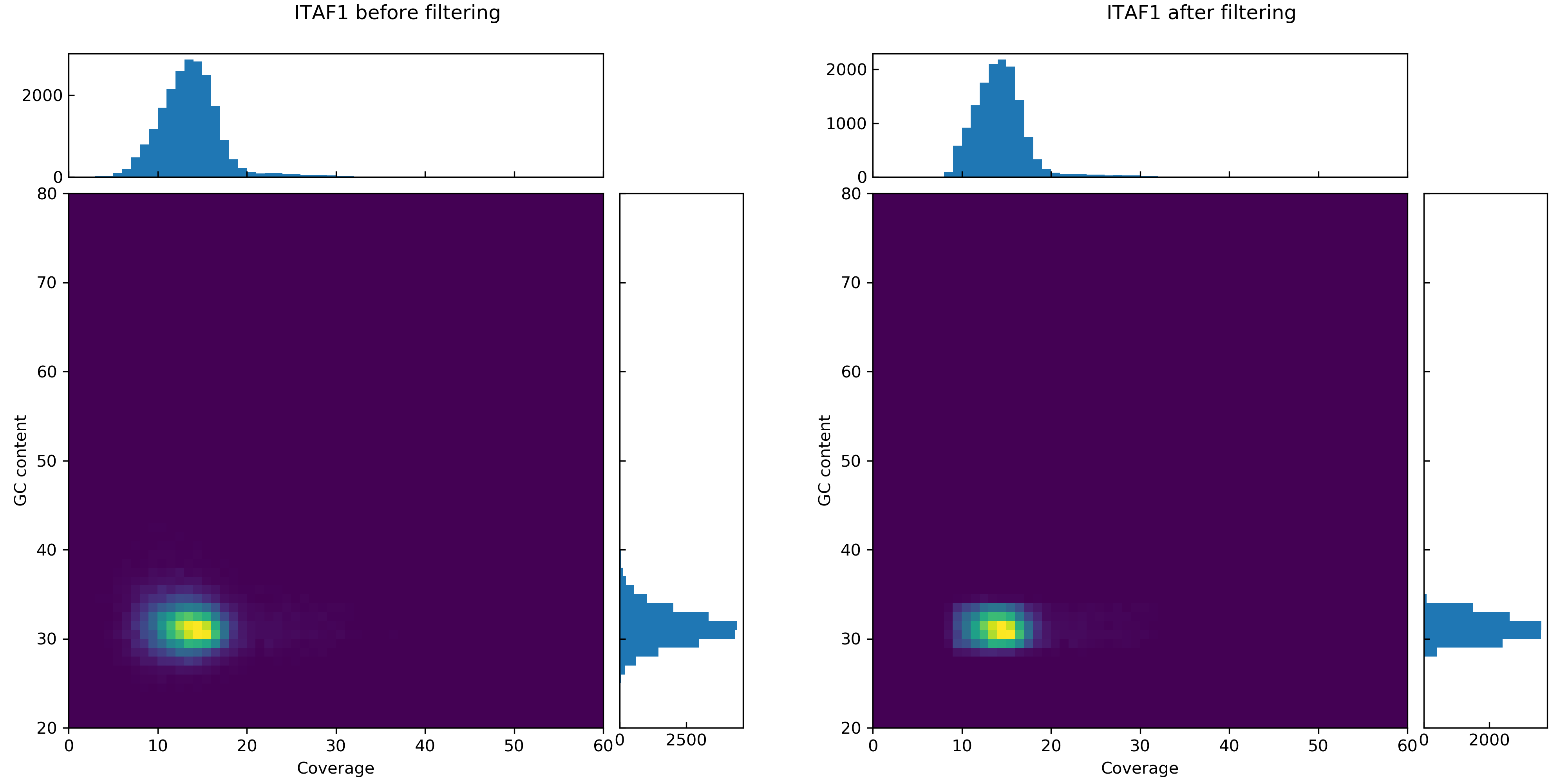


**Fig. S86**. **GC content and sequencing coverage of ITAF1 contigs, before and after the decontamination/filtering process.** The contig distribution is shown as a density heat map, whose interpretation is simplified by the histograms found at the top and the side of the graphs.


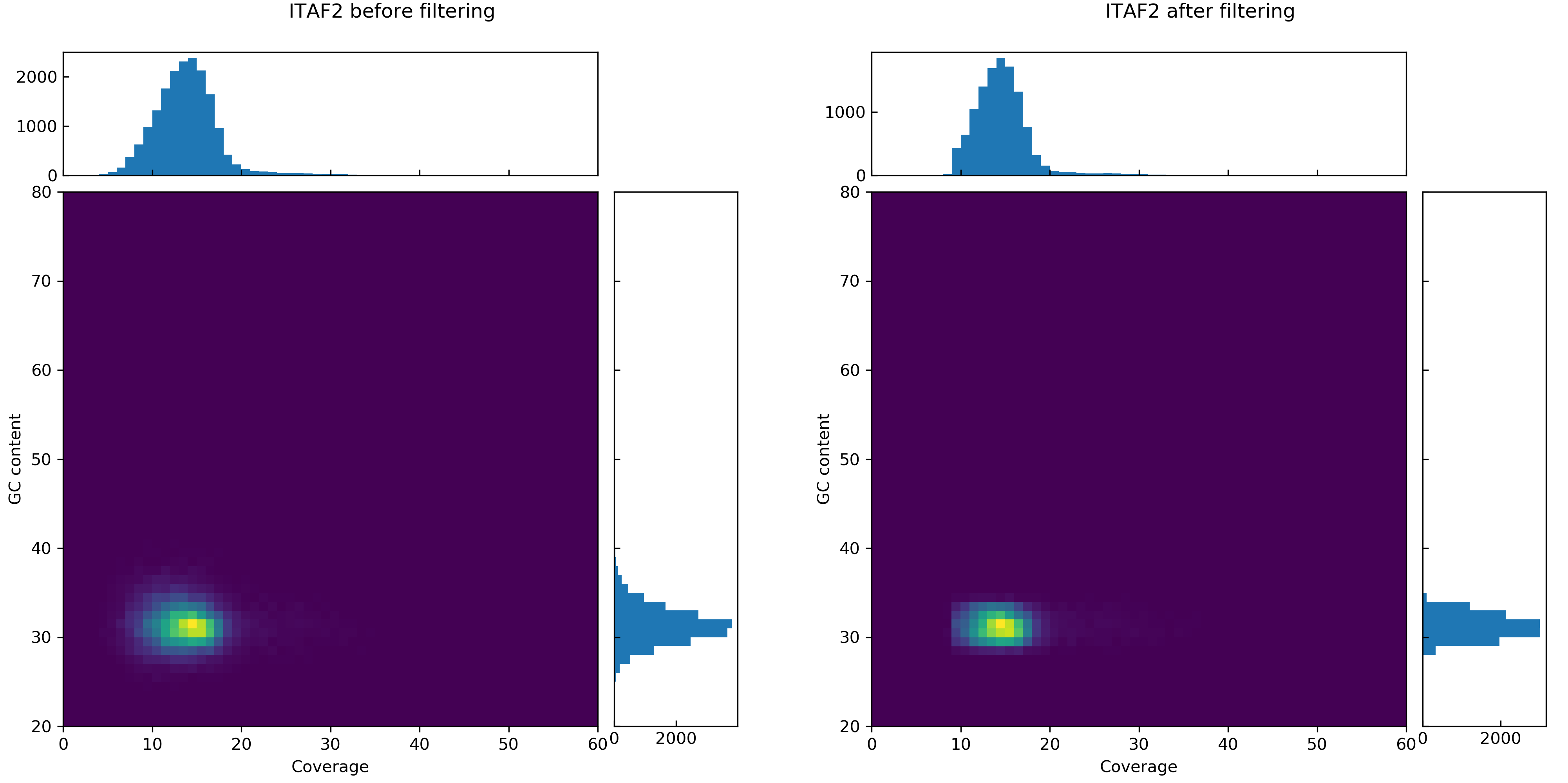


**Fig. S87**. **GC content and sequencing coverage of ITAF2 contigs, before and after the decontamination/filtering process.** The contig distribution is shown as a density heat map, whose interpretation is simplified by the histograms found at the top and the side of the graphs.


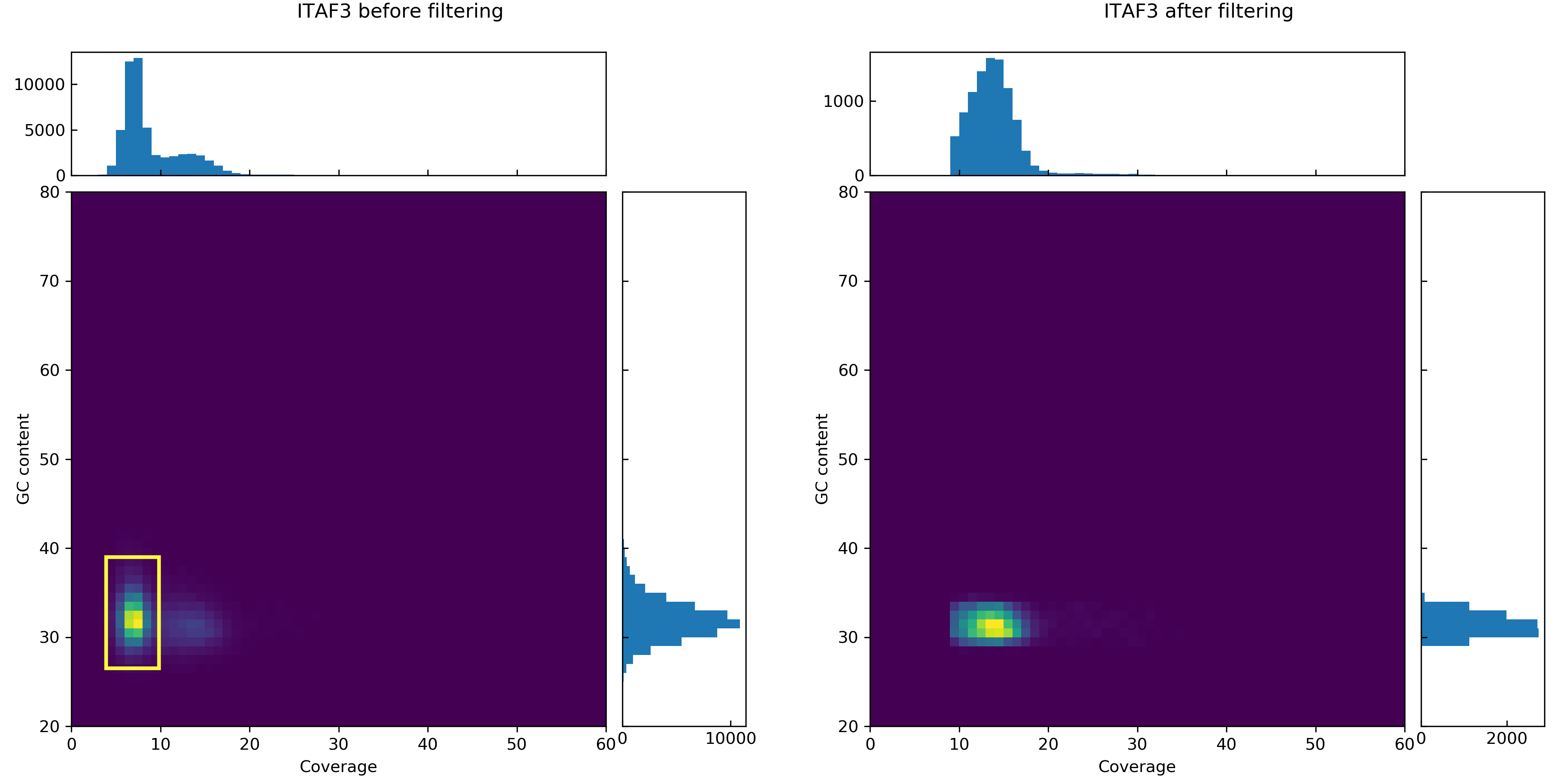


**Fig. S88**. **GC content and sequencing coverage of ITAF3 contigs, before and after the decontamination/filtering process.** The contig distribution is shown as a density heat map, whose interpretation is simplified by the histograms found at the top and the side of the graphs. Note the significant exogenous contamination identified in this sample, and its efficient removal with the decontamination process.


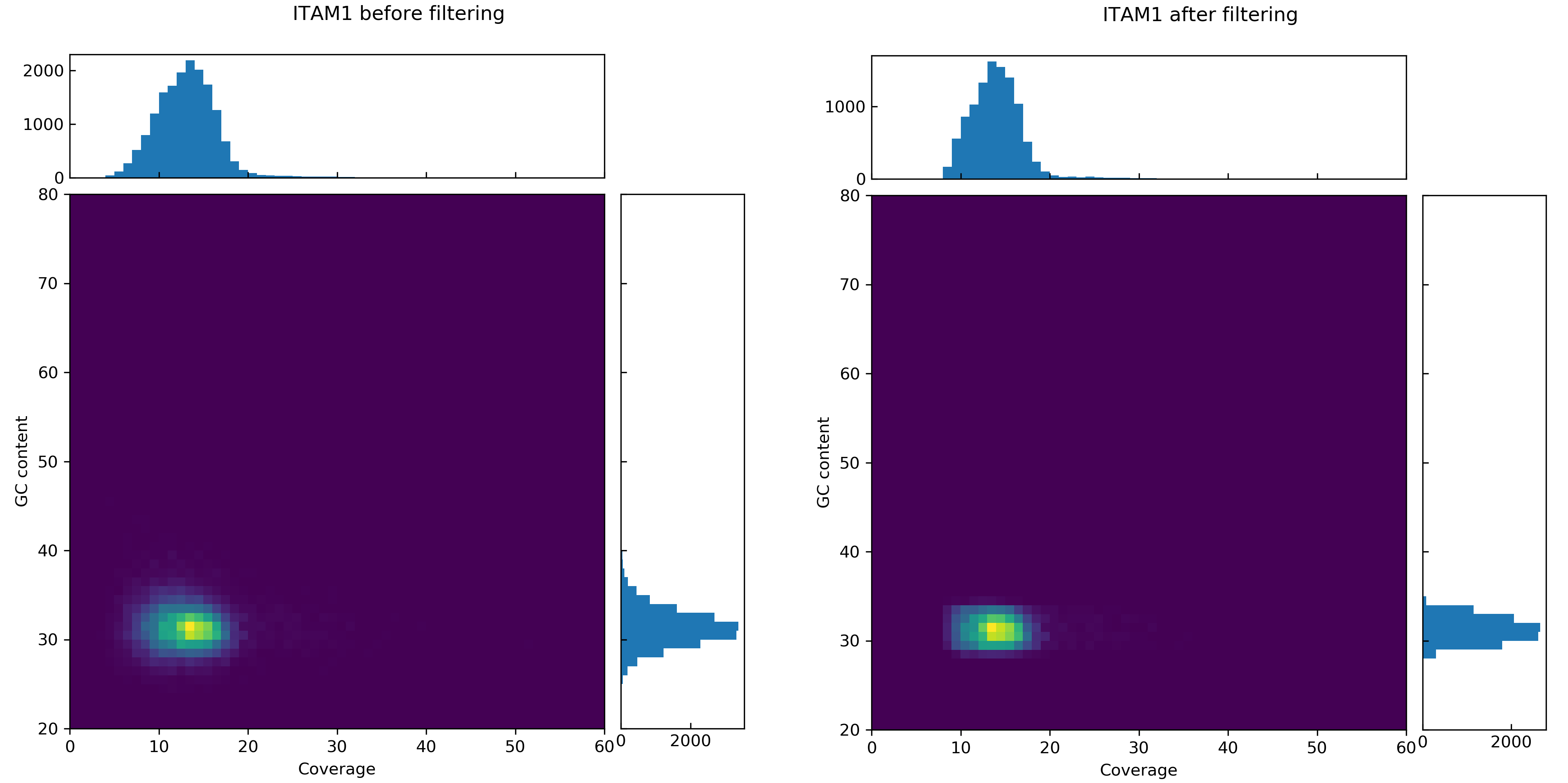


**Fig. S89**. **GC content and sequencing coverage of ITAM1 contigs, before and after the decontamination/filtering process.** The contig distribution is shown as a density heat map, whose interpretation is simplified by the histograms found at the top and the side of the graphs. Note: this graph was obtained using the sequencing output obtained from the gills sample.


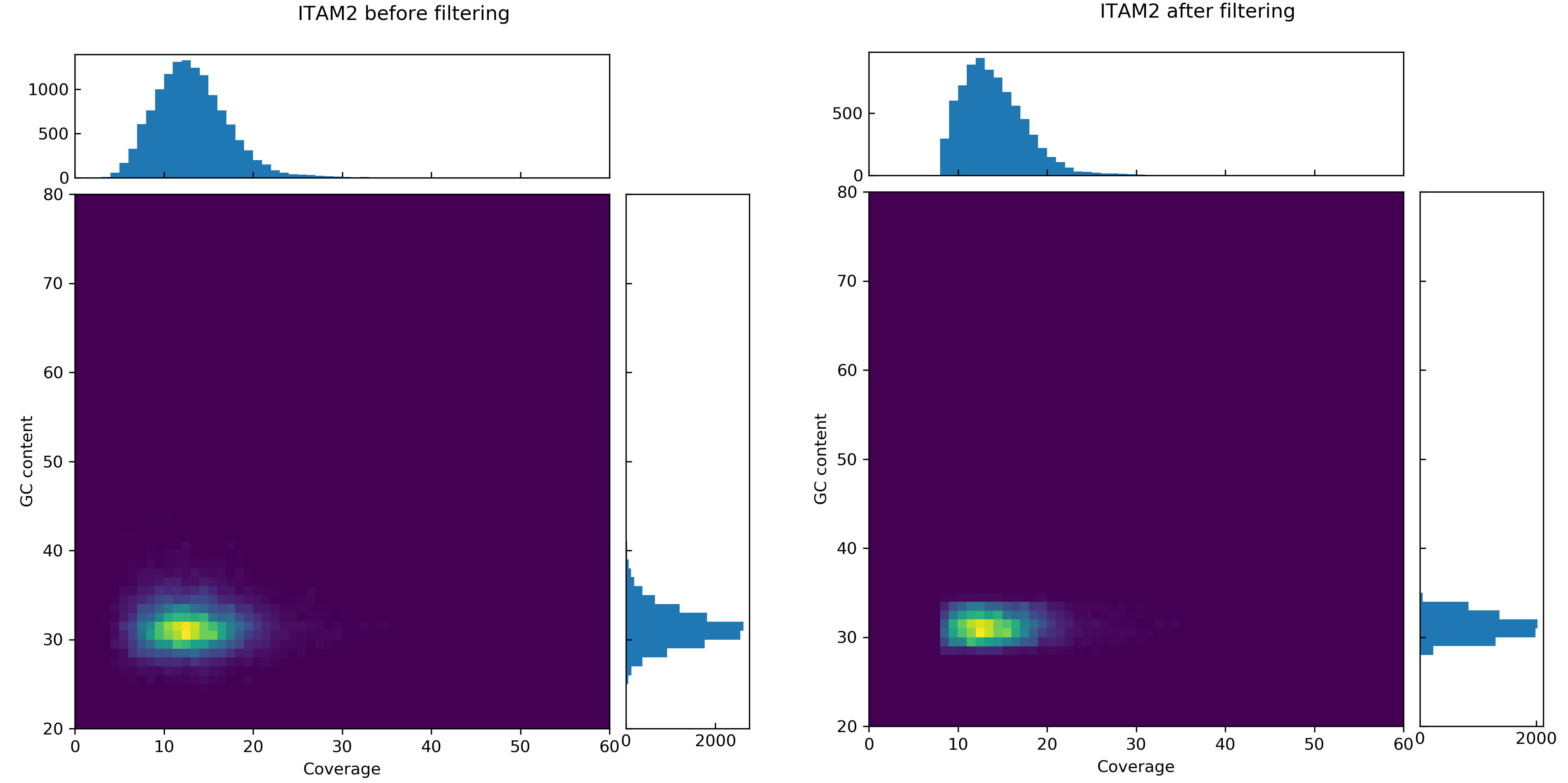


**Fig. S90**. **GC content and sequencing coverage of ITAM2 contigs, before and after the decontamination/filtering process.** The contig distribution is shown as a density heat map, whose interpretation is simplified by the histograms found at the top and the side of the graphs.


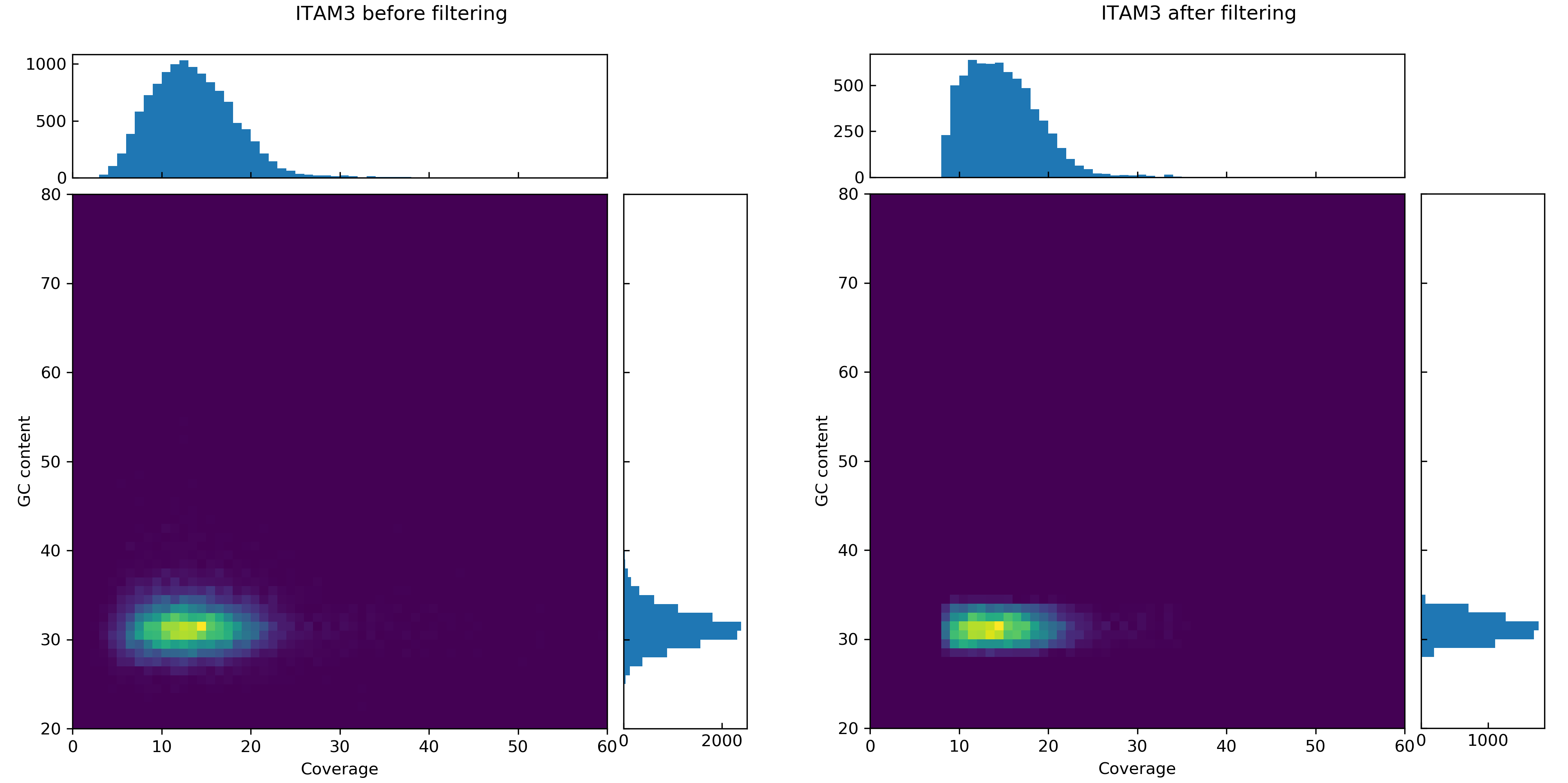


**Fig. S91**. **GC content and sequencing coverage of ITAM3 contigs, before and after the decontamination/filtering process.** The contig distribution is shown as a density heat map, whose interpretation is simplified by the histograms found at the top and the side of the graphs.

## ITAF3: an exemple of efficient decontamination from the exogenous DNA of a mantle parasitic hydrozoan

As previously mentioned, several of the contigs obtained from the ITAF3 individual displayed a significant bias in terms of both GC content (towards higher values compared with the expectations from *M. galloprovincialis*, see **Fig. S73**) and in terms of sequencing coverage (towards lower values compared with expectations, see **Fig. S74**). These anomalies are highlighted by the density plot shown in **Fig. S88**.

As mentioned above, the application of stringent GC content and coverage thresholds, as well as the use of a custom contaminant database used for BLAST checks, allowed to remove the vast majority of these contaminant contigs (see **Fig. S68**), with the flagging of 82.02% of the total contigs assembled from this sample. Due to their characteristics, we might expect these contigs to have been originated by a unique source of exogenous contamination, characterized by a lower GC content than mussel, and found in lower proportion in the original sample compared with mussel DNA (as evidenced by the lower ,median sequencing coverage of the samples).

We positively identified this source of contamination as *Eugymnanthea inquilina*, an hydrozoan species known to sometimes infest *M. galloprovincialis*, living attached to the mantle tissue [132], and hence very likely to have been accidentally sampled along with the dissection of the mantle tissue in this particular individual. As no full genome or transcriptome was available for this species, the identification of this species was made possible thanks to the comparison between the voucher 16S rRNA sequence (GenBank accession ID: AY789832.1), the voucher 18S rRNA sequence (GenBank accession ID: AY789775.1) and the voucher COI sequence (GenBank accession ID: AY789915.1) [136] and assembled contigs.

The 16S rRNA sequence resulted to be 100% identical to a contig found in the ITAF3 original assembly (**Fig. S92**) and so was the 18S rRNA sequence (**Fig. S93**). The COI sequence displayed a single nuscleotide mismatch, attaining a 99.83% identity level, but it was still highly consistent with species identification (**Fig. S94**).


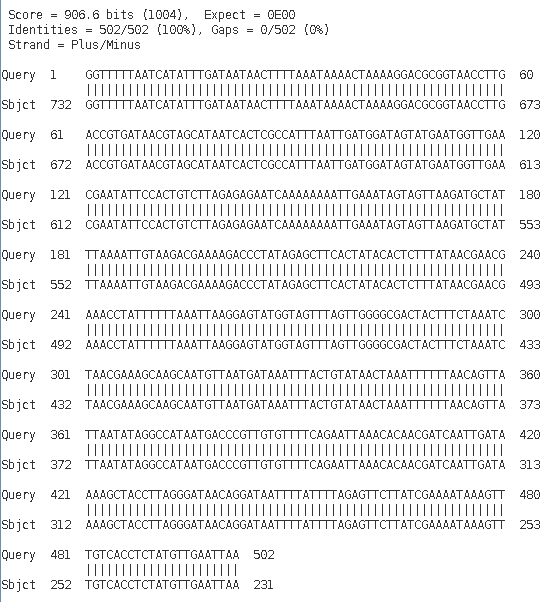


**Fig. S92**. **Alignment between the voucher nucleotide sequence of the 16S rRNA from *E. inquilina* (query) and the best match in the ITAF3 reassembly (subject).**


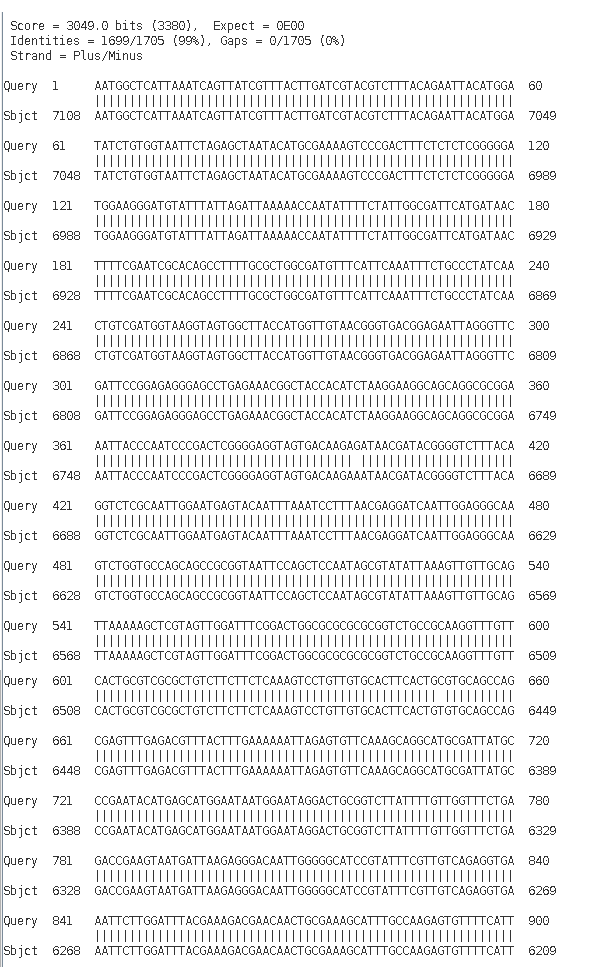


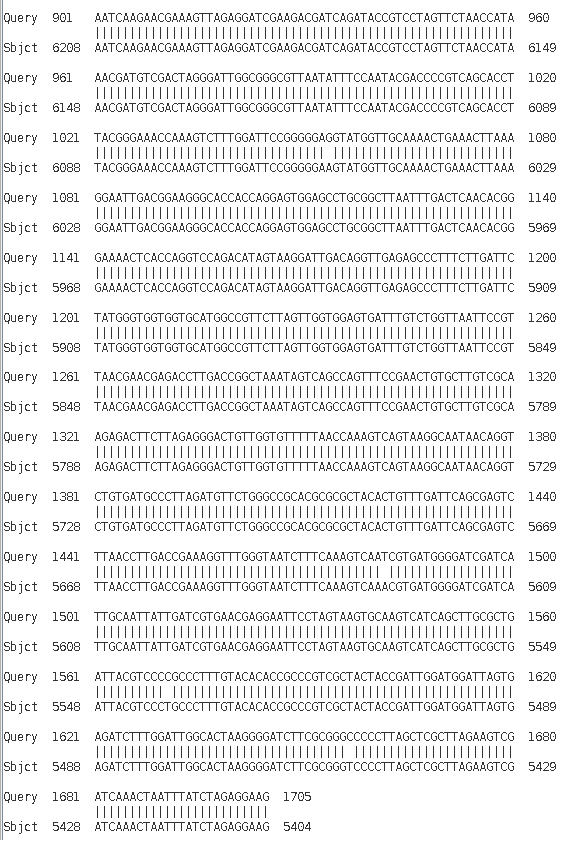


**Fig. S93**. **Alignment between the voucher nucleotide sequence of the 18S rRNA from *E. inquilina* (query) and the best match in the ITAF3 reassembly (subject).**

**
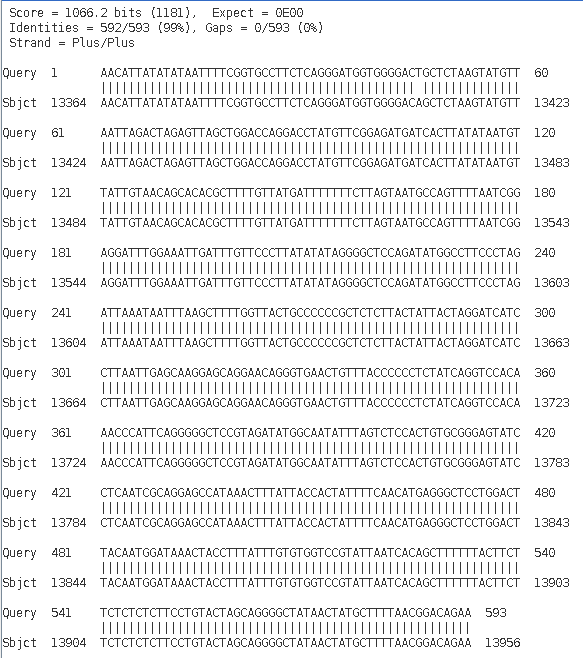
**

**Fig. S94**. **Alignment between the voucher nucleotide sequence of the COI from *E. inquilina* (query) and the best match in the ITAF3 reassembly (subject).**

# Data Note 15 – Evaluation of Presence-Absence Variation on the *dispensable* genes from the pan-genome

## Pan-genomic *dispensable* genes annotation

The gene annotation process was carried out using the same methodology described in **Data Note 2**. However, most of the reassembled pan-genomic contigs were short (see **Data Note 14**), in part due to the “relatively small” size of the hemizygous genomic regions, but likely also due to the unavailability of long (i.e., SMRT PacBio) reads to improve their *de novo* assembly in resequenced individuals. This factor might have led to a significant fragmentation of the hemizygous regions, for example whenever repeated elements were present, determining the breakage of long genes among multiple contigs. To mitigate this issue and avoid excessively inflating the number of annotated genes, we chose to restrict the annotation pipeline to a subset of *de novo* recursively reassembled contigs whose size exceeded the median length of the *dispensable* genes annotated in *Lola* mg10 (i.e., 4,310 nucleotides). This strategy was implemented at the inevitable cost of losing a certain number of small complete genes that could have been potentially annotated, but which were contained in particularly short contigs.

The contigs size distribution of the pan-genome assembly is reported in **Fig. S95**, which indicates that the number of contigs subjected to annotation was 15,964, equal to 5.97% of the total.


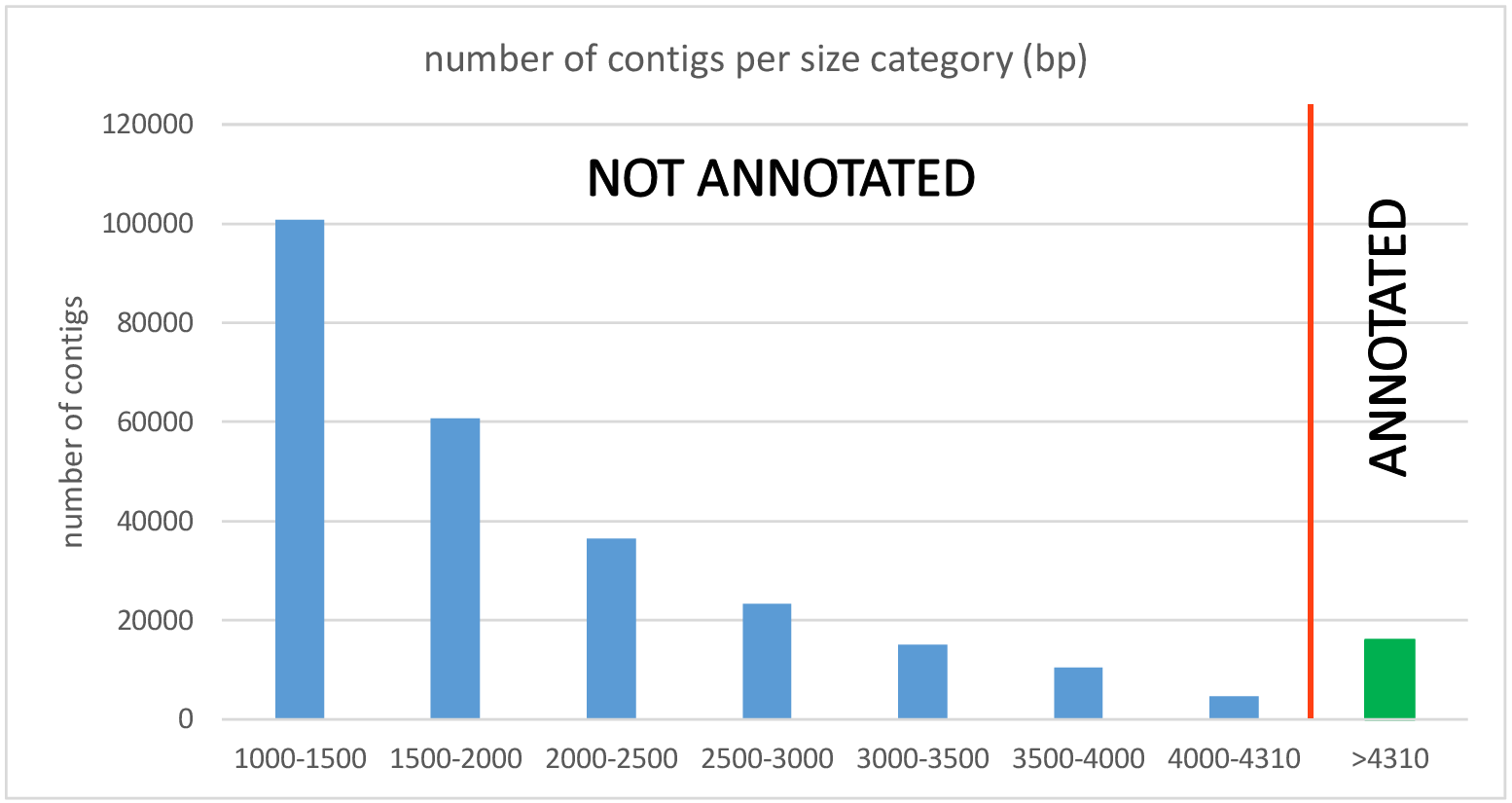


**Fig. S95**. **Distribution of *de novo* recursively reassembled pan-genomic contigs size, with indication about the threshold used for annotation.**

The same data are reported in **Fig. S96**, which shows the curve of the cumulative number of contigs subjected to annotation.


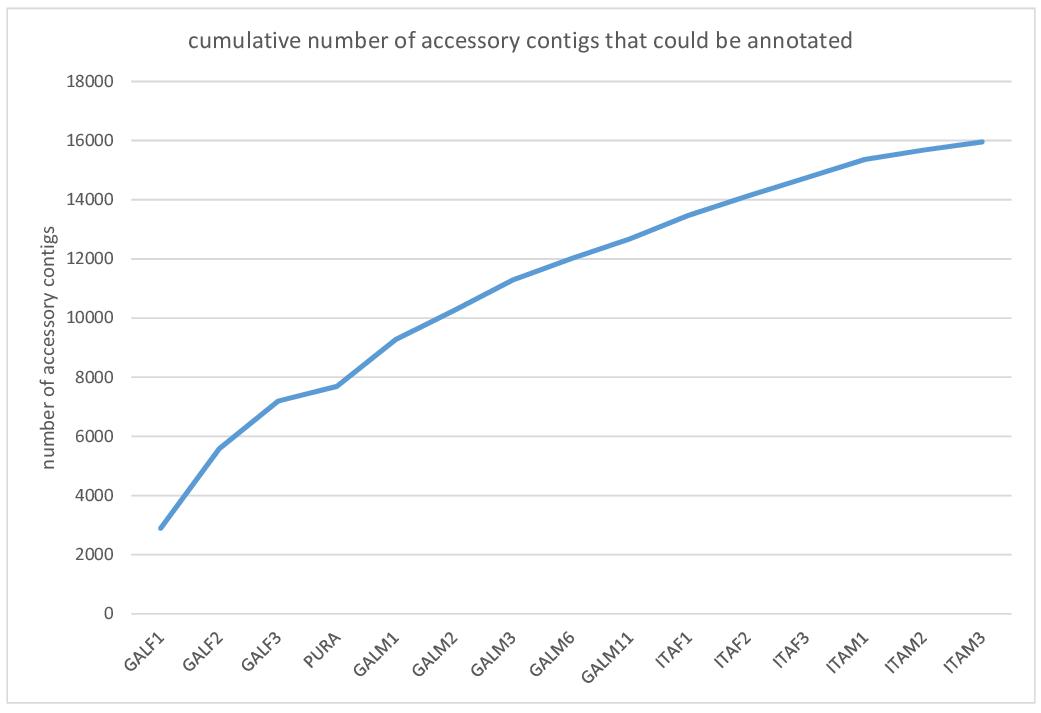


**Fig. S96**. **Cumulative number of accessory contigs that could be annotated (i.e., whose length exceeded the minimum threshold set) included in the mussel pan-genome recursive reassembly, after the filtering/decontamination step.**

The annotation process allowed to identify 5,286 genes, 1,767 (33.43%) of which were full-length, and 3,519 (66.57%) were considered to be partial. This observation confirms the limitations of a *de novo* assembly of the mussel genome using only short reads, evidenced by previous sequencing and assembly efforts carried out in *M. galloprovincialis* [17,27], and further justifies our strategy of restricting annotation to a subset of contigs satisfying a minimum length threshold. Nevertheless, these results also point out that the annotation statistics obtained from this dataset should be considered with caution, as they might be affected by fragmentation, and the consequent over-estimate of gene families characterized by short length, or under-estimate of gene families characterized by large size.

A total of 5,045 contigs were annotated with at least one gene, accounting for 31.60% of those analyzed, revealing that a significant number of the contigs exceeding 4,310 bp in length were devoid of protein-coding genes, This further reinforces the idea that hemizygous genomic regions often contain non-coding genes (not analyzed for this contig dataset, see **Data Note 9**) and, most likely, they often correspond to intergenic regions.

Consistently with the relatively short size of the assembled contigs, most of them included a single annotated gene (4,917, i.e., 96.72%). A total of 146 contigs (2.87%) included two annotated genes, and two contigs included 7 annotated protein-coding genes (**Fig. S97**).


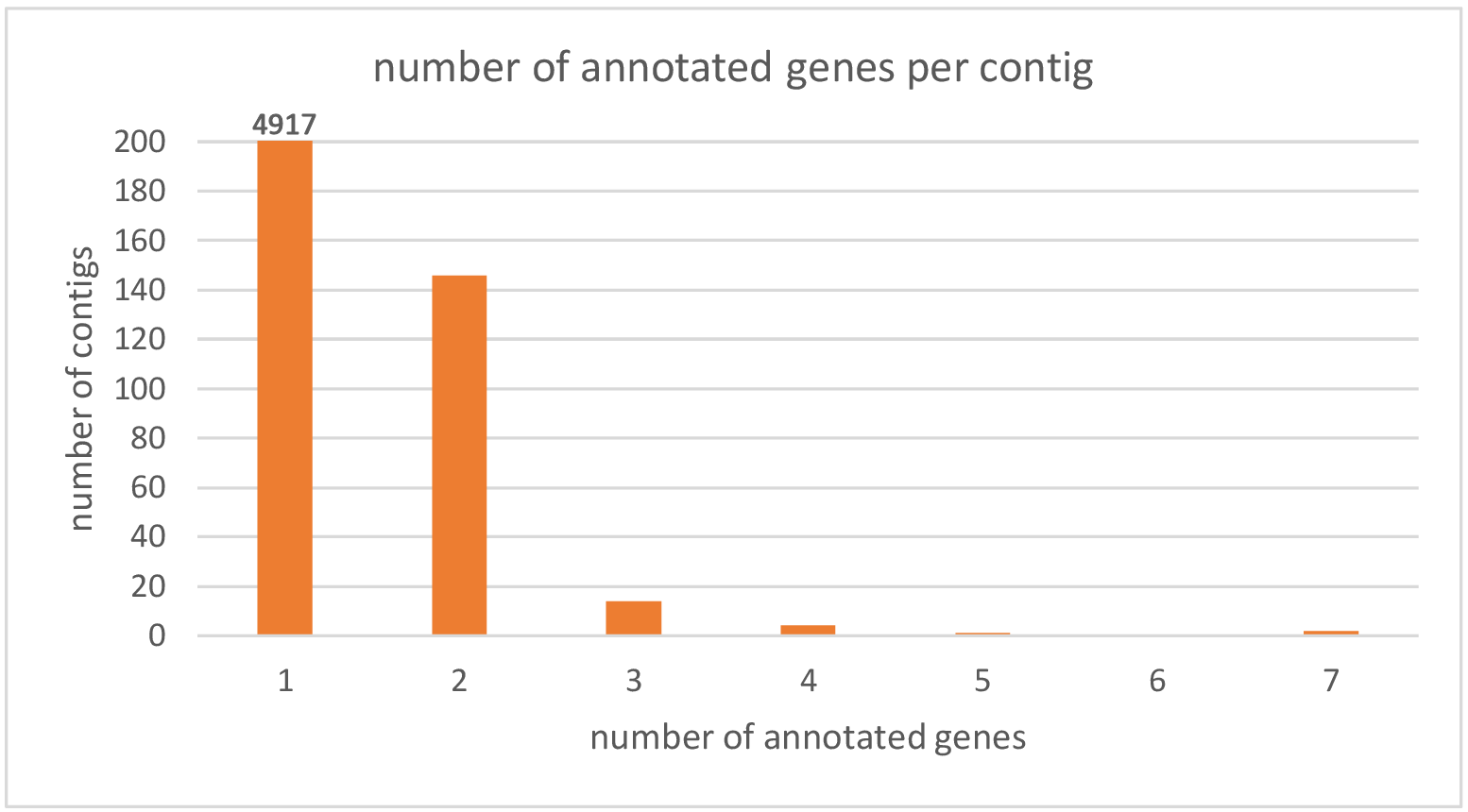


**Fig. S97**. **Number of annotated genes per contig in the *dispensable* contigs part of *de novo* reassembled pan-genome.**

## Presence-Absence Variation analysis

The *dispensable* nature of the newly annotated genes was confirmed though the mapping of the sequencing data for each genome, as described in **Data Note 8**. The absence of these genes in *Lola* was evaluated by the mapping of the reads obtained from the gill tissue. This additional step was used as a further confirmation of the efficiency of the filtering/decontamination protocol described in detail in **Data Note 14**.

On average, 1,974 out of the 5,286 newly annotated *dispensable* genes (25.99% out of the total) were identified in each resequenced genome (**Fig. S98**). The highest number was observed in ITAF3 (1,497), whereas the lowest number was observed in *Pura* (1,157). These numbers, added to the number of *dispensable* genes identified from the coverage analysis of the *dispensable* protein-coding genes found in the reference genome (**Data Note 8**), indicates that each individual lacks, on average, 8,141 out of the *dispensable* genes identified in the mussel- pan-genome. As a control, in line with our expectations and with the rigorous decontamination protocol employed (see **Data Note 14**), none of these genes were detected in *Lola*.

The majority of these *dispensable* genes were found at very low frequencies in our sample. In detail, 1,541 genes were “private”, i.e., only detected in the genome of a single individual out of the 15 resequenced (16, if we include *Lola*). 914 genes (17.29%) were found in two individuals, 663 (12.54%) were found in three individuals and 2,194 (41.59%) were found in four or more individuals (**Fig. S99**). Only 25 genes (0.55%) were present in all resequenced genomes, but absent in *Lola*. This distribution closely recalls the one previously observed for the *in silico* validation of PAV with RNA-seq data (**Data Note 13**).


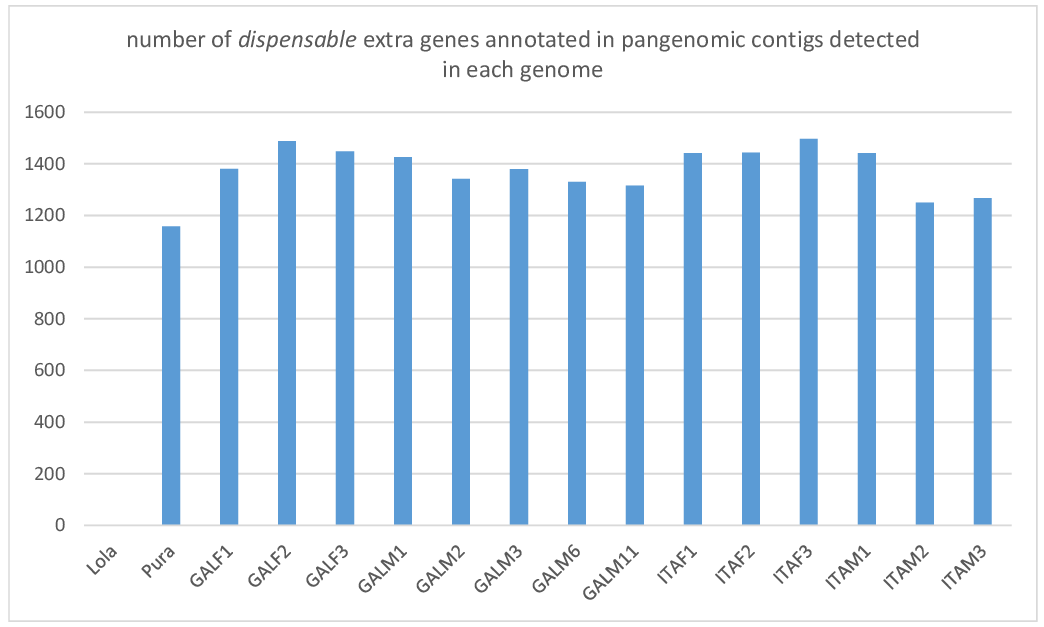


**Fig. S98. Number of *dispensable* genes, annotated in the *de novo* recursively reassembled pan-genomic contigs, detected in each genome.**


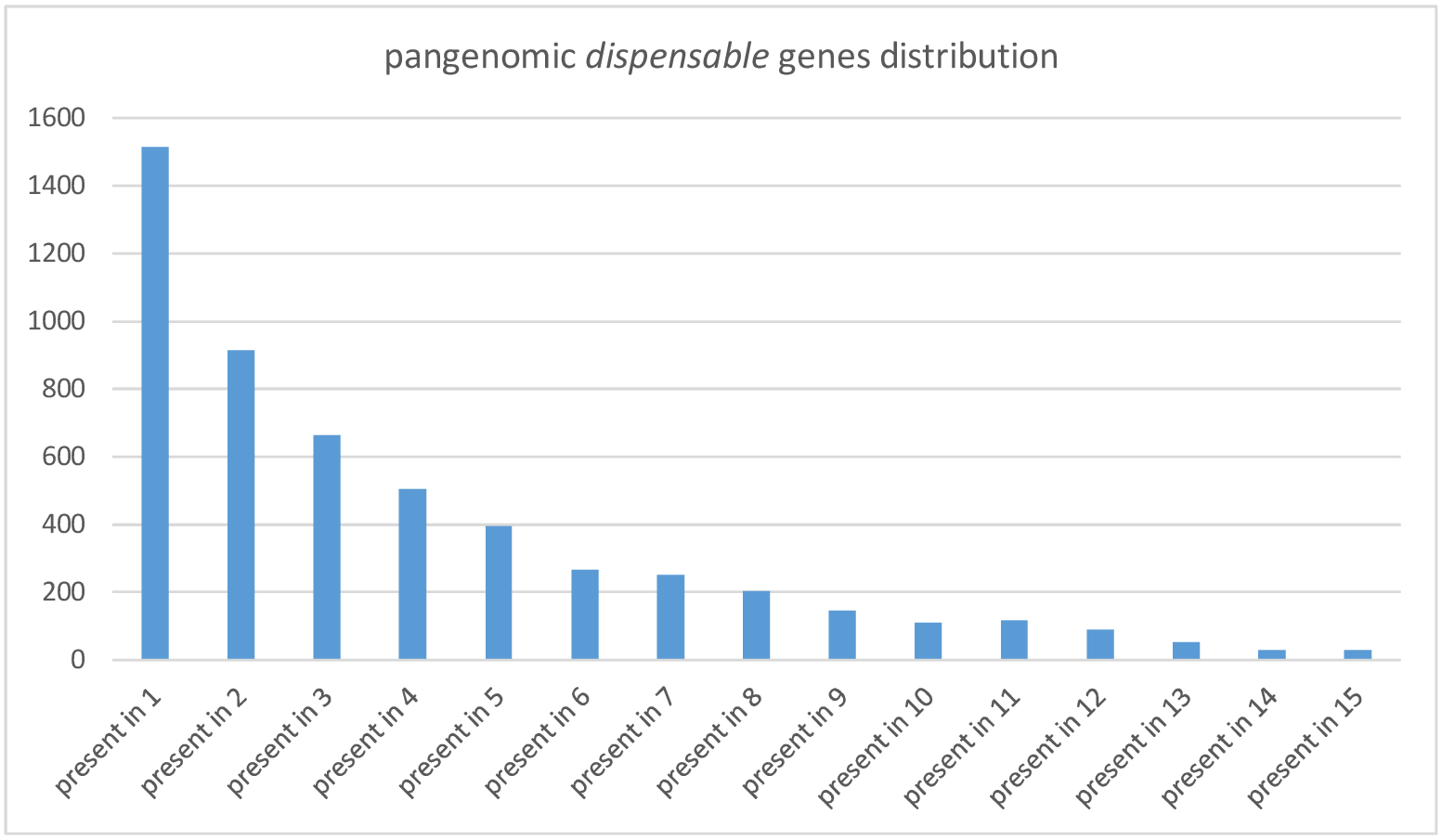


**Fig. S99. Distribution of pan-genomic *dispensable* genes in resequenced genomes.** Note that all these genes ae absent in *Lola*, as verified by the absence of mapped reads.

The high number of “private *dispensable* genes, as well as the high number of hypothetical full-length transcripts encoded by genes absent in the genomes of *Lola* and all the 15 resequenced individuals (**Fig. S64**) further support the idea that the mussel pan-genome might be significantly larger and that the inclusion of additional individuals from other populations might lead to a further increase in size.

The distribution of “private” *dispensable* genes identified in the recursively reassembled pan-genomic contigs is displayed in **Fig. S100**. The highest number of such private genes was observed in ITAF3 (148 genes), and the lowest in *Pura* (36 genes). The low number of observations in *Pura* is most likely linked with the lower quality (and lower read length) of the sequencing data obtained for this individual, targeted by a previous approach [17]. The presence of the highest number of private genes in ITAF3 might still result from the presence of residual contamination from the hydrozoan contamination (e.g. we can expect this to occur in the case of chimeric contigs, showing significant BLAST hits vs *Lola*, and coverage and GC content within the expected ranges. Nevertheless, if present, we expect such residual contamination to be neatly negligible, as the number of private dispensable genes in ITAF3 just exceeds the mean value of all resequenced individuals by about 50 units.

**Fig. S100. Number of “private” *dispensable* pan-genomic genes per resequenced individual.** This graph is based on the genes annotated in the reassembled pan-genomic contigs only.

Unlike the *dispensable* genes found in *Lola* (**Fig. S153**) and those identified with RNA-seq data mapping (**Fig. S65**), the presence-absence matrix of the *dispensable* genes identified in the recursively reassembled pan-genomic contigs displayed a very weak correlation with the geographical origin of the resequenced mussels, based on the results of a phylogenetic analysis carried out with MrBayes [127], with two independent MCMC analyses run in parallel for 100,000 generations. The obtained tree displayed very short branches, with many politomies and nodes supported by low posterior probability (data not shown)

The normalized per gene coverages of all the 5,286 re-annotated genes in the different genomes are reported in Supplementary **Figures S101-S104**. The values reported are normalized on the expected coverage of a gene present in a single allele in the diploid genome, i.e., the coverage of a *dispensable* gene included in a heterozygous genomic region. The graphs clearly highlight that, while most such genes were absent in individual genomes (in line with the data reported in **Fig. S99**), whenever present they nearly invariably displayed a normalized coverage close to 1, indicating their inclusion in genomic regions subject to hemizygosity. This was less evident in the genomes of the male mussels subject to a higher degree of coverage skew due to the extraction of DNA from mantle tissue (see **Data Note 23**). In particular, see **Fig. S104** for **ITAM2** and **ITAM3**.

**Fig. S101. Per gene coverage of the new genes annotated in the recursively reassembled pan-genome contigs for GALF1-3 and *Pura*.** Coverage was normalized on the expected haploid mussel genome size, calculated based on the mapping of Illumina PE libraries obtained from the mantle tissue of (A) GALF1, (B) GALF2, (C) GALF3 and (D) *Pura*.

**Fig. S102. Per gene coverage of the new genes annotated in the recursively reassembled pan-genome contigs for GALM1, GALM2, GALM3, GALM6 and GALM11.** Coverage was normalized on the expected haploid mussel genome size, calculated based on the mapping of Illumina PE libraries obtained from the gill tissue of (A) GALM1, and from the mantle tissue of (B) GALM2, (C) GALM3, (D) GALM6 and (E) GALM11.

**Fig. S103. Per gene coverage of the new genes annotated in the recursively reassembled pan-genome contigs for ITAF1, ITAF2 and ITAF3.** Coverage was normalized on the expected haploid mussel genome size, calculated based on the mapping of Illumina PE libraries obtained from the mantle tissue of (A) ITAF1, (B) ITAF2 and (C) ITAF3.

**Fig. S104. Per gene coverage of the new genes annotated in the recursively reassembled pan-genome contigs for ITAM1, ITAM2 and ITAM3.** Coverage was normalized on the expected haploid mussel genome size, calculated based on the mapping of Illumina PE libraries obtained from the gill tissue of ITAM1 (A), and from the mantle tissue of (B) ITAM2 and (C) ITAM3.

The median normalized coverage of the newly annotated genes found in pan-genomic contigs was also calculated (**Fig. S105**). To disregard the high proprortion of individuals where such genes were absent (**Fig. S99**), the median was calculated based on their average coverage in the individuals where each gene was marked as “present”. This graph clearly shows that, whenever present, these genes displayed in the vast majority of cases a normalized coverage close to 1, indicating their presence in hemizygous genomic regions, which is fully consistent with their *dispensable* nature. As expected, a minor “shoulder” was observed towards lower coverages, which can be most likely explained by crossmapping among similar variants (see **Data Note 10**). On the other hand, a very few genes displayed a median coverage equal to 2 (which would indicate the presence in a heterozygous region), or higher than 2 (which would indicate the presence of multiple paralogous copies). Even though this is a possibility that has been empirically observed in a few cases for the *dispensable* genes found in *Lola* (see **Data Note 17**), we found that just a very minor fraction of the pan-genomic *dispensable* genes (1.63% of the total) fell within this category (**Fig. S105**).

**Fig. S105. Median normalized sequencing coverage of the *dispensable* genes identified in the recursively reassembled pan-genomic contigs.** Coverage was normalized on the expected haploid mussel genome size, calculated based on the mapping of Illumina PE libraries, either from the mantle or from the gill tissue, depending on the individual taken into account.

**Figure 106** displays four examples consistent with the observations reported above. The four selected genes were found with differentfrequency in the resequenced individuals: PAV1A005939 was identified in 12 individuals, PAV1A002922 in 10, PAV1A007694 in 4 and PAV1A008253 in just 3 individuals. Nevertheless, whenever present, they displayed a sequencing coverage consistent with the presence of a single allele, indicating their association with hemizygous genomic regions.

**Fig. S106. Normalized sequencing coverage (i.e. predicted number of alleles) of four *dispensable* genes identified in the recursively reassembled pan-genomic contigs, in the resequenced individuals.** Panel A: PAV1A005939, found in 12 individuals; panel B: PAV1A002922, found in 10 individuals; panel C: PAV1A007694, found in 4 individuals; panel D: PAV1A008253, found in 3 individuals.

## Functional enrichment analysis

The *dispensable* genes found in the newly assembled pan-genomic contigs were functionally annotated following the pipeline described in detail in **Data Note 2**.

We then explored whether the the distribution of the observed Interpro annotations in this gene set, in terms of the most abundant conserved domains, was comparable with the situation highlighted in the *dispensable* gene set of *Lola* (**Data Note 18**). The top 30 most abundant Interpro domains (based on unique number of contigs, i.e., removing multiple hits within the same contig) are reported in **Table S48**.

**Table S48**. Top 30 most abundant Interpro domains in the set of *dispensable* genes annotated in the *de novo* recursively reassembled pan-genomic contigs.

| **Domain ID** | **number of annotated genes** |
| --- | --- |
| P-loop containing nucleoside triphosphate hydrolase | 767 |
| Immunoglobulin-like fold | 622 |
| Ankyrin repeat-containing domain | 604 |
| Ankyrin repeat | 555 |
| Six-bladed beta-propeller, TolB-like | 540 |
| Ankyrin repeat region circular profile. Ankyrin repeat-containing domain | 536 |
| Ankyrin repeat profile. Ankyrin repeat | 519 |
| Ankyrin repeats (3 copies) Ankyrin repeat-containing domain | 489 |
| Immunoglobulin-like domain | 487 |
| Zinc finger B-box type profile. B-box-type zinc finger | 450 |
| Ig-like domain profile. Immunoglobulin-like domain | 412 |
| Immunoglobulin subtype | 299 |
| Tumour necrosis factor-like domain | 291 |
| C1q domain C1q domain | 253 |
| B-box-type zinc finger | 232 |
| Ankyrin repeats (many copies) | 199 |
| Mab-21 protein Mab-21 domain | 197 |
| Death-like domain | 193 |
| Immunoglobulin subtype 2 | 175 |
| Fibrinogen, alpha/beta/gamma chain, C-terminal globular domain | 169 |
| Fibrinogen beta and gamma chains, C-terminal globular domain Fibrinogen, alpha/beta/gamma chain, C-terminal globular domain | 166 |
| Fibrinogen C-terminal domain profile. Fibrinogen, alpha/beta/gamma chain, C-terminal globular domain | 158 |
| Fibrinogen, alpha/beta/gamma chain, C-terminal globular, subdomain 1 | 154 |
| Mab-21 domain | 151 |
| Immunoglobulin domain | 146 |
| Fibronectin type III | 144 |
| Fibrinogen, alpha/beta/gamma chain, C-terminal globular, subdomain 2 | 136 |
| Complement C1Q domain signature C1q domain | 134 |
| Fibronectin type-III domain profile. Fibronectin type III | 125 |
| C-terminal of Roc, COR, domain C-terminal of Roc (COR) domain | 108 |

We observed a significant linear correlation between the abundances of these conserved domains in the *dispensable* genes found in *Lola* and in the pan-genome reassembly (R^2^ = 0.7118, see **Fig. S107**). This observation confirms that the accessory genes identified with our recursive reassembly approach (**Data Note 14**) were not technical artefacts. We did however notice a few significant differences in terms of under-representation of some specific domains, which included the C1q globular domain, the Fibrinogen-like C-terminal domain, and others. We suspect that this discrepancy may be due to the relatively high number of fragmented genes found in reassembled contigs (see **section 15.1**), with the consequent under-representation of conserved domains which typically display significant lengths.

In support of this hypothesis, we re-calculated domain abundances for full-length genes only (**Table S49**) ad computed once again the linear correlation between these observations and those collected from *Lola* *dispensable* genes, reveling an increase in the R^2^ metric (**Fig. S108**).

**Fig. S107. Correlation between the abundance of the Interpro domains annotated in *dispensable* genes.** This graph compares the abundances of conserved domains in the *dispensable* genes identified in *Lola* and those found in the recursively reassembled pan-genomic contigs.

**Table S49**. Top 30 most abundant Interpro domains in the set of *dispensable* genes annotated in the *de novo* assembled pan-genomic contigs. This table only takes into account annotations linked with genes labeled as “complete”.

| **Domain ID** | **number of annotated genes** |
| --- | --- |
| P-loop containing nucleoside triphosphate hydrolase | 188 |
| Ankyrin repeat-containing domain | 147 |
| Ankyrin repeat | 139 |
| Ankyrin repeat region circular profile. | 135 |
| Immunoglobulin-like fold | 128 |
| Ankyrin repeat profile. | 127 |
| Ankyrin repeats (3 copies) | 124 |
| Immunoglobulin-like domain | 93 |
| Ig-like domain profile. | 72 |
| Six-bladed beta-propeller, TolB-like | 71 |
| B-box-type zinc finger | 53 |
| Zinc finger B-box type profile. | 53 |
| AIG1 family | 51 |
| AIG1-type guanine nucleotide-binding (G) domain | 51 |
| Ankyrin repeats (many copies) | 51 |
| Immunoglobulin subtype | 46 |
| Ankyrin repeat signature | 46 |
| Fibronectin type III | 35 |
| Immunoglobulin domain | 31 |
| AIG1-type G domain profile. | 31 |
| Very large inducible GTPASE (VLIG)-type guanine nucleotide-binding (G) domain | 27 |
| Very large inducible GTPASE (VLIG)-type guanine nucleotide-binding (G) domain profile. | 27 |
| Fibronectin type-III domain profile. | 25 |
| Immunoglobulin subtype 2 | 23 |
| B-box zinc finger | 21 |
| Death-like domain | 20 |
| Mab-21 domain | 18 |
| Mab-21 protein | 18 |
| WD40/YVTN repeat-like-containing domain | 16 |
| Immunoglobulin I-set | 15 |

**Fig. S108. Correlation between the abundance of the Interpro domains annotated in *dispensable* genes.** This graph compares the abundances of converved domains in the *dispensable* genes identified in *Lola* and those found in the recursively reassembled pan-genomic contigs. This graph only taken into account the genes labeled as “complete” in the pan-genome reassembly.

# Data Note 16 –Expression levels of *core* and *dispensable* genes

## Experimental setup

The data generated for the assessment of mussel gene model support and reported in **Data Note 4** was also used to evaluate whether any significant difference existed between the expression levels of genes predicted to be part of the *core* set of the mussel genome and *dispensable* genes.

Subsequently, we separately addressed the expression levels of *core* and *dispensable* *M. galloprovincialis* genes, limiting aor analysis to protein-coding genes. In detail, we computed the expression levels of 14,570 mussel genes subject to PAV and compared these with the data obtained from 45,768 genes invariably present in all the 15 analyzed genomes (14 resequenced in the present study, plus *Pura*). This analysis was aimed at evaluating the functionality of *dispensable* genes, under the assumption that actively transcribed genes are likely to retain a biological function.

In detail, genes achieving a maximal TPM < 1 were considered as poorly supported by transcriptomic evidence. Genes with maximal TPM between 1 and 3 were considered as mildly supported by transcriptomic evidence. Genes whose maximal expression level exceeded 3 TPM were considered as strongly supported by transcriptomic evidence.

## Overview of gene expression levels of *core* and *dispensable* genes

Overall, *dispensable* protein-coding genes displayed expression values significantly lower than that of *core* genes, as 41.65% of genes subject to PAV was supported by poor transcriptomic evidence (19.51% by mild evidence), compared to the lower fraction of *core* genes supported by similar evidence score (23.15% by low, 15.15% by mild evidence) (**Fig. S109**). An unpaired t-test, carried out by taking into account the maximum expression level observed across the 51 RNA-seq datasets for each gene, revealed that the difference in the mean expression values observed for *core* genes (128.09 TPM) and *dispensable* genes (35.35 TPM), equal to 3.62-folds, was supported by a highly statistically significant p-value (<0.0001).

**Fig. S109**. **Percentage of genes showing a gene expression level below a given threshold (considering increasing threshold values up to the maximal expression level observed in the 51 analyzed samples**). The graph separately reports *core* genes, *dispensable* genes and all genes annotated in *Lola*.

The lower expression of *dispensable* genes compared to *core* genes could be also detected by the inspection of median, 1^st^ and 3^rd^ quartile of gene expression levels (considering the maximum expression level observed across the 51 RNA-seq datasets) (**Fig. S110**), given that:

1. The median expression value of *core* genes was 3.90-fold higher than *dispensable* genes
2. The 1^st^ quartile of expression value of *core* genes was 3.20-fold higher than *dispensable* genes
3. The 3^rd^ quartile of expression value of *core* genes was 3.80-fold higher than *dispensable* genes

**Fig. S110**. **Gene expression levels of *core* and *dispensable* genes from RNA-seq.** Comparative overview of mean, median. 1^st^ and 3^rd^ quartile of gene expression values of *core* and *dispensabl*e genes, compared to all *Lola* genes, based on their maximal expression value observed in the 51 RNA-seq datasets considered in this study.

## Gene expression levels of *core* and *dispensable* genes in *Lola*

Similar results, with significantly higher mean and median expression values, could be also observed in the RNA-seq data obtained from *Lola* digestive gland and gill tissues (**Fig. S111**), indicating that the lower expression levels observed for genes subject to PAV cannot be simply explained by an underestimation linked to their lower frequency of occurrence in mussel populations (see main text).

**Fig. S111**. **Gene expression levels of *core* and *dispensable* gene from *Lola*.** Comparative overview of mean, median, 1^st^ and 3^rd^ quartile of gene expression values of *core* and *dispensable* genes, compared to all *Lola* genes, based on gene expression profiles obtained from *Lola* gills and digestive gland.

The detailed analysis of gene expression levels in *Lola* revealed that just four and two *dispensable* genes were included in the top100 most highly expressed genes in *Lola* gills and digestive gland, respectively, and 94 and 95 *dispensable* genes were included in the top1000 (**Table S49**).

**Table S49**. **Top 12 expressed *dispensable* genes in *Lola* digestive gland and gills**. “rank” represents the ranking of the gene, when gene expression levels of all genes (*core* + *dispensable*) are ordered from the most to the least expressed.

| **Digestive gland** | | | **Gills** | | |
| --- | --- | --- | --- | --- | --- |
| **Gene ID** | **Expression level (TPM)** | **Rank** | **Gene ID** | **Expression level (TPM)** | **Rank** |
| MGAL10A003200 | 4.013.55 | 39 | MGAL10A003200 | 4.846.88 | 46 |
| MGAL10A053997 | 2.578.82 | 78 | MGAL10A068752 | 3.195.95 | 72 |
| MGAL10A066945 | 1.424.15 | 114 | MGAL10A053997 | 2.811.91 | 79 |
| MGAL10A082667 | 1.423.72 | 115 | MGAL10A077590 | 2.436.27 | 93 |
| MGAL10A032349 | 1.297.43 | 121 | MGAL10A094625 | 1.673.77 | 108 |
| MGAL10A094625 | 1.193.26 | 127 | MGAL10A032349 | 1.669.61 | 109 |
| MGAL10A052780 | 841.78 | 167 | MGAL10A051171 | 1.400.16 | 121 |
| MGAL10A051171 | 829.30 | 169 | MGAL10A082667 | 1.204.33 | 138 |
| MGAL10A027444 | 544.37 | 220 | MGAL10A001102 | 792.78 | 186 |
| MGAL10A077590 | 527.67 | 224 | MGAL10A066945 | 719.50 | 193 |
| MGAL10A069761 | 493.37 | 234 | MGAL10A017128 | 674.34 | 202 |
| MGAL10A013415 | 482.20 | 237 | MGAL10A039699 | 623.87 | 211 |

## Contribution of *dispensable* genes to transcriptional activity

These observations are coherent with previous data collected in plants, like the potato *Solanum tuberosum*, where *dispensable* genes have been associated to limited transcription [137]. While it remains to be established whether this data may be linked to a progressive loss of function and pseudogenization of genes subjected to PAV, a number of *dispensable* genes most definitely retain functionality (**Data Note 17**). This is as also suggested by the high expression levels reached by some *dispensable* genes in *Lola* and other RNA-seq datasets, such as MGAL10A053997, encoding a C-type lectin highly expressed in *Lola* digestive gland and gills.

Overall, we could estimate that the transcription of *dispensable* protein-coding genes involves between 3 and 10% of the global transcriptional efforts in *M. galloprovincialis*, depending on the tissue and gene expression dataset considered (**Fig. S112**). Therefore, in spite of the identified mRNA levels attenuated if compared to *core* genes, mussel *dispensable* genes contribute to a non-negligible amount of the transcriptional activity and, consequently, to a noticeable use of energetic investment.

**Fig. S112**. **Global contribution of *dispensable* genes to gene expression in different RNA-seq dataset**. Each bar (and number) indicates a row of the datasets indicated in **Table S11** (the samples follow the same order in the histogram and in the table).

# Data Note 17 – Structural features of PAV

## Genomic organization of *dispensable* gene clusters

We present here a few examples which might help to clarify the relationship between the PAV phenomenon and the structural organization of the mussel genome. First, consider the organization of protein-coding gene models annotated in two relatively small genomic scaffolds (mg10_s02822. ~136 Kb long, and mg10_s05693, ~64 Kb long) (**Fig. S113)**.

**Fig. S113**: **Gene models annotated in the genomic scaffolds mg10_s02822 (A) and mg10_s05693 (B), respectively.**

Based on mapping data (**Data Note 8**), the average sequencing coverage was calculated and normalized with the expected coverage of the haploid genome, and calculated for each gene in *Lola, Pura* and the 14 resequenced mussel genomes (**Table S1**), obtaining an approximate estimate of the number of times any given sequence was found in any genome. Namely, a normalized average coverage lower than 0.25 was considered as evidence of “absence”, based on the criteria listed in **Data Note 8**. Genes displaying a normalized coverage close to 1 can be considered as likely to be present in hemizygous regions (i.e., found in only one out of the two homologous chromosomes), whereas those with coverage = 2 are most likely *core* genes found in two copies in the genome. Higher levels of coverage might indicate copy number variation. The results for the two scaffolds are summarized, as histograms, in **Fig. S114** and **Fig. S115**. In these examples, scaffold mg10_s05693 (**Fig. S114** and detailed in **Figure 2D** in the main text) presents a single *core* gene. i.e., MGAL10A011823 annotated as an alkaline phosphatase, which indeed displays a quite uniform normalized coverage (close to 2) in all genomes, consistent with the presence of two nearly identical allelic variants in the diploid genome. The 19 remaining gene models contained in this scaffold (**Table S50**) were all subject to PAV, as they were absent in 12 genomes. Curiously, when they were present, these genes were either detected with average coverage close to 1 (i.e., in *Lola*), indicating the presence of a single allele in the diploid genome, or with multiple copies (3/4 in *Pura*, 4/8 in GALM1 and 6/9 in GALM6). These results indicate that a large portion of this scaffold, encompassing 19 protein-coding genes comprised in a 50 Kb-large genomic region, might be subject to haplome-specific deletion, i.e., hemizygosity. This deletion event was balanced in most genomes, as evidenced by the absence of the entire block of PAV genes, and unbalanced in *Lola*, where a single gene allele was observed. On the other hand, the high coverage calculated in *Pura*, GALM1 and GALM6 indicates the possibility that, whenever present, the entire region or some portions of it are likely to have underwent complex events of duplication. However, the fragmented nature of the resequenced assemblies (**Table S41**), as well as the lack of long reads to improve scaffolding (as explained in detail in **Data Note 14**), did not allow to investigate in depth the nature of the structural variations at this stage.

**Table S50.** Annotation of the 20 genes located in the genomic scaffold mg10_s05693

| **Locus ID** | **Annotation** |
| --- | --- |
| MGAL10A041721 | Putative E3 ubiquitin-protein ligase |
| MGAL10A025495 | Unknown |
| MGAL10A021236 | Unknown |
| MGAL10A038341 | Ribonucleoside-diphosphate reductase small chain |
| MGAL10A006169 | Baculoviral IAP repeat-containing protein |
| MGAL10A040493 | Baculoviral IAP repeat-containing protein |
| MGAL10A046398 | Unknown |
| MGAL10A002899 | Unknown |
| MGAL10A052538 | unknown |
| MGAL10A016640 | Unknown |
| MGAL10A080632 | Unknown |
| MGAL10A050728 | Unknown |
| MGAL10A069289 | Unknown |
| MGAL10A086703 | Unknown |
| MGAL10A046961 | Baculoviral IAP repeat-containing protein |
| MGAL10A008710 | Tripartite terminase subunit 3 |
| MGAL10A090554 | Unknown |
| MGAL10A069343 | Unknown |
| MGAL10A044743 | Unknown |
| MGAL10A011823 | Alkaline phosphatase, tissue-nonspecific isozyme |

In the second example, scaffold mg10_s02822, PAV events are also quite evident for all the eight genes encoded (**Table S51**), paired with rare copy number variation events, e.g., for the MGAL10A030648 and MGAL10A014001 genes (both found with 0 to 6 alleles) (**Fig. S115**). Curiously, the entire scaffold was missing in ITAM2, and almost completely missing (with the exception of MGAL10A090248) in GALM2 and GALM3. *Lola* displays a coverage consistent with the presence of 4 alleles in the diploid genome for all the genes of this scaffold, with the exception of MGAL10A048191, which shows relevant fluctuations in the number of alleles across all genomes.

**Table S51. Annotation of the 8 genes located in the genomic scaffold mg10_s02822**

| **Locus ID** | **Annotation** |
| --- | --- |
| MGAL10A014001 | Unknown |
| MGAL10A021937 | Histone-lysine N-methyltransferase |
| MGAL10A029989 | Unknown |
| MGAL10A030648 | Unknown |
| MGAL10A048191 | Unknown |
| MGAL10A067925 | Unknown |
| MGAL10A074646 | Unknown |
| MGAL10A090248 | Unknown |

Altogether, these examples indicate that PAV events are not limited to isolated genes (i.e., to relatively small genomic regions), but they can involve relatively large regions (up to 50 Kb in size, as in the case of scaffold 05693). Moreover, PAV events cannot be considered just as the result of unbalanced indels, but they might on some occasions involve the duplication of *dispensable* genes, as in the cases shown in **Fig. S114** and **S115**. However, as previously shown, these considerations cannot be extended to the majority of *dispensable* genes that, whenever present, usually show a hemizygous normalized coverage (i.e., the presence of only a single allele in the diploid genome) (**Data Note 10**).

**Fig. S114**. **Sequencing coverage of the gene models contained in scaffold 05693, normalized to the expected size of the haploid mussel genome**.

**Fig. S115. Sequencing coverage of the gene models contained in scaffold 02822, normalized to the expected size of the haploid mussel genome**.

## An example: mytilin K

To further illustrate the association between *dispensable* genes and indels, we present here the case of mytilin K, an atypical sequence pertaining to the a multigenic family of antimicrobial peptides, named mytilins [138,139]. Unlike “canonical” mytilins, largely expressed in mussel hemocytes, this sequence was first described in 2015 as a mRNA seldom found in a few RNA-seq datasets [140]. Indeed, mytilin K was found to be a *dispensable* gene, absent in *Lola*, but present in one of the resequenced genomes, GALM1 (**Data Note 12**), and confirmed as a *dispensable* gene by PCR analysis (**Figures S58-S62**). All canonical mytilins (i.e., mytilin B, C, D and G1) appear to be *core* genes, located in a 35 Kb long genomic region, along with two pseudogenes (mytilin J and mytilin P), within scaffold mg10_s00171, in a cluster organization, suggesting a common origin for all mytilin genes from an ancestral gene by multiple duplication events, as also recently evidenced by a recent study [139] (**Fig. S116**). The inspection of the *de novo* genome assembly obtained from GALM1 (**Table S41**) revealed that the mytilin K gene is part of a large insertion of at least 11 Kb in size, located in a genomic region found within the intron 3 of the mytilin G1 gene, leading the facto to the disruption of this gene. The entire indel region displays a hemizygous coverage, indicating that the mytilin K sequence is only carried by one out of the two homologous chromosomes. A fully functional mytilin G1 gene is expected to be retained by the second chromosome, which is expected to present a canonical mytilin cluster, as confirmed by the homozygous normalized coverage of all the other mytilin genes and by the *de novo* assembly of a full mytilin G1 gene sequence on top of this truncated variant (**Fig. S116**).

**Fig. S116. Mytilin gene cluster**. Located on scaffold 00171, includes the protein-coding genes mytilin C, mytilin D, mytilin B and mytilin G1, and the two pseudogenes mytilin J and mytilin P. The example shows the genomic organization of this gene cluster in *Lola*. Mytilin K is a *dispensable* gene, first identified by the analysis of transcriptome data, which was only found in one of the resequenced individuals, GALM1. This gene appears to be encoded by an 11 Kb region inserted within exon 3 of the mytilin G1 gene, as exemplified by the lower panel of this figure.

Mytilin K appears to be a functional gene, as supported by its strong expression in RNA-seq datasets, e.g., in one of the two hemocyte samples subjected to RNA-seq in a recent study by Moreira *et al*. [18]. Moreover, the promoter region of mytilin K is highly conserved (**Fig. S117A**): its main structural motif (which consensus is displayed in **Fig. S117B**), surrounding the TATA box, is virtually indistinguishable from that of the other highly expressed genes mytilin B, C, D and G1. Its placement with respect to the transcription start site is consistent with that of other mytilins and, together with evidence of expression collected by RNA-seq data, clearly points out that mytilin K is an actively transcribed gene.

**Fig. S117. Multiple sequence alignment of the promoter regions of mytilins.** Showing mytilin B, C, D and G1 (obtained from the *Lola* mg10 assembly) and mytilin K (obtained from the *de novo* assembly of the GALM1 genome) (panel B). This motif was *de novo* discovered using MEME [141] and its consensus is shown in panel B. The motif was detected with the following p-values: mytilin G1 1.92e-26, mytilin D 1.61e-25, mytilin B 2.26e-25, mytilin C 4.75e-25, mytilin K 1.16e-24.

## Coding sequence features of *core* and *dispensable* genes

We also evaluated whether *dispensable* genes showed significant differences in terms of codon usage compared to *core* genes. In detail, the Open Reading Frame sequences of four different sets of mussel genes were extracted and analyzed with the Emboss *cusp* tool [142], to calculate the frequency of usage for each of the 64 codons in protein-coding sequences and, consequently, the Relative Synonymous Codon Usage (RSCU) values [143]. Namely, the four sets of mussel genes used were:

1. All genes
2. *core* genes
3. *dispensable* genes
4. expressed *core* genes, i.e., displaying TPM > 3 (see **Data Note 16**)

The computed matrix of RSCU values was added to the dataset from the study by Gerdol *et al.* [144], who collected codon usage data from 64 different bivalve species, based on transcriptome data, i.e., on TransDecoder (a tool part of the Trinity suite) ORF predictions [145]. This dataset also included codon usage data from the *M. galloprovincialis* transcriptome, which was also considered in this case.

As shown in **Fig. S118**, which includes the hierarchical clustering of bivalves based on codon usage (RSCU values), no relevant differences could be observed between the four gene sets, even though their codon usage appeared to be somewhat different from the estimates obtained from transcriptome data only. This slight discrepancy can be most likely explained by the inclusion of some biases in the prediction of gene models (e.g., the use of a minimal ORF length, as well as some incorrect predictions), which may have resulted in minor alterations of RSCU calculations.

Based on the same genes sets, we also calculated (i) the general GC content of ORFs and (ii) the GC content for the first, second and third codon position with the Emboss cusp tool [142], to investigate the possible presence of significant differences between *core* and *dispensable* genes. As shown in **Fig. S119**, *core* and *dispensable* genes displayed a highly similar GC content in all codon positions, regardless of their level of expression. On the other hand, the ORFs of *core* genes were significantly longer (T-test p-value < 0.000001) than those of *dispensable* genes, achieving an average length of 1,635 nucleotides, compared to 1,170 nucleotides of *dispensable* genes. The median length of the ORFs of *core* and *dispensable* genes were 1,108 and 807 nucleotides, respectively (**Fig. S120**).

**Fig. S118**. **Hierarchical clustering of bivalve species according to the variation of codon usage**. Five different sequence sets are represented for *M. galloprovincialis*: (i) all genes, (ii) *core* genes, (iii) *dispensable* genes, (iv) expressed *core* genes, i.e., those displaying an expression level > 3 TPM in at least one of the available RNA-seq datasets (**Data Note 16**) and (v) *de novo* assembled transcripts, as reported in Gerdol et al. 2015 [144].

**Fig. S119**. **GC content of Open Reading frames, first, second and third codon position of mussel genes**. Separately calculated for (i) all genes, (ii) *core* genes, (iii) *dispensable* genes and (iv) expressed *core* genes, i.e., those displaying an expression level > 3 TPM in at least one of the available RNA-seq datasets (**Data Note 16**).

**Fig. S120. Mean and median length of Open Reading Frames of mussel *core* and *dispensable* genes.**

*Dispensable* genes also showed a considerably lower complexity in terms of genomic architecture, as clearly evident from the analysis of average and median number of exons per gene (10.52 and 4 in *core* genes, vs 4.53 and 2 in *dispensable* genes, respectively) (**Fig. S121**).

**Fig. S121. Mean and median number of exons per gene of *core* and *dispensable* genes.**

Overall, these results, together with the evaluation of gene expression levels reported in **Data Note 16**, suggest that mussel *dispensable* genes have peculiar structural features compared to *core* genes. While they do not display any significant difference in terms of GC content and codon usage, they are subject to a narrower range of expression, which is on average lower than *core* genes, and display on average a shorter ORF length and lower degree of complexity of genomic architecture.

## Assessment of the presence of transposable elements in the genomic regions neighboring PAV genes

We investigated whether the presence of the variable gene content in different individuals could be explained by the presence and activity of transposable elements. Although the fragmentation of the mg10 genome assembly hampers a proper study of the genomic repeat content, we checked if any of the repeats included in the custom repeat library (see **Data Note 2**) was enriched in the regions surrounding *dispensable* genes, especially, when the gene next to them was a *core* gene. For this, we extracted the annotated transposable elements in regions surrounding genes, hereafter on called *boundaries*, dividing them in four categories:

1. *core-core* boundaries,
2. *core-dispensable* boundaries
3. *dispensable-core* boundaries
4. *dispensable-dispensable* boundaries

Several lengths for the boundaries were also used. For instance, we checked regions 2 Kb, 5 Kb and 7 Kb upstream and downstream of target genes. We stopped at 7 kb because the median distance between genes in the genome annotation was 6,125bp. Hence, if any mechanism responsible for making genes jump through genomes exists, it is likely present in this surrounding genomic region.

We did not find any significant increase of transposable element presence (and therefore activity) between the different type of boundaries, which in all cases was around 12-13%. Also, we did not find presence of any  family or repeats whose occurrence significantly increased in boundaries between *core* and *dispensable* genes. Despite our negative results, we believe that this aspect deserves more attention, and that a definitive answer to this question will be only made possible by the release of a chromosome-scale genome assembly.

# Data Note 18 – Functional enrichment of *dispensable* genes

## Detection of significantly over-represented annotations

The annotations associated with the mussel *dispensable* protein-coding genes identified in **Data Note 8** were subjected to a functional enrichment test with hypergeometric tests [146]. Namely, Pfam conserved domains, Gene Ontology cellular component (CC), molecular function (MF) and biological process (BP) annotations were separately analyzed and the obtained enrichment p-values were corrected for multiple testing using the False Discovery Rate method as proposed by Benjamini and Hochberg [147]. Annotations were considered as significantly over- or under-represented for FDR-corrected p-values lower than 0.05 and negative or positive difference between observed and expected observations, respectively. Log_2_-transformed FDR-corrected p-values were subsequently plotted against the total number of observations for each annotation (in the full mussel genome), to obtain Volcano plots. Overall, the analysis of Pfam conserved protein domains (see **Figure 4A** in the main text and **Additional file 2:** **Table S52-S53**) was the one providing the most interesting results, evidencing 71 over-represented annotations and 117 under-represented annotations.

## Over-represented domains

Here we provide a brief overview on the most outstanding domains found to be significantly associated with *dispensable* genes and, consequently, with the PAV phenomenon (**Additional file 2:** **Table S52**). The most significantly enriched annotations were clearly linked to domains associated with the immunoglobulin-like fold and with ankyrin-like repeats, which were often found to be associated within the same predicted proteins. The immunoglobulin fold is one of the most successful and evolutionarily widespread structural motifs found in nature [148,149], and it is commonly found in a plethora of diverse proteins, in association with multiple domains, serving as a module for protein-protein interaction. The immunoglobulin domain is associated with immune functions in the vertebrate adaptive immune system, as it serves as a fundamental and sophisticated antigen recognition module in immunoglobulins and TCRs [150]. While the function of the immunoglobulin domain has not been clearly linked with immune recognition in bivalves to date, several hundred genes encoding proteins bearing this domain have been reported in the oyster genome [42]. Moreover, besides the implication if the somatic diversification of the Ig domain of FREPs in immune defense in gastropods [151], recent evidence supports the possible role of the immunoglobulin domain in PAMP recognition in bivalve mollusks as well [152]. Curiously, the topologically similar, but evolutionarily unrelated fibronectin-type III domain, also very common and involved in protein-protein interactions [153], was also highly over-represented.

Ankyrin repeats on the other hand, despite being extremely common in metazoans [154], and known to act as important mediators of protein-protein interaction, are not known to be involved as major players in recognition molecules part of the immune system [155].

The over-representation of proteins involved in protein binding is not limited to immunoglobulin domain and ankyrin repeats. Another well documentable example of such over-representation in the context of presence-absence variation is represented by the C1q domain/Tumor necrosis-like domain superfamily, which has been linked on multiple occasions to immune recognition, as a massively expanded gene family, in mussels and other bivalves [156]. Similarly, the fibrinogen C-terminal domain, characterizing the so-called FReD protein family and the main structural domain of gastropod FREPs, was also significantly more subject to PAV than expected. While in bivalves the fibrinogen C-terminal domain, unlike gastropod FREPs, is not linked to immunoglobulin domains, relevant cases of sequence hypervariability, possibly linked with immune recognition, have been reported [157].

The scavenger receptor cysteine-rich (SRCR) domain is another extremely widespread motif covering a multitude of diverse functions, and it has been most commonly studied within the context of protein-protein interaction and ligand binding [158].

Also, the highly over-represented domain Mab-21, expanded in bivalves, which has been recently linked to partners of STING in the detection of PAMPs in the cytosol, due to the potential activity of proteins bearing this domain as cyclic GMP-AMP synthase (cGAS) [159].

Diverse classes of immune-related GTPases were also found to be significantly over-represented within the set of *dispensable* genes. Namely, IMAP GTPases (characterized by the presence of the AIG1 domain), with over 100 members, emerge as interesting targets for further studies. IMAP GTPases have been implicated in various aspects of animal physiology and pathology, but most notably in the regulation of apoptosis in response to disease and in resistance to infection [160,161]. Similarly, interferon-inducible GTPases (IIGP) are fundamental mediators of cytoskeletal organization, vesicular trafficking and protein complex assembly in response to infection [162]. The ROC-COR tandem [163] was also found to be significantly associated with *dispensable* genes.

The B-box type zinc-finger domain, strongly enriched in the *dispensable* gene set, is frequently associated with proteins involved in ubiquitinylation [164]. Possibly linked with the alleged function of this domain, we could also observe the over-representation of the DEATH domain and of the connected CARD domain, which are primarily involved in cell-death-associated signaling, as well with other non-apoptosis related functions [165]. At the same time, also proteins containing BIR (baculovirus inhibitior of apoptosis protein repeat) repeats, usually characterizing proteins acting as negative regulators of apoptosis, were found to be over-represented [166].

The over-representation of many other conserved protein domains, most notably the Six-bladed beta-propeller (TolB-like), currently remains unexplained.

## Under-represented domains

Many protein domains were found to be under-represented in the set of *dispensable* genes (**Additional file 2: Table S53**). A large fraction of these domains was clearly linked to transposable elements, which are likely to be present with multiple nearly-identical copies in the mussel genome and which are, consequently, unlikely to be subject to PAV. In detail, the most under-represented domain was “Reverse transcriptase”, typical of retrotransposons [167], and several entries linked to C2H2-type zinc finger domains, typically associated with the activity of integrases (<https://www.ebi.ac.uk/interpro/entry/IPR000477>). Other domains identified as under-represented in the *dispensable* gene set were also associated with retroviral polyproteins, namely RNase H and aspartic protease domains [168,169]. The endonuclease/exonuclease/phosphatase domain might be also linked to the activity of transposable elements present in multiple copies in the mussel genome.

Besides domains associated with mobile elements, many other conserved motifs linked with housekeeping functions were found to be rarely associated with PAV. Most notably, these included protein kinase domains, G-protein coupled receptors (GPCRs) –in particular those similar to rhodopsins-, transporters of the Major Facilitator Superfamily (MFS) and NAD(P)-binding domains. Many other widespread structural motifs denoting repeats (e.g. WD40 repeats, armadillo-type fold repeats, EF-hand and C2H2-type zinc finger domains) were also prominent under-represented.

## Gene Ontology – Cellular component annotations

Hypergeometric tests identified only 20 enriched Gene Ontology cellular component annotations in the *dispensable* gene set. Out of these, only one (“intracellular) was over-represented, whereas the remaining 19 (**Additional file 2:** **Table S54**) were under-represented. These included basically all the other major cellular compartments (membrane, nucleus, cytosol, Golgi apparatus, ribosome, cytoskeleton), in line with the high expected evolutionary conservation of proteins bearing these annotations. The reasons behind the over-representation of annotations linked with the “intracellular” GO annotation remain to be investigated.

## Gene Ontology – Biological process annotations

A total of 21 Gene Ontology biological process annotations were found to be under-represented, and none over-represented in the *dispensable* gene set (Additional file 2: **Table S55**). All annotations were linked to housekeeping functions, often connected to conserved protein domains described above (**Additional file 2:** **Table S52**). Among these, transcription, translation and the associated processes of protein folding and degradation covered a prominent role. In addition, transmembrane transport (linked to the MSF family), GPCR signaling, protein phosphorylation (linked with protein kinase activity) and other key housekeeping processes were also rarely associated with PAV.

## Gene Ontology – Molecular function annotations

A total of 39 Gene Ontology molecular function annotations were found to be significantly enriched in the *dispensable* genes set, 36 under-represented and just three over-represented (**Additional file 2:** **Table S56**). The latter were “protein binding”, coherently with the over-representation of domains involved in protein-protein interactions, “scavenger receptor activity”, in line with the over-representation of the SRCR domain, and “GTP binding”, mirroring the over-representation of IMAP, interferon-inducible and other GTPases (**Additional file 2:** **Table S52**).

# Data Note 19 – Evolutionary considerations on *dispensable* genes

## Relationship between *dispensable* genes and gene duplication events

Based on the datasets generated in **Data Note 5**, we investigated the pairwise orthology status between *M. galloprovincialis* and the other metazoan species included in **Figure 1** (see main text). In detail, based on the number of orthologous genes identified, mussel sequences were classified within the following categories:

1. “one-to-one” orthologs
2. “one-to-many” orthologs (one gene in *Mytilus* that presents more than one ortholog in the other species)
3. “many-to-one” orthologs (several genes in *Mytilus* that present just one ortholog in the other species)
4. “many-to-many” orthologs (several genes in *Mytilus* that presents more than one ortholog in the other species)

As it can be observed in **Fig. S122**, *dispensable* genes tend to be mainly classified within the “many-to-one” orthologs category, pointing to a recent origin by lineage-specific gene duplication. The recent origin of genes subject to PAV can be inferred from the high rate of “many-to-one” orthologs observed in the comparisons between *M. galloprovincialis* and other mytilids, namely *L. fortunei*, *M. philippinarum* and *B. platifrons*, as opposed to the very limited number of “one-to -many” orthologs identified. This rate was also significantly higher than the background rate observed at the whole-genome level (where the size of the “many-to-one” category is negligible), pointing out a peculiar evolutionary situation for mussel *dispensable* genes.

In further support of the young origin of mussel *dispensable* genes, the rates of gene gain for *dispensable* genes were found to be significantly higher than the background rate of the genome for the most recent branches of the phylogenetic tree (**Fig. S123**), i.e., N1 (the *Mytilus* spp. lineage), N2 (the latest common ancestor of Mytilidae) and N3 (the latest common ancestor of all Bivalvia). On the contrary, the gene gain rate for *dispensable* genes was much lower than the background rate (i.e., the rate for all genes) for the older branches of the tree (N5-N9).

While testing for enrichment in the *dispensable* gene set, taxonomically restricted genes (**Data Note 20**) were found to be significantly over-represented (56% more than expected, FDR-corrected p-value < 0.0000001), bringing additional support to the recent origin of the PAV phenomenon in mussels.

**Fig. S122. *M. galloprovincialis* orthologs**. Line headers marked with the prefix “PAV” represent the subset of *dispensable* genes identified in *M. galloprovincialis* (**Data Note 8**). The second line header reported for each species represents the whole genome. Black, light blue, yellow and dark blue segments represent the percentage of “on- to-one” orthologs, “one-to-many” orthologs (one gene in *Mytilus* that presents more than one ortholog in the other species), “many-to-one orthologs” (several genes in *Mytilus* have only one match in the other species) and “many-to-many” orthologs.

**Fig. S123: Species tree obtained from the concatenation of 177 widespread single-gene families.** Species names in bold indicate genomes that have been sequenced in this study. Numbers in red represent the statistical support of the branch (aLRT), although only branches with a support value below 0.99 are represented. The topology agrees with the established phylogeny of mollusks, with Bivalvia as sister branch to Gastropoda; both forming a clade sister to Cephalopoda. Mollusca appears as sister branch of a clade containing Phoronida (Phoronis), Nemertea (Notospermus) and Brachiopoda (Lingula) with low support (0.503). Sisterhood of Brachiopoda + Phoronida is highly supported, though. Circles represent duplication rate values associated to each node as estimated by the phylome analyses. Yellow circle represents duplication values before removing large expansions consisting on more than 20 paralogs appearing in a single branch. Green circles represent duplication rates after removal of such events. Black circle serves as a scale and corresponds to a duplication rate of 1, equivalent of having every gene in the genome duplicated once on average.

**Fig. S124. Duplication rates per branch: PAV vs total**. Blue bar represents duplication rates calculated using all genes, while yellow bar represents duplication rates for genes subjected to PAV. N1 represent *Mytilus*. N2 represents Mytilidae. N3 represents Bivalvia. N4 represents Bivalvia+Gastropoda. N5 represents Mollusca. N6 represents Lophotrochozoa, excluding Annelida. N7 represents Lophotrochozoa. N8 represents Protostomia. N9 implies that the gene orthology can be traced all the way from *Mytilus* to, at least, Bilateria. See **Fig. S123** for details on the tree topology.

**Fig. S125**. ***M. galloprovincialis* genes gains against the oldest node with inferred orthology, represented as a percent of total.** Blue bars represent all genes while yellow bars represent only the subset of *dispensable* genes (subject to presence-absence variation). Branches refer to the topology in the phylogenetic tree (**Fig. S123**). N1 corresponds to the *Mytilus* lineage. N2 represents Mytilida. N3 represents Bivalvia. N4 represents Bivalvia+Gastropoda. N5 represents Mollusca. N6 represents Mollusca. N6 represents Lophotrochozoa, excluding Annelida. N7 represents Lophotrochozoa. N8 represents Protostomia. N9 implies that the gene orthology can be traced all the way from *Mytilus* to, at least, Bilateria.

## Association between PAV and expanded gene clusters

We also investigated whether any of the expanded gene clusters previously described as frequently occurring in the mussel lineage (see **Data Note 5**) was significantly associated with PAV. The gene set enrichment test revealed that 65 gene clusters were significantly over-represented in the *dispensable* gene set (see **Additional file 2:** **Table S57**), whereas only a single one was under-represented. This single gene cluster (cluster 1, including 309 proteins) comprised low complexity proteins with unknown function and high content in Glu, Leu and Lys (> 10% each) residues. On the other hand, enriched gene clusters encoded proteins that, despite lacking in most cases significant GO annotations and significant BLAST similarity with proteins with known functions deposited in public sequence databases, often possessed recognizable conserved domains. Some examples are reported in **Fig. S126**.

Not surprisingly, many of the over-represented gene clusters were characterized by the presence of domain annotations enriched in the *dispensable* gene set (**Table S52**). Most notably, many families comprised ankyrin domains (e.g. cluster 2, 241 and 436), scavenger receptor cysteine-rich domains (cluster 121), Mab-21 (cluster 3), C1q (cluster 39) and fibrinogen C-terminal domains (cluster 19). Interesting associations were also observed in some cases, e.g., cluster 300, where immunoglobulin-like, helicase and RIG-I domains were simultaneously present in proteins displaying significant similarity with Helicard, a protein regulating DNA degradation during apoptosis [170].

**Fig. S126. Examples of gene clusters expanded in *M. galloprovincialis* that resulted to be significantly enriched in the *dispensable* gene set.** ANK: ankyrin repeat; FBG: fibrinogen C-terminal domain; IG: immunoglobulin; DEXDc: DEAD-like helicase; HELICc: helicase C-terminal domain; SR: scavenger receptor cysteine-rich; RIG-I_CRD: Regulatory domain of RIG-I.

A number of gene clusters associated with PAV could not be associated to any conserved protein domains, likely due to the taxonomically restricted nature of many *dispensable* mussel sequence (see **Data Note 20**) and the lack of previously characterized functional domains.

We further investigated the phylogenetic relationships among the genes belonging to a few of the aforementioned clusters, highly enriched in *dispensable* genes, using Bayesian inference. As it can be observed in **Fig. S127** for cluster 19 and **Fig. S128** for cluster 39, almost no identical or nearly-identical paralogous gene copies were identified, ruling out the possibility that some of our observations linked with massive lineage-specific gene family expansions (**Data Note 5**) are connected with artefactual dupications of homologous haplotype stretches, which were explicitly removed during the early stages of the assembly process (**Data Note 1.2**). The two phylogenetic trees reported here as an example were generated based on the multiple sequence alignment of the protein sequences obtained from the longest splicing isoform, extracted from the mg10 assembly. The MSAs, obtained with MUSCLE [73] and converted in a NEXUS format, were used as an input for a Bayesian phylogenetic inference analysis with MrBayes 3.2.6 [127], run for 200,000 generations, which allowed convergence of the estimated parameters of the molecular model of evolution, as estimated by Tracer. The selected model of molecular evolution, in both cases, was LG+G+I, based on ModelTest-NG [91] estimates (<https://github.com/ddarriba/modeltest>). The resulting trees are displayed below with midpoint rooting. Poorly supported branches (posterior probability < 0.5) were collapsed.

**Fig. S127. Bayesian phylogeny of *M. galloprovincialis* genes associated with cluster 19** (See **Table S57**). *Dispensable* genes are highlighted with a yellow background. The analysis was carried out with MrBayes, using two parallel MCMC analyzes, run for 200,000 generations under a LG+I+G model of molecular evolution. The phylogenetic tree is reported here with midpoint rooting.

**Fig. S128. Bayesian phylogeny of *M. galloprovincialis* genes associated with cluster 39** (See **Table S57**). *Dispensable* genes are highlighted with a yellow background. The analysis was carried out with MrBayes, using two parallel MCMC analyzes, run for 200,000 generations under a LG+I+G model of molecular evolution. The phylogenetic tree is reported here with midpoint rooting.

# Data Note 20 – Taxonomically restricted genes and their relationship with PAV

## Bayesian reconstruction of Mytilida phylogeny

Based on the observation that many mussel *dispensable* genes are significantly younger than the *core* genes (see **Data Note 19**), we evaluated the impact of “taxonomically restricted genes” (TRGs, i.e., genes whose presence is limited to particular taxa [171]), in the mussel genome. In detail, we expanded the analyses presented in **Data Note 5**, by characterizing the protein-coding sequences from the *Lola* mg10 assembly lacking any significant BLAST hit against the other sequenced molluscan genomes (**Data Note 3**). *Lola* mg10 proteins were subjected to a tBLASTn analysis against the aforementioned genomes, using an e-value threshold of 1E-5 and an identity level threshold = 30%. Proteins lacking positive hits were selected and their annotations were extracted. This approach permitted to identify 5,240 TRGs, which can be considered as innovations of the *Mytilus* lineage.

To better pinpoint along the evolution of Mytilida when such innovations could have taken place, we carried out a Bayesian phylogenetic analysis based on the concatenated multiple sequence alignment of 978 universally conserved single copy orthologs identified with BUSCO [25], based on the set of metazoan orthologs from OrthoDB v.9 [63]. Besides *M. philipinarum*, *B. platifrons*, *L. fortunei* [48,67] and *M. galloprovincialis* (*Lola* mg10), species with gene models available from previously published genome studies, we included in this analysis other *de novo* assembled mytilid transcriptomes available in the NCBI SRA database (with the CLC Genomics Workbench 12 *de novo* assembly tool). Namely, we selected:

-*Bathymodiolus manusensis* (PRJNA360359)

-*Bahymodiolus puteoserpentis* [172]

-*Modiolus modiolus* (PRJNA353979)

-*Modiolus kurilensis* (PRJNA360359)

-*Mytilisepta virgata* [173]

-*Perumytilus purpuratus* (PRJNA343253)

-*Trichomya hirsuta* (PRJNA407964)

-*Perna viridis* [174]

-*Mytilus edulis* (PRJNA249058)

-*Mytilus chilensis* (PRJNA296917)

-*Mytilus trossulus* (PRJNA249058)

-*Mytilus californianus* (PRJNA249058)

-*Mytilus coruscus* (PRJNA269004)

Gene models from the scallop *Mizuhopecten yessoensis* [51] were used as outgroups to root the tree.

The amino acid sequences of each individual BUSCO for all the species taken into account were aligned, and the resulting alignments were trimmed and refined using GUIDANCE2 [175], further keeping into consideration only positions of the alignment where at least 50%+1 of the included species were represented. The resulting alignments were concatenated and subjected to Prottest [176] evaluation, to assess the best-fitting model of molecular evolution. This resulted to be WAG+G+I, i.e., a WAG model [177], with a gamma-distributed rate of variation across sites and a proportion of invariable sites. The dataset, comprising 318,332 aligned amino acids, was subsequently analyzed with MrBayes v.3.2.6 [127], applying the WAG+G+I model of molecular evolution and running the analysis for 100,000 generations, until the two independent analyses reached convergence (average standard deviation of split frequencies < 0.05) and all the estimated parameters of the model reached an ESS (effective sample size) > 100. Run convergence was evaluated with Tracer (<http://www.beast2.org/tracer-2/>).

The results of this analysis are presented in **Fig. S129**. In agreement with recent studies, the four species pertaining to the *Mytilus edulis* species complex (*M. edulis*, *M. galloprovincialis*, *M. trossulus* and *M. chilensis*) were grouped within the same clade, with *M. californianus* and *M. coruscus*, also pertaining to the same genus, forming a separate lineage [178]. The groups of Bathymodiolinae, Modiolinae, Arcuatulinae and Brachidontinae (i.e., *M. virgata* plus *P. purpuratus*, supporting the need for a revision of the classification of the former species. as suggested by Gerdol *et al.* [173]) were also clearly visible. The Asian green mussel *P. viridis* was placed at the basis of the mytilid tree, similar to another recent study [178]. The closest outgroup to the *Mytilus* clade was identified as *T. hirsuta*, a species currently classified as a member of *Septiferinae* (<http://www.marinespecies.org/aphia.php?p=taxdetails&id=506191>), but previously shown to be closely related to *Mytilus* spp. based on molecular evidence [179].

**Fig. S129**. **Majority rule consensus** **Bayesian phylogenetic tree of Mytilida based on genome and transcriptome data.** The tree is based on the concatenated multiple sequence alignment of 978 BUSCOs. Posterior probability support values are shown for each node. The scallop *M. yessoensis* was used as an outgroup. Species with an available genome are marked with a yellow background. The ancestral node of the “*Trichomya + Mytilu*s clade”, identified as the plausible earliest point of origin of the TRGs of *M. galloprovincialis*, is marked with a red star.

Although this tree cannot be considered as an exhaustive representation of the diversity of mytilids due to the lack of sequence data from several relevant taxa (e.g., from the subfamilies Crenellinae and Musculinae), it can provide a general overview of the relationships among key mytilid species. Consistent with the lack of significant sequence similarity between the 5,240 *M. galloprovincialis* TRGs and gene models from *L. fortunei*. *M. philippinarum* and *B. platifrons*, the origin of most of these sequences could be hypothetically placed somewhere between the split of the *Mytilus* + *Trichomya* clade from other mytilids (red star in **Fig. S129**) and the most recent split between *M. galloprovincialis* and other *Mytilus* spp., unless gene loss events are considered to explain these patterns.

## Examples of taxonomically-restricted gene families

An in-depth analysis of the 5,240 *M. galloprovincialis* TRGs revealed that they contained, among the others, several *Mytilus*-specific gene families encoding antimicrobial peptides and defense molecules, namely:

- 6 mytilins, cysteine rich AMPs highly expressed in hemocytes [180].

- 8 myticins, cysteine rich AMPs highly expressed in hemocytes, showing a 3D arrangement similar to defensins [138].

- 5 myticalins, recently described linear cationic AMPs expressed in gills [181].

- 5 CRP-I, cysteine-rich cationic pre-propeptides with unknown function but subject to extraordinary sequence diversity and positive selection [182].

## Correlation between TRGs and PAV

We also evaluated whether any significant association could be defined between TRGs and PAV, as this would have been somehow expected considering the recent origin of several *dispensable* genes (**Data Note 19**). We observed that only 8.72% of mussel *core* genes could be identified as TRGs at whole-genome scale, a percentage that raised to 37.50% for *dispensable* genes. These results, fully consistent with the indications gathered from the evolutionary analyses of the PAV phenomenon (**Data Note 19**) indicate that TRGs are 4.29 times more likely than evolutionary conserved genes to be subject to PAV.

# Data Note 21 – Key examples of PAV

We selected some examples of gene families subject to PAV to provide an in-depth overview about the most characterizing features of this phenomenon in the mussel genome. These analyses were based on the *de novo* assembly of Illumina PE libraries obtained from the resequenced mussel genomes (**Data Note 1**, **Additional file 2:** **Table S34**). Considering the lack of long (PacBio) reads, the genome assemblies obtained were quite fragmented (see **Table S41**), but still of sufficient quality to retrieve meaningful sequence information for short-range analyses (e.g., for analyses based on the retrieval of single exons). The genome assembly from *Pura*, previously published [17], was also analyzed in a similar way.

## Elongation factor 1 alpha

Elongation factor 1 alpha (EEF1A1) is a housekeeping gene that encodes the alpha subunit of the elongation factor-1 complex, frequently used as an internal control for normalization in many gene expression studies carried out on bivalves [183–185]. EEF1A1 is generally considered to be a universally conserved single-copy gene for Metazoa in OrthoDB v9 [63]. Surprisingly, the analysis of the mussel genome assembly (version mg3) enabled the detection of a second gene copy (hereafter named EEF1A1_bis, not to be confused with the molecular marker EFbis, used for the assessment of genetic introgression in **Data Note 7**), most likely corresponding to an inactive pseudogene. Indeed, in spite of a gene architecture nearly identical to that of EEF1A1 (**Fig. S130**), the second gene copy displayed a markedly divergent first coding exon, which lacked the expected ATG start codon encoding Met1. Moreover, no evidence in support of the expression of EEF1A1_bis could be found, neither in the transcriptome of *Lola*, nor in any of the other RNA-seq datasets available.

Although the two sequences share 84.5% sequence identity at the nucleotide level within the coding region (only hypothetically in EEF1A1_bis due to the lack of the initial ATG, which translates in > 86% sequence identity at the amino acid level. **Fig. S130B**), they are placed on different genomic scaffolds in *Lola* and display significant intron length polymorphisms (**Fig. S130A**). We used the sequence of exon 4, which appeared to be the most appropriate region for phylogenetic inference due to its higher sequence variability compared to the other 5 coding exons (**Fig. S130A**), as a molecular marker to track the evolution of the two genes in *M. galloprovincialis*.

**Fig. S130**. **EEF1A1 gene**. Panel A: gene architecture of the EEF1A1 gene and EEF1A1_bis pseudogene in *Lola*. Panel B: sequence alignment of EEF1A1 and EEF1A1_bis virtually translated proteins from *Lola*.

The use of a single exon was necessary in order to: (i) discriminate between the two allelic variants possibly present for each of the two genes in each resequenced genome, and (ii) enable the inclusion of all the variants identified, regardless to the quality of the assembled genomes (i.e., independently of the relevant level of fragmentation of some genome assemblies). In detail, the nucleotide sequences of exon 4 from *Lola* EEF1A1 and EEF1A1_bis were used as queries in BLASTn similarity searches [13] against *de novo* assembled genomes. Subsequently, clean Illumina reads from each genome were mapped to the sequences obtained, using the CLC genomics Workbench *map reads to contigs* tool (length fraction = 0.75, similarity fraction = 0.95) to obtain information concerning SNPs (whenever present), thereby enabling the reconstruction of the complete allelic variants.

While one or two allelic variants were detected, in all cases, for EEF1A1, EEF1A1_bis resulted to be a *dispensable* gene, as no significant BLAST hits could be detected in many genomes. The absence of the gene was confirmed, on a case-by-case basis, through the back-mapping of clean reads on the EEF1A1_bis reference sequence from *Lola*, which evidenced, in all cases, a complete lack of mappable reads. The situation observed in all the available mussel genomes is summarized in **Table S58** below. The *dispensable* nature of this pseudogene is in line with the frequent occurrence of non-coding *dispensable* genes observed in *Lola* (see **Data Note 9**).

**Table S58**: summary of presence-absence variation of the EEF1A1_bis pseudogene in *M. galloprovicnialis*.

BLAST searches were extended to a number of transcriptomic datasets from Mytilidae (described in **Data Note 20**). The oysters *C. gigas* and *C. virginica* were used as outgroup. The nucleotide sequences were aligned with MUSCLE [73], included in the MEGAX suite [123], treating the sequences as protein-coding and thereby preserving codon positions in the multiple sequence alignment (MSA). The MSA, converted in a NEXUS format, was used as an input for a Bayesian phylogenetic inference analysis with MrBayes 3.2.6 [127], run for 100,000 generations, which allowed convergence of the estimated parameters of the molecular model of evolution, as estimated by Tracer. The selected model of molecular evolution was WAG+G+I, based on ModelTest-NG [91] estimates (<https://github.com/ddarriba/modeltest>). The resulting tree, displayed in **Fig. S131**, was rooted on the branch leading to the two outgroup species. Poorly supported branches (posterior probability < 0.5) were collapsed. The consensus tree topology indicates:

1. High divergence between *M. galloprovincialis* EEF1A1 and EEF1A1_bis, much higher than the inter-species divergence between EEF1A1 from *Mytilus* and other Mytilida
2. The presence of different allelic variants, in both genes. These occur more frequently in EEF1A1 (a *core* gene) than in EEF1A1_bis (a *dispensable* gene, expected to be in most cases present in just one out of the two homologous chromosomes)
3. A topology consistent with the evolution of Mytilidae, as previously estimated by phylotrascriptomics (**Data Note 20**)

Overall, the branch including EEF1A1_bis alleles is deeply rooted in the Mytilidae lineage, stemming from a node closer to the root than the one marking the divergence between *P. viridis* and other Mytilida. This may indicate an ancient origin for this gene copy, which would consequently suggest that two EEF1A1 genes were likely to be present in the most recent common ancestor of all Mytilida and that, for unknown reasons, one of the two copies underwent progressive pseudogenization. Unfortunately, no other genome for other *Mytilus* species is available to test whether genes orthologous to EEF1A1 are present, and the pseudogene-like status of this gene determines the impossibility of detecting its mRNA in the available transcriptomes. Moreover, we could not detect EEF1A1 paralogous gene copies in the genomes of *L. fortunei*, *B. patifrons* and *M. philippinarum*. These observations leave many alternative evolutionary scenarios open: first, one may argue that the ancestral accessory EEF1A1 gene copy might have been lost in all Mytilida, except in *Mytilus* spp., explaining the apparent absence of this sequence in the genomes of the three species mentioned above.

The study of the EEF1A1 and EEFA1A1_bis gene pair, with the identification of the ancient origins of the latter, has further implications, on a larger scale, on all Mytilidae. Indeed, this finding suggests the possibility that the PAV phenomenon might be more widespread, possibly also involving other bivalve genomes characterized by high heterozygosity levels and very large repertoires of protein-coding genes (**Data Note 3**).

**Fig. S131**. **Bayesian phylogenetic tree of EEF1A1 in Mytilida based on exon 4 sequence** (see text for details). The two sequence clusters obtained for *M. galloprovincialis* indicate the EEF1A1 *core* and the EEF1A1_bis *dispensable* pseudogene, respectively.

## Mytilins

Mytilins represent one of the first families of antimicrobial peptides described in marine mussels, thanks to their isolation from active fractions of hemolymph [186]. In the subsequent years, mytilins emerged as primary effectors of innate immunity, produced as pre-propeptides, rich in cysteine residues (arranged in four disulfide bridges) and adopting a cysteine-stabilized alpha helix/beta sheet fold [138]. In *M. galloprovincialis*, following the identification of mytilin B, C, D and G1 either by proteomics methods or by the screening of EST databases [187], some additional unusual variants, mytilin K and N, and a small group of divergent sequences, pseudomytilins, have been recently described [140]. Preliminary screenings revealed than neither mytilin K nor mytilin N were present in the *Pura* genome (data not shown), and the availability of *Lola* and several resequenced individual genomes permitted to obtain in-depth information concerning the organization and presence-absence of such genes. **Fig. S116** depicts the organization of the genomic region that includes mytilin genes, which show a cluster organization. The *core* mytilin genes identified in *Lola* whose presence could be confirmed in all genomes are mytilin B, C, D, G1 and the two pseudogenes mytilin J and P. On the other hand, pseudomytilins appear to be encoded in a different genomic region, which may include multiple nearly identical paralogous genes, as shown by the difficulties observed in its *de novo* assembly in different genomes, as well as by the high number of independent contigs assembled in each individual genome (see below).

As previously demonstrated, mytilin K is most certainly a *dispensable* gene (**Data Note 12**), inserted within the mytilin gene cluster (**Fig. S116**), which does however maintain signatures of functionality (**Fig. S117**). We assessed whether any other mytilin genes shared the same features of presence-absence, considering the sequence of exon 3 (**Fig. S132**), encoding the mature peptide region displaying maximal diversity and thereby most informative from a phylogenetic point of view. This strategy was also used for other AMP families due to the fragmented nature of *de novo* assembled mussel genomes (**Table S41**).

**Fig. S132. Structure of the mytilin D gene**. All the members of the mytilin gene family comprise 4 exons, with the first only including 5’UTR. Exon 3, entirely embedding the mature peptide region (indicated with a box), was selected for phylogeny.

In brief, reference amino acid mytilin sequences from *M. galloprovincialis* (mytilin B, C, D, G1, K and N, plus pseudomytilin-1, -2 and -3) were retrieved from publicly available sequence databases at NCBI and used as tBLASTn queries against the *de novo* assembled mussel genomes. Regions corresponding to the mature peptide region (see **Fig. S132**) were extracted, translated, and aligned with MUSCLE [73]. Psuodogenes were also included in this case. STOP codons and positions with frameshift mutations were identified with a “*” symbol. MSAs were used as an input for a simple neighbor-joining (NJ) phylogenetic analysis, to briefly summarize the levels of similarity across the different variants. NJ trees were obtained with MEGA X [123], based on the calculation of p-distances, assuming a gamma distribution of rates among sites, with a proportion of invariable sites. The support of the tree was evaluated with 100 bootstrap replicates. Although, probably due to the short length of the alignment, most branches were poorly supported, the diversification patterns obtained are still useful to inspect the degree of sequence variation of mytilins (and this consideration extends to all the other AMP families that will be described in the following sections). The phylogenetic tree of the mytilin sequences is displayed in **Fig. S133**. In spite of the apparent simplicity of the canonical gene cluster identified in *Lola* (**Fig. S116**), many additional sequences could be identified besides the six *core* genes (mytilin B, C, D, G1 and the two pseudogenes J and P). This resulted in a complex branching pattern, denoting a somewhat unexpected degree of molecular diversity compared to that described for mytilins in previous works [188], but which has been recently evidenced by a study focused on the evolution of this AMP family [139].

First, we could detect a high number of contigs encoding pseudomytilins, a group of recently described sequences [140] that appear to be part of a relatively large multigenic cluster, possibly comprising up to a dozen different genes in each individual genome. Overall, we could detect 31 unique pseudomytilin sequences, some of which are obvious pseudogenes due to the presence of stop codons within the exon 3 ORF. Curiously, only one pseudomytilin sequence (PM1) was shared, with no variation, by all mussels, followed by a second sequence (PM8) shared by 11 mussels, and a third sequence (PM7) shared by 9. Since pseudomytilins are found in all mussel genomes, including *Lola*, we could observe that they are not part of the mytilin gene cluster, even though their sequence similarity and identical genomic organization (exon intron boundaries and intron phase) strongly supports their relatedness with canonical mytilins.

Mytilin B, mytilin C and mytilin G1 were invariably found, with a major variant, in all mussel genomes, even though a second slightly divergent version (variations of a single amino acid residue) was found for MytlC (in GALM1 and GALF3) and MytlG1 (in GALM2). On the other hand, the fourth *core* functional mytilin gene, mytilin D, was present with two variants (D1 and D2), found in 11 and 7 mussel genomes, respectively. As individual mussels can share the two variants, it appears likely that these two represent allelic variants of the same gene, as in the case of the MytlC and MytlG1 variants. Other cases of similar sequences characterized by a higher level of diversity compared to the four canonical sequences require further investigation. Indeed, the discrimination between a novel gene and an allelic variant is not straightforward (see **Data Note 24** for an example) and, in the absence of a detailed characterization of the entire genomic locus (impossible at this stage given the high fragmentation of resequenced genomes), it needs to be considered as preliminary and arbitrary. Most notably, two variants (named mytilin F1 and F2), found in two (GALF1 and GALM3) and one (ITAM1) mussel genomes, respectively, correspond to a sequence which has been previously defined as a highly divergent allelic variant of mytilin B, possibly resulting from introgression [189].

The other sequences included in the tree comprise the previously described sequences of mytilin K (found in a single genome, GALM1) and N (absent in all mussels) [140], as well as other novel sequences derived from the *de novo* assembly of various RNA-seq resources (**Table S11**). These include mytilin L and M [139]. Moreover, additional sequences were detected in the *de novo* assembled mussel genomes. These include mytilin K2 (a complete and possibly functional gene), mytilin O1 and O2 (two pseudogenes with multiple frameshift and nonsense mutations), all identified in the GALM1 genome (which also harbors mytilin K). Therefore, GALM1 includes a total of 6 functional mytilin genes (B, C, D, G1, K and K2) and 4 pseudogenes (J, P, O1 and O2). We could also detect an additional sequence variant, named mytilin H, in a single genome (GALM2). As this sequence is somewhat similar to mytilin D (**Fig. S133**), its assignment as the product of an independent *dispensable* gene is preliminary, like in the case of mytilin F.

The two *core* pseudogenes part of the canonical mytilin cluster, mytilin J and P, were found to bear a higher rate of inter-individual sequence variability, consistently with the expected relaxed evolutionary constraints which apply to non-coding regions. Despite the presence of 7 unique variants of MytlJ and 10 unique variants of MytlP, these all created well-supported monophyletic clusters in the phylogenetic tree (**Fig. S133**). As no more than two different variants were found in any mussel genome, it seems likely that the sequence variants found for these pseudogenes represent allelic variants of the same locus.

**Fig. S133**. **Neighbor-Joining tree of mytilins based on exon 3 sequence**. *Core* genes (found in all resequenced mussels) are circled in red. Note that pseudomytilins are present in multiple gene copies, whereas no more than two variants (likely allelic) were found for any of the other *core* mytilin genes, which are therefore expected to be present as single-copy genes. The name of each sequence is paired with the name(s) of the genome(s) where any gene sequence was identified. Mytilin B, C, D, G1, J and P are part of the “canonical” mytilin gene cluster (**Fig. S63**). See the text above for a detailed discussion. Bootstrap support values are shown for each node. “PG” indicates pseudogenes.

## Mytimacins

Mytimacins are another family of cysteine-rich AMPs, originally described with 5 variants in *M. galloprovincialis* [190]. Besides their function as antibiotic peptides, macins have been also connected with wound healing in annelids [191], but this role remains to be established in bivalves. Unlike mytilins, mytimacins are encoded by genes scattered in different genomic scaffolds, that are intronless and encode peptides with variable number of cysteine patterns and disulfide connectivity [192]. The strategy we used for the analysis of mytimacin sequences was similar to that described above for mytilins, with the exception of the use of the full-length ORF in the generation of MSA. Only 2 out of the 5 sequences originally described by Gerdol *et al.* by the screening of transcriptome data [190] were identified as *core* genes. Namely: (i) mytimacin-1, displaying a relatively low degree of inter-individual diversity, with the main variant found in 7 genomes, accompanied by 10 other variants showing minor modification in single residues (with the exception of MM-1g, found in GALM3, with a STOP codon disrupting the ORF); and (ii) mytimacin-3, a sequence displaying a much higher level of intraspecific diversity (**Fig. S134**). The most frequent MM-3 variant was found in 9 genomes, but other 10 variants (all potentially functional due to the lack of nonsense mutations) could be identified.

Mytimacin-2, characterized by the presence of a peculiar stretch of glycine residues at the N-terminus of the mature peptide region [190], was present in a single genome, ITAF3, whereas the most complex sequence, mytimacin-5, showing two additional cysteine residues and a C-terminal extension, was absent in *Lola* and in all resequenced genomes. Mytimacin-4, another sequence supported by gene expression data, could be only identified with three different variants, in just genomes, i.e., GALF2, ITAF2, ITAF3, ITAM2 and ITAM3. This data clearly implies that mytimacin-2, -4 and -5 are *dispensable* genes and that MM-2 and MM-5 might be found with very low frequency in mussel populations.

Surprisingly, a third *core* gene was identified in the genomes of *M. galloprovincialis*, even though this sequence had not been reported in previously sequenced transcriptomes [190]. MM-6 displayed 13 different variants, with the most frequent one represented in 7 genomes. It is definitely noteworthy that 5 out of 13 variants were found to contain premature STOP codons, suggesting that this gene copy might be currently undergoing pseudogenization, as the lack of reported expression (in the transcriptome of *Lola* and in all available RNA-sequencing datasets available for *M. galloprovincialis*) also suggests.

Besides the aforementioned sequences, two additional *dispensable* genes (MM-7 and MM-8) were detected in a single individual (GALF2), with MM-7 likely to be a pseudogene due to the substitution of one of the cysteine residues of the disulfide array with a STOP codon.

As an additional note, the endogenous origin of MM-2 needs to be validated, as (i) this variant was only identified in ITAF3, the individual subject to a significant contamination from a hydroid mantle parasite (see **section 14.5**), and (ii) this variant shared significant similarity with a mytimacin gene from the cnidarian species *C. hemisphaerica*.

**Fig. S134. Neighbor-Joining tree of mytimacins based on the complete translated CDS sequence**. *Core* genes (found in all resequenced mussels) are circled in red. The name of each sequence is paired with the name(s) of the genome(s) where any gene sequence was identified. See the text above for a detailed discussion. Bootstrap support values are shown for each branch. “PG” indicates pseudogenes.

## Myticins

Myticins are a third example of cysteine-rich AMPs specifically expressed in circulating cells [180], which share a similar gene architecture and, possibly, also a structure similar to that of defensins and mytilins [193]. However, myticins possess some peculiar features that clearly differentiate them from the other mussel AMP families, such as an extreme, and still not completely understood, level of intraspecific sequence diversity [194,195], and remarkable properties as antiviral agents and chemotactic molecules [196]. However, the genetic basis of this astonishing sequence diversity is still a matter of debate and, while it has been established that myticin C is encoded by at least two paralogous gene copies [197], as previously mentioned the assembly of the genomic scaffold containing myticin genes has been a particularly challenging task (**Data Note 1**). Due to the complexity of the locus and the presence of repeated elements, the precise number of myticin genes in *Lola* remains uncertain, but the most significant data which emerges with certainty is that the myticin gene cluster possess more genes than the three originally hypothesized main sequences, i.e., mytilin A. B and C. The in-depth analysis of WGR data, and in particular of exon 3 that, like in mytilins, encodes the entire mature peptide region (**Fig. S135**), enabled to obtain a detailed overview of the molecular diversification of myticins in *M. galloprovincialis*.

**Fig. S135.** **Structure of the myticin A gene.** All the members of the myticin gene family comprise 4 exons, with the first only including 5’UTR. Exon 3, entirely embedding the mature peptide region (indicated with a box), was selected for phylogenetic reconstruction.

Overall, we identified 63 myticin variants, plus 10 pseudogenes containing nonsense mutations within exon 3, and nine additional variants of pseudomyticins (see below). The reference sequences of myticin A and B [180] were only found in five mussel genomes (MytA: GALF2, GALF3, GALM3, ITAF1 and ITAM3; MytB: *Lola*, *Pura*, GALM1, GALM6 and GALM11). The reference sequences of myticin A and B are shown with arrowheads in **Fig. S136**. Several similar variants were identified in the other genomes. While considering the reference sequence of myticin C reported by Venier *et al.* [198], this variant was only found in five genomes (GALM1, GALF3, ITAF1, ITAF3 and ITAM1). Several variants, consistent with previously reported molecular diversity of myticin C transcripts [194,195], are present in the other resequenced genomes. These are no exceptions, as we could not find any variant shared by all genomes. The variant with the highest frequency was found in 11 genomes (one of the sequences present in the clade of myticin B), followed by a sequence found in nine genomes (pertaining to the large myticin A clade). Several of the variants identified were only found in one or two genomes.

**Fig. S136. Neighbor-Joining tree of myticins based on the exon 3 sequence.** The name of each sequence (in this case the sequences are numbered, with the exception of the reference sequences of MytA, B and C, indicated with arrowheads), is paired with the name(s) of the genome(s) where any gene sequence was identified. Pseudomyticins, discussed in detail in the text and portrayed with an example in **Fig. S137,** are highlighted with a red circle. The Myticin G clade, also discussed in detail in the text, is highlighted with a blue circle. Bootstrap support values are shown for each node. “PG” indicates pseudogenes.

Besides the 10 pseudogenes found (containing in-frame nonsense mutations), another clade of sequences, named pseudomyticins, was identified. These contained either one or two variants per individual (circled in red in **Fig. S136**). The most frequently found variant was found in 9 genomes. Pseudomyticins lacked two cysteine residues (Cys1 and Cys5) which are allegedly involved in the formation of an intermolecular disulfide bond [193]. These sequences also retained a well conserved signal peptide, a net positive charge (theoretical pI > 9) and a few key conserved residues, in particular a 8 amino acid-long stretch in the region surrounding Cys2 and Cys3, which was found to be nearly identical to myticin A, B and C. The deletion of Cys5 however determines relevant structural changes and other deletions in the central region, comprised between Cys3 and Cys6, even though Cys4 appears to be conserved. In addition, also the C-terminal extension region appeared to be considerably shorter than canonical myticins, despite the maintenance of a negative charge. Overall, pseudomyticin precursor proteins are 69 aa long, but their mature peptide is expected to be just ~30 aa (**Fig. S137**). The analysis of the neighboring genomic regions confirmed the presence of pseudomyticins in the myticin cluster, highlighting their relatedness with canonical myticins. Although evidence supporting their poor expression could be collected by the analysis of RNA-seq data, we could also identify point non-sense mutations in the second exon (encoding the signal peptide region), suggesting that this group of sequences are undergoing a process of progressive loss of function.

**Fig. S137. Multiple sequence alignment of full-length precursors of myticin A, B, C and pseudomyticin(-2).**

Overall, the myticin tree (**Fig. S136**) displays a level of molecular diversity far superior than the previously reported presence of just three major myticin variants (A, B and C) in *M. galloprovincialis*, even though several sequences similar to these three references were observed. Compared to the cases of mytilins and mytimacins discussed above, the identification of clades likely to identify a single gene with its allelic variants is much more difficult, due to the extreme amount of diversification observed (**Fig. S136**). For example, while a myticin B cluster is somewhat apparent, both myticin A and myticin C clearly pertain to a large clade of highly diverse sequences, which also include pseudogenes. Interestingly, several novel clades of myticins were found that clearly represent cases of PAV (e.g., the clade comprising sequences 45 and 53 was only found in four genomes), and possible cases of *core* genes with a substantial level of allelic variation (e.g. the large clade circled in blue in **Fig. S136**). The 14 sequences comprised in this well-supported clade (bootstrap support value = 93), hereafter named the myticin G clade (**Fig. S138**), were found in all genomes. Nevertheless, quite surprisingly, we did not find any report in literature on any of these sequence variants to date, possibly due to their restricted level of expression compared to the myticin A, B and C variants. This interpretation is also supported by the presence of nonsense mutations in at least 5 myticin G-like sequences. While an intact exon 3 sequence was observed in the other 9 variants, we cannot exclude that deleterious mutations are present in the other two exons hat contain portions of the ORF (exons 2 and 4).

**Fig. S138**. **Multiple sequence alignment of the translated sequences of exon 3 of myticin genes pertaining to the G clade**. See **Fig. S136** and text.

Indeed, as shown in **Fig. S139**, with the exception of pseudomyticins a particular concentration of pseudogenes is apparent within the myticin G clade and, less frquently, in the larger myticin A clade. However, it needs to be remarked that the analysis of exon 3, by itself, is not sufficient to pinpoint a pseudogene, as deleterious mutations might be also present in the other exons or in regulatory regions in the promoter, which might impair the functionality of a given gene.

Please note that the myticin sequence data has been further analyzed and elaborated for the preparation of a recently published study [199].

**Fig. S139**. **Distribution of confirmed pseudogenes (i.e., sequences with in-frame stop codons in exon 3) in the myticin phylogenetic tree.** Note the presence of multiple pseudogenes in the myticin A and G sequence clades. The position of pseudomyticins (also likely to be pseudogenes due to the loss of two cysteine residues) is also shown.

## Mytimycins

Mytimycins are part of the cysteine-rich AMPs originally isolated in the ‘90s from the active fraction of the mussel hemolymph [186]. In contrast with myticins and mytilins, mytimycins display a strictly antifungal activity [200] and, in spite of the presence of a cysteine array, they do not appear to be related with the other cysteine-rich AMP families, adopting an undisclosed three-dimensional fold and showing a different gene architecture (**Fig. S140**). Early transcriptomic investigations have revealed a certain level of sequence diversity, permitting to identify several variants characterized by a variable number of cysteine residues, similar to the case of mytimacins [198]. Regardless of these structural variations, all mytimycin genes appear to comprise three exons, with the coding region split between the second (encoding the signal peptide and the mature region) and the third one (encoding the C-terminal extension, which includes an EF-hand domain). Although apparent differences in the expression of mytimycins across individuals have been interpreted as the result of a different ability to react to a given stimulus [201], we can here provide evidence in support of PAV as the main factor underlying the anomalies pointed out by PCR approaches.

**Fig. S140. Structure of the mytimycin K gene**. All the members of the mytimycin gene family comprise 3 exons, with the first only including 5’UTR. Exon 2, entirely embedding the signal peptide and mature peptide regions, was selected for phylogeny.

Using the same approach outlined above for other AMP families, we used the sequence of exon 2 for phylogenetic reconstruction. Overall, the mytimycin sequences clustered in 13 different clades (A-M) that, despite being arbitrarily set, are well recognizable in the NJ tree (**Fig. S141**) and clustered with high support (bootstrap > 90). The cysteine arrays characterizing each sequence group are summarized in **Fig. S142**.

- Cluster A was found in a total 6 genomes, with 2 variants. Its typical disulfide array comprised 12 cysteine residues, organized as follows: CC-C-C--C-C--C-C-C-C-C-C. Mytimycin-A can be therefore considered as a *dispensable* gene.

- Cluster B was found in all genomes, even though in many cases its sequence included variations and nonsense mutations, displaying a broader cluster compared to other mytimycins (**Fig. S141**) and supporting the loss of function of this gene in some individuals (see **Fig. S143**). In detail, 8 potentially valid and 7 pseudogenic variants could be identified. Its typical disulfide array comprised 12 cysteine residues, organized as follows: CC-C-C-CC-C-CC-C-C-C--C-C, but many variants lacked one of such residues, further suggesting a possible progressive loss of function for this gene. In any case, mytimycin B can be considered as a *core* gene, albeit with limited signatures of functionality.

- Cluster C was found in all genomes, with the single exception of GALF2. A total of 12 variants were identified, all potentially functional. Its typical disulfide array comprised 12 cysteine residues, organized as follows: CC-C-C-CC-C-CC-C-C-C. Mytimycin-C can be therefore considered as a *dispensable* gene.

- Cluster D was found in the majority of genomes, with the exception of *Lola* and GALM2. A total of 7 variants were identified, all potentially functional. Its typical disulfide array comprised 12 cysteine residues, organized as follows: CC-C-C-CC-C-CC-C-C-C-C-C. Mytimycin-D can be therefore considered as a *dispensable* gene.

- Cluster E was found in 7 genomes, with 2 variants, both potentially functional. Its typical disulfide array comprised 12 cysteine residues, organized as follows: CC-C-C-CC-C-CC-C-C-C-C-C. Mytimycin-E can be therefore considered as a *dispensable* gene.

- Cluster F was found in 11 genomes, with 3 variants, all potentially functional. Its disulfide array comprised 12 cysteine residues, organized as follows: CC-C-C-CC-C-CC-C-C-C-C-C. Mytimycin-F can be therefore considered as a *dispensable* gene.

- Cluster G was found in 7 genomes, with 2 potentially functional and 3 pseudogenic variants (**Fig. S144**), containing missense mutations. Consequently, a potentially functional gene was only present in four mussel genomes. Its tyical disulfide array comprised 12 cysteine residues, organized as follows: CC-C-C-CC-C-CC-C-C-C-C-C. It is however noteworthy that one of the two variants lacking in-frame STOP codons, lacks two of the cysteine residues expected to be engaged in intramolecular disulfide bonds. Mytimycin-G can be therefore considered as a *dispensable* gene with limited signatures of functionality.

- Cluster H was found in 10 genomes, with 2 potentially functional and 3 pseudogenic variants (**Fig. S144**), containing missense mutations. Consequently, a potentially functional gene was only present in ten mussel genomes. Its typical disulfide array comprised 12 cysteine residues, organized as follows: CC-C-C-CC-C-CC-C-C-C. Mytimycin-H can be therefore considered as a *dispensable* gene with limited signatures of functionality.

- Cluster I was found in 8 genomes, with a single functional variant found in a single genome (ITAM3) and 5 pseudogenic variants (**Fig. S144**), containing missense mutations. Its typical disulfide array comprised 12 cysteine residues, organized as follows: CC-C-C--C-C--C-C-C-C-C-C. Mytimycin-I can be therefore considered as a *dispensable* gene with very limited signatures of functionality, potentially retaining a biological activity only in a single genome out of those subject to WGR.

- Cluster J was found in 7 genomes, with a single potentially functional variant (found in all the 7 genomes where the gene could be detected) and a pseudogenic variant with missense mutations (only found in GALF1). Its typical disulfide array comprised 12 cysteine residues, organized as follows: CC-C-C--C-C--C-C-C-C-C-C. Mytimycin-J can be therefore considered as a *dispensable* gene.

- Cluster K was found in 9 genomes, with 2 variants, both potentially functional. Its typical disulfide array comprised 12 cysteine residues, organized as follows: CC-C-C--C-C--C-C-C-C-C-C. Mytimycin-K can be therefore considered as a *dispensable* gene.

- Cluster L was found in 6 genomes, with 4 variants, all potentially functional. Its typical disulfide array comprised 12 cysteine residues, organized as follows: CC-C-C-CC-C-CC-C-C-C-C-C. Mytimycin-L can be therefore considered as a *dispensable* gene.

- Cluster M was only found in 2 genomes. The variant identified in GALM1 was potentially functional, whereas that found in GALF3 contained multiple missense mutations. The typical disulfide array of the variant without STOP codons comprised 12 cysteine residues, organized as follows: CC-C-C-CC-C-CC-C-C-C-C-C. Mytimycin-M can be therefore considered as a *dispensable* gene, with very limited signatures of functionality.

Overall, it is interesting to note that among the 13 types of mytimycins found in the resequenced mussel genomes, only one (cluster B) can be considered as a *core* gene. However, even in this case the sequence displays important intraspecific variations (**Fig. S141**), which in several cases lead to the loss of function of the sequence, due to the inclusion of in-frame STOP codons (**Fig. S143**). Therefore, mytimycins appear as a case where PAV is extremely widespread, including most, if not all, the genes included in the family. Curiously, the mytimycin sequence which has been so far the target of most studies^175.176^ only appears to be a *dispensable* gene present in a fraction of genomes (nine out of 16), so many of the considerations reported in previous studies about the apparent different responsiveness of the gene in different individuals are most likely related with PAV.

**Fig. S141. Neighbor-Joining tree of mytimycins based on exon 2 sequence** (**Fig. S140**). The name of each sequence (based on their arbitrary classification within 13 clusters) is paired with the name(s) of the genome(s) where any gene sequence was identified. The sequences are subdivided in 13 clusters, discussed in detail in the text above.

**Fig. S142**. **Summary of the cysteine clusters characterizing each mytimycin sequence cluster.** “-“ indicates the presence of a variable number of non-cysteine residues. Cysteine residues which are not found in all sequence variants are marked with a yellow background.

The study of the mytimycins reveals that different mussels may not only differ in terms of the type of mytimycins present, but also in their number, ranging from just 3 (in *Lola*) to 10 (in *Pura*, GALM1 and ITAM1), averaging 7. Even more interestingly, mytimycins can be grouped in 3 major types of disulfide arrays (**Fig. S142**). i.e., type I (clusters C and H), type II (clusters A, I, J and K) and type III (clusters B, D, E, F, G, L and M). While all mussels possessed at least one sequence pertaining to class II and class III, a single individual, i.e., GALF2, lacked type I sequences (**Fig. S144**).

**Fig. S143**. **Distribution of confirmed pseudogenes (i.e., sequences with in-frame stop codons in exon 3) in the mytimycin phylogenetic tree.** Note the presence of multiple pseudogenes in the mytimycin B. G. H and I clades.

**Fig. S144. Distribution of mytimycin clusters across mussel genomes**.

## Big defensins

Big defensins (BDs) are unusual antimicrobial peptides that combine two potentially active regions, the N-terminal alpha helical region, and the C-terminal cysteine-rich region, which structurally resembles vertebrate β-defensins [203,204]. The typical disulfide array involved six cysteines, arranged in a C-C-C-C-CC motif. Big defensins have been identified in a number of bivalve species [205–207], including *M. galloprovincialis*. where 8 different variants have been previously described [190]. BDs do not show specificity of expression to hemocytes, unlike other invertebrates, rather displaying a broad expression pattern in all tissues, which may suggest a role in mucosal immune response [204]. Studies carried out in oyster, following the observation of unusual gene expression profiles, have previously pointed out high intraspecific sequence divergence, which might result in PAV [204,208]. Here we provide evidence that mussel big defensins are indeed subject to widespread PAV. In this case, the exon encoding the cysteine-rich region (exon 3) was used for phylogenetic reconstruction (**Fig. S145**).

**Fig. S145**. **Structure of the BD1 gene**. All the members of the big defensing gene family comprise 3 exons, with the first only including 5’UTR. Exon 3, entirely embedding the cysteine-rich region, was selected for phylogeny.

Mussel BDs displayed a remarkable inter-individual sequence diversity (**Fig. S146**). Notably, none of the 8 variants previously described was found in all genomes, and a total of 25 unique sequences (17 novel), plus 12 pseudogenic variants (i.e., carrying nonsense or frameshift mutations, or lacking any of the conserved cysteine residues) were identified in the 16 mussel genomes. Namely:

- BD1 was found in 14 genomes

- BD2 was found in 13 genomes

- BD3a was not detected in any of the sequenced genomes, but this sequence is supported by RNA-seq evidence from other individuals [190]

- BD3b was found in 8 genomes

- BD3c was found in 3 genomes

- BD4 was found in a single genome (*Pura*)

- BD5 was found in 11 genomes

- BD6 was not detected in any of the sequenced genomes, but this sequence is supported by RNA-seq evidence from other individuals [190]

Many of the remaining novel variants identified were only found in single individuals, with some notable exceptions (i.e., BD11, found in 14 genomes. BD13 in 6, BD16 in 5 and BD15 in 4). Like in the case of myticins, the discrimination between allelic variants and products of different genes is not straightforward, but the close similarity of different variants (**Fig. S146**) suggests that a number of the variants identified may represent allelic variants of the same gene. Nevertheless, big defensins most definitely pertain to a multigenic family, including up to 13 variants within the same individual (GALF2), subject to massive PAV.

**Fig. S146**. **Neighbor-Joining tree of big defensins, based on exon 3 sequence** (**Fig. S145**). The name of each sequence (based on their arbitrary classification within 13 clusters) is paired with the name(s) of the genome(s) where any gene sequence was identified. The sequences previously reported in Gerdol *et al*. 2015 are marked with a red star. “PG” indicates pseudogenic variants.

The mussel big defensin gene data has been further analyzed in a recent work [204], which took into account the uncertainties concerning the assignment of the different isoforms to genes. With a maximum parsimony approach, the authors grouped all the big defensin variants of *M. galloprovincialis* in six clusters, i.e., *Mg*-BigDef1, *Mg*-BigDef2/6, *Mg*-BigDef3, *Mg*-BigDef4, *Mg*-BigDef5 and *Mg*-BigDef7. PAV analysis substantially confirmed the indications provided by the data reported above (**Fig. S147**), with *Mg*-BigDef2/6 being the only cluster found in all individuals with potentially functional variants, whereas *Mg*-BigDef1, which was also present in all individuals, was represented by pseudogenes in two individuals. The four other clusters were subject to PAV and sometimes also displayed pseudogenic variants only.

**Fig. S147**. **Neighbor-Joining tree of big defensins, based on exon 3 sequence** (**Fig. S145**). The name of each sequence indicates the six major groups identified, with a maximum parsimony approach, in a recent study [204].

## Myticalins

Myticalins are the latest additions to the growing group of AMPs of *Mytilus* spp. These antibacterial molecules, produced as prepropeptides, are mainly expressed in gills and display a broad spectrum of activity against Gram+ and Gram- bacteria [181]. Myticalins are characterized by a hypervariable central mature peptide region, whose amino acid composition is used for classification purpose, defining four subgroups of sequences, i.e., myticalins A, B, C and D. Here we extend the data published in a recent paper [181], augmenting the family of myticalins to 74 members, including 15 pseudogenes (**Fig. S148**). Due to the extreme levels of divergence of the mature peptides of myticalins and the impossibility to align members of the four different subfamilies, the molecular diversity of these AMPs will be reported here only in terms of presence-absence across the 16 available genomes.

The global overview on the presence-absence matrix of myticalin genes (**Fig. S149**) reveals that:

- each mussel contains a unique set of myticalins genes, consisting of a combination of myticalin A, B, C and D sequences

- each mussel shows a different number of unique variants, ranging from 4 (*Pura* and *Lola*) to 13 (GALM1)

- each mussel possesses at least 1 myticalin A and one myticalin C sequence

- the single member of the myticalin B subfamily is subject to PAV, being found in only three out of 16 mussel

- the mussel individuals analyzed in this study contain a variable number of myticalin D sequences, from 0 (*Lola*, *Pura*, GALM2, ITAM2 and ITAM3) to 5 (GALM1). However, all mussels studied here possessed at least one (and possibly multiple) myticalin D pseudogenes.

**Fig. S148**. **Sequences of the mature peptides of the 74 myticalin variants identified in mussel genomes and transcriptomes.** “PG” denotes pseudogenic variants.

**Fig. S149. Presence-absence matrix of myticalins in *M. galloprovincialis*.** Pseudogenes and variants found in other *Mytilus* spp. species or only detected in transcriptomes have been omitted.

# Data Note 22 – Size and composition of the mussel pan-genome

## Estimates of the size of the mussel pan-genome

1. *Lola* possess more than 300 “private” genes which are not found in any of the 14 resequenced genomes (plus *Pura*) (**Table S37**).
2. that a slightly lower number of “private” gene (about 100) was also found, on average, in the recursively reassembled pan-genomic contigs (**Fig. S100**) and, in total, 1,541 off the 5,286 newly annotated genes were “private” (29.15%).
3. a number of additional *dispensable* genes, absent in *Lola*, can be found in multiple transcriptomic datasets (**Data Note 13 and 16**).

In light of the existence of the PAV phenomenon, and based on the three observations listed above, one might argue that the global size of the mussel pan-genome may be considerably larger than the 60,338 genes annotated in the reference genome. Specifically, each of the 15 additional genomes might be expected to possess a number of private genes similar to *Lola*, providing a contribution of approximately 4,500 additional *dispensable* genes. This computational estimate was actually very close to the annotation of 5,286 genes present in the recursively reassembled pan-genomic contigs (**Data Note 15**).

The random sampling model of the mussel pan-genome (**Fig. S150**), should be extended to match the actual (very large) effective population size of the mussel genome, possibly bringing the size of the pan-genome to about 70,000 genes. However, this estimate does not consider a number of factors which may play an important role in shaping up the mussel genome. First, the number of individuals considered is too low to provide an accurate estimate, and only mussels from two distinct populations were considered (i.e., Galicia and Northern Adriatic Sea). Second, the impact of genetic introgression on PAV is presently unknown. Similarly, the estimate of the *core* genome size presented in **Data Note 5** may be considered as a slight over-estimate of its actual size, and only a larger sampling size may provide a precise definition of the mussel *core* genome (**Fig. S150**).

**Fig. S150. Expected increase of the pan-genome size, and decrease of the *core* genome size.** Based on a random sampling model, with 100 independent replicates.

An estimate of the total size of the pan-genome, in terms of assembled nucleotides, is provided in **Data Note 15**, **Figures S69** and **S70**. Overall, 578 Mb of additional genome sequence data, not included in the reference individual (*Lola*) could be *de novo* recursively reassembled from 15 individuals. As mentioned above for the estimate of the number of protein-coding genes, this estimate suffers from limitations, i.e., the technical limitations in obtaining high quality *de novo* assemblies from short reads only, and the fact that only a relatively low number of individuals from two independent mussel populations were sampled.

## Comparison with other species

As mentioned in the main text, the study of PAV has been so far mostly focused on prokaryotes. Just a few examples have been documented in eukaryotes, and large-scale studies have been limited to a few species of plants and microalgae, and no data whatsoever has been ever produced in animals. The data collected so far in bacteria indicate that the size of pan-genomes, as well as the proportion between *core* genes and *dispensable* genes, largely vary across species and critically depend on the capability of colonization of new environments of a given species [209]. With this respect, pan-genomes with a large proportion of *dispensable* genes are defined as “open pan-genomes”, whereas those where *core* genes represent the overwhelming majority are defined as “close pan-genomes”. Even though a precise estimate of the size of a pan-genome depends on the number of individual genomes sequenced, the data currently available and previously presented by McInerney *et al.* [209] can be used for a preliminary comparative assessment (**Fig. S151**). This includes the genomes of *M. galloprovincialis* and *Homo sapiens* [210] among metazoans, a few representative bacterial species, and some non-metazoan eukaryotes where such data is presently available, i.e., *Oryza sativa* [211], *Glycine soja* [212] and *Emiliania huxleyii* [213]. Note that the data reported below do not include the >5,000 additional *dispensable* genes annotated in the contigs resulting from the recursive pan-genome reassembly (see **Data Note 14** and **15**)

**Fig. S151. Comparative overview of pan-genome structure.** Data extracted from McInerney *et al*., 2018 [209]*.*

Overall, the *M. galloprovincialis* pan-genome shows a fraction of *dispensable* genes significantly lower than most bacterial species, which are more prone to large insertions of genetic material through plasmids. However, when compared to plants and microalgae, mussel displays an open pan-genome, including 24.25% *dispensable* genes, a value higher than the two land plants analyzed and just slightly lower than the coccolithophore *E. huxleyii*. Moreover, it needs to be considered that, as mentioned before, this figure represents an underestimation of the actual fraction of *dispensable* genes in the mussel pan-genome, as only the genes annotated in the *Lola* reference genome were taken into account here.

Recently, a few studies have expanded the pan-genome concept to higher metazoans, with reports in humans [210,214,215] as well as in pigs [216]. With the exception of the study by Sudmant and colleagues, these studies did not explore in detail the gene content of pan-genomic contigs, but they provided interesting estimates of the size of “novel sequence”, not included in the reference genome assembly. In detail the study by Sherman and colleagues, which analyzed a much higher number of individuals (i.e., 910) compared with our study, reported 296 Mb genomic sequence not included in the reference human genome [215]. Li and colleagues worked on a much smaller sample size, joining the human reference genome assembly with two assemblies of genomes with African and Asian descent, identifying ∼5 Mb of novel sequence data in each of them, which anabled the authors to estimate that the complete human pan-genome may include between 19 and 40 Mb of sequence not included in the reference assembly [214]. In pigs, the size of the pig pan-genome, estimated based on a number of individuals similar to those we studied in mussels (12) revealed the presence of 72.5 Mb additional genomic sequence, accounting for about 3% of the pig haploid genome size [216].

We compared these estimates with our estimates, based on the recursive *de novo* reassembly strategy discussed in detail in **Data Note 14**. This comparison, graphically represented in **Fig. S152**, reveals the large difference in the extent of the *dispensable* part of the mussel pan-genome compared with the estimated relative size of the human and pig pan-genomes, scaled at the haploid genome size (**Fig. S152A**). In detail, the mussel pan-genomic contigs account for 45.14% of the reference assembly size, whereas this value stands at 9.55% in humans (based on the data provided by Sherman et al.) or 1.29% (based on the data provided by Li et al.). In pigs, the relative size of the dispensable portion of the pan-genome is equal to 2.90%.

Hence, based on these data, the *dispensable* portion of the mussel pan-genome exceeds by nearly 5 times the upper estimate for humans, and by 15 times the estimate for pigs. Moreover, we need to remark that such estimate (i) did not take into account the hemizygous fraction of the mussel reference assembly (expected to account for additional 600 Mb of *dispensable* genomic sequence), and (ii) was based on a much lower number of individuals (15), compared with the study by Sherman et al., which included 910 individuals (i.e., 60 times more).

A very recent study [217] ha deeply expanded our knowledge of structural variation in humans, in particular in relation with the association of large indels with protein-coding genes. Abel and colleagues, through the analysis of 17,795 genomes, found that ultra-rare structural variants affected on average 4.2 genes per individual. This estimate provides the opportunity for comparison with the mussel reference genome, which reveals a highly significant difference between humans and mussels (**Fig. S152C**).

As far as *dispensable* genes are concerned, an opportunity for a comparison between *H. sapiens* and *M. galloprovincialis* is offered by a recent study, which took into account 2,504 individual human genomes to build a detailed structural variation map [210]. This genomic revealed that a total of 240 genes were occasionally subject to homozygous deletions in healthy individuals. These genes, that can therefore be considered as likely to be *dispensable*, account for 1.17% of the protein-coding genes annotated in the most recent Ensembl human genome release, indicating that the fraction of dispensable protein-coding genes in *M. galloprovincialis* is about 20-folds higher than in *H. sapiens* (**Fig. S151**).

Overall, the relative contribution of *dispensable* genomic regions to the mussel pan-genome appears to largely exceed all the estimates presently available for human and pig.

**Fig. S152. Comparative overview of the reference genome assembly and pan-genome accessory contigs reassembly in Metazoa.** Panel A shows a comparative overview of the pan-genome size, scaled on the size of the haploid genome assembly, whereas panel B shows the raw assembly size data. Panel C shows a comparison between the proportion of the average number of protein-coding genes affected by PAV in mussel and SVs in human, based on the data reported by Abel and colleagues [217].

## Correlation with the geographical origin

The observed patterns of PAV seem to correlate with the geographical origin of the mussel specimens, as suggested by the Bayesian tree constructed based on the binary matrix of presence-absence of *dispensable* genes (**Fig. S153**), and previously hinted by a similar tree built using *dispensable* genes identified in *M. galloprovincialis* transcriptomes (see **Fig. S65**). In detail, the six Italian genomes are grouped in a clade supported by 1.0 posterior probability, the tree shows a star-like topology, with very long branches, suggesting some population structure for these traits.

**Fig. S153**. **Bayesian tree of the 14 resequenced mussel genomes (plus *Pura*) based on a binary presence-absence matrix calculated for the *dispensable* genes annotated in the reference genome**. The tree was built with MrBayes [127], with two independent MCMC analyses run in parallel for 100,000 generations. Numbers above branches are posterior probabilities.

## Assessment of the possible origin of *dispensable* genes from congeneric mussel species

The PAV pattern suggests that other factors, maybe linked to the complex evolutionary history of mussel populations, like relatively recent gene flow from geographically distant populations due to human activities, and possibly also genetic introgression from other congeneric species (see **Data Note 7**), might have been somehow involved in the generation of the current status of extreme intraspecific genomic variation of *M. galloprovincialis*. Although no genome data is presently available for other *Mytilus* species (except *M. coruscus*), we preliminarily assessed the likelihood that (i) *dispensable* genes have a *M. edulis*/*M. trossulus* origin, and (ii) that, alternatively, *M. galloprovincialis* *dispensable* genes (or highly similar sequences) are found in other species of the *Mytilus* genus. This analysis was performed by screening *de novo* assembled mussel transcriptomes (see **Data Note 13**) for the presence of a subset of 481 broadly expressed *M. galloprovincialis* *dispensable* genes (TPM > 10 based on the data reported in **Data Note 4**). The *M. coruscus* genome data was not used for this analysis, as its higher level of completeness compared with the transcriptomes of other mussel species might have introduced a source of bias.

The detection was based on BLASTn [118], and positive matches were only selected for a query coverage > 75%, e-value < 1E-30 and sequence identity > 85%. These thresholds were set to avoid the incorrect detection of paralogous gene copies, while enabling, at the same time, the detection of sequences that would were detected as “present” based on the mapping procedures applied in **Data Note 8**. This procedure should avoid the detection of false positives at the expense of the possible inclusion of some genes, characterized by high allelic divergence, as false negatives. Positive matches were further subdivided into two categories, i.e., “high confidence matches”, with sequence identity > 95%, and “low confidence matches, with sequence identity comprised between 90% and 95%. This was done keeping in mind the results of the simulations shown in **Fig. S55**, that indicate that a sequence divergence equal to 10% should not result in a significant decrease of mapping rates. In summary, if present, these matches were expected to obtain BLAST matches more significant than those any closely related gene identified as “absent” by our PAV dectection pipeline. Obviously, as the detection of *dispensable* genes in transcriptomes is strictly dependent on the completeness of transcriptomes themselves, the number of individuals used for its generation, and sequencing depth, the results presented below cannot be considered as quantitative, and have merely with a qualitative value.

The main results, portrayed in **Fig. S154**, strongly suggest that a certain number of *dispensable* genes which are absent in some of the resequenced genomes from *M. galloprovincialis* are present in the congeneric species *M. edulis*, in particular. In detail, 94 high confidence (many showing 100% sequence identity with *M. galloprovincialis*) and 17 low confidence hits, respectively, could be detected in this species. These numbers were just slightly lower than those observed in a *de novo* assembled *M. galloprovincialis* transcriptome of similar size. *M. trossulus*, in spite of a less complete transcriptome, also displayed traces of presence of *dispensable* genes (18 with high and other 18 with low confidence). These outcomes can be interpreted in three possible ways, none of which can be conclusively disproven at this time, in the absence of genomic data from any of these two species:

1. a number of *dispensable* genes in *M. galloprovincialis* have an origin from other species of the *M. edulis* species complex, which are partially inter-fertile, consistently with the presence of detectable genetic introgression (**Data Note 7**)
2. a number of *dispensable* genes of *M. galloprovincialis* origin has been transferred to other species of the *M. edulis* species complex
3. A number of *dispensable* genes in *M. galloprovincialis* have a relatively ancient origin from an ancestor shared with the other species of the *M. edulis* species complex.

In any case, these results strongly hint that the PAV phenomenon could be found in other Mytilida, most certainly in *M. edulis* and *M. trossolus*, whose genomes are likely to share similar structural features, as also suggested by the apparent ancient origin of some *dispensable* genes (see the example of EEF1A1_bis in **Fig. S131**). For this reason, we also included the non-interfertile congeneric species *M. californianus* and *M. coruscus* in the analysis, as well as *Trichomya hirsuta*, the most closely related species available not part of the *Mytilus* genus (**Fig. S129**). Although the proportion between low and high confidence hits in *M. californianus* and *M. coruscus* was reversed compared to the aforementioned cases of *M. edulis* and *M. trossulus*, a significant number of matches (30 and 21, respectively) were detected, suggesting that some *dispensable* genes may have a relatively ancient origin, maybe from past genetic introgression from the ancestors of the present species. *T. hirsuta*, included here as an outgroup, only resulted in the identification of two potential hits with low confidence, which are most likely explained as cases of convergent evolution, or otherwise as falsely detected *dispensable* genes in *M. galloprovincialis*.

In summary, while it is presently impossible to ascertain with certainty whether the origin of *dispensable* genes lays in past and present events of crossings between congeneric interfertile species, this preliminary analysis certainly points out that the PAV phenomenon is highly likely to be present in all the species of the *M. edulis* complex, possibly extending further beyond in mytilid taxonomy.

**Fig. S154**. **Detection of *M. galloprovincialis dispensable* genes (and similar sequences) in the transcriptomes of congeneric species**. High confidence hits indicate transcripts sharing a sequence identity > 95% with *M. galloprovincialis dispensable* genes. Low confidence hits indicate transcripts sharing sequence identity comprised between 90 and 95%. For comparison’s purpose, the results obtained from a *de novo* assembled transcriptome from *M. galloprovincialis* of similar size to that of *M. edulis* is also shown.

# Data Note 23 – Discussion of anomalous read mapping in male mussel gonads

## Observation of aberrant mapping profiles in male libraries obtained from the mantle tissue

As anticipated in **Data Note 8**, unexpected mapping profiles were obtained from the analysis of WGR data of some samples, specifically from sequencing data derived from genomic DNA extracted from the mantle tissue of male mussels. In detail, the two main peaks corresponding to *core* genes (normalized average coverage =2, indicating the presence of two alleles in the diploid genome) and *dispensable* genes (normalized average coverage = 1, indicating the presence of a single allele in the diploid genome), respectively, were not recognizable in GALM1 (**Fig. S36**) and ITAM1 (**Fig. S39**). Somewhat aberrant profiles, although at a lesser extent, were also observed in ITAM2 and ITAM3 (**Fig. S40**), whereas only minor alterations were observed for the two samples obtained from male mantle tissues, i.e., GALM2 and GALM3 (**Fig. S37**). On the other hand, no visible deviation compared to females was detectable in the GALM6 and GALM11 samples (**Fig. S37**).

The mantle tissue of mussels, like other bivalves, harbors gonads that largely expand during the reproductive season. In the non-reproductive season, the mantle tissue is mostly composed by connective tissue, which supports follicles, where germinal cells differentiate and maturate. The proportion between connective (i.e., diploid cells) and germinal tissues (including a large fraction of gametes, i.e., haploid cells) largely varies depending on the reproductive status [218] and period of the season. However, at the apex of the reproductive status, before spawning, the vast majority of cells found in this tissue are haploid. This is particularly true in males (**Fig. S155B**), where the ratio between spermatozoids (n) and spermatogonia (2n) is much higher than the ratio between oocytes (n) and oogonia (2n) in females (**Fig. S155A**).

**Fig. S155**. **Micrographs of female (A) and male (B) gonads, at the apex of reproductive season, before spawning.** Examples of oocytes, oogonia, spermatozoids and spermatogonia are indicated.

Genomic DNA extractions were carried out on male mussel individuals and, regardless of the reproductive season and of the diploid:haploid cells ratio in each mussel, all genomic regions would have been expected to be equally represented in the Illumina PE libraries generated as explained in **Data Note 1**. In other words, no genomic region should have been over- or under-represented compared to expectations, leading to a mapping profile comparable to those obtained in *Lola* and all female mussels (**Figure 2A** and **Data Note 8**).

As shown in **Fig. S156A**, the first library obtained from GALM1 (replicate A), resulted in a single, very “relaxed” peak of coverage, peaking at 0.6 (i.e., less than the expected hemizygous peak of coverage), whereas no homozygous peak of coverage could be detected. Similarly, the *k-mer*-based analyses did not enable an estimate of heterozygosity rates, due to the uneven distribution of *k-mer* frequencies. However, a significant fraction of genes still displayed a coverage level consistence with PAV (i.e., > 0.25). To exclude the possibility that this unexpected result might have been related to issues with the preparation of the library, a second replicate library (replicate B) was independently sequenced, leading to virtually identical results (**Fig. S156A**). The gene-wide mapping rates obtained from the two replicates displaying a R^2^ correlation coefficient = 0.87 (**Fig. S156C**), with just a single gene (MGAL10A023001) significantly deviating from the bisector of the graph, for unknown reasons. However, the preparation of sequencing libraries from genomic DNA extracted from the (non-germinal) gills tissue of the same individual permitted to obtain a mapping profile in line with the observations collected from all female mussels (**Figure 2A** and **Fig. S156A**), with two peaks clearly identifiable at normalized coverage equal to 1 (hemizygous peak) and 2 (homozygous peak), respectively.

An identical strategy was applied to ITAM1, the Italian male mussel displaying the most deviant mapping profile compared to expectations. Like GALM1, also ITAM1 showed nearly identical profiles from the two replicate libraries obtained from mantle genomic DNA (**Figures S156B** and **S156D**), with an R^2^ correlation = 0.84, definitely ruling out the possibility that issues related with library preparation may be at the basis of the observations. Curiously, the gene MGAL10A023001, as in the case of GALM1, for unexplained reasons was the only significant outlier. The observation of “normal” two-peaked expression profiles in the Illumina PE library obtained from the gills of the same individual (**Fig. S156D**) further confirmed that this anomaly was likely linked to the tissue of origin.

**Fig. S156.** **Mapping rates from the mantle tissue of GALM1 and ITAM1.** Panels A and C: comparison of the mapping rates obtained for mussel gene models in GALM1 and ITAM1, respectively. Two libraries were independently generated and sequenced at different times, starting from genomic DNA extracted from the mantle tissue (replicates A and B). The third library was obtained from genomic DNA extracted from gills of the same individuals. Panels B and D: linear regression analysis of the normalized mapping coverage rates obtained from the two replicate Illumina libraries generated from genomic DNA extracted from the mantle tissue of GALM1 and ITAM1, respectively.

In an attempt to pinpoint the exact relationship between the reproductive stage and the aberrant mapping profiles obtained from the reproductive tissue of male mussels, we collected the mantle tissue from two additional mussel individuals from Galicia (GALM6 and GALM11), characterized by a histologically-determined ripe and post-spawned gonadal status, respectively (**Table S1**, **Fig. S157**). Unfortunately, neither of the two samples displayed aberrant mapping profiles, suggesting that the reproductive status, by itself, cannot be considered as the only factor at the basis of the observations reported above.

**Fig. S157**. **Micrographs displaying the mantle tissue, with gonads, of ripe (GALM6, panel A) and spent/post-spawning (GALM11, panel B) male mussels.** Note the massive presence of haploid cells in the former, opposed to the absence of gametes in the latter.

While the massive presence of aneuploidy gametes in the gonadal tissue of ripe individuals remains the most likely explanation for this unexpected phenomenon, it remains to be established: (i) why this apparently only occurs in males, but not in females; (ii) whether the reproductive status and, consequently, the haploid:diploid cell ratio has an a significant effect; (iii) whether other factors (e.g. the genetic diversity of parental genomes) are somehow involved in the generation of a large number of aneuploid gametes in male mussel gonads.

## Peak coverage calibration in male mussels

Due to the aforementioned anomalies, while the identification of *dispensable* genes based on the visual inspection of mapping graphs and detection of hemizygous and homozygous peaks of coverage was straightforward, the same procedure in some male mussels (i.e., GALM2, GALM3, ITAM2 and ITAM3) was not equally simple. We applied a calibration procedure based on the identification of the *core* genes which displayed a very high stability in terms of coverage across all female genomes, plus male genomes with gills libraries available (ITAM1, GALM1, GALM6 and GALM11). First, the average coverage per gene were normalized, for each genome, based on the expected haploid genome size. Only genes displaying a normalized coverage comprised between 1.95 and 2.05 and a standard deviation lower than 0.2 were selected. This gene set, comprising 4,277 non-redundant sequences, was used as a benchmark to evaluate the skewness of mapping rates in the Illumina libraries obtained from male mantle tissues.

The distribution of normalized gene mapping rates for female genomes, ordered from the most to the least stable gene, are displayed in **Figures S158-S162**. Please note that, as *core* genes, these stable genes are expected to show a normalized coverage close to two, consistent with the presence of two alleles in the diploid genome. As expected, all female genomes displayed a highly uniform distribution of coverage, perfectly centered on 2, with no exception, independently from sample origin (Italy or Spain).

**Fig. S158**. **Normalized coverage of 4,277 high confidence *core* genes in (A) *Pura*, (B) GALF1, (C) GALF2 and (D) GALF3.** Genes are ordered from the most to the least “stable” (i.e., from the one showing the lowest to the one showing the highest standard deviation in female mussel samples + male mussels samples obtained from gills, see the text above for detail).

**Fig. S159**. **Normalized coverage of 4,277 high confidence *core* genes in (A) ITAF1, (B) ITAF2 and (C) ITAF3.** Genes are ordered from the most to the least “stable” (i.e., from the one showing the lowest to the one showing the highest standard deviation in female mussel samples + male mussels samples obtained from gills, see the text above for detail).

In addition to female samples, also male samples obtained from genomic DNA extracted from gills (in GALM1 and ITAM1) showed a highly uniform coverage distribution, centered on 2 (**Fig. S160A/B**), virtually indistinguishable from that obtained from genomic DNA extracted from the mantle of females (**Figures S158-S159**). Similarly, the mapping of the Illumina PE libraries obtained from the mantle tissue of GALM6 and GALM11 were highly uniform (**Figures S160C/D**).

**Fig. S160**. **Normalized coverage of 4,277 high confidence *core* genes in (A) GALM1, (B) ITAM1, (C) GALM6 and (D) GALM11.** The libraries represented here for GALM1 and ITAM1 were obtained from genomic DNA extracted from the gills tissue. Genes are ordered from the most to the least “stable” (i.e., from the one showing the lowest to the one showing the highest standard deviation in female mussel samples + male mussels samples obtained from gills, see the text above for detail).

Slight deviations from the homozygous peak of coverage were observed in GALM3 (**Fig. S161B**), which became more evident in GALM2 (**Fig. S161A**). In this case, with a few exceptions, the observed normalized coverage of most *core* genes was comprised between 1.5 and 2.5. This situation mirrors the observed skewed distribution previously described in **Data Note 8** and exemplified in **Figure 3A**. Deviations even more evident were notable in ITAM2 (**Fig. S161C**) and ITAM3 (**Fig. S161C**), once again in full agreement with the skewed distributions previously described for these two genomes in **Data Note 8** and exemplified in **Figure 2A**.

**Fig. S161**. **Normalized coverage of 4,277 high confidence *core* genes in (A) GALM2, (B) GALM3, (C) ITAM2 and (D) ITAM3**. Genes are ordered from the most to the least “stable” (i.e., from the one showing the lowest to the one showing the highest standard deviation in female mussel samples + male mussels samples obtained from gills, see the text above for detail).

Finally, consistent with the data previously reported for GALM1 and ITAM1 in **Fig. S162** and discussed above, these two genomes showed an extremely non-homogeneous mapping of reads to *core* genes, which roughly ranged between 0.5 (half of the expected coverage of a *dispensable* gene) and 4X the coverage expected for a gene present with four alleles in the diploid genome). The mapping profiles obtained were very similar, both between technical replicates and between the two individuals (**Fig. S162**). As previously mentioned, the mapping of reads obtained from a non-germinal tissue (gills) resulted in a mapping profile very similar to that obtained from the mantle of female individuals (**Fig. S160**).

**Fig. S162**. **Normalized coverage of 4,277 high confidence *core* genes the two technical replicates obtained from genomic DNA extracted from the mantle tissue, from GALM1 (A and B) and ITAM1 (C and D).** Genes are ordered from the most to the least “stable” (i.e., from the one showing the lowest to the one showing the highest standard deviation in female mussel samples + male mussels samples obtained from gills, see the text above for detail).

# Data Note 24 – Recommendations for gene expression studies

## How can *dispensable* genes be efficiently managed in gene expression studies?

One of the most important implications of the finding of widespread PAV in the mussel genome is that extra care should be taken while interpreting gene expression data originated from RNA-seq, microarray and even from qPCR studies. This may potentially have some repercussions also on studies that include antibodies, which may or may be not appear functional depending on the genetic background of each individual mussel and, consequently, on the protein variant produced. Several gene expression studies have indeed pointed out an outstanding variability of expression across biological replicates, even when mussels had been sampled in the same location and subjected to controlled laboratory tests [68,198]. These results, so far unexplained, now find a justification in the high number of *dispensable* genes expected to be found in each individual mussel, which might give rise to a large number of false positives and false negatives in genome-wide gene expression studies.

Based on these observations, we recommend that all future studies should consider differential gene expression (DGE) only if evidence in support of the status of any given gene as part of the mussel *core* gene set has been provided. On the other hand, the possibility of PAV should be carefully considered whenever a given gene is marked as part of the mussel *dispensable* gene set. Adjustments will be most certainly needed in the interpretation of differential gene expression in *M. galloprovincialis* and, until the real taxonomical extent of PAV will be elucidated, also in all *Mytilus* species. These considerations might be provisionally extended to other bivalves which, as explained in **Data Note 5 and 6**, also present a high rate of gene family lineage-specific expansions, large number of genes and high heterozygosity.

So far, we provisionally advise researchers interested in carrying out genome-wide gene expression studies in *M. galloprovincialis* by RNA-sequencing to:

1. Use the *Lola* assembly as a reference, limiting the inference of DEGs on *core* genes.
2. Use stringent FDR- or Bonferroni-corrected p-value and fold change thresholds for DEG detection, to limit the chances of false positive detection.
3. Use several independent biological replicates, as this might implement the ability to detect outliers, most likely linked to PAV.
4. Validate DEGs by qPCR, thereby ruling out the possibility of read cross-mapping among *core* and *dispensable* genes sharing local regions of high similarity.

Obviously, these expedients have some limitations, i.e., they rely on the accurate annotation of the reference genome. Indeed, despite its good level of completeness, a limited number of *core* genes might be absent from the genome assembly, either due to gaps or due to their short ORF length or divergence from known gene models in other species (**Data Note 2**). Most definitely, dedicated studies will be needed to determine the most appropriate pipeline of analysis for gene expression studies in *M. galloprovincialis*.

To help researchers with this task, we provide here a reliable list of highly stable reference housekeeping genes (part of the *core* gene set), which might be used to validate DEGs across a broad range of samples and tissues in addition to those most frequently used in literature (e.g. EF1α, actin, tubulin, 18S and 28S ribosomal RNA, etc). In detail, these genes were selected based on the mapping of RNA-seq data from multiple tissues and experimental conditions (see **Data Note 4**). So far, many studies have been carried out to establish stable housekeeping genes to be used as a reference for qPCR experiments. but in all cases these investigations have been carried out on a very limited number of genes (<20) [219–221]. Now we provide a broader view on this issue, also considering the PAV phenomenon. The list (**Table S59**) contains the 12 most stable genes identified in *M. galloprovincialis*, detected as follows. Briefly, the average TPM gene expression level of gene, previously calculated in multiple samples as described in **Data Note 4**, was computed. All gene expression values were divided by the average to obtain values centered on “1”. Finally, the standard deviation of each gene was calculated and the genes were ordered from the most stable (lower SD value) to the least stable (higher SD value). The average expression value of the 12 selected genes is also provided in order to further help researchers in the choice of the most appropriate housekeeping gene (or genes) in relation with the expected expression level of the target mRNAs.

Overall (**Table S59**), ribosomal proteins emerge as the best candidate housekeeping genes for qPCR. In general, they display strong expression levels (> 2,000 TPM), which implies their presence in the top 100 most expressed genes in each tissue. Based on stability, we suggest the use of Cytochrome c oxidase subunit 5B, mitochondrial and 26S proteasome complex subunit SEM1 as housekeeping genes whenever the target genes display a low expected expression level (i.e., whenever a high delta Ct may be observed between the reference and the housekeeping gene). These stable housekeeping genes show expression levels in the range of 150-300 TPM, and they can be therefore considered as part of the top 300/top 800 most expressed mRNAs, depending on the tissue of interest and experimental condition.

**Table S59. List of the 12 most stable housekeeping genes identified in the mussel genome, evaluated by RNA-sequencing across a broad range of tissues and experimental conditions**. *genes are ordered based on stability, i.e., from the lowest to the highest standard deviation across all samples, calculated after normalizing all expression values to the average expression level across all samples.

| **gene ID** | **annotation** | **average expression level (TPM)** | **stability*** |
| --- | --- | --- | --- |
| MGAL10A075356 | 60S ribosomal protein L32 | 3061.45 | 0.24 |
| MGAL10A009412 | 60S acidic ribosomal protein P2 | 4311.81 | 0.25 |
| MGAL10A086281 | 60S ribosomal protein L14 | 2747.49 | 0.25 |
| MGAL10A086168 | 60S ribosomal protein L34 | 2288.28 | 0.27 |
| MGAL10A081062 | Cytochrome c oxidase subunit 5B. mitochondrial | 303.35 | 0.28 |
| MGAL10A044516 | 40S ribosomal protein S19 | 2884.44 | 0.28 |
| MGAL10A041348 | 60S ribosomal protein L11 | 3353.90 | 0.28 |
| MGAL10A061238 | 60S ribosomal protein L18a | 1659.67 | 0.28 |
| MGAL10A017475 | 26S proteasome complex subunit SEM1 | 168.36 | 0.29 |
| MGAL10A087486 | 60S ribosomal protein L7a | 4164.32 | 0.29 |
| MGAL10A049522 | 40S ribosomal protein S21 | 2305.60 | 0.29 |
| MGAL10A090487 | 40S ribosomal protein S5a | 3078.93 | 0.30 |

## On the possible collapse of the product of paralogous gene copies

Another issue related to the high genomic variability of *M. galloprovincialis* is linked to the presence of multiple expressed sequences which, despite sharing high sequence similarity within the coding region, are the product of different genes. In such cases, the discrimination between allelic variants and paralogous gene products is not straightforward. In particular, the level of sequence identity between two paralogous gene products may often be so high that algorithms dedicated at the *de novo* assembly of RNA-seq data may fail in producing two distinct mRNA sequences, or produce partial transcripts due to fragmentation. This factor may possibly contribute to the generation of “*transcriptomic messiness*”, an issue previously described in other marine mollusk species [222].

We show here as an example the case of four closely related sequences pertaining to the large CRP-I gene family [182], encoding hypervariable cysteine-rich peptides with uncharacterized function. The four sequences depicted in **Fig. S163A**, CRP-I 9, 13, -58 and -60, all derive from the reference genome, and are therefore simultaneously present and, in spite of their remarkable sequence identity (up to 98.07% for the CRP-13/-60 pair at the nucleotide level within the ORF, **Fig. S163B**), there are clearly the product of four independent genomic loci. Theoretically, these four sequences may be the product of at least two independent genes (bearing two allelic variants each). However, the analysis of the neighboring genomic regions clearly points out these four sequences as the product of four different genes, as they are located on four different genomic scaffolds. Obviously, the high sequence similarity between these four sequences at the nucleotide level might represent a significant obstacle in the correct *de novo* assembly of four transcripts in a transcriptome study in the absence of a reference genome, due to possible fragmentation and the generation of chimeric contigs.

**Fig. S163**. **CRP-I gene family.** Panel A: multiple sequence alignment of the precursor peptides of CRP-I 9, -13, -58 and -60. Panel B: sequence similarity matrix, based on the multiple sequence alignment of the nucleotide sequences corresponding to the Open Reading Frames.

When the analysis was carried out at the genome level however, the divergence among the four genes emerged clearly. As shown in **Fig. S164**, the four sequences display considerable intron length polymorphisms and very poor sequence conservation in all intronic regions, with significant conservation only in the four regions corresponding to the four exons and, partly, in the putative promoter region (2 Kb of sequence upstream to the transcription start sites are also shown). While CRP-I 9, 13, -58 and -60 are just an example, we argue that hundreds of similar cases might exist in the mussel genome, representing potential sources of uncertainty for *de novo* transcriptome studies and PCR-based approaches alike.

**Fig. S164**. **Sequence alignment of the full-length CRP-I 9, 13, -58 and -60 genes, all present in *Lola*, with intron size indicated.** The 2Kb of genomic sequence upstream of the transcriptome start site are also included. Sequence conservation, calculated in a sliding window of 33 nucleotides of length, is also displayed.

# References

1. Martin M. Cutadapt removes adapter sequences from high-throughput sequencing reads. EMBnet.journal. 2011;17:10–2.

2. Magoč T, Salzberg SL. FLASH: fast length adjustment of short reads to improve genome assemblies. Bioinforma Oxf Engl. 2011;27:2957–63.

3. Marco-Sola S, Sammeth M, Guigó R, Ribeca P. The GEM mapper: fast, accurate and versatile alignment by filtration. Nat Methods. 2012;9:1185–8.

4. Marçais G, Kingsford C. A fast, lock-free approach for efficient parallel counting of occurrences of k-mers. Bioinformatics. 2011;27:764–70.

5. Liu B, Shi Y, Yuan J, Hu X, Zhang H, Li N, et al. Estimation of genomic characteristics by analyzing k-mer frequency in de novo genome projects. ArXiv13082012 Q-Bio. 2013

6. Cruz F, Julca I, Gómez-Garrido J, Loska D, Marcet-Houben M, Cano E, et al. Genome sequence of the olive tree, Olea europaea. GigaScience. 2016;5:29.

7. Simpson JT, Wong K, Jackman SD, Schein JE, Jones SJM, Birol İ. ABySS: A parallel assembler for short read sequence data. Genome Res. 2009;19:1117–23.

8. Ye C, Hill CM, Wu S, Ruan J, Ma Z (Sam). DBG2OLC: Efficient Assembly of Large Genomes Using Long Erroneous Reads of the Third Generation Sequencing Technologies. Sci Rep. 2016;6:31900.

9. Parra G, Bradnam K, Korf I. CEGMA: a pipeline to accurately annotate core genes in eukaryotic genomes. Bioinforma Oxf Engl. 2007;23:1061–7.

10. Boetzer M, Henkel CV, Jansen HJ, Butler D, Pirovano W. Scaffolding pre-assembled contigs using SSPACE. Bioinforma Oxf Engl. 2011;27:578–9.

11. Boetzer M, Pirovano W. SSPACE-LongRead: scaffolding bacterial draft genomes using long read sequence information. BMC Bioinformatics. 2014;15:211.

12. Wood DE, Salzberg SL. Kraken: ultrafast metagenomic sequence classification using exact alignments. Genome Biol. 2014;15:R46.

13. Altschul SF, Madden TL, Schäffer AA, Zhang J, Zhang Z, Miller W, et al. Gapped BLAST and PSI-BLAST: a new generation of protein database search programs. Nucleic Acids Res. 1997;25:3389–3402.

14. Camacho C, Coulouris G, Avagyan V, Ma N, Papadopoulos J, Bealer K, et al. BLAST+: architecture and applications. BMC Bioinformatics. 2009;10:421.

15. English AC, Richards S, Han Y, Wang M, Vee V, Qu J, et al. Mind the Gap: Upgrading Genomes with Pacific Biosciences RS Long-Read Sequencing Technology. PLoS ONE. 2012;7:e47768.

16. Hackl T, Hedrich R, Schultz J, Förster F. proovread: large-scale high-accuracy PacBio correction through iterative short read consensus. Bioinforma Oxf Engl. 2014;30:3004–11.

17. Murgarella M, Puiu D, Novoa B, Figueras A, Posada D, Canchaya C. A First Insight into the Genome of the Filter-Feeder Mussel Mytilus galloprovincialis. PLOS ONE. 2016;11:e0151561.

18. Moreira R, Pereiro P, Canchaya C, Posada D, Figueras A, Novoa B. RNA-Seq in Mytilus galloprovincialis: comparative transcriptomics and expression profiles among different tissues. BMC Genomics. 2015;16:728.

19. McKenna A, Hanna M, Banks E, Sivachenko A, Cibulskis K, Kernytsky A, et al. The Genome Analysis Toolkit: A MapReduce framework for analyzing next-generation DNA sequencing data. Genome Res. 2010;20:1297–303.

20. Dobin A, Davis CA, Schlesinger F, Drenkow J, Zaleski C, Jha S, et al. STAR: ultrafast universal RNA-seq aligner. Bioinforma Oxf Engl. 2013;29:15–21.

21. Zhang SV, Zhuo L, Hahn MW. AGOUTI: improving genome assembly and annotation using transcriptome data. GigaScience. 2016;5:31.

22. Kajitani R, Toshimoto K, Noguchi H, Toyoda A, Ogura Y, Okuno M, et al. Efficient de novo assembly of highly heterozygous genomes from whole-genome shotgun short reads. Genome Res. 2014;24:1384–95.

23. Laetsch DR, Blaxter ML. BlobTools: Interrogation of genome assemblies. F1000Research. 2017;6:1287.

24. Challis R, Richards E, Rajan J, Cochrane G, Blaxter M. BlobToolKit – Interactive Quality Assessment of Genome Assemblies. G3 GenesGenomesGenetics. 2020;10:1361–74.

25. Simão FA, Waterhouse RM, Ioannidis P, Kriventseva EV, Zdobnov EM. BUSCO: assessing genome assembly and annotation completeness with single-copy orthologs. Bioinforma Oxf Engl. 2015;31:3210–2.

26. Mapleson D, Garcia Accinelli G, Kettleborough G, Wright J, Clavijo BJ. KAT: a K-mer analysis toolkit to quality control NGS datasets and genome assemblies. Bioinformatics. 2017;33:574–6.

27. Nguyen TTT, Hayes BJ, Ingram BA. Genetic parameters and response to selection in blue mussel (Mytilus galloprovincialis) using a SNP-based pedigree. Aquaculture. 2014;420–421:295–301.

28. Haas BJ, Salzberg SL, Zhu W, Pertea M, Allen JE, Orvis J, et al. Automated eukaryotic gene structure annotation using EVidenceModeler and the Program to Assemble Spliced Alignments. Genome Biol. 2008;9:R7.

29. Iwata H, Gotoh O. Benchmarking spliced alignment programs including Spaln2, an extended version of Spaln that incorporates additional species-specific features. Nucleic Acids Res. 2012;40:e161.

30. Parra G, Blanco E, Guigó R. GeneID in Drosophila. Genome Res. 2000;10:511–5.

31. Lomsadze A, Ter-Hovhannisyan V, Chernoff YO, Borodovsky M. Gene identification in novel eukaryotic genomes by self-training algorithm. Nucleic Acids Res. Oxford Academic; 2005;33:6494–506.

32. Stanke M, Waack S. Gene prediction with a hidden Markov model and a new intron submodel. Bioinforma Oxf Engl. 2003;19 Suppl 2:ii215-225.

33. Quinlan AR, Hall IM. BEDTools: a flexible suite of utilities for comparing genomic features. Bioinformatics. 2010;26:841–2.

34. Hunter S, Apweiler R, Attwood TK, Bairoch A, Bateman A, Binns D, et al. InterPro: the integrative protein signature database. Nucleic Acids Res. 2009;37:D211–5.

35. Kanehisa M, Sato Y, Kawashima M, Furumichi M, Tanabe M. KEGG as a reference resource for gene and protein annotation. Nucleic Acids Res. 2016;44:D457–62.

36. Conesa A, Götz S, García-Gómez JM, Terol J, Talón M, Robles M. Blast2GO: a universal tool for annotation, visualization and analysis in functional genomics research. Bioinformatics. 2005;21:3674–3676.

37. Petersen TN, Brunak S, von Heijne G, Nielsen H. SignalP 4.0: discriminating signal peptides from transmembrane regions. Nat Methods. 2011;8:785–6.

38. Marchler-Bauer A, Lu S, Anderson JB, Chitsaz F, Derbyshire MK, DeWeese-Scott C, et al. CDD: a Conserved Domain Database for the functional annotation of proteins. Nucleic Acids Res. 2011;39:D225-229.

39. Jones P, Binns D, Chang H-Y, Fraser M, Li W, McAnulla C, et al. InterProScan 5: genome-scale protein function classification. Bioinforma Oxf Engl. 2014;30:1236–40.

40. Moriya Y, Itoh M, Okuda S, Yoshizawa AC, Kanehisa M. KAAS: an automatic genome annotation and pathway reconstruction server. Nucleic Acids Res. 2007;35:W182–5.

41. Simakov O, Marletaz F, Cho S-J, Edsinger-Gonzales E, Havlak P, Hellsten U, et al. Insights into bilaterian evolution from three spiralian genomes. Nature. 2013;493:526–31.

42. Zhang G, Fang X, Guo X, Li L, Luo R, Xu F, et al. The oyster genome reveals stress adaptation and complexity of shell formation. Nature. 2012;490:49–54.

43. Nawrocki EP, Eddy SR. Infernal 1.1: 100-fold faster RNA homology searches. Bioinforma Oxf Engl. 2013;29:2933–5.

44. Nawrocki EP, Burge SW, Bateman A, Daub J, Eberhardt RY, Eddy SR, et al. Rfam 12.0: updates to the RNA families database. Nucleic Acids Res. 2015;43:D130-137.

45. Lowe TM, Eddy SR. tRNAscan-SE: a program for improved detection of transfer RNA genes in genomic sequence. Nucleic Acids Res. 1997;25:955–64.

46. Li R, Zhang W, Lu J, Zhang Z, Mu C, Song W, et al. The Whole-Genome Sequencing and Hybrid Assembly of Mytilus coruscus. Front Genet. Frontiers; 2020;11.

47. Uliano-Silva M, Dondero F, Dan Otto T, Costa I, Lima NCB, Americo JA, et al. A hybrid-hierarchical genome assembly strategy to sequence the invasive golden mussel Limnoperna fortunei. GigaScience. 2017;7:gix128.

48. Sun J, Zhang Y, Xu T, Zhang Y, Mu H, Zhang Y, et al. Adaptation to deep-sea chemosynthetic environments as revealed by mussel genomes. Nat Ecol Evol. 2017;1:0121.

49. Gómez-Chiarri M, Warren WC, Guo X, Proestou D. Developing tools for the study of molluscan immunity: The sequencing of the genome of the eastern oyster, Crassostrea virginica. Fish Shellfish Immunol. 2015;46:2–4.

50. Du X, Fan G, Jiao Y, Zhang H, Guo X, Huang R, et al. The pearl oyster Pinctada fucata martensii genome and multi-omic analyses provide insights into biomineralization. GigaScience. 2017;6:1–12.

51. Wang S, Zhang J, Jiao W, Li J, Xun X, Sun Y, et al. Scallop genome provides insights into evolution of bilaterian karyotype and development. Nat Ecol Evol. 2017;1:0120.

52. Kenny NJ, McCarthy SA, Dudchenko O, James K, Betteridge E, Corton C, et al. The gene-rich genome of the scallop Pecten maximus. GigaScience. 2020;9.

53. Mun S, Kim Y-J, Markkandan K, Shin W, Oh S, Woo J, et al. The Whole-Genome and Transcriptome of the Manila Clam (Ruditapes philippinarum). Genome Biol Evol. 2017;9:1487–98.

54. Li C, Liu X, Liu B, Ma B, Liu F, Liu G, et al. Draft genome of the Peruvian scallop Argopecten purpuratus. GigaScience. 2018;7.

55. Powell D, Subramanian S, Suwansa-Ard S, Zhao M, O’Connor W, Raftos D, et al. The genome of the oyster Saccostrea offers insight into the environmental resilience of bivalves. DNA Res Int J Rapid Publ Rep Genes Genomes. 2018;

56. Ran Z, Li Z, Yan X, Liao K, Kong F, Zhang L, et al. Chromosome-level genome assembly of the razor clam Sinonovacula constricta (Lamarck, 1818). Mol Ecol Resour. 2019;19:1647–58.

57. McCartney MA, Auch B, Kono T, Mallez S, Zhang Y, Obille A, et al. The Genome of the Zebra Mussel, Dreissena polymorpha: A Resource for Invasive Species Research. bioRxiv. Cold Spring Harbor Laboratory; 2019;696732.

58. Calcino AD, de Oliveira AL, Simakov O, Schwaha T, Zieger E, Wollesen T, et al. The quagga mussel genome and the evolution of freshwater tolerance. DNA Res. 2019;26:411–22.

59. Renaut S, Guerra D, Hoeh WR, Stewart DT, Bogan AE, Ghiselli F, et al. Genome Survey of the Freshwater Mussel Venustaconcha ellipsiformis (Bivalvia: Unionida) Using a Hybrid De Novo Assembly Approach. Genome Biol Evol. 2018;10:1637–46.

60. Nam B-H, Kwak W, Kim Y-O, Kim D-G, Kong HJ, Kim W-J, et al. Genome sequence of pacific abalone (Haliotis discus hannai): the first draft genome in family Haliotidae. GigaScience. 2017;6:1–8.

61. Adema CM, Hillier LW, Jones CS, Loker ES, Knight M, Minx P, et al. Whole genome analysis of a schistosomiasis-transmitting freshwater snail. Nat Commun. 2017;8:15451.

62. Albertin CB, Simakov O, Mitros T, Wang ZY, Pungor JR, Edsinger-Gonzales E, et al. The octopus genome and the evolution of cephalopod neural and morphological novelties. Nature. 2015;524:220–4.

63. Zdobnov EM, Tegenfeldt F, Kuznetsov D, Waterhouse RM, Simão FA, Ioannidis P, et al. OrthoDB v9.1: cataloging evolutionary and functional annotations for animal, fungal, plant, archaeal, bacterial and viral orthologs. Nucleic Acids Res. 2017;45:D744–9.

64. Ieyama H, Kameoka O, Tan T, Yamasaki J. Chromosomes and nuclear DNA contents of some species of Mytilidae. Venus. 1994;53:327–31.

65. Rodríguez-Juíz AM, Torrado M, Méndez J. Genome-size variation in bivalve molluscs determined by flow cytometry. Mar Biol. 1996;126:489–97.

66. Hinegardner R. Cellular DNA content of the Mollusca. Comp Biochem Physiol A Physiol. 1974;47:447–60.

67. Uliano-Silva M, Dondero F, Dan Otto T, Costa I, Lima NCB, Americo JA, et al. A hybrid-hierarchical genome assembly strategy to sequence the invasive golden mussel, Limnoperna fortunei. GigaScience. 2018;7:1–10.

68. Gerdol M, Moro GD, Manfrin C, Milandri A, Riccardi E, Beran A, et al. RNA sequencing and de novo assembly of the digestive gland transcriptome in Mytilus galloprovincialis fed with toxinogenic and non-toxic strains of Alexandrium minutum. BMC Res Notes. 2014;7:722.

69. Romiguier J, Gayral P, Ballenghien M, Bernard A, Cahais V, Chenuil A, et al. Comparative population genomics in animals uncovers the determinants of genetic diversity. Nature. 2014;515:261–3.

70. Bjärnmark NA, Yarra T, Churcher AM, Felix RC, Clark MS, Power DM. Transcriptomics provides insight into Mytilus galloprovincialis (Mollusca: Bivalvia) mantle function and its role in biomineralisation. Mar Genomics. 2016;27:37–45.

71. Wagner GP, Kin K, Lynch VJ. Measurement of mRNA abundance using RNA-seq data: RPKM measure is inconsistent among samples. Theory Biosci Theor Den Biowissenschaften. 2012;131:281–5.

72. Huerta-Cepas J, Gabaldón T. Assigning duplication events to relative temporal scales in genome-wide studies. Bioinforma Oxf Engl. 2011;27:38–45.

73. Edgar RC. MUSCLE: multiple sequence alignment with high accuracy and high throughput. Nucleic Acids Res. 2004;32:1792–7.

74. Katoh K, Standley DM. MAFFT multiple sequence alignment software version 7: improvements in performance and usability. Mol Biol Evol. 2013;30:772–80.

75. Lassmann T, Sonnhammer EL. Kalign – an accurate and fast multiple sequence alignment algorithm. BMC Bioinformatics. 2005;6:298.

76. Wallace IM, O’Sullivan O, Higgins DG, Notredame C. M-Coffee: combining multiple sequence alignment methods with T-Coffee. Nucleic Acids Res. 2006;34:1692–9.

77. Capella-Gutiérrez S, Silla-Martínez JM, Gabaldón T. trimAl: a tool for automated alignment trimming in large-scale phylogenetic analyses. Bioinforma Oxf Engl. 2009;25:1972–3.

78. Guindon S, Gascuel O. A simple, fast, and accurate algorithm to estimate large phylogenies by maximum likelihood. Syst Biol. 2003;52:696–704.

79. Huerta-Cepas J, Capella-Gutiérrez S, Pryszcz LP, Marcet-Houben M, Gabaldón T. PhylomeDB v4: zooming into the plurality of evolutionary histories of a genome. Nucleic Acids Res. 2014;42:D897–902.

80. Luo Y-J, Kanda M, Koyanagi R, Hisata K, Akiyama T, Sakamoto H, et al. Nemertean and phoronid genomes reveal lophotrochozoan evolution and the origin of bilaterian heads. Nat Ecol Evol. 2018;2:141.

81. Luo Y-J, Takeuchi T, Koyanagi R, Yamada L, Kanda M, Khalturina M, et al. The Lingula genome provides insights into brachiopod evolution and the origin of phosphate biomineralization. Nat Commun. 2015;6:8301.

82. Adams MD, Celniker SE, Holt RA, Evans CA, Gocayne JD, Amanatides PG, et al. The genome sequence of Drosophila melanogaster. Science. 2000;287:2185–95.

83. Venter JC, Adams MD, Myers EW, Li PW, Mural RJ, Sutton GG, et al. The Sequence of the Human Genome. Science. 2001;291:1304–51.

84. Huerta-Cepas J, Dopazo J, Gabaldón T. ETE: a python Environment for Tree Exploration. BMC Bioinformatics. 2010;11:24.

85. Al-Shahrour F, Minguez P, Tárraga J, Medina I, Alloza E, Montaner D, et al. FatiGO +: a functional profiling tool for genomic data. Integration of functional annotation, regulatory motifs and interaction data with microarray experiments. Nucleic Acids Res. 2007;35:W91-96.

86. Finn RD, Clements J, Eddy SR. HMMER web server: interactive sequence similarity searching. Nucleic Acids Res. 2011;39:W29–37.

87. Wehe A, Bansal MS, Burleigh JG, Eulenstein O. DupTree: a program for large-scale phylogenetic analyses using gene tree parsimony. Bioinformatics. 2008;24:1540–1.

88. Mirarab S, Warnow T. ASTRAL-II: coalescent-based species tree estimation with many hundreds of taxa and thousands of genes. Bioinforma Oxf Engl. 2015;31:i44-52.

89. Zhang C, Rabiee M, Sayyari E, Mirarab S. ASTRAL-III: polynomial time species tree reconstruction from partially resolved gene trees. BMC Bioinformatics. 2018;19:153.

90. Stamatakis A. RAxML version 8: a tool for phylogenetic analysis and post-analysis of large phylogenies. Bioinforma Oxf Engl. 2014;30:1312–3.

91. Darriba D, Posada D, Kozlov AM, Stamatakis A, Morel B, Flouri T. ModelTest-NG: A New and Scalable Tool for the Selection of DNA and Protein Evolutionary Models. Mol Biol Evol. Oxford Academic; 2020;37:291–4.

92. Domeneghetti S, Varotto L, Civettini M, Rosani U, Stauder M, Pretto T, et al. Mortality occurrence and pathogen detection in Crassostrea gigas and Mytilus galloprovincialis close-growing in shallow waters (Goro lagoon, Italy). Fish Shellfish Immunol. 2014;41:37–44.

93. Fraïsse C, Belkhir K, Welch JJ, Bierne N. Local interspecies introgression is the main cause of extreme levels of intraspecific differentiation in mussels. Mol Ecol. 2016;25:269–86.

94. El Ayari T, Trigui El Menif N, Hamer B, Cahill AE, Bierne N. The hidden side of a major marine biogeographic boundary: a wide mosaic hybrid zone at the Atlantic–Mediterranean divide reveals the complex interaction between natural and genetic barriers in mussels. Heredity. Nature Publishing Group; 2019;122:770–84.

95. Vurture GW, Sedlazeck FJ, Nattestad M, Underwood CJ, Fang H, Gurtowski J, et al. GenomeScope: fast reference-free genome profiling from short reads. Bioinforma Oxf Engl. 2017;33:2202–4.

96. Li Y, Sun X, Hu X, Xun X, Zhang J, Guo X, et al. Scallop genome reveals molecular adaptations to semi-sessile life and neurotoxins. Nat Commun. 2017;8:1721.

97. Van der Auwera GA, Carneiro MO, Hartl C, Poplin R, Del Angel G, Levy-Moonshine A, et al. From FastQ data to high confidence variant calls: the Genome Analysis Toolkit best practices pipeline. Curr Protoc Bioinforma. 2013;43:11.10.1-33.

98. DePristo MA, Banks E, Poplin R, Garimella KV, Maguire JR, Hartl C, et al. A framework for variation discovery and genotyping using next-generation DNA sequencing data. Nat Genet. 2011;43:491–8.

99. Li H, Durbin R. Fast and accurate short read alignment with Burrows-Wheeler transform. Bioinforma Oxf Engl. 2009;25:1754–60.

100. Heng L. Aligning sequence reads, clone sequences and assembly contigs with BWA-MEM. arXiv. 2013;1303.3997.

101. Li H, Handsaker B, Wysoker A, Fennell T, Ruan J, Homer N, et al. The Sequence Alignment/Map format and SAMtools. Bioinforma Oxf Engl. 2009;25:2078–9.

102. Neph S, Kuehn MS, Reynolds AP, Haugen E, Thurman RE, Johnson AK, et al. BEDOPS: high-performance genomic feature operations. Bioinforma Oxf Engl. 2012;28:1919–20.

103. Riginos C, Cunningham CW. Local adaptation and species segregation in two mussel (Mytilus edulis x Mytilus trossulus) hybrid zones. Mol Ecol. 2005;14:381–400.

104. Kartavtsev YP, Katolikova MV, Sharina SN, Chichvarkhina OV, Masalkova NA. A population genetic study of the hybrid zone of Mytilus trossulus Gould, 1850 and an introduced species, M. galloprovincialis Lamarck, 1819, (Bivalvia: Mytilidae) in peter the great bay in the Sea of Japan. Russ J Mar Biol. 2014;40:208–16.

105. Inoue K, Odo S, Noda T, Nakao S, Takeyama S, Yamaha E, et al. A possible hybrid zone in the Mytilus edulis complex in Japan revealed by PCR markers. Mar Biol. 1997;128:91–5.

106. Bierne N, Borsa P, Daguin C, Jollivet D, Viard F, Bonhomme F, et al. Introgression patterns in the mosaic hybrid zone between Mytilus edulis and M. galloprovincialis. Mol Ecol. 2003;12:447–61.

107. Fraïsse C, Roux C, Welch JJ, Bierne N. Gene-flow in a mosaic hybrid zone: is local introgression adaptive? Genetics. 2014;197:939–51.

108. Rawson PD, Joyner KL, Meetze K, Hilbish TJ. Evidence for intragenic recombination within a novel genetic marker that distinguishes mussels in the Mytilus edulis species complex. Heredity. 1996;77:599–607.

109. Inoue K, Waite JH, Matsuoka M, Odo S, Harayama S. Interspecific variations in adhesive protein sequences of Mytilus edulis, M. galloprovincialis, and M. trossulus. Biol Bull. 1995;189:370–5.

110. Daguin C, Borsa P. Genetic characterisation of Mytilus galloprovincialis Lmk. in North West Africa using nuclear DNA markers. J Exp Mar Biol Ecol. 1999;235:55–65.

111. Daguin C, Bonhomme F, Borsa P. The zone of sympatry and hybridization of Mytilus edulis and M. galloprovincialis, as described by intron length polymorphism at locus mac-1. Heredity. 2001;86:342–54.

112. Ohresser M, Borsa P, Delsert C. Intron-length polymorphism at the actin gene locus mac-1: a genetic marker for population studies in the marine mussels Mytilus galloprovincialis Lmk. and M. edulis L. Mol Mar Biol Biotechnol. 1997;6:123–30.

113. Bierne N, David P, Boudry P, Bonhomme F. Assortative fertilization and selection at larval stage in the mussels Mytilus edulis and M. galloprovincialis. Evol Int J Org Evol. 2002;56:292–8.

114. Bierne N, David P, Langlade A, Bonhomme F. Can habitat specialisation maintain a mosaic hybrid zone in marine bivalves? Mar Ecol Prog Ser. 2002;245:157–70.

115. Gérard K, Bierne N, Borsa P, Chenuil A, Féral J-P. Pleistocene separation of mitochondrial lineages of Mytilus spp. mussels from Northern and Southern Hemispheres and strong genetic differentiation among southern populations. Mol Phylogenet Evol. 2008;49:84–91.

116. Stewart DT, Sinclair-Waters M, Rice A, Bunker RA, Robicheau BM, Breton S. Distribution and frequency of mitochondrial DNA polymorphisms in blue mussel (Mytilus edulis) populations of southwestern Nova Scotia (Canada). Can J Zool. 2018;96:608–13.

117. Śmietanka B, Burzyński A, Hummel H, Wenne R. Glacial history of the European marine mussels Mytilus, inferred from distribution of mitochondrial DNA lineages. Heredity. 2014;113:hdy201423.

118. Altschul SF, Gish W, Miller W, Myers EW, Lipman DJ. Basic local alignment search tool. J Mol Biol. 1990;215:403–10.

119. Faure MF, David P, Bonhomme F, Bierne N. Genetic hitchhiking in a subdivided population of Mytilus edulis. BMC Evol Biol. 2008;8:164.

120. Saitou N, Nei M. The neighbor-joining method: a new method for reconstructing phylogenetic trees. Mol Biol Evol. 1987;4:406–25.

121. Zouros E, Oberhauser Ball A, Saavedra C, Freeman KR. An unusual type of mitochondrial DNA inheritance in the blue mussel Mytilus. Proc Natl Acad Sci U S A. 1994;91:7463–7.

122. Skibinski DO, Gallagher C, Beynon CM. Sex-limited mitochondrial DNA transmission in the marine mussel Mytilus edulis. Genetics. 1994;138:801–9.

123. Kumar S, Stecher G, Li M, Knyaz C, Tamura K. MEGA X: Molecular Evolutionary Genetics Analysis across Computing Platforms. Mol Biol Evol. 2018;35:1547–9.

124. Rawson PD, Hilbish TJ. ASYMMETRIC INTROGRESSION OF MITOCHONDRIAL DNA AMONG EUROPEAN POPULATIONS OF BLUE MUSSELS (MYTILUS SPP.). Evol Int J Org Evol. 1998;52:100–8.

125. Kijewski TK, Zbawicka M, Väinölä R, Wenne R. Introgression and mitochondrial DNA heteroplasmy in the Baltic populations of mussels Mytilus trossulus and M. edulis. Mar Biol. 2006;149:1371–85.

126. Rosani U, Gerdol M. A bioinformatics approach reveals seven nearly-complete RNA-virus genomes in bivalve RNA-seq data. Virus Res. 2017;239:33–42.

127. Huelsenbeck JP, Ronquist F. MRBAYES: Bayesian inference of phylogenetic trees. Bioinforma Oxf Engl. 2001;17:754–5.

128. Lusk RW. Diverse and widespread contamination evident in the unmapped depths of high throughput sequencing data. PloS One. 2014;9:e110808.

129. Rice P, Longden L, Bleasby A. EMBOSS: The European Molecular Biology Open Software Suite. Trends Genet. 2000;16:276–7.

130. Mohanty S, Khanna R. Genome-wide comparative analysis of four Indian Drosophila species. Mol Genet Genomics MGG. 2017;292:1197–208.

131. Hu X, Xiao G, Zheng P, Shang Y, Su Y, Zhang X, et al. Trajectory and genomic determinants of fungal-pathogen speciation and host adaptation. Proc Natl Acad Sci U S A. 2014;111:16796–801.

132. Mladineo I, Petrić M, Hrabar J, Bočina I, Peharda M. Reaction of the mussel Mytilus galloprovincialis (Bivalvia) to Eugymnanthea inquilina (Cnidaria) and Urastoma cyprinae (Turbellaria) concurrent infestation. J Invertebr Pathol. 2012;110:118–25.

133. Leclère L, Horin C, Chevalier S, Lapébie P, Dru P, Peron S, et al. The genome of the jellyfish Clytia hemisphaerica and the evolution of the cnidarian life-cycle. Nat Ecol Evol. 2019;3:801–10.

134. Helmkampf M, Bellinger MR, Geib SM, Sim SB, Takabayashi M. Draft Genome of the Rice Coral Montipora capitata Obtained from Linked-Read Sequencing. Genome Biol Evol. 2019;11:2045–54.

135. Wood DE, Lu J, Langmead B. Improved metagenomic analysis with Kraken 2. Genome Biol. 2019;20:257.

136. Govindarajan AF, Boero F, Halanych KM. Phylogenetic analysis with multiple markers indicates repeated loss of the adult medusa stage in Campanulariidae (Hydrozoa, Cnidaria). Mol Phylogenet Evol. 2006;38:820–34.

137. Hardigan MA, Crisovan E, Hamilton JP, Kim J, Laimbeer P, Leisner CP, et al. Genome Reduction Uncovers a Large Dispensable Genome and Adaptive Role for Copy Number Variation in Asexually Propagated Solanum tuberosum. Plant Cell. 2016;28:388–405.

138. Mitta G, Vandenbulcke F, Hubert F, Salzet M, Roch P. Involvement of Mytilins in Mussel Antimicrobial Defense. J Biol Chem. 2000;275:12954–62.

139. Greco S, Gerdol M, Edomi P, Pallavicini A. Molecular Diversity of Mytilin-Like Defense Peptides in Mytilidae (Mollusca, Bivalvia). Antibiot Basel Switz. 2020;9.

140. Gerdol M, Venier P. An updated molecular basis for mussel immunity. Fish Shellfish Immunol. 2015;46:17–38.

141. Bailey TL, Williams N, Misleh C, Li WW. MEME: discovering and analyzing DNA and protein sequence motifs. Nucleic Acids Res. 2006;34:W369–73.

142. Rice P, Longden I, Bleasby A. EMBOSS: the European Molecular Biology Open Software Suite. Trends Genet TIG. 2000;16:276–7.

143. Roth A, Anisimova M, Cannarozzi GM. Measuring codon usage bias. Codon Evol Mech Models. 2012.

144. Gerdol M, De Moro G, Venier P, Pallavicini A. Analysis of synonymous codon usage patterns in sixty-four different bivalve species. PeerJ. 2015;3:e1520.

145. Grabherr MG, Haas BJ, Yassour M, Levin JZ, Thompson DA, Amit I, et al. Trinity: reconstructing a full-length transcriptome without a genome from RNA-Seq data. Nat Biotechnol. 2011;29:644–52.

146. Falcon S, Gentleman R. Hypergeometric Testing Used for Gene Set Enrichment Analysis. In: Hahne F, Huber W, Gentleman R, Falcon S, editors. Bioconductor Case Stud. New York, NY: Springer; 2008. p. 207–20.

147. Benjamini Y, Hochberg Y. Controlling the false discovery rate: a practical and powerful approach to multiple testing. J R Stat Soc Ser B Methodol. 1995;289–300.

148. Bork P, Holm L, Sander C. The immunoglobulin fold. Structural classification, sequence patterns and common core. J Mol Biol. 1994;242:309–20.

149. Potapov V, Sobolev V, Edelman M, Kister A, Gelfand I. Protein–Protein Recognition: Juxtaposition of Domain and Interface Cores in Immunoglobulins and Other Sandwich-like Proteins. J Mol Biol. 2004;342:665–79.

150. Barclay AN. Ig-like domains: Evolution from simple interaction molecules to sophisticated antigen recognition. Proc Natl Acad Sci U S A. 1999;96:14672–4.

151. Hanington PC, Forys MA, Loker ES. A Somatically Diversified Defense Factor, FREP3, Is a Determinant of Snail Resistance to Schistosome Infection. PLoS Negl Trop Dis. 2012;6:e1591.

152. Wang X, Wang M, Xu Q, Xu J, Lv Z, Wang L, et al. Two novel LRR and Ig domain-containing proteins from oyster Crassostrea gigas function as pattern recognition receptors and induce expression of cytokines. Fish Shellfish Immunol. 2017;70:308–18.

153. Petersen TE, Thøgersen HC, Skorstengaard K, Vibe-Pedersen K, Sahl P, Sottrup-Jensen L, et al. Partial primary structure of bovine plasma fibronectin: three types of internal homology. Proc Natl Acad Sci U S A. 1983;80:137–41.

154. Li J, Mahajan A, Tsai M-D. Ankyrin repeat: a unique motif mediating protein-protein interactions. Biochemistry. 2006;45:15168–78.

155. Mosavi LK, Cammett TJ, Desrosiers DC, Peng Z. The ankyrin repeat as molecular architecture for protein recognition. Protein Sci Publ Protein Soc. 2004;13:1435–48.

156. Gerdol M, Venier P, Pallavicini A. The genome of the Pacific oyster Crassostrea gigas brings new insights on the massive expansion of the C1q gene family in Bivalvia. Dev Comp Immunol. 2015;49:59–71.

157. Romero A, Dios S, Poisa-Beiro L, Costa MM, Posada D, Figueras A, et al. Individual sequence variability and functional activities of fibrinogen-related proteins (FREPs) in the Mediterranean mussel (Mytilus galloprovincialis) suggest ancient and complex immune recognition models in invertebrates. Dev Comp Immunol. 2011;35:334–44.

158. Resnick D, Pearson A, Krieger M. The SRCR superfamily: a family reminiscent of the Ig superfamily. Trends Biochem Sci. 1994;19:5–8.

159. Gerdol M. Immune-related genes in gastropods and bivalves: a comparative overview. Invertebr Surviv J. 2017;14:95–111.

160. Schwefel D, Fröhlich C, Eichhorst J, Wiesner B, Behlke J, Aravind L, et al. Structural basis of oligomerization in septin-like GTPase of immunity-associated protein 2 (GIMAP2). Proc Natl Acad Sci U S A. 2010;107:20299–304.

161. Reuber TL, Ausubel FM. Isolation of Arabidopsis genes that differentiate between resistance responses mediated by the RPS2 and RPM1 disease resistance genes. Plant Cell. 1996;8:241–9.

162. Kim B-H, Shenoy AR, Kumar P, Bradfield CJ, MacMicking JD. IFN-inducible GTPases in Host Defense. Cell Host Microbe. 2012;12:432–44.

163. Gotthardt K, Weyand M, Kortholt A, Van Haastert PJM, Wittinghofer A. Structure of the Roc-COR domain tandem of C. tepidum, a prokaryotic homologue of the human LRRK2 Parkinson kinase. EMBO J. 2008;27:2239–49.

164. Short KM, Cox TC. Subclassification of the RBCC/TRIM superfamily reveals a novel motif necessary for microtubule binding. J Biol Chem. 2006;281:8970–80.

165. Bridgham JT, Wilder JA, Hollocher H, Johnson AL. All in the family: evolutionary and functional relationships among death receptors. Cell Death Differ. 2003;10:19–25.

166. Liston P, Roy N, Tamai K, Lefebvre C, Baird S, Cherton-Horvat G, et al. Suppression of apoptosis in mammalian cells by NAIP and a related family of IAP genes. Nature. 1996;379:349–53.

167. Wilhelm M, Wilhelm FX. Reverse transcription of retroviruses and LTR retrotransposons. Cell Mol Life Sci CMLS. 2001;58:1246–62.

168. Dunn BM, Goodenow MM, Gustchina A, Wlodawer A. Retroviral proteases. Genome Biol. 2002;3:reviews3006.1-reviews3006.7.

169. Champoux JJ, Schultz SJ. Ribonuclease H: Properties, Substrate Specificity, and Roles in Retroviral Reverse Transcription. FEBS J. 2009;276:1506–16.

170. Kovacsovics M, Martinon F, Micheau O, Bodmer JL, Hofmann K, Tschopp J. Overexpression of Helicard, a CARD-containing helicase cleaved during apoptosis, accelerates DNA degradation. Curr Biol CB. 2002;12:838–43.

171. Khalturin K, Hemmrich G, Fraune S, Augustin R, Bosch TCG. More than just orphans: are taxonomically-restricted genes important in evolution? Trends Genet. 2009;25:404–13.

172. Breusing C, Biastoch A, Drews A, Metaxas A, Jollivet D, Vrijenhoek RC, et al. Biophysical and Population Genetic Models Predict the Presence of “Phantom” Stepping Stones Connecting Mid-Atlantic Ridge Vent Ecosystems. Curr Biol. 2016;26:2257–67.

173. Gerdol M, Fujii Y, Hasan I, Koike T, Shimojo S, Spazzali F, et al. The purplish bifurcate mussel Mytilisepta virgata gene expression atlas reveals a remarkable tissue functional specialization. BMC Genomics. 2017;18:590.

174. Leung PT, Ip JC, Mak SS, Qiu JW, Lam PK, Wong CK, et al. De novo transcriptome analysis of Perna viridis highlights tissue-specific patterns for environmental studies. BMC Genomics. 2014;15:804.

175. Sela I, Ashkenazy H, Katoh K, Pupko T. GUIDANCE2: accurate detection of unreliable alignment regions accounting for the uncertainty of multiple parameters. Nucleic Acids Res. 2015;43:W7–14.

176. Abascal F, Zardoya R, Posada D. ProtTest: selection of best-fit models of protein evolution. Bioinforma Oxf Engl. 2005;21:2104–5.

177. Whelan S, Goldman N. A General Empirical Model of Protein Evolution Derived from Multiple Protein Families Using a Maximum-Likelihood Approach. Mol Biol Evol. 2001;18:691–9.

178. Lemer S, González VL, Bieler R, Giribet G. Cementing mussels to oysters in the pteriomorphian tree: a phylogenomic approach. Proc R Soc B. 2016;283:20160857.

179. Combosch DJ, Collins TM, Glover EA, Graf DL, Harper EM, Healy JM, et al. A family-level Tree of Life for bivalves based on a Sanger-sequencing approach. Mol Phylogenet Evol. 2017;107:191–208.

180. Mitta G, Hubert F, Noël T, Roch P. Myticin, a novel cysteine-rich antimicrobial peptide isolated from haemocytes and plasma of the mussel Mytilus galloprovincialis. Eur J Biochem FEBS. 1999;265:71–8.

181. Leoni G, De Poli A, Mardirossian M, Gambato S, Florian F, Venier P, et al. Myticalins: A Novel Multigenic Family of Linear, Cationic Antimicrobial Peptides from Marine Mussels (Mytilus spp.). Mar Drugs. 2017;15:261.

182. Gerdol M, Puillandre N, Moro GD, Guarnaccia C, Lucafò M, Benincasa M, et al. Identification and Characterization of a Novel Family of Cysteine-Rich Peptides (MgCRP-I) from Mytilus galloprovincialis. Genome Biol Evol. 2015;7:2203–19.

183. Morga B, Arzul I, Faury N, Renault T. Identification of genes from flat oyster Ostrea edulis as suitable housekeeping genes for quantitative real time PCR. Fish Shellfish Immunol. 2010;29:937–45.

184. Gerdol M, Manfrin C, De Moro G, Figueras A, Novoa B, Venier P, et al. The C1q domain containing proteins of the Mediterranean mussel Mytilus galloprovincialis: a widespread and diverse family of immune-related molecules. Dev Comp Immunol. 2011;35:635–43.

185. Rossi F, Palombella S, Pirrone C, Mancini G, Bernardini G, Gornati R. Evaluation of tissue morphology and gene expression as biomarkers of pollution in mussel Mytilus galloprovincialis caging experiment. Aquat Toxicol. 2016;181:57–66.

186. Charlet M, Chernysh S, Philippe H, Hetru C, Hoffmann JA, Bulet P. Isolation of several cysteine-rich antimicrobial peptides from the blood of a mollusc, Mytilus edulis. J Biol Chem. 1996;271:21808–13.

187. Venier P, Pittà CD, Bernante F, Varotto L, Nardi BD, Bovo G, et al. MytiBase: a knowledgebase of mussel (M. galloprovincialis) transcribed sequences. BMC Genomics. 2009;10:72.

188. Rosani U, Varotto L, Rossi A, Roch P, Novoa B, Figueras A, et al. Massively parallel amplicon sequencing reveals isotype-specific variability of antimicrobial peptide transcripts in Mytilus galloprovincialis. PloS One. 2011;6:e26680.

189. Boon E, Faure MF, Bierne N. The flow of antimicrobial peptide genes through a genetic barrier between Mytilus edulis and M. galloprovincialis. J Mol Evol. 2009;68:461–74.

190. Gerdol M, De Moro G, Manfrin C, Venier P, Pallavicini A. Big defensins and mytimacins, new AMP families of the Mediterranean mussel Mytilus galloprovincialis. Dev Comp Immunol. 2012;36:390–9.

191. Jung S, Sönnichsen FD, Hung C-W, Tholey A, Boidin-Wichlacz C, Haeusgen W, et al. Macin family of antimicrobial proteins combines antimicrobial and nerve repair activities. J Biol Chem. 2012;287:14246–58.

192. Hung C-W, Jung S, Grötzinger J, Gelhaus C, Leippe M, Tholey A. Determination of disulfide linkages in antimicrobial peptides of the macin family by combination of top-down and bottom-up proteomics. J Proteomics. 2014;103:216–26.

193. Domeneghetti S, Franzoi M, Damiano N, Norante R, M El Halfawy N, Mammi S, et al. Structural and Antimicrobial Features of Peptides Related to Myticin C, a Special Defense Molecule from the Mediterranean Mussel Mytilus galloprovincialis. J Agric Food Chem. 2015;63:9251–9.

194. Pallavicini A, Costa M del M, Gestal C, Dreos R, Figueras A, Venier P, et al. High sequence variability of myticin transcripts in hemocytes of immune-stimulated mussels suggests ancient host-pathogen interactions. Dev Comp Immunol. 2008;32:213–26.

195. Costa MM, Dios S, Alonso-Gutierrez J, Romero A, Novoa B, Figueras A. Evidence of high individual diversity on myticin C in mussel (Mytilus galloprovincialis). Dev Comp Immunol. 2009;33:162–70.

196. Balseiro P, Falcó A, Romero A, Dios S, Martínez-López A, Figueras A, et al. Mytilus galloprovincialis Myticin C: A Chemotactic Molecule with Antiviral Activity and Immunoregulatory Properties. PLoS ONE. 2011;6:e23140.

197. Vera M, Martínez P, Poisa-Beiro L, Figueras A, Novoa B. Genomic Organization, Molecular Diversification, and Evolution of Antimicrobial Peptide Myticin-C Genes in the Mussel (Mytilus galloprovincialis). PLoS ONE. 2011;6:e24041.

198. Venier P, Varotto L, Rosani U, Millino C, Celegato B, Bernante F, et al. Insights into the innate immunity of the Mediterranean mussel Mytilus galloprovincialis. BMC Genomics. 2011;12:69.

199. Rey-Campos M, Novoa B, Pallavicini A, Gerdol M, Figueras A. Comparative Genomics Reveals a Significant Sequence Variability of Myticin Genes in Mytilus galloprovincialis. Biomolecules. Multidisciplinary Digital Publishing Institute; 2020;10:943.

200. Sonthi M, Cantet F, Toubiana M, Trapani M-R, Parisi M-G, Cammarata M, et al. Gene expression specificity of the mussel antifungal mytimycin (MytM). Fish Shellfish Immunol. 2012;32:45–50.

201. Cantet F, Toubiana M, Parisi M-G, Sonthi M, Cammarata M, Roch P. Individual variability of mytimycin gene expression in mussel. Fish Shellfish Immunol. 2012;33:641–4.

202. Sonthi M, Toubiana M, Pallavicini A, Venier P, Roch P. Diversity of Coding Sequences and Gene Structures of the Antifungal Peptide Mytimycin (MytM) from the Mediterranean Mussel, Mytilus galloprovincialis. Mar Biotechnol. 2011;13:857–67.

203. Zhu S, Gao B. Evolutionary origin of β-defensins. Dev Comp Immunol. 2013;39:79–84.

204. Gerdol M, Schmitt P, Venier P, Rocha G, Rosa RD, Destoumieux-Garzón D. Functional Insights From the Evolutionary Diversification of Big Defensins. Front Immunol. 2020;11.

205. Li M, Zhu L, Zhou C, Sun S, Fan Y, Zhuang Z. Molecular characterization and expression of a novel big defensin (Sb-BDef1) from ark shell, Scapharca broughtonii. Fish Shellfish Immunol. 2012;33:1167–73.

206. Zhao J, Song L, Li C, Ni D, Wu L, Zhu L, et al. Molecular cloning, expression of a big defensin gene from bay scallop Argopecten irradians and the antimicrobial activity of its recombinant protein. Mol Immunol. 2007;44:360–8.

207. Rosa RD, Santini A, Fievet J, Bulet P, Destoumieux-Garzón D, Bachère E. Big defensins, a diverse family of antimicrobial peptides that follows different patterns of expression in hemocytes of the oyster Crassostrea gigas. PloS One. 2011;6:e25594.

208. Rosa RD, Alonso P, Santini A, Vergnes A, Bachère E. High polymorphism in big defensin gene expression reveals presence-absence gene variability (PAV) in the oyster Crassostrea gigas. Dev Comp Immunol. 2015;49:231–8.
[truncated: 2,648 more chars]
