# Supplementary material for: Massive gene presence-absence variation shapes an open pan-genome in the Mediterranean mussel
Source: Genome Biol. 2020 Nov 10;21:275. doi: 10.1186/s13059-020-02180-3 (PMC7653742; doi:10.1186/s13059-020-02180-3)
Supplement: Supplementary file 3 — Additional file 3. Review history. [file 13059_2020_2180_MOESM3_ESM.docx]

Review History

**First round of review**

**Reviewer 1**

**Are you able to assess all statistics in the manuscript, including the appropriateness of statistical tests used?** Yes, and I have assessed the statistics in my report.

**Comments to author:**

This is a well-written paper describing an intensive effort to characterize the genomic architecture of the mussel Mytilus galloprovincialis. The main conclusion is that this species appears to harbor extensive gene presence/absence polymorphism. The authors interpret this outcome as evidence for a pan-genome, and with a likely adaptive basis. This is quite a claim, perhaps not quite "extraordinary", but still demands very thorough consideration of other possibilities if it is to hold up. In particular, I'd like to see the authors to thoughtfully and rigorously consider what I view is a reasonable set of perhaps more likely explanations than an adaptively maintained set of dispensable genes. If they cannot rule out these explanations, I believe that their conclusions regarding a pan-genome, and its adaptive value, are premature. I lay out these alternatives, and associated concerns, below.

Before I get to these concerns, I should note that I am puzzled by the authors' repeated claims (e.g., p. 5, line 31; p. 7, lines 14-19; p. 10, lines 38-41, p. 14, line 14) that this is the first-ever reported example of a metazoan pan-genome. Just a bit of googling revealed decade-old evidence for pan genomes in humans (Li et al. 2010, Nature Biotechnology 28: 57, https://www.nature.com/articles/nbt.1596) along with a much more recent report( Sherman et al. 2019, Nature Genetics 51: 30, https://www.nature.com/articles/s41588-018-0273-y; Sherman et al. 2019). There is also a paper describing evidence for a pan-genome in pigs (Tian et al. 2019, Science China Life Sciences 1-14, https://link.springer.com/article/10.1007%2Fs11427-019-9551-7).

Alternative (and non-mutually exclusive) explanations

1. Contamination. This is an obvious explanation for many seemingly odd genomics outcomes. Unfortunately, I do not believe that the authors have fully excluded this possibility. My primary concern has to do with the fact that the "confirmatory" PCRs apparently used the same DNA extractions and/or the same individuals used for the genomic sequencing. If contamination of these DNA extractions is what led to spurious results in genomic data, the "confirmatory" data will be no better. The gold standard of confirmation in this case would be to generate new DNA extractions, and ideally in a different laboratory setting as well. I also suggest that the authors consider using Blobtools (https://blobtools.readme.io/docs) to identify potential contaminants that might not be detected by the decontamination steps that they took.  

2. (Allo)Polyploidy events that have diploidized at different rates in different M. galloprovincialis lineages. This phenomenon is hinted at by the high number of genes, in particular, as well as clear evidence of introgression from other mussel species.

3. Contagious cancer: This disease is rampant in other mussel species (including a new report in eLife, Yonemitsu et al. 2019, https://elifesciences.org/articles/47788), is characterized by DNA sequences that look to be of intraspecific or close heterospecific origin, and can translate into "extra" genes or alleles (including a gene that gets a lot of attention in the manuscript, elongation factor 1 alpha; Fig. 1 Yonemitsu et. al. 2019). Also consistent with potential for a link to contagious cancer is the enrichment of genes involved in stress response and survival in the variable fraction of the M. galloprovincialis genome: because these contagious cancer cells are thought to disseminate through water currents, it seems reasonable to expect that they would need to resist a lot of environmental stressors. Cancer as an explanation for PAV would indicate that pathology, not adaptation, underlies what appears to be a pan-genome.

Specific comments
1. P. 6, line 25: "of", not "by".
2. P. 6, line 27, other places in the MS: no hyphen needed after "randomly" because no hyphenation needed after an adverb.
3. P. 6, line 32: citation needed after mention of resistance to stress and infection.
4. P. 6, line 35: comma after (4), delete "and" after (4).
5. P. 6, line 37: "experiencing", not "experimenting".
6. P. 6, line 57: word missing, perhaps, in "we built and improved genome assembly"...
7. P. 6, lines 56-58: There is no information given either here or in the supplemental information to justify the selection of these particular individuals or populations for sequencing.
8. P. 7, line 7, line 14: delete "remarkably" and "unexpected", respectively - let the reader decide for themselves how to interpret these results.
9. P. 7, line 9: "to", not "with".
10. Figure 1 caption: Insert word "Open" in front of "Circles represent..." to indicate that you are describing the unfilled circles.
11. Figure 2: Use the same colors (e.g., orange) for populations across figure panels: like Galician is orange in panel A but blue in panel C. What "circles" refer to in panel C is also not clear.
12. Figure 3: Explain why myticalin, a gene that seemed absent everywhere, was included in this experiment.

**Reviewer 2**

**Are you able to assess all statistics in the manuscript, including the appropriateness of statistical tests used?** There are no statistics in the manuscript.

**Comments to author:**

I enjoyed very much reading the manuscript "Massive gene presence/absence variation in the mussel genome as an adaptive strategy: first evidence of a pan-genome in Metazoa" by Gerdol and collaborators. The study explores the variation in gene complement in populational genomes of the mussel Mytilus galloprovincialis. Interestingly, they find evidence for massive differences in the gene complements of different individuals, with 45k genes shared by all the individuals and 15k that are variable. The study is well designed and written, and the analyses robust. I am convinced the pattern the authors see in their data is real and not and artefact. The findings are very relevant to our understanding of biodiversity and genome variation in animal populations. I would like to congratulate the authors for their work.

I have a minor philosophical disagreement on the meaning of a pangenome. However, I am happy to be proven wrong. Originally the concept of pangenome was also linked to the concept of public goods, the idea that the dispensable genes could contribute to the fitness of other members of the population either by being shared by lateral gene transfer (e.g. antibiotic resistance genes) or environmental "pooling" of their products. This might be the case in some eukaryotes like Emiliana huxleyi. But in the case of animals (or even plants) what is the difference between a pangenome and just an exacerbated populational polymorphism? Stretching the argument a lot, is the difference in gene complement between female and male mammals (due to a small Y chromosome) also considered a pangenome? This does not diminish the findings in this study, the presence/absence polymorphism in these mussels is brutal and outstanding. But I am concerned that there is a trend towards coming up with new buzzwords that just apply to concepts that have been in the field for decades. I would like to hear the authors opinion in that subject.

Other than this minor philosophical disagreement, "pangenomes" have been often associated with the activity of transposable elements. I wonder if the analyses show any evidence of jumping genes activity in these genomes. Also, there is no discussion on the possible mechanisms that allow the dispensable genes to arise.

Importantly, the study makes no mention of the gender of the individuals selected. I believe these mussels have separated sexes, but I have no idea about the basis of their sex determination. Is it possible that all or a part of these dispensable genes are really sex-linked genes?

I have some other comments and questions that I hope the authors can kindly address:
1)     In the significance statement, page 5 line 47, "large-scale structural variations which involve the loss of genomic regions encoding protein coding genes in some individuals (i.e. presence/absence variation, PAV)". In a similar manner to indels, I wonder if these changes can really be polarised. Are they really "losses" of regions, or just "absence" (ancestral or derived)?
2)     Figure 1 and associated analyses, I wonder why the study left out Crassostrea gigas, which next to Lottia is possibly one of the best sequenced and annotated mollusc genomes. Moreover, there are dozens of developmental transcriptomes associated to it, which could be very helpful to track the functions of some of the genes of interest.
3)     Use of term "phylostratigraphy", I wonder if the analyses here really use the same approach developed by Domazet-Lošo, Tautz and others (e.g., one way BLAST from a single "query" species against a depauperate taxon sampling of other taxa, assuming one to one relationships from the BLAST output). This approach has major flaws that have been criticised in the literature (see papers by Moyers or Casola in MBE and GBE). Some of the authors of this study are world-leading experts on orthology assignment, so I guess they would avoid such methods to the best of their judgement. I think this is not the case, as a paper by Huerta-Cepas is cited. Mapping origin of traits to a node in the phylogeny based on the taxonomic presence of these traits and the evolutionary relationships of organisms is nothing new or that deserves a new term.
4)     Figure 2B, the axis could use some labels.
5)     The paper keeps insisting that "pangenomes" have never been described in metazoans before, but then keep citing a paper on pangenomes of human African populations. I'd suggest to rephrase this to "never described in invertebrates". Otherwise, this is an unsupported claim of unprecedented findings.
6)     Page 9, line 19, "most genes present in single-copy" I am not sure if the text refers to zygosity (alleles) or to orthologs (single-copy). If the first, this sentence would need rephrasing.
7)     There is a disconnect between the sections of the paper describing the presence/absence variation and the function of the genes. I find the later comes too late. I'd humbly suggest to move up the section "PAV genes may be associated with survival one" before "The mussel pan-genome".

**Reviewer 3**

**Are you able to assess all statistics in the manuscript, including the appropriateness of statistical tests used?** No, I do not feel adequately qualified to assess the statistics.

**Comments to author:**

This manuscript presents a new reference genome for the mussel Mytilus galloprovincialis, as well as resequencing data for 14 additional individuals. Based on the resequencing data, the authors conclude that there is evidence for extreme presence/absence variation (PAV) in M. galloprovincialis, with 45,518 genes identified in all individuals, called core genes, and 14,820 genes missing in at least one individual, called dispensable genes. The PAV phenomenon has been described in bacteria and plants, and to some degree in humans, but has not been described in other metazoans, and certainly not to this scale. This result, if verified, is highly significant and of interest to a wide range of biologists. However, there are a number of unresolved questions about the results as presented that should be addressed prior to publication.

Although the main text is well written, I found it very difficult to interpret the results based on the main text alone. The supplement contains a massive amount of information that is challenging to parse. The essential information is all included, but because so much necessary information is only in the supplement, I wonder if this manuscript might be better suited to a longer-format article or journal. Much of the text of the main manuscript deals with ideas about the origins and function of PAV in mussels, most of which is largely speculative. While the discussed ideas are intriguing, I felt that documentation of the evidence for the PAV phenomenon should receive more space in the main text.

My background is in non-model animal genome assembly, and as such, I was less familiar with the PAV phenomenon and the processes used to identify it. I found a review entitled "Towards Plant Pangenomics" by Golicz, Batley, and Edwards extremely helpful in assessing the evidence for PAV in this manuscript. Those authors discuss the following four factors that have significant impact on pangenome analyses: assembly quality, annotation quality, orthologous gene detection, and selection of appropriate samples. The remainder of my review will discuss these four points.

1) Assembly Quality

The new reference genome for Mytilus galloprovincialis, assembled using a multi-step hierarchical strategy with both long and short reads, has considerably better assembly statistics than previous versions. The assembly methods are quite thorough, and the authors employ numerous strategies to counteract various potential issues. I applaud the authors' thorough reporting of the multiple assemblies in the supplement. Understandably, the different approaches lead to assemblies with different potential problems. For example, the mg10 assembly has a less complete CEGMA/BUSCO gene set, but fewer duplicated genes.

The results of downstream analyses are conducted using different assemblies, and it can be difficult to keep track of which assembly was used for a given analysis, particularly in the main text. For the most part, this is not likely to be a big issue, given that the assembly stats that are used in different contexts are reasonably similar, and the supplement does include these details most of the time. That being said, a flow chart or similar diagram might help the reader keep track of which assembly (and subsequently, which annotation), is being used for downstream analyses.

The genomes of 14 additional individuals were resequenced with short-read sequencing. Theoretical sequencing coverage for resequenced individuals ranged from 28-46X.  In section 12.1 of the supplement, the authors state "the fragmented nature of the de novo assembled genomes (see Table S37), due to the use of short reads only (Illumina paired-end), prevented a validation approach based on genome re-annotation and a comprehensive comparative analysis of annotated genes between Lola and the 15 resequenced genomes." Ultimately, in my opinion this analysis is needed for the PAV result to be fully convincing. I appreciate that this is beyond the scope of the current manuscript, but in the absence of a more comprehensive comparative analysis, the presentation of the PAV results should be toned down.

One method that could be used to strengthen the results using the data already in hand would be an iterative assembly  aimed at producing a non-redundant pan-genome assembly (described in Golicz, Batley, and Edwards 2016).     


2) Annotation quality

The authors use a range of both ab initio and RNAseq-based annotation methods. The approach appears to be thorough, but I had difficulty interpreting the annotation results.      Annotation was conducted using the mg8 and mg9 assemblies,
not mg10, the assembly optimized to have fewer issues with gene duplications. I understand that this likely has to do with the timeline of the project, and the challenges of lengthy genome projects with large teams of scientists. However, given that duplications could have a significant impact on the conclusions of the paper, this could be a concern.

Additionally, I found it very difficult to keep track of the predicted gene sets, and what method was used to generate them. For example, Supplementary Table S8 summarizes functional annotation results based on assembly mg8. Perhaps I missed something, but it was not clear to me how the numbers in the top two lines of this table were generated. The top line of Table S8 gives 67,440 genes and 85,682 proteins in the MGAL8A annotation.  Section 2.4 states "the final MGAL9D annotation is made of 58,125 genes, whose 79,966 transcripts encode 75,788 unique protein products." The main body of the text says there are 60,338 protein-coding genes in the Lola genome. I cannot determine the source of this 60,338 number. The PAV result is dependent on appropriate gene prediction, and as such, this section should be much clearer.

3) Orthology determination

Figure 1 and supplementary data note 5 deal with phylome analyses indicating that there is a high gene duplication rate in the Mytilida lineage, with 23,815 genes inferred to have been duplicated in the LCA of Mytilida. There are even more duplications in Mytilus, with 35,027 genes inferred to have duplicated after Mytilus split from the rest of Mytilida. I have a few concerns about this analysis. First, it is not indicated which gene set was used for the phylome analyses. Given the variation in numbers of genes between sets, this information is needed. Secondly, these analyses are based on the assumption that the gene numbers are not inflated. If the numbers of predicted gene models were inflated somehow, the pattern would likely look like 'multiple lineage-specific duplication events that took place after the split between Mytilus and the rest of Mytilida".  Over 9,000 genes are members of large expansions with >20 in-paralogs, which could alternatively be the result of gene modeling errors. In other areas of the manuscript, it is mentioned that there are large numbers of genes of unknown function in these large Mytilus-specific duplications. I would be very interested to see some additional data on these large duplications, including a few example phylogenies of the gene family expansions, to help verify that these in-paralogs are not redundant (i.e., they are in fact all unique duplication products). I'd also like to see a further breakdown of the cluster sizes for the gene clusters included in the >20 cluster size bar in Figure S17. What is the highest number of in-paralogs for a single cluster?


4) Selection of appropriate samples
The selection of 14 individuals from separate populations in Spain and Italy is likely to provide a good baseline for understanding the pangenome in M. galloprovincalis.

Other notes:

Given that there are multiple publications about the human pan-genome, the subtitle "first evidence of a pan-genome in Metazoa" is inaccurate.


In supplementary data note 18, one of the key examples of PAV is EF1a-bis, a dispensable pseudogene paralogous to EF1a. Based on the way the main manuscript is written, I expected the dispensable genes to be functionally transcribed genes. As such, I was surprised to see a pseudogene as a key example. I tried to determine from the supplement the percentage of dispensable genes that are identified as pseudogenes, and was unable to do so. The concept of "core pseudogenes" is also discussed in the supplement in the context of the canonical mytillin cluster. Are these "core pseudogenes" included in the 45,000 core gene set?

Very minor comments:

Out of only curiosity: why is the individual called Lola?

Supplementary Data Table 50 can't be read in the current version- all the symbols show up as a box with a question mark.

Reference:
"Towards plant pangenomics" 2016 Golicz, A.A., J. Batley, D. Edwards. Plant Biotechnology Journal. 14:1099-1105

**Authors Response**

AU: Dear Editor, we appreciate the thorough assessment made by the three peer reviewers and the useful and constructive comments they have provided. At the same time, we are grateful for the kind opportunity given by the Editor to extend the revision deadline during this difficult Covid-19 outbreak, which allowed us to carry out all the necessary revisions in an appropriate timeframe. We have carefully considered all the suggestions listed by the referees and we provided a detailed point-by-point response letter to address all the concerns pointed out during the first round of peer-review. Whenever necessary, we have carried out additional experiments to provide further support to our main conclusions and we have restructured the main text to match the regular format of Genome Biology articles, including a Methods section. We hope that our response, together with the new data included in this revised version and the updated structure of the main text, have contributed to significantly improve the quality of the manuscript, and we are fully prepared to undertake further modifications and provide additional clarifications if these will be deemed necessary by the reviewers.

Please note that, based on the suggestions provided by the reviewers, the title of the manuscript has been changed to “Massive gene presence/absence variation in the Mediterranean mussel shapes an adaptive open pan-genome”.

**REVIEWER #1:**

**1.1 This is a well-written paper describing an intensive effort to characterize the genomic architecture of the mussel Mytilus galloprovincialis. The main conclusion is that this species appears to harbor extensive gene presence/absence polymorphism. The authors interpret this outcome as evidence for a pan-genome, and with a likely adaptive basis. This is quite a claim, perhaps not quite "extraordinary", but still demands very thorough consideration of other possibilities if it is to hold up. In particular, I'd like to see the authors to thoughtfully and rigorously consider what I view is a reasonable set of perhaps more likely explanations than an adaptively maintained set of dispensable genes. If they cannot rule out these explanations, I believe that their conclusions regarding a pan-genome, and its adaptive value, are premature. I lay out these alternatives, and associated concerns, below.**

AU1.1: We appreciate the comments provided by reviewer #1, with particular reference to the alternative explanations that have been proposed, as we agree these had not been adequately discussed or investigated in the original version of the manuscript. The three alternative hypotheses proposed by the referee stem from reasonable observations and scenarios that may explain the observed patterns in other contexts. However, in our opinion, neither contamination, nor allotetraploidy and a transmissible cancer can provide a valid explanation to the complex genomic situation highlighted in our manuscript. Below we develop our argumentation.

**1.2 Before I get to these concerns, I should note that I am puzzled by the authors' repeated claims (e.g., p. 5, line 31; p. 7, lines 14-19; p. 10, lines 38-41, p. 14, line 14) that this is the first-ever reported example of a metazoan pan-genome. Just a bit of googling revealed decade-old evidence for pan genomes in humans (Li et al. 2010, Nature Biotechnology 28: 57,** [**https://www.nature.com/articles/nbt.1596**](https://www.nature.com/articles/nbt.1596)**) along with a much more recent report( Sherman et al. 2019, Nature Genetics 51: 30,** [**https://www.nature.com/articles/s41588-018-0273-y**](https://www.nature.com/articles/s41588-018-0273-y)**; Sherman et al. 2019). There is also a paper describing evidence for a pan-genome in pigs (Tian et al. 2019, Science China Life Sciences 1-14,** [**https://link.springer.com/article/10.1007%2Fs11427-019-9551-7**](https://link.springer.com/article/10.1007%2Fs11427-019-9551-7)**).**

AU1.2: The referee is right, and a similar comment has been also made by the other two reviewers. We have modified the text in the revised version of the manuscript, toning down our claim and focusing on the “exceptionality” of the mussel pan-genome rather than on its novelty in the animal kingdom. Still, we believe that previous animal pan-genomes reports do not describe such a widespread phenomena as the one we found in this mussel. Please note that the title of the manuscript has also been changed in order to put more emphasis on the gene PAV aspect of this work (“Massive gene presence/absence variation in the Mediterranean mussel shapes an adaptive open pan-genome”).

In our opinion, the exceptionality of the mussel pan-genome compared with the (few) other known metazoan pan-genomes, stands in two key aspects: (i) its size relative to the reference genome assembly and (ii) its seemingly adaptive nature, with a massive involvement of protein-coding genes. In the revised version, we make clear upfront that we refer to the original “gene-centric” definition of pan-genome provided by Medini and colleagues (Current Opinion in Genetics & Development, 2005). Neither the human, nor the pig pan-genomes properly fall within this definition, as the dispensable genomic regions not present in the reference genome assemblies mostly correspond to inter-genic regions and are seldom described as associated with protein-coding genes.

In the new version of this manuscript, based on the 16 individuals sequenced, we provide an estimate of the mussel pangenome size, which includes ~580 Mb of DNA sequence not included in the reference genome, extending its size (~1.28 Gb) by 45%. The study by Sherman and colleagues, which analyzed a much higher number of different individuals (i.e., 910), only reported 296 Mb genomic sequence not included in the reference human genome, indicating a much smaller size of the accessory fraction of the human pan-genome (~9.5%). The size of the pig pan-genome, estimated based on a number of individuals similar to those we studied in mussel (12) revealed the presence of 72.5 Mb additional genomic sequence, accounting for about 3% of the pig haploid genome size. Overall, we believe that these metrics reveal quite clearly that the extent of this phenomenon is much more relevant in mussels than it is in humans and pigs (i.e. 5X and 15X larger).

The second key factor that in our opinion marks the exceptional character of the mussel pan-genome is the association of dispensable genomic regions with protein-coding genes. Moreover, PAV is clearly not associated with random genes, but we have demonstrated that there is a clear association with specific gene families and function, which overall suggest that the mussel pan-genome has an adaptive nature. Nearly 15,000 genes, i.e., 25% of those annotated in the reference genome, plus 5,000 additional genes annotated in the recursively reassembled pan-genomic contigs, were found to be subject to PAV. On the other hand, this phenomenon does not seem to occur with high frequency neither in humans, nor in pigs. A study by Sudmant and colleagues (2015), which investigated 2,000 human genomes, observed homozygous gene deletions for just 240 genes in healthy individuals, which may suggest their dispensability. This estimate, which accounts for 1.17% of the human protein-coding genes, is about 20 times lower than our observations in the mussel pan-genome, where we identified about 15,000 dispensable genes subject to PAV, 25% of the total. In their 2019 paper, Tian and colleagues do not provide an estimate of the number of genes subject to PAV in pig and only discuss a single specific case of a gene subject to PAV, the TIG3 gene.

**1.3 Alternative (and non-mutually exclusive) explanations: Contamination. This is an obvious explanation for many seemingly odd genomics outcomes. Unfortunately, I do not believe that the authors have fully excluded this possibility. My primary concern has to do with the fact that the "confirmatory" PCRs apparently used the same DNA extractions and/or the same individuals used for the genomic sequencing. If contamination of these DNA extractions is what led to spurious results in genomic data, the "confirmatory" data will be no better. The gold standard of confirmation in this case would be to generate new DNA extractions, and ideally in a different laboratory setting as well. I also suggest that the authors consider using Blobtools (**[**https://blobtools.readme.io/docs**](https://blobtools.readme.io/docs)**) to identify potential contaminants that might not be detected by the decontamination steps that they took.**

AU1.3: Exogenous contamination has been the first issues we have tried to sort out in the first place, being well aware of other similar cases where this factor has previously led to erroneous interpretations (see for example the controversial case of tardigrades, Boothby et al. 2015 and the subsequent publications by Bemm et al., 2016 and Kotsovoulous et al. 2016. Exogenous contamination, either from microalgae, zooplankton, or bacteria dispersed in seawater is indeed a potential issue in a filter-feeding marine organism.

There are a number of ways and tools to assess the presence of exogenous contamination in raw sequencing data and assemblies, that we have strictly applied. Overall, we can provide multiple lines of evidence that we believe allow to rule out with near certainty any possibility that the observed patterns of presence/absence variation are due to contamination.

First and foremost, the reference assembly was generated from a single individual (Lola), and only the genes annotated found in this individual were tested for PAV. Therefore, if any contamination was present, then we need to assume that contamination was introduced with the sequencing of this single individual. Starting from this observation, our reply to the alternative hypothesis proposed by the reviewer will be structured in different points:

1) As far as the reference genome is concerned, we also obtained sequencing data from a second tissue (gills), with genomic DNA extracted at a different time (more than one year after the initial sequencing of the mantle gDNA sample). PE Illumina libraries were also prepared and sequenced by different companies and at different times. As reported in the supplementary material, the average coverage of all contigs in the two samples were highly correlated a R2 value = R2 = 0.917 and p-value for linear correlation < 0.0000001. Therefore, if any contamination was present in Lola, then we would need to assume that is was present in exactly the very same amount in the two tissues analyzed, which does not seem very likely.
2) In Lola, we did not observe any significant difference in the sequencing coverage of dispensable genes from the expected “hemizygous peak” (i.e. half of the expected coverage of homozygous regions). This would have only happened in the extremely unlikely situation of having an amount of exogenous DNA equal to exactly half the amount of mussel genomic DNA. In other words, this could have only happened if the number of sampled contaminating cells was equal to half the number of sampled mussel cells. We believe this is not a realistic possibility, considering the long-standing experience in bivalve dissection and mussel histology of the people responsible of this task.
3) The dispensable genes identified in Lola were found repeatedly in several re-sequenced genomes, obtained from different populations and at different times and, whenever present, they almost invariably showed a sequencing coverage consistently with their presence in hemizygous genomic regions. This observation cannot be explained by contamination, unless we assume that the very same rate of mussel DNA to exogenous DNA abundance was maintained in all the 15 independently sampled mussels, which seems almost impossible.
4) In further confirmation of the observations reported in points #1-3, please note that dispensable genes were found in completely different combinations across the 15 re-sequenced individuals. Hence, the observed pattern of PAV could not be explained by the presence of a single contaminant species, but it would require the contemporary presence of at least a dozen different sources of contamination, all present in the same amount in the same individual, but with a different combination across individuals. We believe that this scenario is extremely unlikely.
5) One of the steps that was included in the early stages of assembly was a filtering step of contaminants detected with KRAKEN 2 (see Wood and Salzberg, 2014). This tool is a k-mer based read taxonomic assignment tool, which can effectively detect contamination in a sequencing dataset, assuming the genomic sequences of the contaminating organisms are deposited in public databases (like Blobtools does). No significant contamination was found in Lola, but this tool was very useful for detecting exogenous contamination in another re-sequenced individual (i.e., an hydrozoan parasite which lives attached to mussel mantle, see the new information included in supplementary data note 14.5), which highlights its potential usefulness if real contamination is present.
6) GC content and, most importantly, codon usage data, show high consistency for core and dispensable genes (this data was already present in the supplementary material of the original submission). Both parameters are broadly used to detect species-specific signatures (and can be used by Blobtools as well), as they are strongly linked with genome composition. Dispensable and core genes share nearly identical GC content and codon usage bias (of >50 bivalve species tested, they cluster together), pointing out a common taxonomic origin of core and dispensable genes.
7) We implemented the use of Blobtools, as suggested by the reviewer, and the main results have been included in the supplementary material. We observed a very narrow distribution of GC content for all contigs, and a high confidence taxonomic assignment to the *Mytilus* genus for the overwhelming majority of mapped reads, with a negligible fraction those that could be mapped to the assembly (<0.5%) flagged as suspicious (in most cases due to a lack of hits).
8) The PAV patterns we experimentally observed on parent and full-siblings by PCR would not be consistent with contamination. On the other hand, they confirm the Mendelian inheritance of dispensable genes.

We have extended the text included in the supplementary material with additional details and analyses to provide a full overview of the decontamination process. This has been also rigorously applied to the recursively reassembled pan-genomic contigs, as detailed in Supplementary Data Note 14.

We would also like to clarify that confirmatory PCRs had to be carried out on the very same individuals subjected to WGR to validate *in silico* predictions. Nevertheless PCR analyses were also extended on three families of three full-sib mussels (including two parents and three F1 offspring each), which confirmed the existence of the PAV phenomenon and the Mendelian inheritance of *dispensable* genes (see Supplementary Data Note 12.3).

**1.4 (Allo)Polyploidy events that have diploidized at different rates in different M. galloprovincialis lineages. This phenomenon is hinted at by the high number of genes, in particular, as well as clear evidence of introgression from other mussel species.**

AU1.4: Allopolyploidy would not be consistent with literature on the subject and available cytogenetic information on Mytilidae. Natural allopolyploids have never been documented in mussels and the karyotype of various species within the *Mytilus* genus has been previously characterized to be highly conserved (2n=28, see Thiriot-Quiévreux, 1994), with some morphological differences across species but a number of shared rRNA and histone markers (see Pérez-García et al. 2014 or Martinez & González-Tizón 2011). Karyotype conservation further extends beyond the *Mytilus* genus, to other Mytilidae, which all show a 2n chromosome number ranging from 24 to 32 (see Thiriot-Quiévreux 2002), with no evidence of whole-genome duplication (WGD).

The phylome analysis included in this work in any case would have been able to detect traces of allopolyploidy, if it had actually occurred. Although this highlighted a high gene duplication rate in *Mytilus* compared with other bivalves, the phylome suggests that this is the result of recent massive gene family-specific duplications, not of ancestral allopolyploidy. In further support of this conclusion, the vast majority of the core metazoan housekeeping genes (as highlighted by the BUSCO analysis) do not show duplication, and the overall gene duplication rate was in line or even lower than the other fully sequenced bivalve genomes. Nevertheless, a sentence was added to the introduction section to clarify that no evidence of past WGD or allopolyploidization events exists at the present time for this species.

**1.5 Contagious cancer: This disease is rampant in other mussel species (including a new report in eLife, Yonemitsu et al. 2019,** [**https://elifesciences.org/articles/47788**](https://elifesciences.org/articles/47788)**), is characterized by DNA sequences that look to be of intraspecific or close heterospecific origin, and can translate into "extra" genes or alleles (including a gene that gets a lot of attention in the manuscript, elongation factor 1 alpha; Fig. 1 Yonemitsu et. al. 2019). Also consistent with potential for a link to contagious cancer is the enrichment of genes involved in stress response and survival in the variable fraction of the M. galloprovincialis genome: because these contagious cancer cells are thought to disseminate through water currents, it seems reasonable to expect that they would need to resist a lot of environmental stressors. Cancer as an explanation for PAV would indicate that pathology, not adaptation, underlies what appears to be a pan-genome.**

AU1.5: While contagious cancer may be considered rampant in some bivalves, its prevalence in *Mytilus* is actually extremely low and it has been documented in just a very few occasions so far, apart from the report by Yonemitsu and colleagues. On the other hand, non-transmissible disseminated neoplasia (not to be confused with the disease reported by Yonemitsu) has been documented for several decades in mussels, but always at very low prevalence in *M. galloprovincialis* populations (this prevalence is higher in the congeneric species *M. trossulus*). To the best of our knowledge, transmissible cancer has been so far only demonstrated in *Mytilus* on two occasions, i.e. Yonemitsu et al. 2019 and Metzger et al, 2016.

We believe multiple lines of evidence make this hypothesis quite unlikely as a possible explanation for PAV in mussel:

1) The presence of alleles with heterospecific origin (for example in the case of EEF1A1) was not surprising. On the contrary, it was expected due to the well-documented genetic introgression that characterizes the *Mytilus edulis* species complex and natural occurrence of hybrid zones. It is not infrequent to observe *M. edulis* or even *M. trossulus* alleles in Mediterranean *M. galloprovincialis* populations, as revealed by multiple studies published over the past few years by Bierne and colleagues. Based on the state of the art, we do not think that the occasional observation of *M. edulis* or *M. trossulus* alleles in *M. galloprovincialis* should be considered as evidence of transmissible cancer in mussel.

2) The presence of disseminated neoplasia is actually quite evident from histological examination. All the sampled mussels have been carefully examined morphologically for sex assignment by co-authors with extensive experience in bivalve pathology, without noticing any sign of neoplasia.

3) Disseminated neoplasia would have led to the presence of different mitochondrial lineages within the same sample. Mitochondrial markers have been analyzed in detail, as explained in the supplementary material, and in no case could we collect observations compatible with the presence of contagious cancer.

4) Similar to our reply to the contamination hypothesis, the observed uniform heterozygous sequencing coverage of dispensable genes provides a strong case against their origin from neoplastic cells. If cancer was present, we would have expected to have a mixture of “normal” cells (in large excess) and a few cancerous cells, most certainly far from a 1:1 abundance rate. Any contig o gene derived from cancer cells of exogenous origin would have been reasonably characterized by a low and variable sequencing coverage in different individuals. We would like to further remark the fact that in most cases dispensable genes either showed a coverage equal to zero or close to the heterozygous peak of coverage (see the violin plots in the main text, further detailed in the supplementary material).

**Specific comments**

1. **P. 6, line 25: "of", not "by".**

AU: Corrected.

2. **P. 6, line 27, other places in the MS: no hyphen needed after "randomly" because no hyphenation needed after an adverb.**

AU: Corrected.

3. **P. 6, line 32: citation needed after mention of resistance to stress and infection.**

AU: A reference has been added.

4. **P. 6, line 35: comma after (4), delete "and" after (4).**

AU: Corrected.

5. **P. 6, line 37: "experiencing", not "experimenting".**

AU: Corrected.

6. **P. 6, line 57: word missing, perhaps, in "we built and improved genome assembly"...**

AU: Corrected.

7. **P. 6, lines 56-58: There is no information given either here or in the supplemental information to justify the selection of these particular individuals or populations for sequencing.**

AU: Thank you for pointing this out. This information has been added to the supplementary material. We randomly selected six individuals from the two populations trying to maintain an equal number of male and female individuals to test for the role of this factor on the patterns we had initially observed. Later, two additional male individuals from Galicia were analyzed to test whether the maturation stage had any effect on PAV patterns. The selection of Galicia and the Adriatic sea as sampling sites was made in part due to practical reasons and in part because two different *M. galloprovincialis* lineages have been described by the use of nuclear molecular markers in the studies by Bierne and colleagues, i.e. an “Atlantic lineage”, exemplified by the population sampled at Ria de Vigo, and a “Mediterranean lineage”, exemplified by the population sampled at Sacca di Goro.

8. **P. 7, line 7, line 14: delete "remarkably" and "unexpected", respectively - let the reader decide for themselves how to interpret these results.**

AU: Corrected.

9. **P. 7, line 9: "to", not "with".**

AU: Corrected.

10. **Figure 1 caption: Insert word "Open" in front of "Circles represent..." to indicate that you are describing the unfilled circles.**

AU: Thank you for this observation. This was fixed.

11. **Figure 2: Use the same colors (e.g., orange) for populations across figure panels: like Galician is orange in panel A but blue in panel C. What "circles" refer to in panel C is also not clear.**

AU: Thank you for this observation. We have revised the colors in this figure to simplify its interpretation.

12. **Figure 3: Explain why myticalin, a gene that seemed absent everywhere, was included in this experiment.**

AU: Our intention was to include dispensable genes found with different frequencies in mussel populations – EEF1A1 as a core gene, the E3 ubiquitin ligase 1 as a dispensable gene with a medium/low frequency of occurrence (3 out of 15 re-sequenced individuals) and myticalin B as a dispensable gene with a very low frequency of occurrence (just one re-sequenced individual). Therefore, in Figure 3 myticalin B mostly served its purpose in panel A, whereas its inclusion in panel B served as a negative control, pretty much like EEF1A1 served as a positive control for a core gene present in all individuals.

**REVIEWER #2:**

**2.1 I enjoyed very much reading the manuscript "Massive gene presence/absence variation in the mussel genome as an adaptive strategy: first evidence of a pan-genome in Metazoa" by Gerdol and collaborators. The study explores the variation in gene complement in populational genomes of the mussel Mytilus galloprovincialis. Interestingly, they find evidence for massive differences in the gene complements of different individuals, with 45k genes shared by all the individuals and 15k that are variable. The study is well designed and written, and the analyses robust. I am convinced the pattern the authors see in their data is real and not and artefact. The findings are very relevant to our understanding of biodiversity and genome variation in animal populations. I would like to congratulate the authors for their work.**
AU2.1: We want to thank the reviewer for her/his kind comment and appreciation of the work done. In the present version of the manuscript, we have tried to further strengthen the results with additional analyses stemming from the observations provided by the three referees.

**2.2 I have a minor philosophical disagreement on the meaning of a pangenome. However, I am happy to be proven wrong. Originally the concept of pangenome was also linked to the concept of public goods, the idea that the dispensable genes could contribute to the fitness of other members of the population either by being shared by lateral gene transfer (e.g. antibiotic resistance genes) or environmental "pooling" of their products. This might be the case in some eukaryotes like Emiliana huxleyi. But in the case of animals (or even plants) what is the difference between a pangenome and just an exacerbated populational polymorphism? Stretching the argument a lot, is the difference in gene complement between female and male mammals (due to a small Y chromosome) also considered a pangenome? This does not diminish the findings in this study, the presence/absence polymorphism in these mussels is brutal and outstanding. But I am concerned that there is a trend towards coming up with new buzzwords that just apply to concepts that have been in the field for decades. I would like to hear the authors opinion in that subject.**

AU 2.2: This is indeed somewhat of a philosophical question and we agree about the need for the scientific community to come up with more appropriate terms to define what characteristics really define a “pan-genome” in the future. Studies like those suggested by reviewer #1, which extended pan-genome-like concepts to human and pig, do indeed mark a significant distancing from the original “public goods”-related definition, as the accessory genomic regions found in human and pig are, for the most part, associated with intergenic indels, with little or no known effect on the fitness.

In this paper we tried to associate the adjective “adaptive” to the noun “pan-genome” to clarify that this phenomenon has, in our opinion, an adaptive value in mussels. Moreover, we made clear that we also refer to the original “gene-centric” definition of the pan-genome provided by Medini and colleagues, which has been only later linked with inter-genic regions, with the first pan-genomic investigations carried out in Eukaryotes. Although the added value of dispensable genes in providing an increased resilience to biotic and abiotic stress is quite hard to be demonstrated with experimental evidence in mussel (as it was in the case of *E. huxleyi*), this can be hinted at from the statistically significant functional enrichment of dispensable genes in specific gene families and conserved domains. The finding that many dispensable genes were linked with antimicrobial activity, PAMP recognition, apoptosis and other related processes seem to point towards an increased ability to cope with the challenges posed by a mutable sea environment. With this respect, we need to keep in mind that mussels are sessile animals, which live in close contact with each other. Hence, a high standing genetic variability might provide a benefit for the fitness of other individuals living in the same community, e.g. by contributing to limit the spread of a disease. In this sense, we see a parallelisms between the mussel pan-genome and the examples provided above by the referee about bacteria and unicellular eukaryotes, and we agree about the fact that the classical definition of pan-genome most certainly does not apply to human or pig, as no adaptive value has been demonstrated so far for vertebrates. A very interesting paper has been published between the original submission of this manuscript and this resubmission, which linked the rapid ability of *M. galloprovincialis* to adapt to ocean acidification and high standing genetic variation in natural populations (see Bitter et al., Nature Communications 2020). We believe that our findings may provide a logical explanation to the observations reported by Bitter and colleagues. Although defining where to draw a line between what can or cannot be considered a pan-genome might be a challenging task, and we realize that many cases might still fall in a “grey area”, it is our opinion that the “pan-genome” concept should be used as much as possible in conjunction with its original meaning, i.e. with what we here called “adaptive pan-genome”.

**2.3 Other than this minor philosophical disagreement, "pangenomes" have been often associated with the activity of transposable elements. I wonder if the analyses show any evidence of jumping genes activity in these genomes. Also, there is no discussion on the possible mechanisms that allow the dispensable genes to arise.**

AU2.3: Thank you for this suggestion. This was one of the points that we planned to address in our original plans for the manuscript, but was later dropped, mostly due to our uncertainly about the confidence of the analysis, due to the relatively high level of fragmentation of the genome assembly. We have incorporated these results in the supplementary materials of the revised version of this manuscript, along with a brief mention in the main text. Although, due to the aforementioned fragmentation issue, these results should be taken with a grain of salt, we could not find any evidence that dispensable genes in mussel are associated with any particular element found in the custom repeat library we constructed. Indeed, none of such elements were found to be significantly enriched in the regions flanking dispensable genes, compared with the genome background. We believe that this aspect deserves more attention, and that a definitive answer to this question will be only made possible by the release of a chromosome-scale genome assembly.

**2.4 Importantly, the study makes no mention of the gender of the individuals selected. I believe these mussels have separated sexes, but I have no idea about the basis of their sex determination. Is it possible that all or a part of these dispensable genes are really sex-linked genes?**

AU2.4: This information went probably lost in the supplementary information, so we made sure to clarify this aspect upfront in the main text. *M. galloprovincialis* does indeed display separate sexes, and the sex of individuals can be determined by the morphological analysis of the mantle tissue, as long as the animals are sufficiently sexually developed (as this tissue holds the gonadal foci). While the individual used for the generation of the reference genome was female, we tried to obtain an even number of male and female mussels from subsequent re-sequencing (i.e. 6 males and 6 females). Finally, we sampled two additional male individuals (GALM6 and GALM11) to test whether the PAV patterns could be linked with the status of sexual maturation of the gonads. The mechanism of sex determination in mussels in still not entirely clear, but it most certainly partly depends on nuclear alleles (citing Yusa at al, Heredity 2013: “*sex in Mytilus is controlled by a pair of nuclear sex ratio alleles expressed in the mother and by minor sex-determining genes inherited from the father and also possibly from the mother*”). All re-sequenced mussel genomes were clustered via Bayesian inference based on the presence/absence patterns of dispensable genes (see supplementary material), but we could not detect any relevant effect of sex on the clustering of individuals (whereas a weak effect of clustering was due to the geographical origin of the populations). In conclusion, we could not collect any evidence that suggest that the sex of the individuals has any correlation with the presence or absence of certain dispensable genes. On the other hand, we found that sex had an apparent effect on the uniformity of the distribution of the mapped reads, as extensively discussed in the supplementary material.

**I have some other comments and questions that I hope the authors can kindly address:**

**2.5 In the significance statement, page 5 line 47, "large-scale structural variations which involve the loss of genomic regions encoding protein coding genes in some individuals (i.e. presence/absence variation, PAV)". In a similar manner to indels, I wonder if these changes can really be polarised. Are they really "losses" of regions, or just "absence" (ancestral or derived)?**

AU2.5: We still do not have a clear answer for this intriguing question, since the mechanisms that originate the PAV phenomena are not entirely clear yet and multiple alternative scenarios may be invoked. We understand that the reviewer means whether we can distinguish whether dispensable genes derive from relatively recent gains or from losses of genes once present in all individuals. We believe the most parsimonious explanation to point towards the “gene loss” hypothesis, which could be consistent with the empirical observation that along long evolutionary timescales, gene loss prevails over gene gain (see for example Wolf and Koonin, 2013). In mussel, dispensable genes often correspond with hemizygous regions which are diploid in other individuals. However, in many cases where dispensable genes occur with a very low frequency (e.g. those that were only found in just 1 or 2 of the 15 re-sequenced individuals), the discrimination between ancient losses and recent gains is not possible. This inference is further complicated by the fact that nearly all dispensable genes are part of expanded multigene families, and that nearly no cases of paralogous core and dispensable genes found in a 1:1 ratio exists, which did not permit to track the evolutionary history of these sequences in detail.

**2.6 Figure 1 and associated analyses, I wonder why the study left out Crassostrea gigas, which next to Lottia is possibly one of the best sequenced and annotated mollusc genomes. Moreover, there are dozens of developmental transcriptomes associated to it, which could be very helpful to track the functions of some of the genes of interest.**

AU2.6: This choice was motivated by the fact that the *Crassostrea gigas* genome assembly is inferior in terms of completeness and overall quality than that of *Crassostrea virginica*, which reaches a chromosome-scale level. Therefore, we used *C. virginica* as a representative of the oyster lineage. The observation about the availability of a high number of RNA-seq datasets for *C. gigas* is correct and we have indeed used them in a number of previous studies (see for example Gerdol et al. DCI, 2015 and 2017). In this particular case, we did not need these datasets for assigning tentative functions to mussel dispensable genes, as most of them lack significant primary sequence similarity with oyster (i.e. they are young and the product of lineage-specific *de novo* gene gains). However, we made extensive use of the multiple RNA-seq datasets available for many tissues of *M. galloprovincialis*, that we discussed in the supplementary materials.

**2.7 Use of term "phylostratigraphy", I wonder if the analyses here really use the same approach developed by Domazet-Lošo, Tautz and others (e.g., one way BLAST from a single "query" species against a depauperate taxon sampling of other taxa, assuming one to one relationships from the BLAST output). This approach has major flaws that have been criticised in the literature (see papers by Moyers or Casola in MBE and GBE). Some of the authors of this study are world-leading experts on orthology assignment, so I guess they would avoid such methods to the best of their judgement. I think this is not the case, as a paper by Huerta-Cepas is cited. Mapping origin of traits to a node in the phylogeny based on the taxonomic presence of these traits and the evolutionary relationships of organisms is nothing new or that deserves a new term.**

AU2.7: Sorry for the confusion, we did not use the method developed by Domazet-Loso et al. Instead, we used the method described in Huerta-Cepas et al, which is based on the topological analysis of gene family trees. We have now removed the term “phylostratigraphy” from the manuscript.

**2.8 Figure 2B, the axis could use some labels.**

AU2.8: Thank you for pointing this out. Axis labels have been added.

**2.9 The paper keeps insisting that "pangenomes" have never been described in metazoans before, but then keep citing a paper on pangenomes of human African populations. I'd suggest to rephrase this to "never described in invertebrates". Otherwise, this is an unsupported claim of unprecedented findings.**

AU2.9: The referee is right and we are sorry about this claim, that has now been toned down and better explained (see also our reply to reviewer #1, AU1.2).

**2.10 Page 9, line 19, "most genes present in single-copy" I am not sure if the text refers to zygosity (alleles) or to orthologs (single-copy). If the first, this sentence would need rephrasing.**

AU2.10: Thank you for pointing this out. We have revised the text to clarify this sentence and other cases where we refereed to zygosity (i.e. we now either define such cases as genes with hemizygous/homozygous coverage, or genes found with one/two alleles in the reference genome).

**2.11 There is a disconnect between the sections of the paper describing the presence/absence variation and the function of the genes. I find the later comes too late. I'd humbly suggest to move up the section "PAV genes may be associated with survival one" before "The mussel pan-genome".**

AU2.11: Thank you for this suggestion. In agreement with the Editor, the manuscript had been originally submitted in a non-canonical format for Genome Biology for its initial evaluation (i.e. it lacked the usual subdivision between a methods, results and discussion sections). Following the suggestions provided by reviewer #3, who also suggested a re-organization and the inclusion of a brief methods section, the main text has been reshaped and we hope that the new structure of the manuscript now flows better, with a separation between the results and the discussion. Any suggestion for further improvement is welcome.

**REVIEWER #3:**

**3.1 This manuscript presents a new reference genome for the mussel Mytilus galloprovincialis, as well as resequencing data for 14 additional individuals. Based on the resequencing data, the authors conclude that there is evidence for extreme presence/absence variation (PAV) in M. galloprovincialis, with 45,518 genes identified in all individuals, called core genes, and 14,820 genes missing in at least one individual, called dispensable genes. The PAV phenomenon has been described in bacteria and plants, and to some degree in humans, but has not been described in other metazoans, and certainly not to this scale. This result, if verified, is highly significant and of interest to a wide range of biologists. However, there are a number of unresolved questions about the results as presented that should be addressed prior to publication.**

AU3.1: We thank the reviewer for recognizing the significance of our work and for suggesting useful revisions. We have followed her/his indications and provided additional data to further support our observations and solve the questions pointed out below. We hope that the reviewer will find these revisions satisfying.

**3.2 Although the main text is well written, I found it very difficult to interpret the results based on the main text alone. The supplement contains a massive amount of information that is challenging to parse. The essential information is all included, but because so much necessary information is only in the supplement, I wonder if this manuscript might be better suited to a longer-format article or journal. Much of the text of the main manuscript deals with ideas about the origins and function of PAV in mussels, most of which is largely speculative. While the discussed ideas are intriguing, I felt that documentation of the evidence for the PAV phenomenon should receive more space in the main text.**

AU3.2: Thank you for this suggestion. In agreement with the Editor, the manuscript had been originally submitted in a non-canonical format for Genome Biology for its initial evaluation (i.e. it lacked the usual subdivision between a methods, results and discussion sections). Based on this suggestion and on a similar comment made by reviewer #2, the main text has been reshaped and we hope that the new structure of the manuscript, which now contains a methods, results and discussion section, flows better. The present revised version leaves much more space to the discussion of the evidence in support for the PAV phenomenon. This part has been enriched by a new effort put into the reassembly of additional dispensable contigs (i.e., genomic regions not present in the reference assembly), and in their annotation (see the response AU 3.6). We believe that this new section, extensively discussed both in the main text and in the supplementary material, significantly strengthened our conclusions, providing a more accurate estimate of the size of the mussel pan-genome (which extends the haploid genome assembly size by 580 Mb) and bring evidence for the presence of about 5,000 additional dispensable genes not present in Lola.

**3.3 My background is in non-model animal genome assembly, and as such, I was less familiar with the PAV phenomenon and the processes used to identify it. I found a review entitled "Towards Plant Pangenomics" by Golicz, Batley, and Edwards extremely helpful in assessing the evidence for PAV in this manuscript. Those authors discuss the following four factors that have significant impact on pangenome analyses: assembly quality, annotation quality, orthologous gene detection, and selection of appropriate samples. The remainder of my review will discuss these four points.**
AU3.3: Thank you very much for this suggestion, we found the suggested publication very useful, as the paper by Golicz, Batley and Edwards and other related publications stimulated internal discussion and inspired new ideas for the novel analyses that we carried out during the revision.

**3.4 Assembly Quality.**

**The new reference genome for Mytilus galloprovincialis, assembled using a multi-step hierarchical strategy with both long and short reads, has considerably better assembly statistics than previous versions. The assembly methods are quite thorough, and the authors employ numerous strategies to counteract various potential issues. I applaud the authors' thorough reporting of the multiple assemblies in the supplement. Understandably, the different approaches lead to assemblies with different potential problems. For example, the mg10 assembly has a less complete CEGMA/BUSCO gene set, but fewer duplicated genes.**

AU3.4: We thank the reviewer for his/her positive assessment of the work carried out about this challenging task.

**3.5 The results of downstream analyses are conducted using different assemblies, and it can be difficult to keep track of which assembly was used for a given analysis, particularly in the main text. For the most part, this is not likely to be a big issue, given that the assembly stats that are used in different contexts are reasonably similar, and the supplement does include these details most of the time. That being said, a flow chart or similar diagram might help the reader keep track of which assembly (and subsequently, which annotation), is being used for downstream analyses.**

AU3.5: We thank the reviewer for this comment. During the revisions, we noticed that the text was often not clear about the version of the assembly that was analyzed. We took particular care in updating this information in this revision, in particular in the supplementary material (the different versions of the assembly are not cited, for simplicity’s sake, in the main text). In the overwhelming majority of cases, the mg9 or mg10 versions were used. These two assembly versions are virtually identical, with the exception of a single scaffold which was re-assembled due to our particular interest in the myticin gene family. Overall, the use of mg9 in a few analyses only led to negligible discrepancies compared with mg10, as just a dozen myticin genes included in this scaffold were re-annotated.

**3.6 The genomes of 14 additional individuals were resequenced with short-read sequencing. Theoretical sequencing coverage for resequenced individuals ranged from 28-46X.  In section 12.1 of the supplement, the authors state "the fragmented nature of the de novo assembled genomes (see Table S37), due to the use of short reads only (Illumina paired-end), prevented a validation approach based on genome re-annotation and a comprehensive comparative analysis of annotated genes between Lola and the 15 resequenced genomes." Ultimately, in my opinion this analysis is needed for the PAV result to be fully convincing. I appreciate that this is beyond the scope of the current manuscript, but in the absence of a more comprehensive comparative analysis, the presentation of the PAV results should be toned down.**

AU3.6: We agree that an annotation-based validation approach would score an important point in favor of our hypothesis. In the absence of the long-range information provided by PacBio reads, unfortunately the genome reassemblies did not allow an efficient re-annotation of all the core genes, as the majority of them resulted to be split between two or more contigs, with several missing exons contained in short contigs (discarded as they did not reach the minimum assembly length), or not assembled at all. Overall, we found the “map reads to reference” approach to be more efficient for evaluating the presence/absence of a given gene than whole genome re-assembly annotation.

While we recognize the limitations of this approach that were pointed out by the reviewer in his/her comment, we tried to use a complementary approach, focusing our attention on the dispensable part of the re-sequenced genomes, that we had completely overlooked and basically not analyzed at all in the original version of this manuscript.

We hope that the referee will appreciate this effort, which is a compromise between the ideal approach (i.e. complete reassembly, as suggested by the reviewer, which we cannot provide due to the high fragmentation of the assemblies) and our previous analysis (which only took into account the genes present in the reference genome).

We would like to thank once again the reviewer for indicating the “recursive reassembly” approach in the next comment, as we took inspiration from the suggested publication and run the analysis as follows:

1) We recursively mapped the reads from the re-sequenced genomes on the reference assembly and collected unmapped reads. For this purpose, we choose to rely on the mg3 “redundant” assembly, as it was revealed to be the most “complete” by BUSCO and was likely the most representative of the two haplotypes found in Lola.
2) Collected unmapped reads from each re-sequenced genome were *de novo* assembled to obtain putative “dispensable contigs”. This process was re-iterated until the unmapped reads from all re-sequenced genomes were collected and *de novo* assembled.
3) We removed a few contigs that displayed a significant number of mapped reads from the reference assembly, which may correspond to small genomic regions that had not been not included due to technical issues in the reference assembly.
4) We applied a very stringent decontamination approach to remove any potential trace of exogenous contamination. This was based on the expected coverage (contigs with an unexpectedly high or unexpectedly low coverage were removed), GC content (that may be indicative of GC bias), matching against known contaminants detected through ITS and COI screening, k-mer-based KRAKEN matches, and detection of significant sequence similarity with the reference genome assembly.
5) The set of re-assembled “dispensable contigs” that passed the decontamination process were annotated with the same pipeline previously used for the annotation of the reference genome. However, to avoid obtaining an inflated estimate of novel dispensable genes (i.e., those included in very short contigs, thereby corresponding to fragmented genes), a minimum length threshold was set for contig annotation. This was arbitrarily set as the median length of the dispensable genes identified in Lola.
6) We ran the PAV detection pipeline on the newly annotated dispensable genes, verifying that most of these were just present in a few of the re-sequenced genomes, coherently with the data previously collected.
7) We obtained functional enrichment data for the novel dispensable genes, highlighting that the most abundant annotations perfectly matched with the most enriched categories found over-represented in our previous analyses.

Overall, this process allowed us to obtain nearly 580 Mb of additional genomic sequence data, highlighting once again the extreme relevance of PAV in mussel, and the “open” nature of the mussel pan-genome compared with those of human and pig. The 5000 newly annotated dispensable genes displayed a frequency of distribution very similar to the one observed for the dispensable genes present in the reference genome and fell within the very same gene families that displayed a significant enrichment in the PAV gene set.

**3.7 One method that could be used to strengthen the results using the data already in hand would be an iterative assembly aimed at producing a non-redundant pan-genome assembly (described in Golicz, Batley, and Edwards 2016).**

AU3.7: We are extremely thankful to the reviewer for this suggestion, as it was the key towards a significant improvement of the results presented in this revised version (see the AU3.6 response above).

**3.8 Annotation quality.**

**The authors use a range of both ab initio and RNAseq-based annotation methods. The approach appears to be thorough, but I had difficulty interpreting the annotation results. Annotation was conducted using the mg8 and mg9 assemblies, not mg10, the assembly optimized to have fewer issues with gene duplications. I understand that this likely has to do with the timeline of the project, and the challenges of lengthy genome projects with large teams of scientists. However, given that duplications could have a significant impact on the conclusions of the paper, this could be a concern.** 

AU3.7: As explained above in AU3.5, we revised the text to clarify the usage of different assemblies. Basically, the differences between mg8, mg9 and mg10 only dealt with the reassembly of a single scaffold of interest (which included the myticin gene cluster). Although the annotation was carried out on mg8, it was updated to include the few genes (about a dozen) added in mg10. In principle, we agree with the reviewer about the potential issues that the retention of duplicated genes might cause in the interpretation of PAV. However, based on the interpretation of the results of BUSCO, we observed that the gene duplication rate was relatively low, i.e. 8.5%, quite in line with the rate observed in many other bivalves and lower, for example, than the duplication rate observed in the chromosome scale assembly of *Crassostrea virginica* (see the supplementary materials). Hence, while we can predict that a few annotated genes may represent actual assembly artefacts, we argue that their number is not expected to be particularly high. In addition, we have used a particular strategy to tackle the potential issues linked with duplicated genomic regions in our PAV detection pipeline, that we realize might have gone unnoticed among the vast amount of information presented in the supplementary material. Specifically, we set the MQ parameter of BWAmem to 0, in order to allow multiple mappings, thereby avoiding the detection of genes with an artificial coverage = zero, that could have therefore been wrongly assessed as “absent”. In other words, duplicated genes would show a higher coverage than expected (e.g., 60X in a genome sequenced with 30X sequencing, while without this option one gene would have been characterized by a coverage = 60X and the other one equal to 0X). In light of this observation, we can affirm that artificial gene duplication (which is certainly present to some extent in the assembly despite out efforts) should not have a relevant impact on our PAV detection results.

**3.9 Additionally, I found it very difficult to keep track of the predicted gene sets, and what method was used to generate them. For example, Supplementary Table S8 summarizes functional annotation results based on assembly mg8. Perhaps I missed something, but it was not clear to me how the numbers in the top two lines of this table were generated. The top line of Table S8 gives 67,440 genes and 85,682 proteins in the MGAL8A annotation.  Section 2.4 states "the final MGAL9D annotation is made of 58,125 genes, whose 79,966 transcripts encode 75,788 unique protein products." The main body of the text says there are 60,338 protein-coding genes in the Lola genome. I cannot determine the source of this 60,338 number. The PAV result is dependent on appropriate gene prediction, and as such, this section should be much clearer.** 

AU3.9: We are sorry about these inconsistencies, that were the product of typos, inclusions of bits of text from previous versions of the text and lack of clarity concerning the version of the assembly used for a given task. We have carefully revised the text and the supplementary material to remove these issues.

**3.10 Orthology determination.**

**Figure 1 and supplementary data note 5 deal with phylome analyses indicating that there is a high gene duplication rate in the Mytilida lineage, with 23,815 genes inferred to have been duplicated in the LCA of Mytilida. There are even more duplications in Mytilus, with 35,027 genes inferred to have duplicated after Mytilus split from the rest of Mytilida. I have a few concerns about this analysis. First, it is not indicated which gene set was used for the phylome analyses. Given the variation in numbers of genes between sets, this information is needed.**

AU3.10: We are sorry for this. All the phylogenomic analyses correspond to the phylome that uses the genes predicted from the genome assembly of Lola (mg10) as seed (see Materials and Methods). PAV genes were annotated by mapping reads from other individuals against Lola and thus, genes absent in Lola are not present in our phylome dataset. We have now made this information explicit in the main text.

**3.11 Secondly, these analyses are based on the assumption that the gene numbers are not inflated. If the numbers of predicted gene models were inflated somehow, the pattern would likely look like 'multiple lineage-specific duplication events that took place after the split between Mytilus and the rest of Mytilida".  Over 9,000 genes are members of large expansions with >20 in-paralogs, which could alternatively be the result of gene modeling errors. In other areas of the manuscript, it is mentioned that there are large numbers of genes of unknown function in these large Mytilus-specific duplications. I would be very interested to see some additional data on these large duplications, including a few example phylogenies of the gene family expansions, to help verify that these in-paralogs are not redundant (i.e., they are in fact all unique duplication products). I'd also like to see a further breakdown of the cluster sizes for the gene clusters included in the >20 cluster size bar in Figure S17. What is the highest number of in-paralogs for a single cluster?**

AU3.11: The inferred clusters are obtained from the analyses of all the gene trees included in the phylome. Our pipeline generates trees from each gene in the seed genome and the corresponding homologs in all species compared (including the seed), and thus the gene trees produced from paralogous genes can significantly overlap. The observed paralogous clusters are obtained from the analysis of several trees and thus no individual phylogeny necessarily contains all of them, especially for the largest ones. One can think of it as collections of genes, across many different trees, all of them forming monophyletic groups with different degrees of overlap with only *Mytilus* species. We have modified figure S17 to better represent the data for clusters larger than 20. The largest number of in-paralogs corresponds to a cluster including 309 genes. We have clarified these results in the text and show two key examples of relatively large and expanded gene families, enriched in PAV genes, whose phylogeny suggests that the overwhelming majority of such genes are note redundant (i.e. they are not the product of residual duplicated genomic regions in the assembly). See supplementary data note 19.2.

**3.12 Selection of appropriate samples.**

**The selection of 14 individuals from separate populations in Spain and Italy is likely to provide a good baseline for understanding the pan-genome in M. galloprovincalis.**

AU3.12: We thank the reviewer for this positive assessment. As mentioned in one of the replies to a question provided by reviewer#1, the two populations include the two main evolutionary lineages recently identified by Bierne and colleagues, i.e. the “Atlantic” lineage and the “Mediterranean” lineage.

**Other notes:**

**3.13 Given that there are multiple publications about the human pan-genome, the subtitle "first evidence of a pan-genome in Metazoa" is inaccurate.**

AU3.13: We agree about this point and we have toned down this claim. We now focus on the first evidence of an adaptive pan-genome, as well as on the first evidence of massive gene PAV in a metazoan. Please note that the manuscript title has been changed to put more emphasis on the gene PAV aspect (“Massive gene presence/absence variation in the Mediterranean mussel shapes an adaptive open pan-genome”).

**3.14 In supplementary data note 18, one of the key examples of PAV is EF1a-bis, a dispensable pseudogene paralogous to EF1a. Based on the way the main manuscript is written, I expected the dispensable genes to be functionally transcribed genes. As such, I was surprised to see a pseudogene as a key example. I tried to determine from the supplement the percentage of dispensable genes that are identified as pseudogenes, and was unable to do so. The concept of "core pseudogenes" is also discussed in the supplement in the context of the canonical mytillin cluster. Are these "core pseudogenes" included in the 45,000 core gene set?**

AU3.14: The reviewer is right, and the implications the pangenomic structure of the mussel genome has on pseudogenes and other non-coding genomic elements were not discussed at all in the previous version, so “core pseudogenes” were not part of the 45K core gene set. We have added a new Supplementary Data Note (#9), accompanied by figures and tables, which show PAV data for non-coding genes as well.

In brief, since PAV is linked with relatively large structural variations, the underlying unbalanced indels may target pseudogenes as well, and in several parts of the supplementary material we provide evidence about this observation, in particular for what concerns AMP genes. We have re-run the PAV detection pipeline to extend the description of the PAV phenomenon to non-coding genes, revealing that a significant fraction of them is subject to PAV. We might speculate that this derives from the fact that several protein-coding dispensable genes, in spite of the presence of a continuous ORF, display a very low expression level, which may suggest that they are undergoing pseudogenization (e.g. though the progressive loss of a functional promoter and other regulatory regions). This would be consistent with the “reduced essentiality” features previously described by other authors for dispensable genes in plants.

**Very minor comments:**

**3.15 Out of only curiosity: why is the individual called Lola?**

AU3.15: This was simply a follow-up to the previous paper by Murgarella and colleagues, where the female mussel sequenced was named Pura. Pura and Lola are two common nicknames in Spain, so they were chosen to label these two female mussels sampled in Galicia with a “catchy” nickname, as a callback to their geographical origins.

**3.16 Supplementary Data Table 50 can't be read in the current version- all the symbols show up as a box with a question mark.**

AU3.16: Thank you for pointing this out. This issue has been fixed.

**Second round of review**

**Reviewer 1**

The authors have generally done an excellent job of responding to my critiques and those of the other referees of the original version of BIO-D-19-01408R1. They have added new data and analyses where necessary as well as improving their scholarship and data interpretation.
    I still have one major criticism: there is no direct evidence presented here that the gene presence/absence variation (PAV) that the authors described is adaptive. Such evidence would require assessments of phenotype and fitness in the context of different PAV genotypes and then demonstration that particular PAV genotypes are associated with distinct phenotypes and higher fitness in a particular context. in the absence of this information, while the authors can certainly speculate about whether the PAV that they report is adaptive, it constitutes a serious overinterpretation to describe this variation as "adaptive" in the title as well as throughout the abstract and other parts of the manuscript. Instead, the authors should focus on the facts of what they observed, stated very nicely on P. 11, lines 53-55: "...an "open pan-genomic architecture with a high rate of dispensable to core genes".

Specific Comments:
P. 3, Line 43, and other places in the MS: Please hyphenate "whole genome" where this phrase occurs in front of another noun (here, "scale"). Same applies to other sequential uses of noun/adjective combinations.
P. 4, Line 57, and other places in the MS: Please DO NOT hyphenate word combinations (like here, "randomly-mating") that include an adverb.
P. 5, line 16: Add comma after "generations".
P. 5, line 20: Exchange "experimenting" with "experiencing".
P. 5, line 50: Word missing here in "role played"?
P. 6, line 21: Insert comma after diseases".
P. 6, line 42: Missing word before "phenomenon"?
P. 7, line 20: Missing "d" after "sequence"?
P. 8, line 30: Hyphenate "one third".
P. 11, line 4: Add a comma after "BIR)".
P. 20, line 7: Missing a word between "allowed to".
P. 21, line 34: "of" instead of "or".
P. 21, line 48: Insert comma after "alterations".
P. 22, line 60: "amplicon", not "amplicons", and then fix plural vs. singular of "were" vs. "was".

**Reviewer 2**

I am satisfied with the changes to the manuscript and the author's responses. The new "format" of the paper is really better. I'd like to congratulate them for this excellent work. I am looking forward to following the adventures of Pura and Lola.

**Authors Response**

**Point-by-point responses to the reviewers’ comments:**

We would like to thank the reviewers for their useful reports and suggestions. We agree with the major criticism posed by reviewer #1 and we do not feel that sufficient experimental evidence has been provided at this stage to strongly claim that the nature of the mussel pan-genome is adaptive. While we discuss this possibility based on the enrichment of dispensable genes in functions associated with immunity and survival, we have toned down several statements that could not be fully supported. Most certainly, we will carry out additional experiments in the years to come to investigate the possibility that PAV is associated to particular phenotypic traits and that it can provide an evolutionary advantage under certain environmental conditions.

Please note that the title of this manuscript has been also modified to remove the work “adaptive”. The updated title now reads: “Massive gene presence/absence variation shapes an open pan-genome in the Mediterranean mussel”.

We are also attaching as a supplementary file the change of authorship request form, signed by all co-authors, to certify the acceptance of all contributors of the inclusion of Dr. Samuele Greco in the list of authors.

**Reviewer #1: The authors have generally done an excellent job of responding to my critiques and those of the other referees of the original version of BIO-D-19-01408R1. They have added new data and analyses where necessary as well as improving their scholarship and data interpretation.**

**I still have one major criticism: there is no direct evidence presented here that the gene presence/absence variation (PAV) that the authors described is adaptive. Such evidence would require assessments of phenotype and fitness in the context of different PAV genotypes and then demonstration that particular PAV genotypes are associated with distinct phenotypes and higher fitness in a particular context. in the absence of this information, while the authors can certainly speculate about whether the PAV that they report is adaptive, it constitutes a serious overinterpretation to describe this variation as "adaptive" in the title as well as throughout the abstract and other parts of the manuscript. Instead, the authors should focus on the facts of what they observed, stated very nicely on P. 11, lines 53-55: "...an "open pan-genomic architecture with a high rate of dispensable to core genes".**

AU 1.1.: We thank the reviewer for his/her positive assessment and we agree about the consideration about the lack of direct evidence in support of the adaptive nature of the PAV phenomenon at this stage. We will most definitely investigate this hypothesis with appropriate experiments in the near future.

The title of the manuscript has been amended by removing the word “adaptive” and now reads: “Massive gene presence/absence variation shapes an open pan-genome in the Mediterranean mussel”

The main text and supplementary materials have been carefully checked to remove and/or tone down some statements concerning the adaptive value of dispensable genes. The manuscript, in this revised version, presents the possible involvement of PAV in adaptation as a working hypothesis that needs to be formally tested in the discussion section. In particular, the following statement has been added to the discussion:

“At the present stage, in absence of experimental data linking phenotypic variation and fitness to PAV in different ecological contexts, this remains a working hypothesis that needs to be formally tested.”

**Specific Comments:**

**P. 3, Line 43, and other places in the MS: Please hyphenate "whole genome" where this phrase occurs in front of another noun (here, "scale"). Same applies to other sequential uses of noun/adjective combinations.**

AU 1.2: Thank you for this suggestion. We have carefully checked the text for such occurrences and we hope we have not missed any. Hopefully, other minor linguistic adjustments will be provided by the editorial assistance team during proofreading.

**P. 4, Line 57, and other places in the MS: Please DO NOT hyphenate word combinations (like here, "randomly-mating") that include an adverb.**

AU 1.3: Corrected.

**P. 5, line 16: Add comma after "generations".**

AU 1.4: Corrected.

**P. 5, line 20: Exchange "experimenting" with "experiencing".**

AU 1.5: Corrected.

**P. 5, line 50: Word missing here in "role played"?**

AU 1.6: Thank you. This should have read “role played by”. This sentence has been corrected.

**P. 6, line 21: Insert comma after diseases".**

AU 1.7: Corrected.

**P. 6, line 42: Missing word before "phenomenon"?**

AU 1.8: Thank you. This should have read “the gene PAV phenomenon”. This sentence has been corrected.

**P. 7, line 20: Missing "d" after "sequence"?**

AU 1.9: Corrected.

**P. 8, line 30: Hyphenate "one third".**

AU 1.10: Corrected.

**P. 11, line 4: Add a comma after "BIR)".**

AU 1.11: Corrected.

**P. 20, line 7: Missing a word between "allowed to".**

AU 1.12: Thank you. This should have read “allowed us to”.

**P. 21, line 34: "of" instead of "or".**

AU 1.13: Corrected.

**P. 21, line 48: Insert comma after "alterations".**

AU 1.14: Corrected.

**P. 22, line 60: "amplicon", not "amplicons", and then fix plural vs. singular of "were" vs. "was".**

AU 1.15: Corrected.

**Reviewer #2: I am satisfied with the changes to the manuscript and the author's responses. The new "format" of the paper is really better. I'd like to congratulate them for this excellent work. I am looking forward to following the adventures of Pura and Lola.**

AU 2.1: We would like to thank the reviewer for his/her positive assessment of our manuscript and for the helpful comments provided in the previous round of review, as we feel they were very helpful in improving the quality of this work.
